# Bicyclic Selenenyl Sulfides with Tuned Bioreductive Step Rates Reveal Constraints for Probes Targeting Thioredoxin Reductase

**DOI:** 10.1002/anie.202508911

**Published:** 2025-07-16

**Authors:** Lukas Zeisel, Lucas Dessen Weissenhorn, Karoline C. Scholzen, Andrea Madabeni, Laura Orian, Elias S. J. Arnér, Oliver Thorn‐Seshold

**Affiliations:** ^1^ Faculty of Chemistry and Food Chemistry TU Dresden Bergstrasse 66 01069 Dresden Germany; ^2^ Department of Pharmacy LMU Munich Butenandtstr. 5–13 81377 Munich Germany; ^3^ Division of Biochemistry, Department of Medical Biochemistry and Biophysics Karolinska Institutet Solnavägen 9 17165 Solna Sweden; ^4^ Department of Chemical Sciences University of Padova Via Marzolo 1 35129 Padova Italy; ^5^ Department of Selenoprotein Research National Institute of Oncology 1122 Budapest Hungary

**Keywords:** Chemical probes, Disulfides, Redox biochemistry, Selenenyl sulfides, Selenium

## Abstract

The reductive activation of dichalcogenide probes by thiol‐type oxidoreductases proceeds through a cascade of consecutive, partly reversible steps. Stereocontrol elements can modulate the reaction rates of these steps to reach substrate‐controlled kinetic selectivity for reductase chemotypes in live cells. We now deploy regio‐, diastereo‐, template‐, and pH‐control elements to shape the reactivity of unprecedented bicyclic selenenyl sulfides (**SeSP**), arriving at probes that selectively target the mammalian selenoenzyme thioredoxin reductase TrxR1. We accessed these densely functionalised *cis*‐ or *trans*‐fused 1,2‐thiaselenanes on gram scale over 5 steps by using a regioselective key step that elaborates an unusual, differentially protected 2,2′‐bis‐aziridine intermediate through sequential one‐pot chalcogen introduction and selenenyl sulfide formation. By profiling a set of regio‐ and diastereoisomeric bicycles for their partly or fully reversible reactivity during reductive activation, we show how effects that slow their reduction steps (addition then resolution) can compensate by vastly accelerating subsequent activation (cyclisation) speeds, such that cellular processing is effective and TrxR‐selective. More broadly, this study shows how multistep cascade probes can leverage conformational effects and internal noncovalent interactions to differentiate step kinetics along their on‐target versus off‐target reaction pathways, thus achieving reaction‐based target selectivity in complex biological settings.

## Graphical abstract

Thiol reductants activate dichalcogenide probes by a cascade of partly reversible steps. Careful dichalcogenide engineering can tune the rates of these steps separately, to aim at probes that are kinetically selective for specific oxidoreductases in live cells. Here, we deploy regio‐, diastereo‐, template‐, and pH‐control elements to shape the reactivity of bicyclic selenenyl sulfides, giving probes that selectively target the selenoenzyme TrxR1.

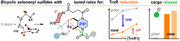

## Introduction

The thioredoxin / thioredoxin reductase (Trx/TrxR) and the glutaredoxin / glutathione / glutathione reductase (Grx/GSH/GR) systems are central to cellular redox homeodynamics.^[^ ^1^ ^]^ These relay systems use NADPH to balance many arms of redox chemistry via redoxin (Trx‐fold) proteins:^[^ ^2^, ^3^ ^]^ e.g., controlling metabolic processes including nucleic acid synthesis, thiol redox proteome switches, and antioxidant defense.^[^ ^4^ ^]^ Despite their key roles in biology, the Trx and Grx systems have long lacked high‐quality probes that could monitor and decrypt their activity in living cells. Conceptually, a suitable turnover probe could feature an artificial redox substrate that is selectively reduced by a specific vicinal dichalcogenide redoxin (Grxs, Trxs; or TrxRs), then fragments irreversibly to accumulate a non‐invasive readout (e.g., release a fluorophore). Yet, identifying substrates that allow fast and catalytic, specific on‐target processing, but are robust to other (di)thiol redox proteins or to monothiols despite their similar reduction chemistry, has been a major problem.

**Figure 1:**
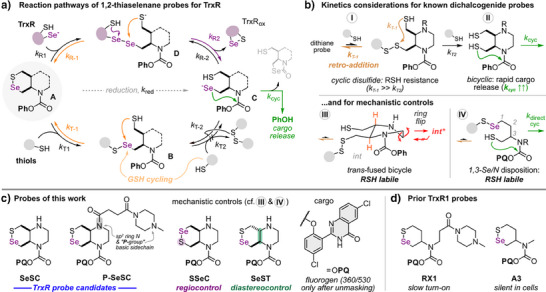
Probe design and mechanism. a) Reaction pathways of 1,2‐thiaselenane‐based probes, with their target selenolthiol enzyme TrxR or with off‐target monothiols. b) Features known for prior art probes for dichalcogenol reductases that affect inertness to reduction by monothiols and overall activation kinetics. Full details in Figure S1. c,d) The novel, piperazine‐fused selenenyl sulfide probes (**SeSC**, **P‐SeSC**), mechanistic controls (**SSeC**, **SeST**), fluorogenic probe cargo PQ‐OH (530 nm emissive only after precipitation as the unmasked phenol), and prior TrxR probes (**RX1**, **A3**).

TrxR's role and reactivity is driven by a functional group that is unique in the mammalian proteome: a cyclic vicinal selenenyl sulfide (‐RSeSR‐). Selenenyl sulfides are also key intermediates in TrxR's biochemistry with Trxs, and in the catalytic cycles of most of the −25 human selenoproteins such as the ferroptosis‐suppressing glutathione peroxidase GPx4. Yet, cyclic selenenyl sulfides are almost totally unstudied as a chemical class, with only 15 unique scaffold types ever reported (Scheme S1), only four of which were ever used in redox biochemistry. In this paper, we will be working with “**SeSP**” probes built around a novel chemotype of bicyclic selenenyl sulfide, that combines multiple structural features affecting the kinetics of various steps in their reduction pathways (Figure 1a: targeted TrxR pathway at the top, off‐target monothiol pathway at the bottom; features include *cis* ring fusion, piperazine annelation, 1,2‐Se,N disposition). The aim of this design was to accumulate kinetic selectivity so that the **SeSPs** would be selective reduction substrates of TrxR^[^ ^5^ ^]^ in the cellular context (low nM TrxR; and avoiding reduction by mid mM thiols), based purely on reduction kinetics (no role for binding affinity).

This design is the culmination of several generations of dichalcogenide redox probe development. Cyclic dichalcogenide substrates can be used to avoid reduction by millimolar cellular monothiols (two consecutive monothiol additions required) while selecting for micromolar vicinal dithiols (bimolecular reaction).^[^ ^6^ ^]^ This arises since their one‐thiol‐adducts have high effective molarity for intramolecular retro‐addition *k*_T‐1_ that leads to expulsion of the initial “attacking” thiol before a second “resolving” thiol can add intermolecularly (Figure 1b,I).^[^ ^7^ ^]^ Yet, with a single equivalent of a vicinal dithiol, retro‐addition *k*_R‐1_ can be out‐competed by intramolecular resolution *k*_R2_ to complete the reduction and trigger a readout (*c.f*. Figure 1a).^[^ ^7^ ^]^ This preference of cyclic dichalcogenides has been used in disulfide‐based probes for the most powerful vicinal dithiol redoxin Trx, where, guided by Whitesides’ disulfide studies,^[^ ^8^ ^]^ highly reduction‐resistant, *cis*‐decalin‐type dithianes have been applied.^[^ ^7^ ^]^

The *cis*‐decalin‐type dithianes were rather Trx‐selective in cell‐free settings, but were not effective in cells since after reduction they were intercepted by re‐oxidation (*k*_T‐2_) before cyclisation (*k*_cyc_) could occur.^[^ ^7^ ^]^ To accelerate cyclisation, the cyclising thiol was stabilised in its reactive thiolate state by a basic annellating piperazine: giving probes with orders of magnitude faster activation, while retaining the Trx preference (Figure 1b,II).^[^ ^9^ ^]^ Noteworthily, the *trans*‐fused dithiane analogues of both probe types were readily reduced by monothiols,^[^ ^10^ ^]^ since a single monothiol attack lets them access a conformationally trapped intermediate that no longer performs efficient retro‐addition (int*, Figure 1b,III).^[^ ^9^ ^]^

Trx's upstream reductase TrxR has also been targeted. This high‐turnover selenoenzyme has a broad substrate scope due to its unique vicinal selenolthiol active site (C‐terminal CU motif), exposed on a flexible tail at the protein surface.^[^ ^11^, ^12^ ^]^ Thus, a reactivity‐based, not binding affinity‐based, approach appears best suited to target TrxR's distinct active site chemistry. In prior work, we designed unsymmetrical 1,2‐thiaselenanes as novel and selective substrates of TrxR,^[^ ^13^, ^14^ ^]^ based on four mechanistic considerations (Figure 1b). In brief: 1) chalcogenols will attack the probe at its Se atom, much more rapidly than at S; and 2) Se‐attack is orders of magnitude faster for Se^−^ than for S^−^ nucleophiles (*k*_R1 _> *k*_T1_): which should cause very fast addition and reduction by Se‐TrxR.^[^ ^15^ ^]^ However, during attack by monothiols, 3) any second equivalent of monothiol encountering the intermediate should, similarly, react at Se; and 4) the increased rate of undesired monothiol addition to the probe's Se centre should be matched by accelerated intramolecular retro‐addition in the intermediate (*k*_T‐1_): which leads monothiols into “fast cycling” rather than full reduction, i.e., there is kinetic selectivity against monothiol reduction. This mechanism‐based design was validated by the monocyclic selenenyl sulfide **RX1** (Figure 1d), the first cellularly‐selective probe for TrxR.^[^ ^13^ ^]^ The kinetics‐based design for selectivity was supported by computational studies,^[^ ^16^ ^]^ and since inverting the ‐SeS‐ regiochemistry to ‐SSe‐ (1,3‐Se/N controls) were GSH‐labile despite having identical reduction thermodynamics (rationale: because thiol‐carbamate cyclisation directly after monothiol addition is now possible; Figure 1b,IV).^[^ ^13^ ^]^

Despite its TrxR selectivity, **RX1** is a sub‐optimal probe since its slow response kinetics require cell‐line‐specific delay times of 20–90 min before analysis. This reduces spatial localisation of the readout, introduces uncertainties, limits overall observation times, and prevents repurposing its selenenyl sulfide to gate the release of other (e.g., electron richer) cargos.

Therefore, we now sought to boost the cellular performance of TrxR‐targeting probes, by pioneering bicyclic selenenyl sulfides with a local base for accelerated cyclisation (**SeSC**, Figure 1c), aiming to improve response kinetics without losing selectivity. This design combines (**I**) a cyclic 6‐membered 1,2‐dichalcogenide to select for reduction only by vicinal dichalcogenol oxidoreductases, (**II**‐**III**) a *cis*‐decalin‐type architecture to boost monothiol resistance, with a piperazine backbone to boost post‐reduction cargo release, and (**IV**) a thiaselenane with a 1,2‐Se/N disposition for TrxR selectivity and inertness to monothiols. We also aimed to test the generality of this cumulative design logic, by making control compounds that subvert these aspects one at a time (e.g., 1,3‐Se/N probe **SSeC**, and *trans*‐fused **SeST**), or that add further performance (*N*‐methyl piperazinamide “P” group in **P‐SeSC**, a group that was found to improve both reduction and cyclisation kinetics for prior art probes, e.g., **A3** versus **RX1**).^[^ ^13^ ^]^ The design uses PQOH (6‐chloro‐2‐(5‐chloro‐2‐hydroxyphenyl)quinazolin‐4(3*H*)‐one) as a fluorogenic cargo that is released after *5‐exo‐trig* cyclisation onto the carbamate, then fluoresces^[^ ^17^ ^]^ (for further design details, incl. resistance to alternative cargo release mechanisms such as hydrolysis, see Supporting Information section 1).

## Results and Discussion

### Synthesis

**Figure 2:**
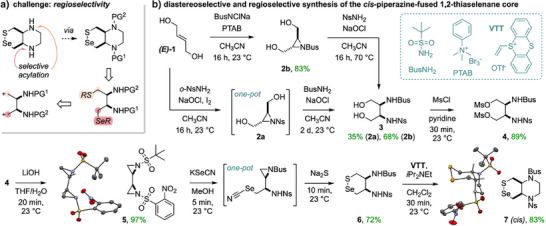
Synthesis. a) The key challenge: developing a 1,4‐pseudosymmetric precursor to introduce the chalcogens then acylate the amines with full regioselectivity (PG = protecting group). b) Diastereo‐ and regioselective synthesis of piperazine‐fused 1,2‐thiaselenanes. The 5‐step, scalable sequence to *cis*‐piperazine fused 1,2‐thiaselenane **7** (for probes **SeSC** and **P‐SeSC**) was readily adapted for *trans*‐fused analogue **7T** (for control **SeST**; see Figure 4). (Non‐oxidising bases can replace NaOCl in the aziridine‐opening steps to **3**: see Table S3; for other details).

Accessing piperazine‐fused 1,2‐thiaselenanes (**SeSP**s) faces two challenges: ring fusion diastereoselectivity (*cis*/*trans*), and regioselective chalcogen installation (SeS/SSe), that both were predicted to determine the biological selectivity. For diastereoselectivity, we could adapt routes to *cis*‐/*trans*‐piperazine‐fused disulfides that we previously^[^ ^9^ ^]^ built by subjecting *E*/*Z*‐olefins to Minakata's 1,2‐*anti*‐diamination then Ritter's 1,2‐bis‐electrophile vinyl thianthrenium (VTT).^[^ ^18^, ^19^ ^]^ However, we found no precedent to regioselectively access the desired Se‐proximal probes (**SeSC** type) rather than the Se‐distal control (**SSeC** type), so developing a regiopure synthesis became our main focus. We aimed to set up orthogonal *N*‐protecting groups (PG) in a common precursor (**7** *cis* or **7T** *trans*), that could be removed selectively: by replacing PG^1^ to create Se‐proximal probes, or PG^2^ for Se‐distal controls, respectively (Figures 2a,b and 4b).

A good synthon for **7** would be a 2,3‐diamine‐1,4‐dication with differentiated *N*‐protecting groups and differentiated cation reactivities for regioselective chalcogen installations (Figure 2a). Bis‐electrophiles such as 1,4‐dimesylates with orthogonal 2,3‐*N*‐protecting groups match this pattern, but we reasoned that the rather long‐range steric or inductive effects from the protecting groups (in *β* to the electrophilic carbons) were unlikely to deliver enough regioselectivity for our needs. Instead, we envisioned differentially protected bis‐aziridines of type **5** as key intermediates for efficient and regiocontrolled (protecting groups in *α*) chalcogen installation by nucleophilic ring openings;^[^ ^20^ ^]^ indeed, varying the *N*‐substituent of an aziridine is well known to modulate its S_N_2 reactivity over a wide range,^[^ ^21^, ^22^ ^]^ due to e.g., leaving group p*K*_a_, *N*‐hybridisation, and FMO geometries.^[^ ^23^ ^]^

Our first choice for the differentiated *N*‐protecting group pair was Boc and *o*‐Ns. They provide a large difference of electrophilicity (*c.f*. N─H p*K*_a_);^[^ ^24^ ^]^ a route to (*N* ^2^‐Boc, *N* ^3^‐Ns)‐2,3‐diaminobutane‐1,4‐diol, which would be a suitable precursor for the bis‐aziridine, was recently established;^[^ ^9^ ^]^ and *S*‐nucleophilic ring opening of Boc‐aziridines was shown to succeed under mild conditions.^[^ ^25^ ^]^ Indeed, we were able to isolate the Boc/Ns‐bis‐aziridine analogue to **5** (Figure 2b), and chalcogenide addition tests towards analogues of **6** proved the viability of this strategy (Tables S1 and S2). However, the Boc group caused practical problems including aziridine instability, intermediate decomposition, and rather low yields, and it also hampered later piperazine assembly with VTT.^[^ ^9^ ^]^

Thus, we next tested Weinreb's *tert*‐butylsulfonyl (Bus) as a sulfonamide alternative to Boc.^[^ ^26^ ^]^ Bus still gives a large p*K*_a_ difference compared to NsN‐H, and allows Ns‐orthogonal deprotection under acidic conditions. We found BusNH_2_ to react well in Minakata's 1,2‐diamination.^[^ ^18^ ^]^ Starting from (*E*)‐2‐buten‐1,4‐diol (**1**), Ns‐aziridination (**2a**) then in situ ring opening with BusN(Cl)Na gave Bus/Ns‐diamine **3** scalably and in satisfactory yield. Alternatively, the order of PG‐NH_2_ introduction could be switched: first isolating the Bus‐aziridine from Sharpless’ olefin aziridination,^[^ ^27^, ^28^ ^]^ then opening with NsNH_2_, in a higher‐yielding albeit two‐step process to **3** (Figure 2b).

After bis‐mesylation of diol **3**, we applied the optimised conditions for sulfonamide aziridine formation (Table S1, entry 5: slight excess of LiOH in THF/H_2_O), giving Bus/Ns‐bis‐aziridine **5** in near‐quantitative yields, without alcohol byproducts. Crystallising **5** confirmed its *cis*‐diastereochemistry and showed its unusual double‐triangular architecture (Figure 2b). Following Maligres’ study on the high reactivity of Ns‐aziridines,^[^ ^29^ ^]^ we reasoned that a single equivalent of chalcogenide would react regioselectively at the nosyl aziridine. Indeed, treatment with KSeCN rapidly gave the Ns‐opened intermediate as the sole product (Figure 2b). Adding Na_2_S in one pot gave the monocyclic 1,2‐thiaselenane **6** cleanly after 10 min, by a rapid double nucleophilic substitution: first displacing cyanide from the Se centre, then intramolecularly opening the Bus‐aziridine (the order of attack is supported since reacting the isolated selenocyanate intermediate with a monothiol gave only the acyclic selenenyl sulfide; Figure S2). Finally, reacting 1,2‐bis‐sulfonamide **6** with VTT triflate^[^ ^19^ ^]^ gave the **SeSP** (bicyclic *cis*‐piperazine‐fused 1,2‐thiaselenane) **7** as a key precursor to the target *cis* TrxR probes, with an X‐ray structure confirming the regioselectivity of chalcogen introduction (Se proximal to the Ns‐amine).^[^ ^30^ ^]^ The *trans* diastereomer control **7T** was synthesised similarly (higher yield since diamination was more efficient; Figure S2). Overall, this scalable sequence gave the densely functionalised, redox active *cis* and *trans* **SeSP** motifs in five steps, with ∼030% overall yield, after only three chromatographic purifications (Figure 2b).

### Computational Studies

**Figure 3:**
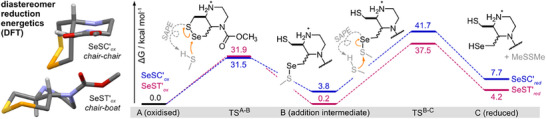
DFT calculations. Lowest‐energy structures and conformations of oxidised and reduced SeSC* and SeST* are shown together with activation and reaction energies (kcal mol^−1^) for their two‐step monothiol reduction using methyl thiol as nucleophile. The asterisk indicates that calculations were conducted on model probes in which the amine is protonated, and which feature a simplified *O*‐methyl carbamate instead of *O*‐PQ. All transition states were modelled via a solvent‐assisted proton‐exchange (SAPE) fashion, using two water molecules to mediate the proton transfer.

We next conducted DFT calculations to study key aspects of the reactions of *cis*/*trans*‐**SeSP**s with thiols or with selenolthiol TrxR (Figure 1a). Geometries for the bicyclic **SeSP** probes (Figure 3) matched the chair‐chair conformation found experimentally for SeSC* (Figure 2b) while providing a chair‐boat for SeST*. These conformations parallel those of the near‐isosteric bicyclic disulfides,^[^ ^9^ ^]^ for which it was supported that a chair–chair conformation makes a probe (here, **SeSC**‐type) inert to monothiols due to fast retro‐addition (Figure 1b,I), while chair‐boat probes (here, *trans*‐fused **SeST**‐type) are instead monothiol‐labile since thiol addition that opens the dichalcogenide can be followed by a boat⇄chair piperazine equilibrium, disfavouring the boat‐based retro‐addition (Figure 1b,III).^[^ ^9^ ^]^ Energies for **SeSP** reduction steps support that initial nucleophilic addition occurs only at Se^[^ ^13^, ^15^, ^16^ ^]^ and has similar ΔG^‡^ for both diastereomers. However, subsequent reduction rather than re‐formation of the *cis*‐**SeSP** (ΔG^‡,B⟶C^ – ΔG^‡,B⟶A^ = +10 kcal mol^−1^) is predicted to be more challenging than for the *trans*‐**SeSP** (+5.6 kcal mol^−1^): supporting the *cis* species' greater inertness to activation by monothiols (Figures 3 and S8–S10).

### Probe Assembly

**Figure 4:**
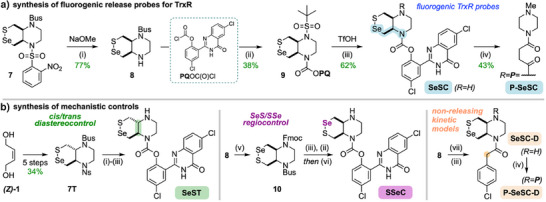
Probe synthesis (details in Figures S5 and S19). a) Synthesis of TrxR probes **SeSC** and **P‐SeSC**. (i) NaOMe (5.4 M in MeOH), THF, 0 → 23 °C; (ii) PQOC(O)Cl, DMAP, CH_2_Cl_2_, 23 °C; (iii) TfOH, *o*‐anisole, CH_2_Cl_2_, 23 °C; (iv) succinic anhydride, DMAP, Et_3_N, DMF, 80 °C, then TSTU, Et_3_N, then *N*‐methyl piperazine, 23 °C. b) Diversification of **SeSP**s to control probes **SeST**, **SSeC**, and non‐release phenylacetamides. (v) FmocCl, pyridine, CH_2_Cl_2_, 0 → 23 °C; (vi) piperidine, DMF, 23 °C; (vii) (COCl)_2_, 4‐chlorophenylacetic acid, DMF, 23 °C.

To test the selenenyl sulfides' performance as reducible motifs for probes, and especially whether **SeSC**‐type probes are improvements over the only current cellularly‐effective TrxR probe **RX1**, we diversified **7** and **7T** into fluorogenic **SeSC**‐ and **SeST**‐type probes, respectively (Figures 4 and S5). For 1,2‐Se,N‐type probes (aim: TrxR selective), *o*‐Ns deprotection is needed as a first step. However, standard thiolate conditions led to SeS‐bond cleavage then intramolecular S_N_Ar to the nitroaryl selenoether. Ns cleavage with non‐chalcophilic NaOMe instead was successful after some optimisation, for both **8** and **8T**. Reacting **8**/**8T** with PQ‐OCOCl to give the carbamates, then Bus‐deprotection with TfOH,^[^ ^26^ ^]^ gave *cis* probe **SeSC** and *trans* diastereocontrol **SeST** (Figure 4). **SeSC** was easily derivatised further to **P‐SeSC** with succinic anhydride, then TSTU and *N*‐methyl piperazine. In contrast, access to the 1,3‐Se,N regiocontrol **SSeC** was less direct, since after Bus‐deprotection and PQ carbamate installation, the intermediate's Ns group could not be removed without cleavage of the carbamate. Thus, we exchanged **8′**s Ns protecting group for an Fmoc, which then allowed straightforward synthesis of **SSeC** (Figure S5).

### Chemical Reductant Assays

**Figure 5:**
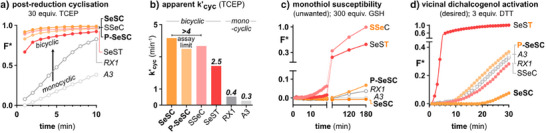
In vitro tests with chemical reductants. a,b) Selenol cyclisation kinetics are rate‐limiting for signal generation by monocyclic probes in TCEP assays, but bicyclic probes cyclise so fast that they surpass the assay resolution (discussed at Figure S13). c) Reactivity with high concentrations of monothiol GSH. d) Reactivity with vicinal dithiol DTT. (F* = F(t)‐F(0); full data and processing in Figures S11–S16).

To start evaluating their activation mechanisms and step kinetics, we first challenged all probes with the strong trialkylphosphine reductant TCEP.^[^ ^31^ ^]^ TCEP rapidly, quantitatively and irreversibly reduces selenenyl sulfides, which typically leaves on‐carbamate cyclisation as the rate‐determining step for signal generation by cargo release (*k*_red_[TCEP] >> *k*_cyc_, Figures 1a and S13a). The unusual cargo PQ‐OH only generates signal after it also precipitates, so the apparent rate constant for signal generation (*k'*_cyc_) actually convolutes cyclisation (*k*_cyc_) with precipitation rates (ca. 10 s timescale). PQ‐OH precipitation is usually so much faster than cyclisation, that previous probes could equate *k'*_cyc_ = *k*_cyc_; however, the bicyclic **SeSP**s cyclised so fast after reduction, that their *k'*_cyc_ values hit the precipitation rate plateau of this assay (apparent halflives ca. 10 s): a stark contrast to the prior monocyclic selenenyl sulfide probes **RX1** and **A3** ^[^ ^13^ ^]^ which do not benefit from annelation preorganisation (halflives ca. 5–10 min; Figure 5a,b; see discussion at Figure S13c). With the bicyclic **SeSP**s thus seeming to cyclise at least > 1–2 orders of magnitude faster than previous **RX1**, we had hopes that they might prove to be more effective as cellular TrxR probes.

Next, we tested the probes’ inertness to monothiols, which is a major prerequisite for a probe to have useful selectivity for vicinal dichalcogenol reductases in cells. We challenged all probes with the most abundant cellular thiol, GSH. Even at a 300‐fold excess of reductant, **SeSC** remained fully inert (Figure 5c): a further improvement over the already marginal GSH activation of reference TrxR probe **RX1** (4% after 3 h), presumably due to **SeSC**’s preorganisation for retro‐addition (*k*_T‐1_, Figure 1a). **P‐SeSC** had marginal GSH reactivity (7%) but this is still orders of magnitude slower than reaction with DTT (dithiothreitol). In contrast, diastereocontrol **SeST** was readily activated by GSH, matching our hypothesis for trans‐bicycles (Figure 1b,III); and regiocontrol **SSeC** was activated even faster, coherent with our hypothesis for 1,2‐ versus 1,3‐Se,N regiocontrol (Figure 1b,IV: **SSeC** can undergo activation after a single monothiol addition, whereas SeS regioisomers require full reduction before release so must also overcome their tendency for GSH cycling Figure 1a).

Finally, we evaluated probe activation by DTT, a chemical mimic for vicinal dichalcogenol reductases (e.g., Trx, Grx, and TrxR) in the sense that its vicinal dithiol ensures the same molecularity during the reduction steps, as the cellular reductases do. This allows us to qualitatively assess the probes’ reactivity along the dichalcogenol reductive pathway of their intended targets. At low DTT concentrations where reduction is rate‐determining (differences of *k’*_cyc_ are neglected), probe candidate **P‐SeSC** had similar activation kinetics as monocyclic **RX1** or **A3** or regiocontrol **SSeC** (Figure 5d). **SeST** stands out for its >20× more efficient activation by even stoichiometric amounts of DTT (reaching its limit of apparent cyclisation rate *k'*_cyc_), which we ascribe to its quasi‐irreversible relaxation of chair‐boat strain to a monocyclic chair intermediate, which we expected would make it promiscuously reactive (discussed at Figure S6). The P group played a minor role in these DTT rates (**RX1** and **A3** had identical rates; **SeSC** had half the rate of **P‐SeSC**; these differences are too small to be interpreted strongly).

Taken together, both **SeSC** probes showed potential to be effective as TrxR probes, in that their post‐reduction cyclisation rate was much improved, while their monothiol resistance as well as dithiol activation rates were unimpaired or improved, as compared to the known monocyclic analogue **RX1** (Figure 5).

### Enzymatic Assays

**Figure 6:**
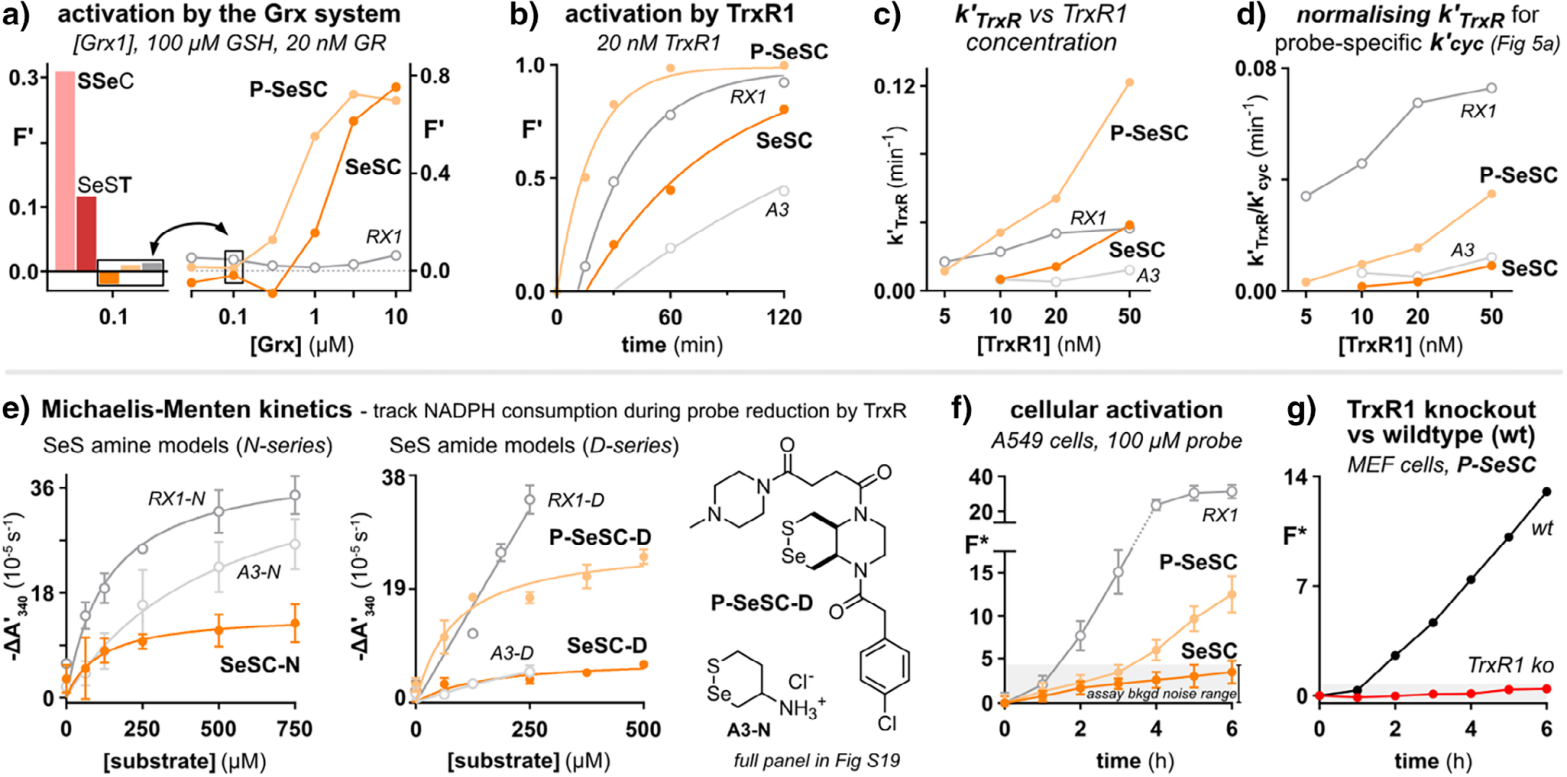
Biological evaluation: enzymatic and cellular assays. a) Qualitative probe comparison and dose‐response profiles to the GR/GSH/Grx cascade (200 µM NADPH, 20 nM GR, 100 µM GSH, 0.01–10 µM Grx1). F'(t) = [F_probe_(t)‐F_NADPH_(t)]/F_TCEP_(t); values for F'(t = 3 h) shown. b) TrxR activation time course (10 µM probes). c) Apparent rate constant for probe activation by TrxR, and d) roughly adjusted for differences in *k’*_cyc_. e) Michaelis‐Menten kinetics of reduction by TrxR, measured by NADPH consumption, for non‐releasing selenenyl sulfide model amines (N) and amides (D). ΔA’ is the initial rate of NADPH consumption (linear fit to ΔA data after correction for typically < 5% background drift; details at Figure S19). f), g) Representative fluorescence activation experiments in f) A549 cells, and g) MEF cells with or without genetic knockout of TrxR. F*(t) = F_probe_(t)‐F_DMSO_(t)‐F*(*t* = 0). See Figures S5 and S19 for all structures of model compounds, and Figures S17–S22 for results from all probes.

A good probe for TrxR also needs to resist probe activation by other reductases, particularly since physiological concentrations of TrxR are orders of magnitude lower than for redoxin‐type proteins (nM versus µM).^[^ ^32^ ^]^ As a counterexample, the control probes **SSeC** and **SeST** were effectively activated by the Grx/GSH/GR system (Figures 6a and S17). While it was never in doubt that those monothiol‐labile mechanistic controls would be too unselective to serve as TrxR probes, these assays highlight the additional constraints of reductase selectivity in that they were activated even when GSH was supplied at concentrations too low for probe activation in GSH‐only assays (Figure S14). **SSeC** was activated dose‐dependently in [Grx1], indicating the dithiol Grx as its effective reductant; while complex effects were seen for **SeST**, likely depending both on Grx1 and on GSH that here is catalytically re‐reduced by GR (see Figures S7, S14, and S17 for all enzymatic results with **SeST** and **SSeC**).

Pleasingly however, the **SeSC** probes were much less labile to the Grx/GSH system, becoming activated at ≥0.3–3 µM Grx. The monocyclic **RX1** was exceptionally inert (Figure 6a), but we caution against simplistically comparing bicyclic versus monocyclic probes' results in an enzyme‐only assay to conclude on their cellular suitability. One reason is that signal generation convolutes several effects that vary strongly between assay and probe types, such that enzyme‐only assays can be indicative, but not predictive. This is because signal generation requires reduction to the selenolthiol (rates unknown), then cyclisation before re‐oxidation by suitable substrates (e.g., disulfides in cells, for which preorganised **SeSC**‐derived selenolthiols are likely faster and stronger reductants than acyclic **RX1**‐derived selenolthiols^[^ ^13^, ^33^, ^34^ ^]^; or oxygen in enzyme‐only assays). We expect re‐oxidation rates to differ vastly between enzyme‐only assay conditions, and even more when compared to cell assays. Since **SeSC** cyclised 10× faster than **RX1** (Figure 5a,b), we expected bicyclic probe activation to be much less sensitive to re‐oxidation rate variations (cell‐free versus cellular) than **RX1** is. A second reason is that enzyme assays feature no alternative targets, so they can drive reductions that in cells would be outcompeted by more favourable substrates or hindered by compartmentalisation. Grxs indeed have stricter substrate preferences than TrxR;^[^ ^35^ ^]^ so we reasoned that if the **SeSC** probes had good TrxR activation kinetics, and if this TrxR reducibility were then better maintained in cells, **SeSC**s could still be improved‐performance probes for cellular TrxR activity.

Thus, we next evaluated the probes’ reducibility by TrxR. Though we expected that preorganisation for retro‐addition in the bicyclic **SeSC**s would make them more slowly reduced by TrxR than the monocyclics (**RX1**/**A3**) (Figure 1b,I), we hoped that their faster cyclisation (Figure 1b,II) would make their translation to other assay conditions less error‐prone, and ideally even make them rate‐competitive for signal generation in the cell‐free assay. Pleasingly, with a cellularly reasonable 20 nM of TrxR, **P‐SeSC** indeed gave signal ca. 2× as fast as **RX1**, and in the somewhat slower series without the “P” piperazinamide, **SeSC** was also ca. 2× as fast as **A3** (Figure 6b). The difference of activation rates between bicyclic and monocyclic probes also scales with TrxR concentration, with the bicyclic probes showing a stronger performance advantage when probe reduction is less rate‐limiting (Figure 6c): matching expectations about the retro‐addition effect. This was highlighted by roughly adjusting the activation rates for their large differences in apparent *k'*_cyc_ (fast for bicyclic **SeSP**s, slow for monocyclic analogues; Figure 5a); the adjusted rates indicate that, e.g., the monocyclic basic **RX1** was a much better substrate for TrxR reduction than its bicyclic analogues (Figures 6d and S18).

To study the initial TrxR reduction rate more cleanly, without such convoluted balances of probe re‐oxidation versus cyclisation, we turned next to Michaelis Menten kinetic studies. We created new sets of non‐fluorogenic selenenyl sulfides as TrxR substrates whose reduction step we could monitor indirectly, by tracking NADPH consumption (Figure S19). All the 1,2‐thiaselenanes were rather poor substrates for reduction by TrxR, in both the basic amine (**N**) and PQ‐mimicking amide (**D**) series.^[^ ^11^ ^]^ However, within each series, the two trends from the fluorogenic probe activation studies were confirmed: monocyclic SeS‐substrates are more rapidly reduced than **SeSP**s (*cf*. retro rates), and the P piperazinamide greatly accelerated reduction (Figures 6e and S19).

### Cellular Assays

To conclude, we tested **SeSP** probe performance in cellular assays, hoping to see TrxR selectivity with acceptable kinetics, although we anticipated that the bicyclic **SeSP**’s slower reduction by TrxR would hamper their activation once inside cells. Fluorescent signal from **P‐SeSC** was indeed low, at ca. 30% relative to monocyclic **RX1** (Figure 6f). Thus, **P‐SeSC**’s exceptionally fast cyclisation kinetics do not compensate for its lower rate of reduction by TrxR (and/or its likely lower rate of cellular entry: see TMU discussion in the Conclusion). Yet, TrxR suppression studies using partial chemical inhibition (Figure S20c, dose‐dependent signal reduction) supported the total genetic knockout assays (Figure 6g, ≫14‐fold signal knockout) to indicate that **SeSC**s retain strong cellular selectivity for TrxR. As plausibility controls, the reduction‐labile **SSeC** and **SeST** matched expectations by being cellularly activated to high levels of fluorescence, that were not much decreased upon TrxR knockout (Figures S20 and S22). Taken together, these data support the hypothesis of a small but TrxR‐selective signal profile for the **SeSC** probes.

## Conclusion

Selenenyl sulfides are key intermediates in redox biochemistry, but they have remained drastically understudied as a chemical class (only 15 unique cyclic scaffold types reported: fewer even than the number of human selenoproteins; Scheme S1). The **SeSC** scaffold is the first aliphatic bicyclic selenenyl sulfide. We provide a regioselective and diastereoselective synthesis of this system, on scale, by a short sequence involving three aziridines as convenient control elements for 1,2‐functionalisations. **SeSC** was designed to merge key hypotheses from several rounds of redox probe development, into a densely functionalised core with i) a cyclic selenenyl sulfide that is ii) piperazine‐annellated in a iii) *cis* fashion, and with an iv) Se that is proximal to the cargo‐releasing carbamate. When combined, these features yield **SeSC**‐based redox probes that strongly resist off‐target activation by monothiols (Figure 5c) yet rapidly release a cargo upon reduction (Figure 5a), and efficiently respond to the mammalian selenoenzyme TrxR (Figure 6b): indicated as their major reductant in live cells (Figure 6g).

Hypothesis controls in which a single parameter of this design was purposely altered, plus recent mechanistic work on disulfides,^[^ ^9^, ^13^ ^]^ suggest general design needs for high‐quality dichalcogenide probes: 1) A stepwise kinetic picture of dichalcogenide redox is required to understand their biochemical reactivity profiles; a crude overall “reduction potential” is not enough. For example: the 1,2‐thiaselenane motif in all **SeSP** probes ensures nucleophilic addition exclusively at Se,^[^ ^13^, ^15^, ^16^ ^]^ but its regiochemistry can be chosen to rationally create probes that are highly TrxR‐selective, or else are monothiol‐labile (Figure S14, e.g., **SSeC** versus **SeSC**). 2) Effective reaction molecularities are particularly important to understand the steps in dichalcogenide exchange sequences. For example: thiol addition onto *trans*‐fused dichalcogenides is almost irreversible (very low *k*_T‐1_), which makes **SeST** reduction by monothiols a quasi‐bimolecular process, with its rate approaching that for activating regiocontrol **SSeC** that can undergo true bimolecular activation. Yet, when using vicinal dithiol reductants, the second step in reduction is unimolecular and thiol cycling at Se is no longer rate‐limiting (due to vicinal dithiol preorganisation): so **SeST** is unprecedently dithiol‐labile, while **SSeC** is no more labile than the TrxR probe **SeSC**.

We perceive that chemical designs which control the preorganisation of artificial dichalcogenides for various steps, from oxidative closure (retro‐addition) through to irreversible cargo expulsion (carbamate cyclisation), can be a crucial means to model^[^ ^36^, ^37^ ^]^ and to probe^[^ ^13^, ^16^ ^]^ biochemical redox in action; and we believe that a surprisingly high degree of selectivity may yet be reached if step kinetics can be rationally and orthogonally tuned. For example, here, we find that dichalcogenide annellation with mono‐ or bis‐*N*‐acylated piperazines delivers exceptionally high *k*_cyc_ rates (compare monocyclic **RX1** versus annellated **SeSC**). While a lowered entropy of activation surely contributes to this rate difference (Figure 1b,II), we may also suspect that the *cis/trans* ratio of the tertiary carbamate (which depends on backbone architecture, at least for related amides^[^ ^38^ ^]^), and the conformational flexibility of the annellating ring (that could be tuned by substitutions in the piperazine 5‐ and 6‐positions), would both play key roles in the carbamate cyclisation step yet would not impact the steps in dithiol or monothiol reductions. By contrast, the distal *N*‐methyl piperazine amide (“P group”) strongly improved the reduction kinetics of **P‐SeSP** as compared to the unsubstituted **SeSP** (which also features a basic amine, so the reactivity differences cannot stem from local basicity alone). We suspect that the P piperazineamide may accelerate forwards dichalcogenide exchanges through protonation of the leaving group chalcogen, as well as by stabilising the addition intermediate to hinder retro‐additions (*k*_R_ **_‐_**_1_ or *k*_T_ **_‐_**_1_),^[^ ^36^ ^]^ which increase processing rates but incur minor monothiol lability.

This work has essentially doubled the range of reagents that can usefully interface with TrxR in live cells. The **SeSP**‐based bicyclic probes feature much‐improved cyclisation step kinetics compared to prior art monocyclic **RX1**, but their cellular signal levels were still rather lower. **SeSP** substrate derivatives that are more reactive for the initial addition step, or linearly signal‐generating fluorogenic cargos offering reduced lag times,^[^ ^43^ ^]^ both seem viable design solutions to create brighter **SeSP**‐based cellular probes in future work. Conceptually, the signal differences between **RX1** and **SeSP** seem to suggest that TrxR reduction kinetics were a key factor limiting cellular processing of **SeSC**s relative to that of **RX1** (*c.f*. the similarities between cellular activation kinetics and cell‐free reduction‐only kinetics, Figure 6d–f). However, we see this as only one piece of the thiol redox selectivity puzzle, and we caution against early interpretations based on too few chemical scaffolds as inputs.

For example, thiol‐mediated uptake (TMU) almost certainly amplifies the overall signal from **RX1** by increasing cellular delivery, thanks to rapid and reversible exchanges of exofacial thiols at the probe's selenium centre;^[^ ^39^ ^]^ but this effect almost certainly depends on specific exofacial and transmembrane thiol species, whose expression and redox poise constitute additional variables between samples and assay types.^[^ ^40^ ^]^ It is tempting to speculate that TMU may also increase the cellular **SeSP** readout compared to that for probes that enter cells purely by passive diffusion, but that much less TMU operates on bicyclic **SeSPs** due to their much faster rate of retro‐addition compared to monocyclic **RX1**. That might in turn favour **SeSPs** being high‐quality probes, by keeping their readouts more cleanly dependent on the intracellular redox activity we aimed to study: or alternatively, since it is quite possible that TrxR/Trx‐system redox activity couples into the TMU networks which are hijacked by selenenyl sulfide probes, foregoing TMU‐moderation of their readout might simply be a drawback for probe sensitivity. These will not be easy topics to study: cellular delivery of reversibly thiol‐engaging motifs is highly dependent on reactivity parameters that are only just beginning to be elucidated despite a fairly large body of work;^[^ ^41^ ^]^ whereas there is essentially zero information known about the interplay of TMU with intracellular thiol redox circuits.

Zooming out from this example, TMU is just one of several^[^ ^42^ ^]^ molecularly‐defined challenges or opportunities that will affect the design and performance of small molecule redox substrates. Designing their structures so that the individual reaction step rates along their intracellular reduction pathways deliver defined cellular redox selectivity profiles, despite the many, variable, and still largely unknown influences of the heterogeneous cellular setting, faces many difficulties. Exploring this barely known landscape will need much discovery and validation of mechanistic hypotheses before satisfying rationales can be established. New scaffold designs and stringent controls that test the dichalcogenide redox chemistry at work, will be the guides for progress in this domain.

## Supporting Information

Synthesis, analysis, biochemistry, and cell biology, including Figures S1–S22, Tables S1–S3, and Scheme S1 (PDF). The authors have cited additional references within the Supporting Information.^[^ ^44^, ^45^, ^46^, ^47^, ^48^, ^49^, ^50^, ^51^, ^52^, ^53^, ^54^, ^55^, ^56^, ^57^, ^58^, ^59^, ^60^, ^61^, ^62^, ^63^, ^64^, ^65^, ^66^, ^67^, ^68^, ^69^, ^70^, ^71^, ^72^, ^73^ ^]^

## Author Contributions

L.Z. performed synthesis, analysis, and chemoreductant assays; designed the study and experiments; and coordinated data assembly. L.D.W. performed enzyme and cellular assays. K.C.S. performed enzyme assays and Michaelis Menten studies, as supervised by E.S.J.A. and A.M. performed computational studies, as supervised by L.O. Together, L.Z. and O.T.‐S. designed the targets, supervised the study, interpreted the data and wrote the manuscript with input from all authors.

## Conflict of Interests

The authors declare no conflict of interests.

## Supporting information



Supporting Information

Supporting Information

## Data Availability

The data that support the findings of this study are available in the Supporting Information of this article.
