# Supplementary material for: Bicyclic Selenenyl Sulfides with Tuned Bioreductive Step Rates Reveal Constraints for Probes Targeting Thioredoxin Reductase
Source: Angew Chem Int Ed Engl. 2025 Jul 16;64(35):e202508911. doi: 10.1002/anie.202508911 (PMC12377444; doi:10.1002/anie.202508911)
Supplement: Supplementary file 1 — Supporting Information [file ANIE-64-e202508911-s002.pdf]

Supporting Information to:

Bicyclic Selenenyl Sulfides with Tuned Bioreductive Step Rates Reveal Constraints for Probes Targeting Thioredoxin Reductase

Lukas Zeisel[1,2]*, Lucas Dessen-Weissenhorn[2], Karoline C. Scholzen[3], Andrea Madabeni[4], Laura Orian[4], Elias S. J. Arnér[3,5], Oliver Thorn-Seshold[1]*

[1] Faculty of Chemistry and Food Chemistry, TU Dresden, Bergstrasse 66, 01069 Dresden, Germany.
[2] Department of Pharmacy, Ludwig Maximilians University Munich, Butenandtstr. 5-13, 81377 Munich, Germany.
[3] Division of Biochemistry, Department of Medical Biochemistry and Biophysics, Karolinska Institutet, Solnavägen 9, 171 65 Solna, Sweden.
[4] Department of Chemical Sciences, University of Padova, Via Marzolo 1, 35129 Padova, Italy.
[5] Department of Selenoprotein Research, National Institute of Oncology, 1122 Budapest, Hungary.
* Corresponding authors: lukas.zeisel@uibk.ac.at, oliver.thorn-seshold@tu-dresden.de

Table of Contents

1 Supplementary tables, notes and discussions S2

Scheme S1: Previously known cyclic selenenyl sulfide substructure types S2
Figure S1: Kinetics considerations for known dichalcogenide probes (extended Figure 1) S2
Table S1: Optimisation of bis-aziridine formation S3
Table S2: Test reactions on S/Se-nucleophilic aziridine ring opening S3
Table S3: *N*-Busyl aziridine opening S4
Figure S2: Probing the *S*-electrophilicity of an aziridine-SeCN intermediate S4
Figure S3: Synthesis of *trans*-SeSP S5
Figure S4: Monitoring aziridine formation/opening by NMR S5
Figure S5: Synthesis of fluorogenic thiaselenane probes S6
Notes on control assays S6
Figure S6: Discussion of SeST's exceptional reactivity with DTT S7
Figure S7: A Grx-mediated (de-)glutathionylation pathway in probe reduction? S7

2 Computational studies (Figures S8-S10) S8

2.1 Methods S8
2.2 Mechanistic analysis of monothiol reduction S9
2.3 Thiol-addition transition states S10
2.4 Intermediates of thiol addition S10

3 General methods for probe evaluation S11

3.1 Cell-free assays S11
3.2 Cellular assays S12

4 Chemical probe activation assays (Figures S11-S16) S13

5 Enzymatic assays (Figures S17-S19) S18

6 Cellular assays (Figures S20-S22) S23

7 Organic synthesis S24

8 Analytics S42

8.1 Atomic coordinates, imaginary frequencies and energies of optimised structures S42
8.2 X-Ray crystallography S50
8.3 NMR spectra S52

9 Supporting references S81

1 Supplementary tables, notes and discussions

Scheme S1: Previously known cyclic selenenyl sulfide substructure types

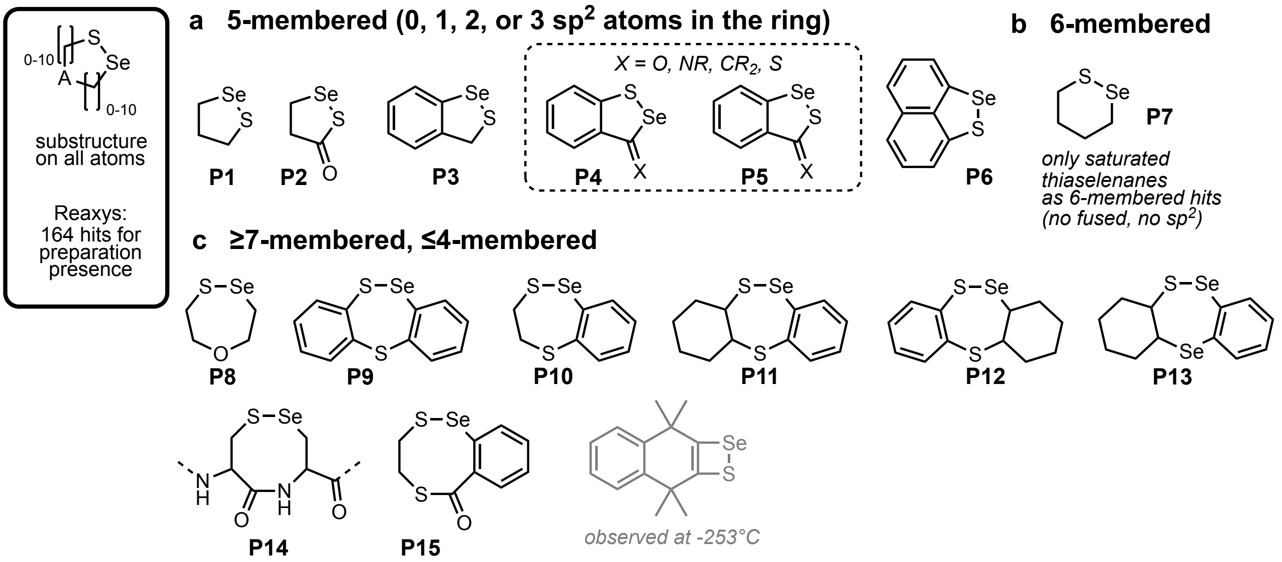

Scheme S1: overview of previously reported cyclic selenenyl sulfide chemotypes

Figure S1: Kinetics considerations for known dichalcogenide probes (extended Figure 1)

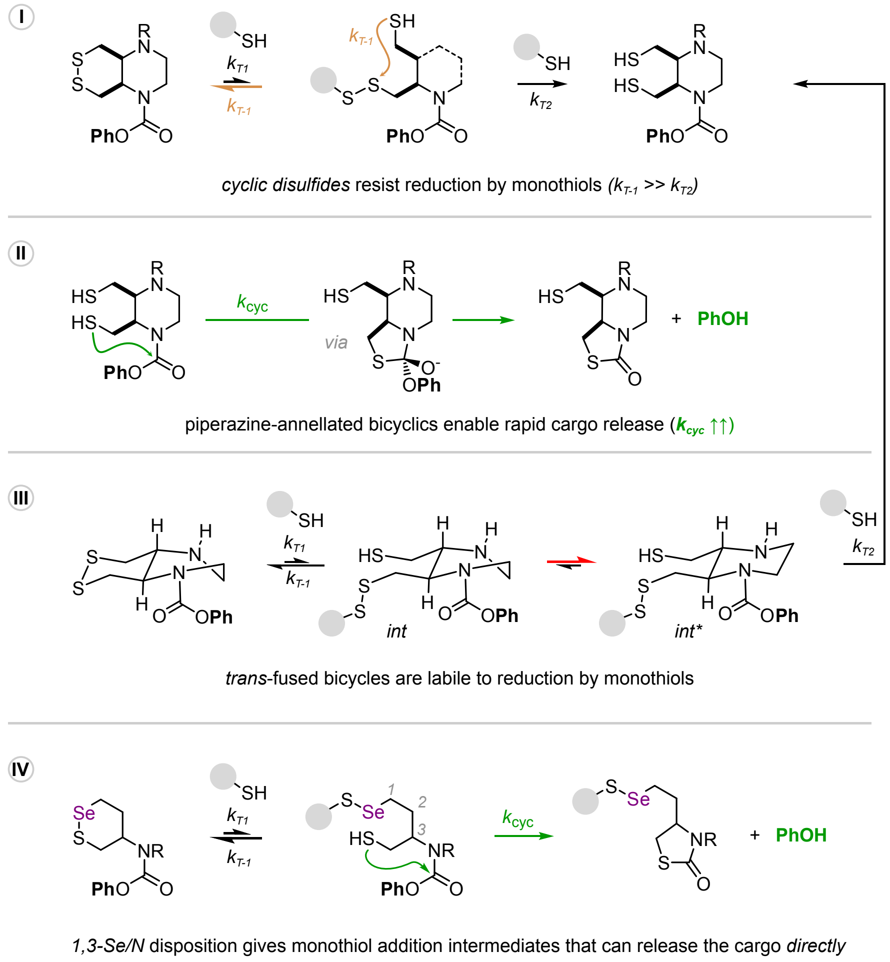

Figure S1 Extended Figure 1 with expanded detail on mechanism and structure.

Table S1: Optimisation of bis-aziridine formation

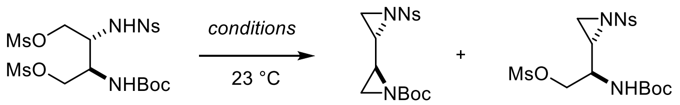

S1 **A (S10)** **B**

#	reagent	solvent	time	LCMS	isol. yield (A)
1	KO*t*Bu (1 M in THF, 2.5 equiv.)	THF	30 min	90% **A**	8%
2	solid KO*t*Bu (4.0 equiv.)	THF	1 h	80% **A**	-
3	NaH (5.0 equiv.)	THF	1 h	80% **A**	-
4	Cs_2CO_3 (1.3 equiv.)	THF	24 h	50% **B**	-
5	LiOH (2.5 equiv.)	THF/H_2O	30 min	100% **B**	-
6	LiHMDS (1 M in toluene, 2.5 equiv.)	THF	2 h	complex	-
7	NaH (2.5 + 2.5 equiv.)	THF	90 min	95% **A**	29%[a]
8	NaH (2.5 + 2.5 equiv.)	THF	90 min	95% **A**	76%[b]

[a]After chromatography on silica. [b]After aqueous workup. Purity was comparable to that after chromatography. In the presence of the Boc-protective group, the dimesylate intermediate decomposes readily with prolonged reaction times (see in the experimental below for synthetic details). Upon isolation, **S1** should be used directly for follow-up transformations.

Table S2: Test reactions on S/Se-nucleophilic aziridine ring opening

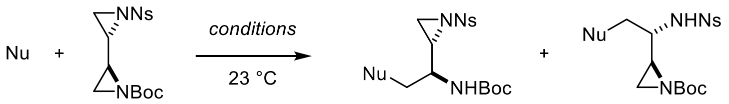

S10 **X** **Y**

#	Nu	solvent	time	NMR
1	HSAc (4.0 equiv.)	CH_2Cl_2	5 h	30% **Y**
2	KSAc (2.0 equiv.)	DMF	1 h	80% **Y**. Chemical shifts match species from *1*
3	KSeCN (1.9 equiv.)	DMF	1 h	**Y** forms, but NMR reveals a complex reaction mixture.
4	KSeCN (1.3 equiv.)	THF	1 h	Complex reaction mixture with traces of **Y**.
5	KSeCN (2.5 equiv.)	MeOH	1 h[a]	Full conversion of SM, 100% **Y**[a]

[a]Directly submitting the reaction to NMR upon mixing all reagents (experiment run in CD_3OD) revealed that full conversion was already achieved within minutes.

Table S3: *N*-Busyl aziridine opening

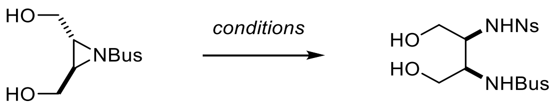

#	reagent	solvent	temp.	time	NMR yield	isol. yield
1	NsNClNa (1.5 equiv.)	MeCN	60 °C	32 h	20%	8%
2	$NsNH_2$ (1.1 equiv.), DIPEA (2.0 equiv.)	MeCN	23 °C	16 h	0%	-
3	$NsNH_2$ (2.5 equiv.)	DMF	80 °C	16 h	0%	-
4	$NsNH_2$ (2.5 equiv.), K_2CO_3 (5.0 equiv.)	DMF	23 °C	16 h	0%	-
5	$NsNH_2$ (2.5 equiv.), K_2CO_3 (5.0 equiv.)	DMF	80 °C	16 h	60%	52%
6	NsNClNa (2.3 equiv.)	MeCN	70 °C	32 h	20%	-
7	$NsNH_2$ (1.1 equiv.), NaOCl (1.1 equiv.)	MeCN	70 °C	32 h	>90%	69%

Figure S2: Probing the *S*-electrophilicity of an aziridine-SeCN intermediate

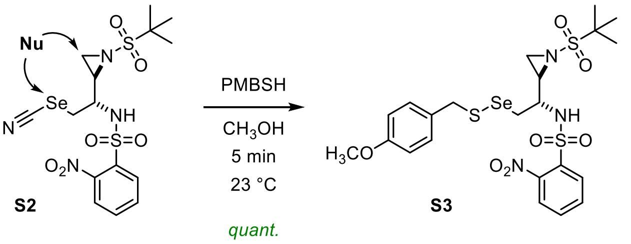

Figure S2 Test reaction to probe whether the tandem thiaselenane formation with bis-nucleophile Na_2S occurred via S_N2 first at Se or the aziridine-carbon. **S2** (12 mg, 24 µmol, 1.0 equiv.) was dissolved in anhydrous THF (0.05 M, 0.5 mL), and PMBSH (3.4 µL, 24 µL, 1.0 equiv.) was added in one batch. The reaction was stirred at 23 °C for 5 min and concentrated under a stream of N_2. NMR analysis revealed full conversion of the selenocyanate (via ^{13}C) and quantitative formation of a single species, whose Busyl-aziridine protons essentially matched the starting material. ESI-LCMS confirmed the target mass (M-H: 621.7). These results indicate that thiaselenane formation occurs via S_N2 of Na_2S first at R*Se*CN, then intramolecular cyclisation of the per-selenenyl sulfide onto the aziridine. However, it might be argued that the soft PMBSH nucleophile is not the ideal mono-nucleophile to model the reactivity of Na_2S.

Figure S3: Synthesis of *trans*-SeSP

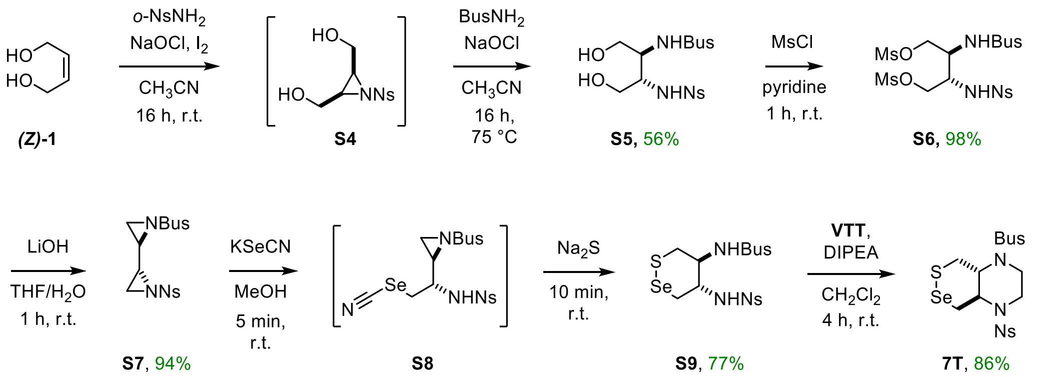

Figure S3 7-step synthesis of *trans*-piperazine-fused, orthogonally protected 1,2-thiaselenane.

Figure S4: Monitoring aziridine formation/opening by NMR

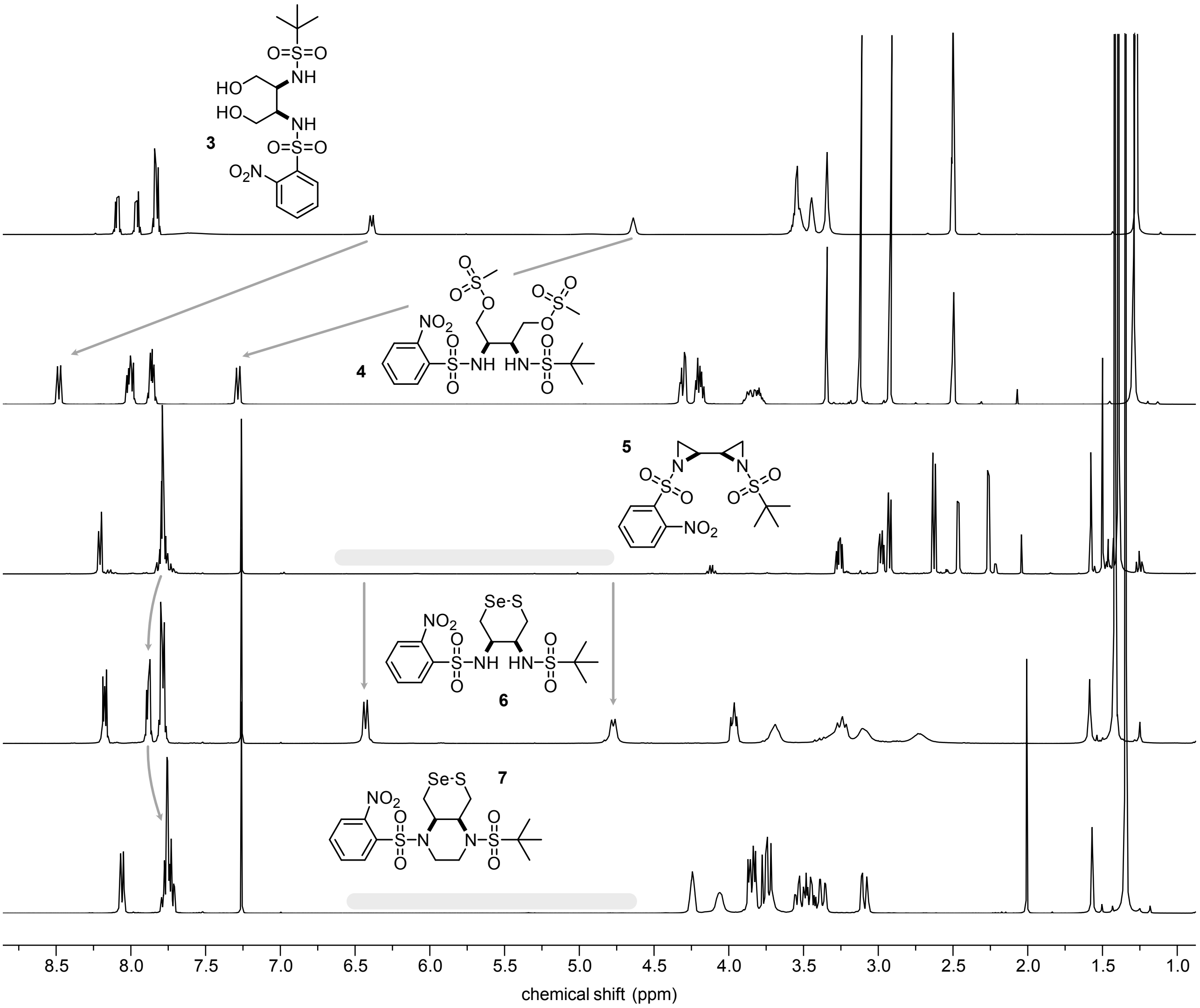

Figure S4 The synthetic sequence towards **SeSP**s is best monitored via NMR, since TLC analysis is often hindered by poor solubility (**3**, **4**) or marginal differences in Rf (**6**→**7**). The sulfonamide N-Hs are very useful diagnostic protons, which undergo significant changes in their chemical shift *en route* from **3** to **7**, or disappear completely (**5**, **7**). Note that spectra for **3** and **4** were measured in DMSO-d$_6$, NMR analysis of **5**-**7** was conducted in $CDCl_3$. For clarity, post-purification spectra are shown.

Figure S5: Synthesis of fluorogenic thiaselenane probes

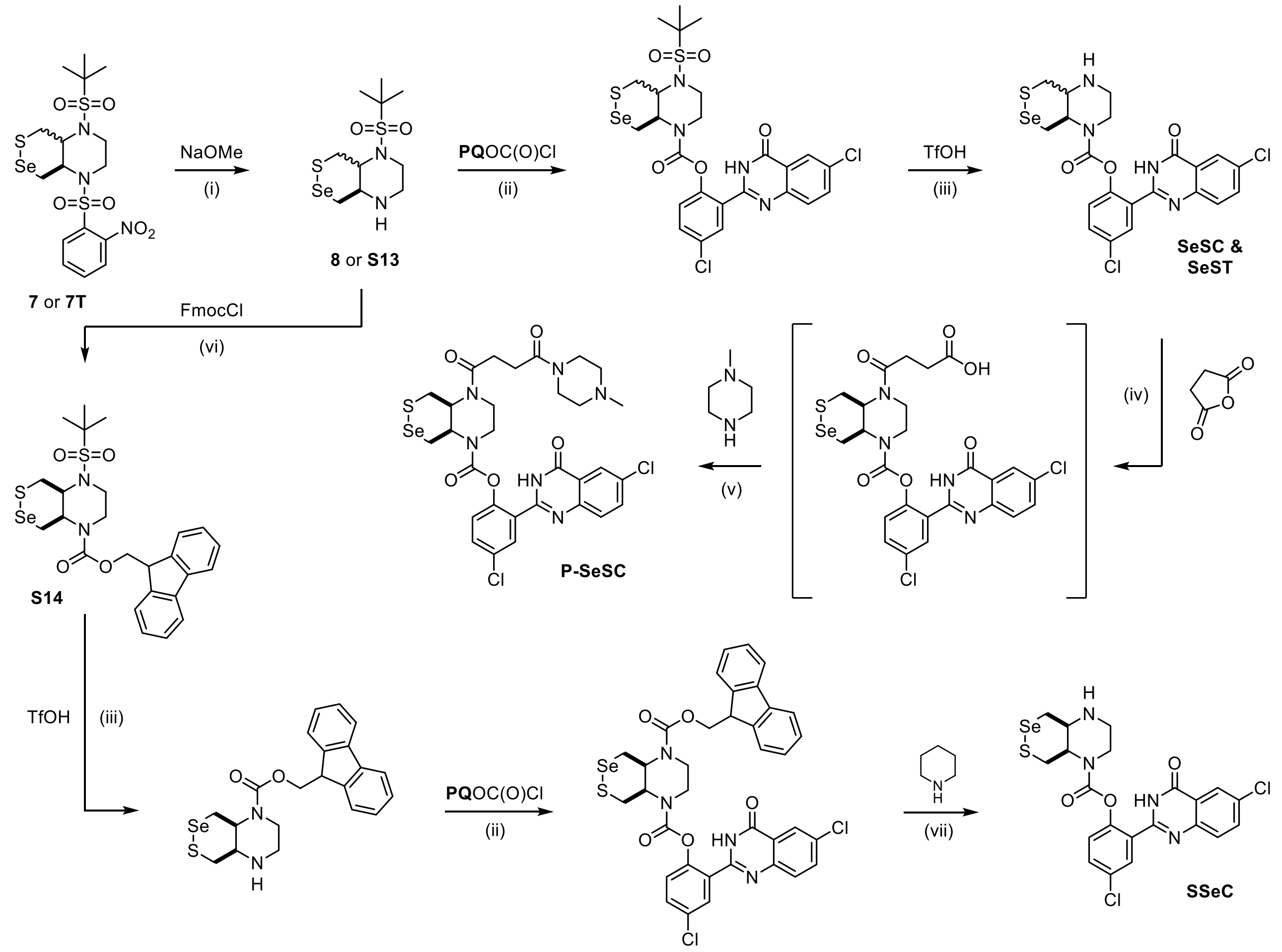

Figure S5 Syntheses of all novel thiaselenane probes featured in this study. (i) NaOMe (5.4 M in MeOH), THF, 0 → 23 °C; (ii) PQOC(O)Cl (60 wt%), DMAP, CH_2Cl_2, 23 °C; (iii) TfOH, *o*-anisole, CH_2Cl_2, 23 °C; (iv) succinic anhydride, DMAP, Et_3N, DMF, 80 °C; (v) TSTU, Et_3N, *N*-methyl piperazine, DMF, 23 °C; (vi) FmocCl, pyridine, CH_2Cl_2, 0 → 23 °C; (vii) piperidine, DMF, 23 °C.

Notes on control assays

Predict: no significant cellular non-reductive cleavage of the probes' tertiary carbamate: Some phenolic carbamates have previously been reported as hydrolytically unstable,[1] however, this instability is unique to NH-type carbamates that can undergo a spontaneous E1cB elimination pathway that is vastly quicker than the hydroxide addition-elimination pathway which *N*-disubstituted phenolic carbamates require for hydrolysis.[2] To avoid this problem, we designed reducible 1,2-thiaselenane probes (in **RX1** and **SeSP**s) using tertiary carbamate linkages. To still control for potential non-catalysed or esterase-mediated hydrolysis in aqueous buffer or within cells, we synthesised non-reducible tertiary carbamate probe **C1** (see ref[3]) and stringently used it as control in all cell-free and cellular assays that profiled **RX1**'s performance.[3] In all cases, **C1** was completely hydrolytically inert, generating no fluorescent signal, providing proof that non-reductive activation of these probes can be neglected.

Predict: no significant cellular activation by H_2S: hydrogen sulfide is a cellularly-present species that can engage in two sequential thiol/disulfide exchanges and thus potentially activate 1,2-dichalcogenide-based probes. However, we note that although it is considered "abundant" for a signalling molecule (in some cases up to 100 µM),[4] these concentrations are far below the monothiol concentrations needed to activate **SeSC**-type probes by two sequential reduction steps (see e.g. Fig S14). Alternatively, with these 1,2-thiaselenanes, inital S_N2 at Se would generate a perselenenyl sulfide ($RSeS^-$) which could, in theory, cyclise onto the carbamate to release the phenolic fluorophore. However, based on mechanistic analyses performed in our earlier study on **RX1** (Figs S33-S37 in that supporting information),[3] we consider such *6-exo-trig* cyclisation to be slow enough to be vastly outcompeted by k_{T-1} (here, Figure S1). Hence, activation of **SeSC**-type probes by H_2S inside cells should be negligible.

Predict: no significant activation by other selenoenzymes: the human proteome contains only 25 selenoproteins, and nearly all of the ubiquitously expressed selenoproteins possess monoselenol active sites, rather than selenolthiols. The most abundant of these are the monoselenol glutathione peroxidases, GPx1 and GPx4.[5] In our earlier study, we challenged

RX1 with GPx1 at concentrations up to 1 mM, which is well above physiological levels, but observed no probe activation over a 6-hour period.[3] This result supports the general requirement of a vicinal selenenolthiol reductase such as TrxR to reduce and activate 1,2-thiaselenane probes such as **RX1** and **SeSP**-based probes reported herein. Considering the exceptional inertness of **RX1** to being activated by GPx1, we omitted this control assay for **SeSP**s.

Figure S6: Discussion of SeST's exceptional reactivity with DTT

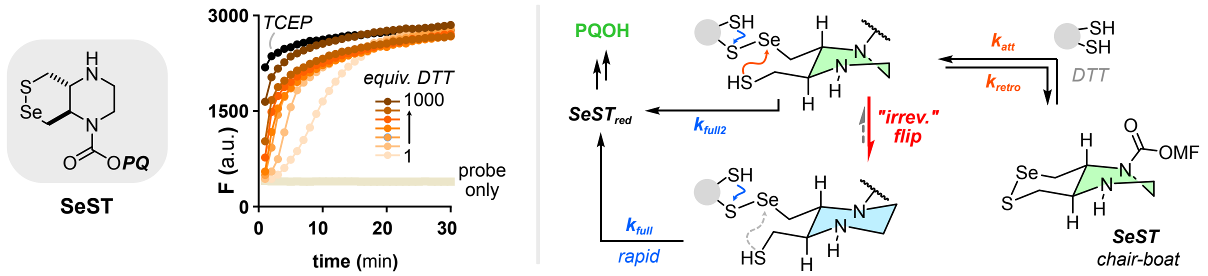

Figure S6 – see Figure S15 for full DTT data. In contrast to other **SeSP**-based probes, **SeST** exhibits exceptional activation kinetics even with equimolar amounts of DTT (this prevented deconvolution of k'_{red}(DTT) from k'_{cyc} in Figure S16). We suggest that initial nucleophilic attack at **SeST** produces an intermediate that relaxes to a lower energy conformer which can't undergo back-cyclisation to the unreacted probe (k_{retro}). Instead, the intermediate is locked and "awaits" full intramolecular reduction by DTT's vicinal thiol. More generally, **SeST**'s chair-boat conformation minimises k_{retro} through the conformational flip and thus is readily reduced by any thiol (see GSH data in Figure S14). Activation rate differences between monothiol GSH and dithiol DTT are due to the bimolecularity for reduction by DTT, i.e., the high effective molarity of DTT's resolving thiol. In addition, the DTT mechanism not only escapes retro-cyclisation through the piperazine conformational change but can already outcompete it from the boat-conformer of the addition intermediate.

Figure S7: A Grx-mediated (de-)glutathionylation pathway in probe reduction?

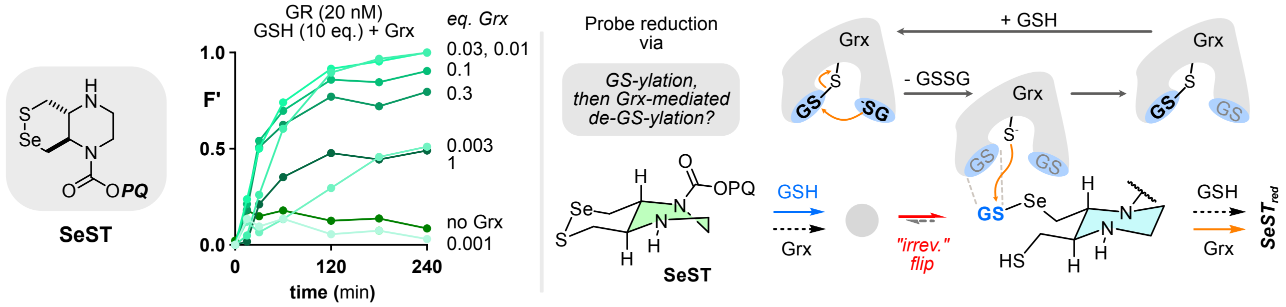

Figure S7 – datasets for Grx1 only (10 µM) and GR only (20 nM) were excluded for clarity, see Figure S17 for full data and a comprehensive assay description. During enzyme specificity screening, we again noted a peculiarity for **SeST**: probe activation was surprisingly efficient down to 0.003 equiv. Grx. Since GSH itself is not capable of activating **SeST** at 100 µM (~0% activation after 3 h, see Figure S14), even catalytically maintained GSH activity (via GR) should not result in efficient reduction. Instead, the actual mechanism at play could involve non-enzymatic GS-ylation of **SeST**, facilitated by high addition rates at Se and low k_{retro} once the chair-intermediate is reached. Subsequent *de*-GS-ylation by Grx produces the fully reduced probe. Since Grxs have evolved to function as cellular de-GS-ylases, this process should be highly efficient in terms of rates and regioselectivity (S vs Se). At very low Grx concentrations (0.001 equiv.), the second step naturally is less efficient (we currently lack an explanation for the self-inhibition observed at high Grx concentrations after similar initial rates). This working hypothesis does not strive to explain the actual biochemical processes in the Grx-system assay, but introduces a previously neglected pathway in dichalcogenide probe reduction – after all, Grxs' disulfide reductase activity does not exclusively relies on a Trx-like dithiol mechanism but, in tandem with GSH, can also follow one of three monothiol-mechanisms.[6]

2 Computational studies (Figures S8-S10)

2.1 Methods

All DFT calculations were performed with the 2019.307 release of the Amsterdam Density Functional (ADF) program. For all geometry optimisations, the OLYP density functional was used,[7,8] combined with a TZ2P basis set and a small frozen core approximation. Scalar relativistic effects were accounted for via the zeroth-order regular approximation (ZORA), as implemented in ADF.[8] This combination of density functional and basis set was previously benchmarked for structural and energetic properties of dichalcogenides,[9] and it has been commonly applied to the theoretical exploration of their reactivity.[10,11] Additionally, OLYP is recognized as one of the best density functional approximations to tackle S_N2 reactions, such as those explored in this work. All transition states in which a proton must be transferred from the reactant (nucleophile) to the product (leaving group), were modelled via a solvent-assisted proton-exchange fashion (SAPE), using two water molecules to mediate the proton transfer (c.f. loopy arrows in Figure 3).[12,13] To aid in the stabilisation of the hydrogen bond network, Grimme D3 correction,[14,15] with the Becke-Johnson damping factor,[16,17] was applied. All geometries were thus optimized at the ZORA-OLYP-D3(BJ)/TZ2P level of theory. Solvation effects were accounted via single point calculations with the COSMO solvation model, as implemented in ADF.[18,19] Water was chosen as the implicit solvent, with the default ADF settings for the dielectric constant, atomic radii, and scaling function. Thus, final energetics were computed at the COSMO-ZORA-OLYP-D3(BJ)/TZ2P // ZORA-OLYP-D3(BJ)/TZ2P level of theory. Thermodynamics corrections were computed from harmonic frequencies via standard statistical thermodynamics relathionships based on the perfect gas approximation at 1 atm and 298.15 K. All energies were corrected for the standard state change which occurs when moving from gas phase (1 atm) to condensed phase (1 M).[20] Conformational sampling of all minima was performed via the CREST routine coupled to the GFN2-xTB method of Grimme and coworkers, to obtain the minimum energy structures.[21,22]

As noted during review, the SAPE setup we used does not attempt to account for protonation state effects (e.g. that (a) deprotonated thiolate / selenolate are ca. 100× more nucleophilic than neutral thiol/selenol; (b) we consider it most plausible that the physiological protonation state of the initially attacking chalcogenols (TS^{A-B}) are anion states (e.g. TrxR selenolate, Trx thiolate, etc), although it is unclear in what contexts the resolving thiols (TS^{B-C}) are deprotonated or not). However, since the SAPE setup enforces enforces proton transfer to occur, and since we wished to keep the reaction mechanisms consistent (for comparing rather small energy differences), we chose to stay with a strictly comparable protonation setup that always uses protonated nucleophiles (and thus protonated leaving groups). Rather than delivering results that are specific to certain bulk pH values (which anyway are not necessarily good predictors for in-enzyme protonation states), we believe this approach gives answers that are indicative of the intrinsic reactivity differences of the @S/@Se processes.

2.2 Mechanistic analysis of monothiol reduction

a Thiaselenane reduction with monothiols

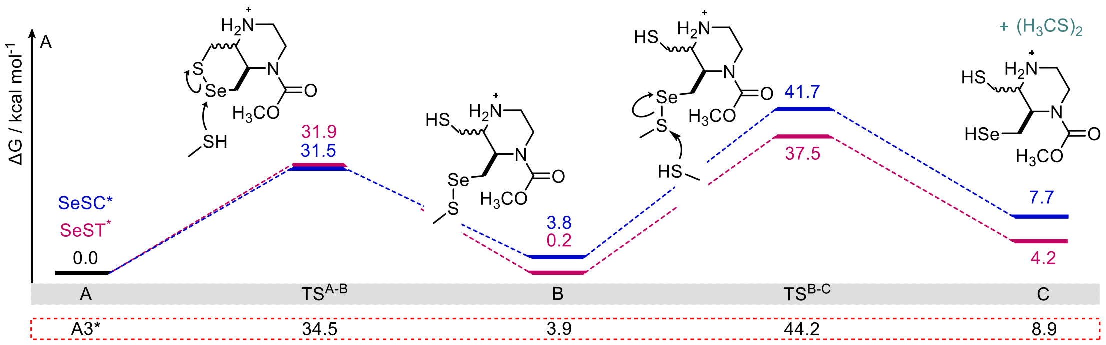

b

	SeST*	SeSC*	A3*
$\Delta G^{\ddagger}$ ($TS^{B\to A}$)	31.7	27.7	30.6
$\Delta G^{\ddagger}$ ($TS^{B\to C}$)	37.3	37.9	40.3
$\Delta\Delta G^{\ddagger}$ ($TS^{B\to C}$ - $TS^{B\to A}$)	5.6	10.2	9.7

c

nucleophile:	SeH - SH	H-SeST*	H-SeSC*	A3*
CH_3SeH vs CH_3SH	$\Delta\Delta G^{\ddagger}$ ($TS^{A\to B}$)	-4.7	-4.8	-3.4
	$\Delta\Delta G_r$ (B)	-5.6	-5.7	-3.8

d Reaction energies for on-carbamate cyclisation

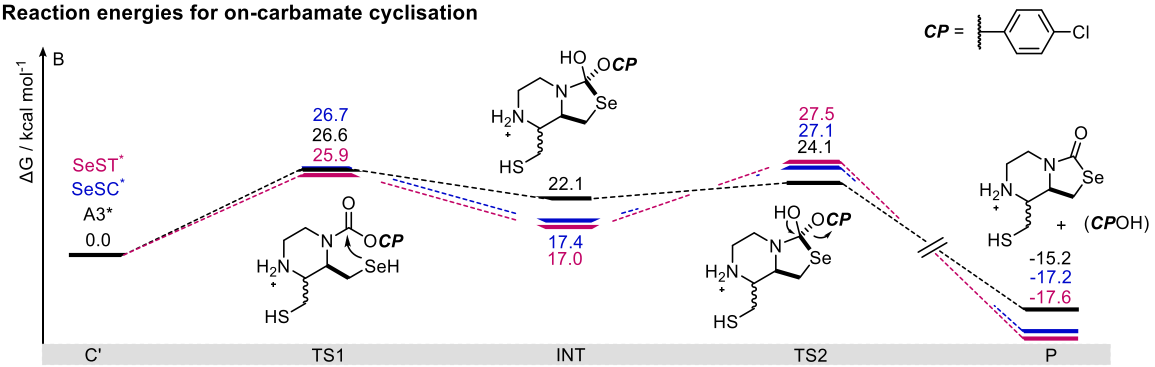

Figure S8 Calculated activation and reaction energies (kcal mol^{-1}) for: (**a**) Monothiol(-selenol) reduction of **SeSC*** and **SeST***, modelled with H_3C-SH or H_3C-SeH as nucleophiles. (**b**) Key values that predict the lifetime of thiol addition intermediates ($\Delta G^{\ddagger}$ ($TS^{B\to C}$)) and a probe's kinetic propensity for full reduction ($\Delta\Delta G^{\ddagger}$). (**c**) The influence of substituting S with Se in the nucleophile of thiol addition. (**d**) Comparison of on-carbamate cyclisation energies for **SeSC***, **SeST*** and **A3***. The asterisk indicates that calculations were conducted for model probes, in which the basic amine is protonated, and which feature a simplified *O*-methyl carbamate instead of *O*-PQ. For **A3***, data from an earlier study was used.[23]

2.3 Thiol-addition transition states

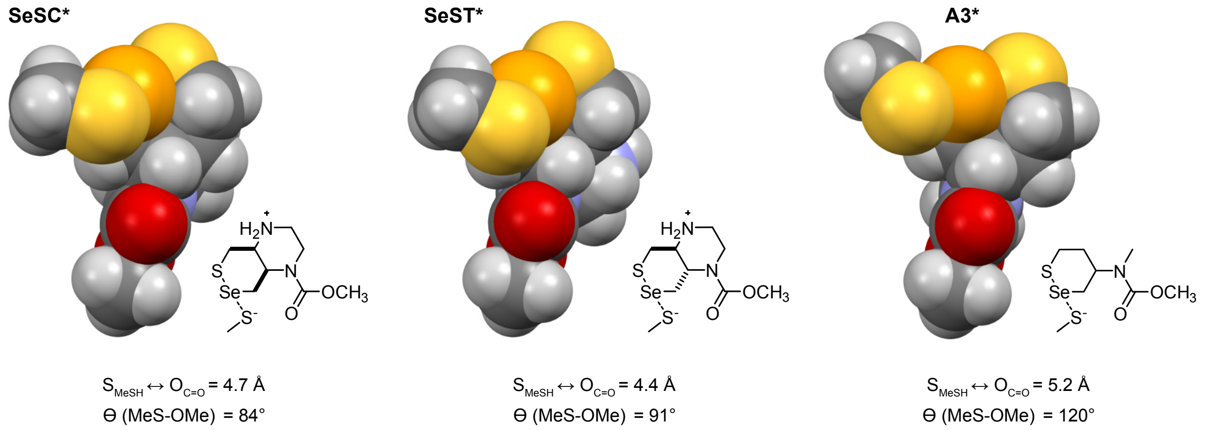

Figure S9 Calculated conformations for the transition states of thiol-addition. SeSC*, SeST* and **A3*** are shown in comparison. Water molecules that mediate proton transfer are omitted, and only selected hydrogen atoms are shown. The distance between the nucleophilic thiolate and the carbamate carbonyl is shown as qualtitative parameter that could sterically (or through elecotrostatic interactions) confine the addition reaction. Along the same lines, the dihedral angle between the nucleophile's entry vector and the carbamate ester's exit vector are shown. Though seemingly not of importance for the activation kinetics of methyl carbamates ($\Delta G^{\ddagger}_{A\text{-}B}$: **A3*** > **SeSC***, **SeST***), lower dihedral angles for **SeSPs** vs **A3*** could play a large role for sterically more demanding reductants such as redoxins and TrxR, and for the much larger PQ-based carbamates. In all cases, The C-Se-S^{Nu} bond angles of the transition state match the ideal transition state geometry (90°) for chalcogenol exchange reactions,[24,25] are already realised in the ground state selenenyl sulfides (93, 95, 95°, respectively) and indicate high reactivity.

2.4 Intermediates of thiol addition

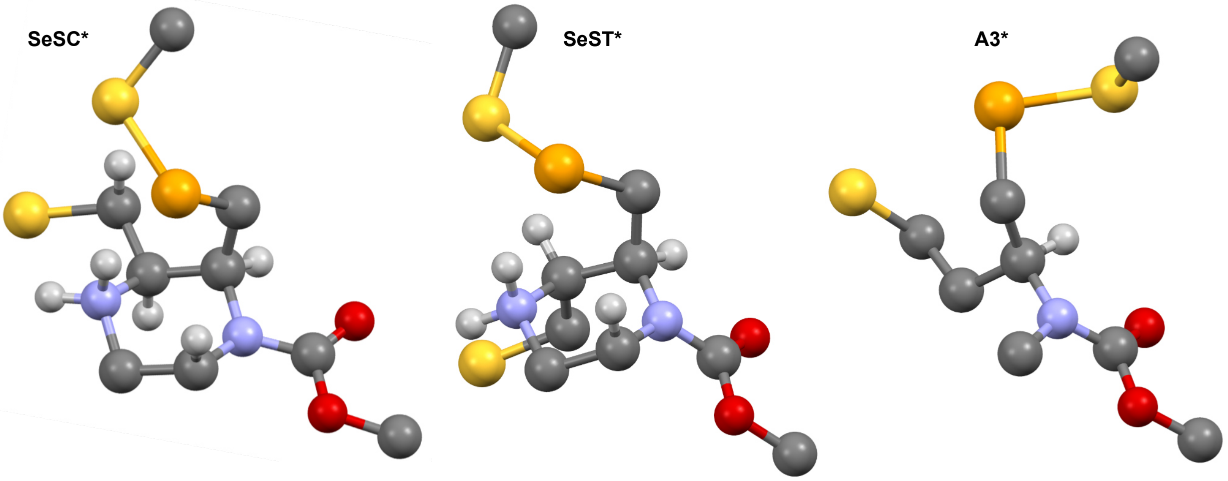

bond distance [Å]	Se - N*H* (B)	Se - N*H* ($TS^{B\text{-}C}$)	S - N*H* (B)	S - N*H* ($TS^{B\text{-}C}$)
SeSC*	2.24	2.08	2.38	2.42
SeST*	2.29	2.12	2.36	2.44

key adjustment to accommodate TS geometry: rotation of thiol

Figure S10 Conformations of intermediates of thiol addition. SeSC*, **SeST*** and **A3*** are shown in comparison. Water molecules that mediate proton transfer are omitted, and only selected hydrogen atoms are shown (thiols were modelled as -SH, the piperazine basic nitrogen as -NH_2^+-. For **SeSPs**, the addition intermediate has both chalcogens locked in a tricyclic conformation that is facilitated by the piperazine's free amine which acts as double hydrogen donor. For both **SeSC*** and **SeST***, to reach the B→C transition state towards full reduction (not shown) from B requires only slight structural changes, i.e., a contraction of the Se-*H*N bond distance and an elongation of the S-*H*N distance. This effective protonation at Se does not result from an elongation of the N-H bond, but a shift of the Se atom towards the hydrogen atom. To reach this sterically encumbered transition state, comparably large activation energies in comparison to **A3*** (where simple C-C bond rotation provides the required TS-geometry) are required. The structure of B(**SeST***) illustrates how the retro-cyclisation to regenerate the intact (oxidised) probe can not occur without energetically costly conformational changes in the piperazine core. This in turn may explain how **SeST**-type probes are readily reduced by monothiols.

General notes: (1) When comparing uncharged (**A3***) vs charged (**SeSC/T***) energies, implicit solvation in the model will never fully represent the actual picture in solution, especially for charged species (due to lack of directional H-bond interactions in the model, which will be relatively strong around the NH^+ moiety). This effect is most likely systematic for species with the same charge, but it will be different for charged and uncharged species. (2) For **SeST***, the structure of B, its transition state B→C and the reduced structure C all feature a 1,2-diaxial conformation with regards to the methylchalcogenide substituents. We are aware that this conformation should be disfavoured by ca. 3-4 kcal/mol when compared to the 1,2-diequatorial conformer and acknowledge that this observation might devalue quantitative data from these calculations).

3 General methods for probe evaluation

3.1 Cell-free assays

Determination of the **PQ** probes' redox properties are based on reduction-triggered fluorophore release. The turn-on of fluorescence was quantified using a plate reader (λ_{ex} = 355 nm, λ_{em} = 520 nm). Probes were dissolved in DMSO and stored as 10 mM stocks at 4 °C for at least 3 months. LC/MS analysis of DMSO stock solutions revealed that several rounds of freezing and thawing, and even storage at r.t. over several months were well tolerated and did not result in measurable decomposition of the probe. DMSO stock solutions (10 mM) dissolved in TE buffer (Tris-HCl (50 mM), EDTA (1 mM), pH = 7.4), giving a final concentration of 10 µM, with 1% DMSO. A black 96-well plate with black bottom was charged with probe and subjected to a variety of chemo- and bioreductants. Fluorophore release kinetics were monitored continuously or at set time points. Unless stated otherwise, in between measurements, the 96-well plates were incubated in a regular heating oven at 37 °C and in a H_2O-saturated atmosphere. Every experiment was run with *blank* control wells, i.e. 10 µM probe in TE buffer, to estimate residual fluorescence of non-activated probe or potential decomposition.

Chemical reductants

In vitro chemoreductant experiments were conducted according to the following protocol: 80 µL of probe (12.5 µM in TE buffer/1.25% DMSO) were placed into a 96-well plate and 20 µL of the respective reductant (TCEP: 0.1, 0.3, 1, 3, 10, 30, 100 equiv.; GSH or DTT: 1, 3, 10, 30, 100, 300, 1000 equiv.) were added. The plate was immediately placed into the plate reader to conduct continuous measurements for 30 min (60 s per cycles, 30 datapoints, 1 per minute). Note that since **SeSP**-based probes exhibited extraordinary activation kinetics, chemical reductant assays were conducted at ambient temperature. Raw data were processed using *Microsoft Excel* and *GraphPad Prism*, the former serving as platform for data assembly, organisation, and calculation of mean values for each data point. F(t) values were further processed by normalising each data point to $F^{TCEP}(t)$, using TCEP as an estimated reference for fast, quantitative probe reduction and activation. Data plotting was conducted using *Prism*.

Enzymatic reductants

Assessment of probe reactivity when challenged with key cellular reductants was conducted as follows: in case of two-component systems, the respective bioreductant (40 µL in TE) was placed into the corresponding wells and 50 µL of probe (20 µM in TE buffer/2% DMSO, to result in a final concentration of 10 µM and 1% respectively) were added. The reaction was initiated by addition of 10 µL of the reductive driving force, (NADPH or GSH; 10 µL, 1 mM in TE, 10 equiv) and the time point zero measurement was conducted.

Three-component experiments were prepared by first adding 40 µL of the reductant (TrxR/GR in TE (50 mM Tris-HCl, 1 mM EDTA, pH 7.5) to reach final concentration of 2 to 50 nM) and/or the corresponding native substrate (Trxs/Grxs in TE, to reach final concentrations of 0.01 to 10 µM). Consecutively, 50 µL probe (20 µM in TE buffer/2% DMSO, to reach final concentrations of 10 µM and 1% respectively) was added and the reaction was started by addition of 10 µL of 1 mM NADPH. TCEP (40 µL, 5 mM in TE, 20 equiv., final concentration of 200 µM) was added to a control-row for maximum activation and a first measurement (time point zero) was conducted right away.

Raw data was processed using *Microsoft Excel* and *GraphPad Prism*. As before, *Excel* was used for data assembly and calculation of mean values for each data point. In contrast to previous experiments, application of NADPH required an additional processing step to eliminate its background fluorescence which interferes with the readout of the fluorescent **PQ** cargo.[26] Absolute fluorescence values F(t) were therefore simply subtracted by $F^{NADPH}(t)$; NADPH control wells were run on every plate under the same conditions, rather than averaging values from other runs. NADPH-corrected absolute values were then further processed by normalising each data point to $F^{TCEP}(t)$, using TCEP as an estimated reference for fast, quantitative probe reduction and activation. Ultimate data visualisation in graphs was conducted in *Prism*.

Human recombinant thioredoxin 1 (Trx 1) (lyophilized), human recombinant glutaredoxin 1 (Grx 1) (lyophilized from 10 µL TE-buffer, pH 7.5), human thioredoxin reductase (TrxR) (1.5 mg/mL in 50% glycerol/TE-buffer, pH 7.5) and baker's yeast glutathione reductase (GR) (100 µM in 50% glycerol/TE-buffer, pH 7.5) were produced as previously described.[27,28]

Michaelis-Menten kinetics

To assess the quality of the probes as a TrxR substrate, classic Michealis-Menten kinetics were determined. In brief, 100 nM TrxR1 were mixed with 2 mM of NADPH (final concentrations 20 nM and 400 µM respectively) in a 96 well plate, and incubated at room temperature for 15 min. Subsequently, a dilution series of the probes was prepared in TE (50 mM Tris-HCl, 1 mM EDTA, pH 7.5) and added to the enzyme/NADPH mix. The absorbance at 340 nm was followed, by measuring every 30 s for 800 s. A no-TrxR control was included for each concentration and a 0 µM control was included for each probe. There was 1% DMSO as cosolvent present in the *N-series* and 5% DMSO in the *D-series*, due to solubilty problems of the amide models. The initial, linear slope of NADPH consumption was calculated as ΔA.

3.2 Cellular assays

General cell cultivation methods

Cells were grown in Dulbecco's modified Eagle's medium (DMEM: L-glucose (4.5 g/L), L-glutamine, L-pyruvate, phenol-red, $NaHCO_3$ (2.7 g/L); PAN Biotech) at 37 °C under 5% CO_2 atmosphere. Media were supplemented with 10% heat-inactivated fetal bovine serum, and Na_2SeO_3 (100 nM) unless stated otherwise. Selenium supplementation changes were always followed by at least 7 days of culturing the cells in the newly supplemented media before performing any experiments. Washing steps were performed in REcombinaDPBS (Merck GmbH, Darmstadt, Germany), cell detachment was performed using TrypLE™ Express (gibco Life Technologies Inc.). Cell growth was monitored using a Nikon Eclipse Ti microscope (Nikon Corp.).

Cell lines

A549 (DSMZ; ACC 107) cell lines were purchased from the German Collection of Microorganisms and Cell Cultures. TrxR knockout and reference mouse embryonic fibroblasts (MEF) were a kind gift from Marcus Conrad. MEFs isolated from conditional TrxR1 knockout mouse embryos, were immortalised by lentiviral transduction. *In vitro* deletion of TrxR1 was achieved by *Tat-Cre* induced recombination and verified by PCR and immunoblotting for TrxR1.[29] The stable expression of TrxR1 was validated by western blot and TrxR1 activity assays. All cell lines are tested regularly for mycoplasma contamination and only mycoplasma negative cells are used in assays.

Cellular activation and inhibition assays

A549 cells were seeded in 96-well plates (20.000 each well) (microplates, 96 well, F-bottom, black, clear bottom, ViewPlateTM-96 F TC, PerkinElmer) in 100 µL medium. After seeding, cells were incubated overnight and treated with probes (in 100% DMSO) to reach 1-100 µM final probe concentrations (1% final DMSO concentration) the next day. In brief: the media was removed and fresh media, containing the probes, was added to the cells. During the timecourse of the measurements, cells were kept at 37 °C under 5% CO_2 atmosphere and fluorescent signal was measured over 6 hours. For inhibition experiments, A549 cell lines were preincubated with TRi-1 (8, 16 µM from DMSO stock solutions, 50 µL medium, 0.23% DMSO) 3 h prior to probe addition or untreated (DMSO control), as non-inhibited internal control. After 3 hours of incubation, 10 µM probe (in 100% DMSO) was added in fresh media, to reach a final volume of 100 µL per well. Inhibitor concentrations given in the following refer to final concentrations (100 µL medium after probe addition).

Time-course fluorescence measurements were conducted to determine kinetics of cellular processing. Fluorescence readout of cell-free activity and/or cell assays was performed either using a *FluoStar Omega* plate reader from BMG Labtech or a Tecan Infinite M200 plate reader (ex/em 480bp10/520lp recording fluorescence intensity). Data was interpreted by representing the absolute, time-dependent fluorescence intensity F. [F(t) is raw fluorescence intensity; F* is an F(endpoint) value normalised to compare between conditions, defined as $F^* = [F(6\ h) - F(t_0)]/F_{max}$ where F_{max} is the largest raw F(6 h) value in the set of technical replicates. Cells were kept at 37 °C under 5 % CO_2 atmosphere between measurements.

4 Chemical probe activation assays (Figures S11-S16)

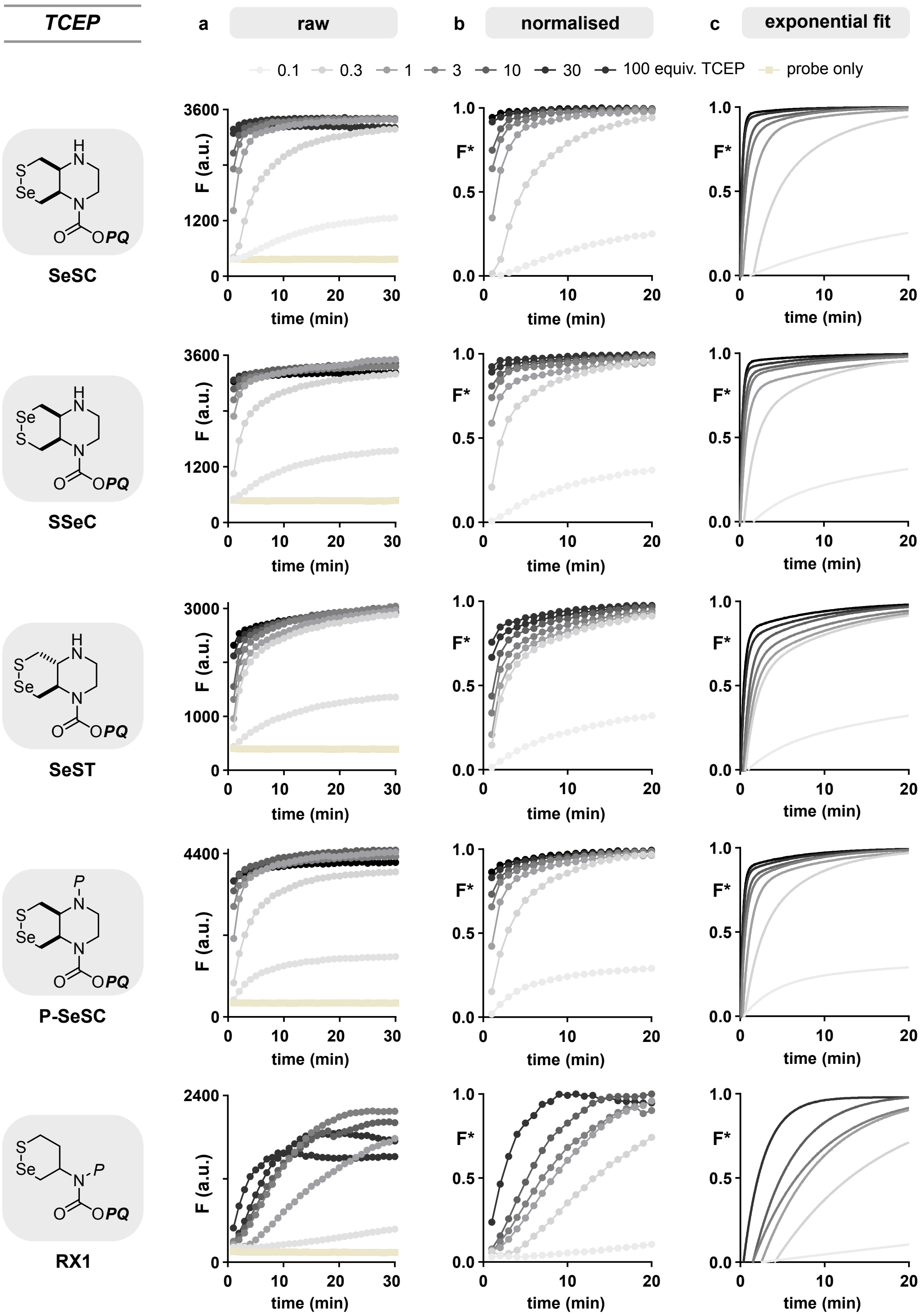

Figure S11 TCEP activation assays (1/2). Probes (at 10 μM, in aq. TE buffer, pH 7.4) were challenged with increasing concentrations of phosphine-reductant TCEP (from 0.1-100 equiv.) at 37 °C for 30 min. Fluorescence turn-on was quantified in a continuous plate reader measurement with a cycle length of 1 min. (**a**) Raw data with F(a.u.) including background fluorescence of probe without reductant. (**b**) Normalisation through (i) subtraction of background fluorescence, then (ii) normalisation by setting $F_{max} = 1.0$ for every individual dataset and (iii) exclusion of less relevant timepoints from 21-30 min. (**c**) Exponential fit, via a two-phase association (constrained to $F_0 = \geq -1$ and $F_{max} = 1$): $F(t) = F_{fast}\times(1-\exp(-\boldsymbol{k_{fast}}\times t)) + F_{slow}*(1-\exp(-\boldsymbol{k_{slow}}\times t))$, with $\boldsymbol{k_{fast}}$ representing an estimate for the TCEP-concentration dependent reaction rate. Due to the non-linear correlation between PQOH release and its fluorescent signal at low concentrations (< 1 μM, i.e., 10% probe activation), datapoints below the dye's precipitation threshold were excluded from the curve fit.

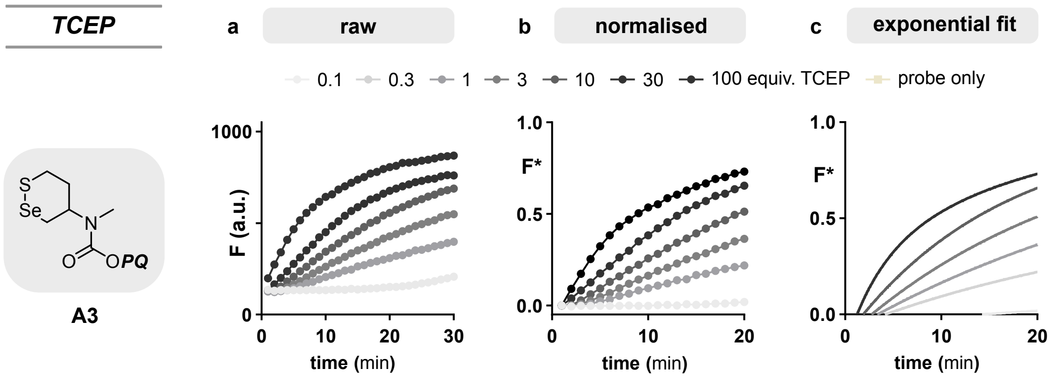

Figure S12 TCEP activation assays (2/2).

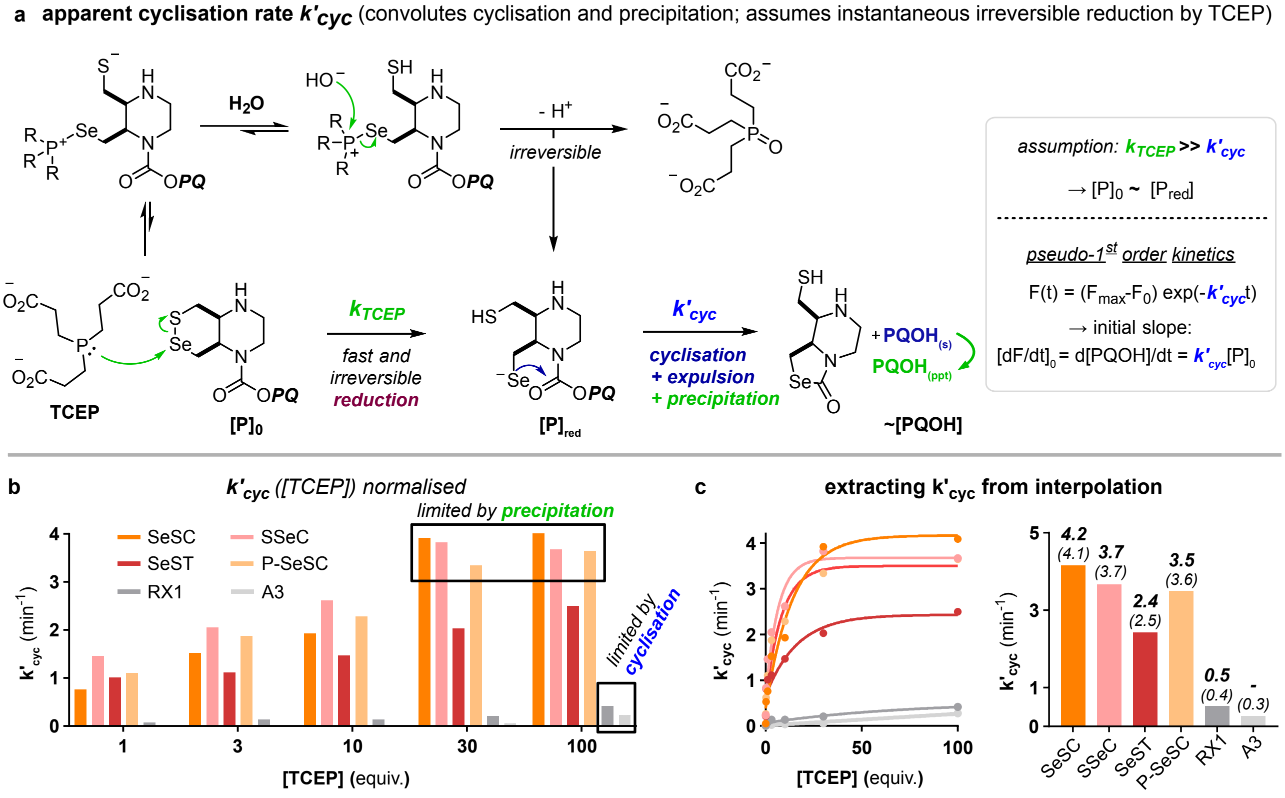

Figure S13 Extracted rate constants for carbamate cyclisation-expulsion. (**a**) Mechanism of probe activation by TCEP exemplified for **SeSC**. (**b**) Based on assumptions in (**a**): every probe's TCEP concentration-dependent ***k'***$_{cyc}$ was calculated as the ***k***$_{fast}$ rate constant for biexponential fitting to Fig S11-S12 data. (**c**) Those ***k***$_{fast}$([TCEP]) values were then fitted as one-phase associations to extract the TCEP concentration-independent plateau value of ***k'***$_{cyc}$. For reference, estimates of ***k'***$_{cyc}$ determined by simply taking the ***k***$_{fast}$ values with saturating TCEP (100 equiv.) are given in parentheses.

Note: The apparent rate constant k'_{cyc} we determine in TCEP assays represents the convolution of chalogenol cyclisation onto the carbamate, followed by expulsion of PQOH, followed by the buildup of released **PQ**-OH above its solubility limit, followed by its rate of precipitation from solution (since we monitor the 360/530 ESIPT fluorescence from the OH group that is only expressed in the anhydrous crystal environment, as is widely used by probes releasing this precipitating fluorogen [see e.g. the comercial probe ELF-97]). This apparent rate constant is therefore best seen as a *close fit to the rate constant for the rate-limiting step within this multi-step sequence* (since the initial concentration of free **PQ**OH is zero, non-linearity is strong at short timescales, and rates can be erratic depending on e.g. crystal formation kinetics and convection/diffusion rates - more details are given in our prior work on **RX1**[3] (in its *Supporting Note 5*)). The near-identical limiting apparent rates for the different **SeSPs** at high [TCEP] is most plausibly interpreted as showing that their true k_{cyc} values were far greater than the apparent k'_{cyc} rate, such that the apparent rates merely reflect the precipitation kinetics.

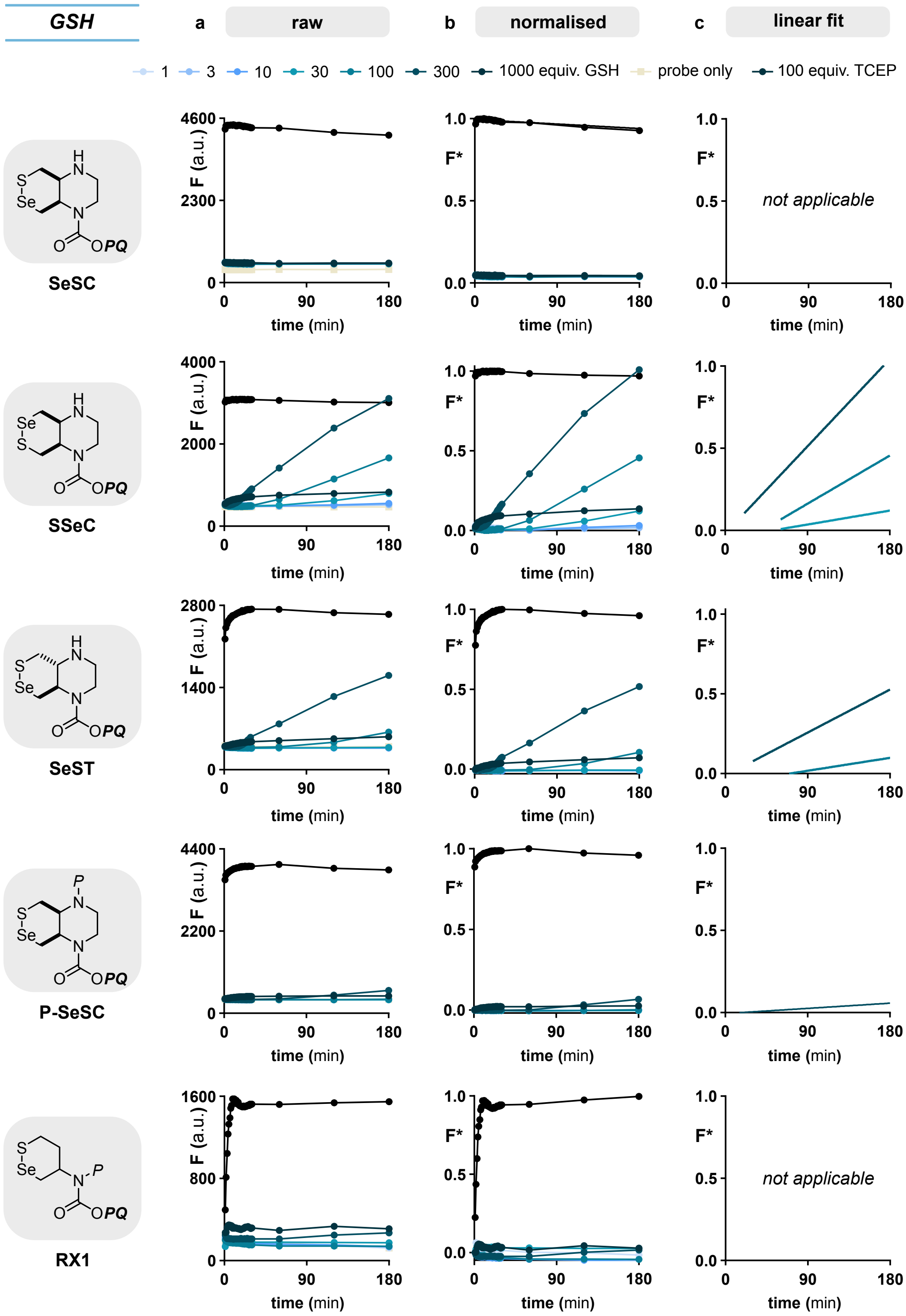

Figure S14 GSH activation assays. Probes (at 10 µM, in aq. TE buffer, pH 7.4) were challenged with increasing concentrations of monothiol-reductant GSH (from 1-1000 equiv.) at 37 °C for 180 min. During the first 30 min, fluorescence turn-on was quantified in a continuous plate reader measurement with a cycle length of 1 min; later timepoints were taken every hour. (**a**) Raw data with F(a.u.) including background fluorescence of probe without reductant. (**b**) Normalisation through (i) subtraction of background fluorescence, then (ii) normalisation by setting a probe's $F(TCEP)_{max}$ = 1.0 (from a parallel experiment). (**c**) Rates for GSH reduction (***k'***$_{GSH}$) were calculated using a linear fit: $F^*(t) = F_0^* + (k'_{GSH} \times t)$. Due to the non-linear generation of PQOH fluorescent signal for concentrations < 1 µM (10% activation), several concentrations (**SSeC**, **SeST**, **P-SeSC**) or entire datasets (**SeSC**, **RX1**) had to be excluded from kinetics analysis.

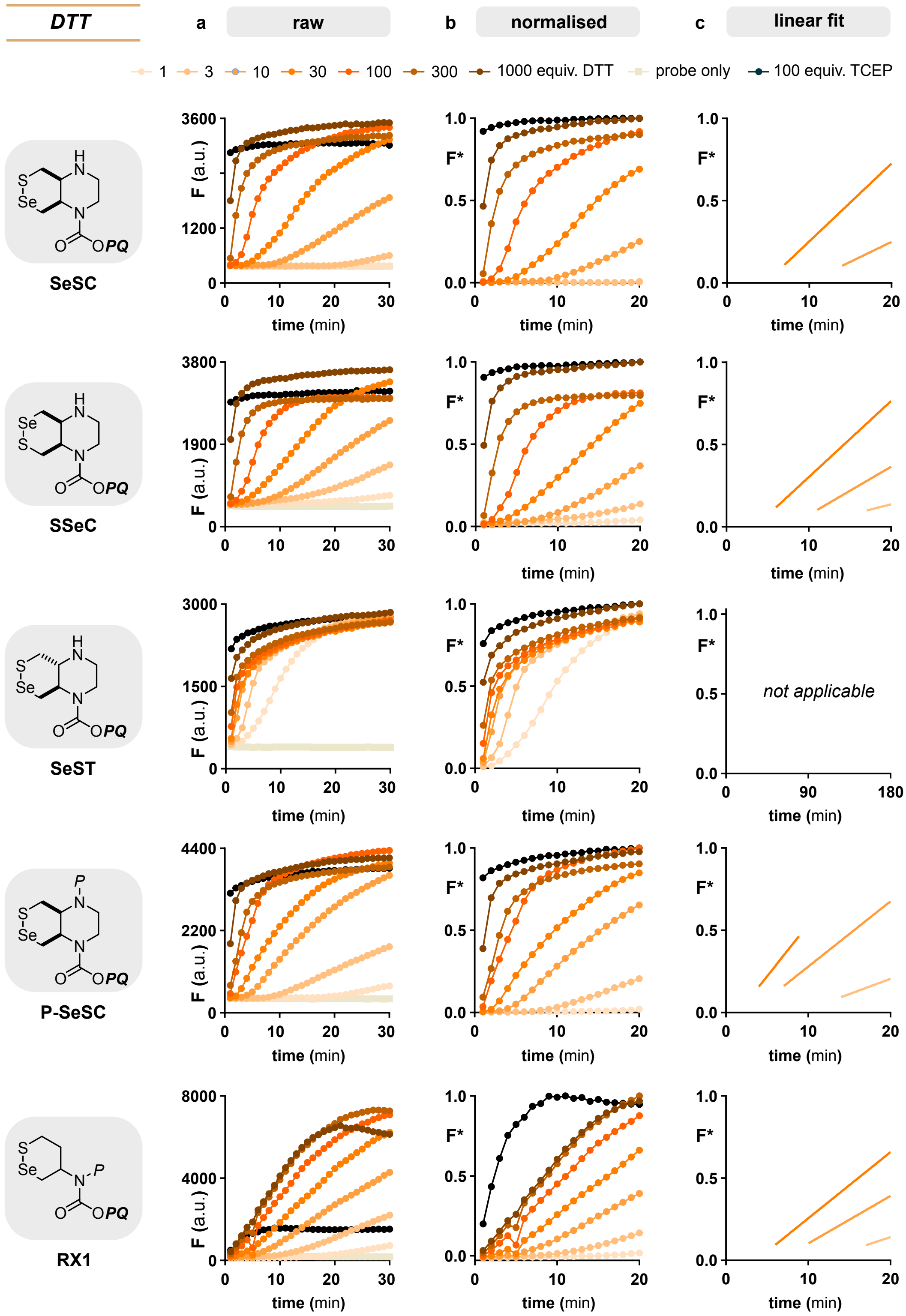

Figure S15 DTT activation assays. Probes (at 10 µM, in aq. TE buffer, pH 7.4) were challenged with increasing concentrations of dithiol-reductant DTT (from 1-1000 equiv.) at 37 °C for 30 min. Fluorescence turn-on was quantified in a continuous plate reader measurement with a cycle length of 1 min. (**a**) Raw data with F(a.u.) including background fluorescence of probe without reductant. (**b**) Normalisation through (i) subtraction of background fluorescence, then (ii) normalisation by setting a probe's $F(TCEP)_{max}$ = 1.0 (from a parallel experiment). (**c**) For datasets for which the overall reaction rate was orders of magnitude less than k'_{cyc}, the concentration-depedent reduction rate k'_{DTT} was extracted using a linear fit: $F^*(t) = F_0^* + (k \times t)$. Due to the non-linear generation of PQOH fluorescent signal for concentrations < 1 µM (10% activation), several concentrations (**SeSC**, **SSeC**, **P-SeSC**, **RX1**) had to be excluded from kinetics analysis. For **SeST**, activation rates with DTT approached k'(TCEP) for every concentration and could thus not be deconvoluted into k'_{cyc} and k'_{red}(DTT)

a **reduction cascade: kinetic steps**

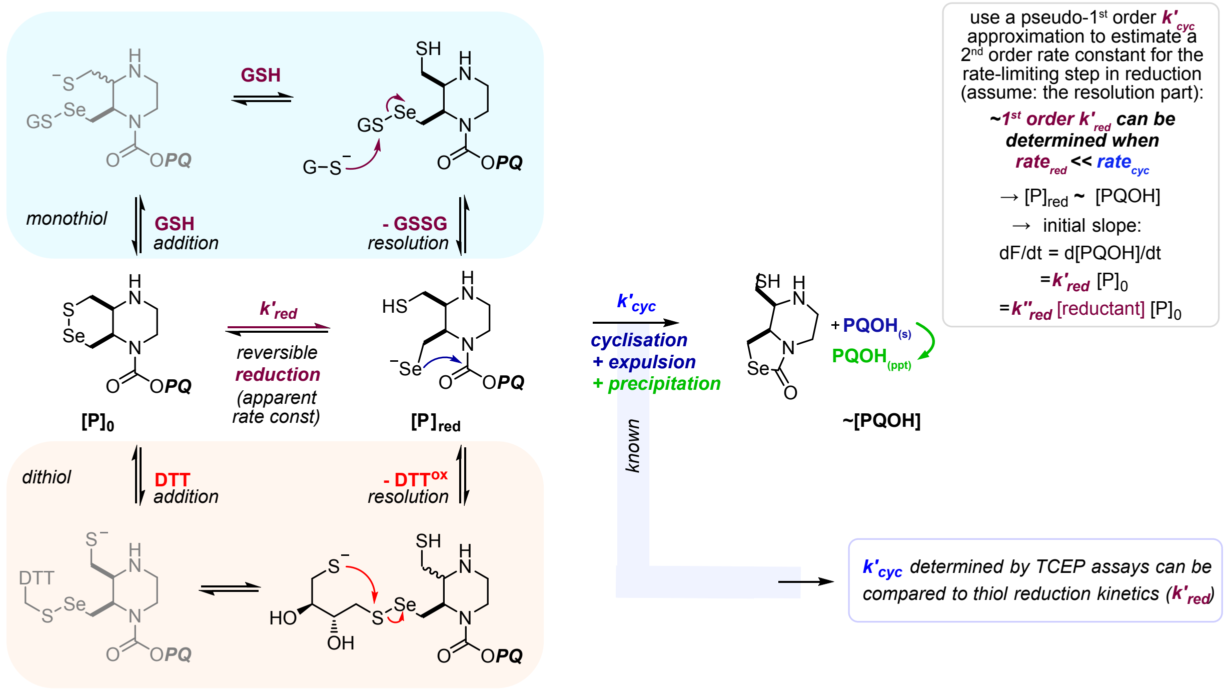

b

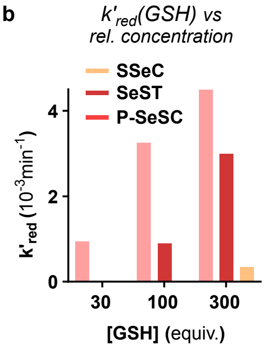

c **calculating GSH-concentration-normalised rate constants k''_{red}**

k'_{red} (10^{-4} min^{-1})	GSH (mM) 0.3	1	3	$k''_{red} = k'_{red}$ / [GSH]			k''_{red}(GSH) (min^{-1}mM^{-1})
**SSeC**	9.4	32.6	59.0	31.3	32.6	19.7	**0.0028**
**SeST**	-	9.0	29.9	-	9.0	10.0	**0.0010**
**P-SeSC**	-	-	3.50	-	-	1.2	**0.0001**

d

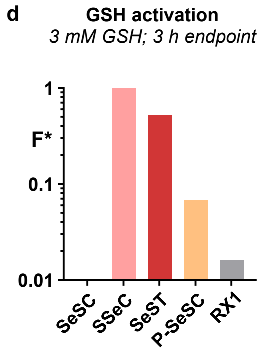

e

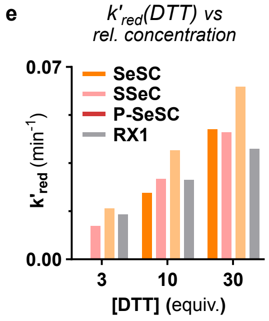

f **calculating DTT-concentration-normalised rate constants k''_{red}**

k'_{red} (10^{-2} min^{-1})	DTT (mM) 0.03	0.1	0.3	$k''_{red} = k'_{red}$ / [DTT]			k''_{red}(DTT) (min^{-1}mM^{-1})
**SeSC**	-	2.42	4.74	-	24.2	15.8	**0.020**
**SSeC**	1.22	2.92	4.62	40.7	29.2	15.4	**0.028**
**P-SeSC**	1.86	3.97	6.28	62.0	39.7	20.9	**0.041**
**RX1**	1.64	2.89	4.03	54.7	28.9	13.4	**0.032**

g

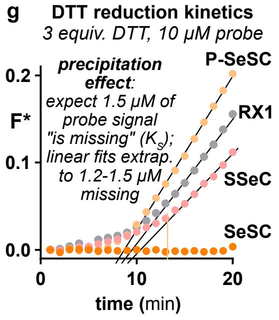

Figure S16 Extracted rates for reduction by GSH and by DTT. (**a**) Mechanism of probe activation with monothiols (GSH) and dithiols (DTT) exemplified for **SeSC**. (**b, e**) Concentration-dependent apparent rate constants for reduction by GSH or DTT, based on the linear fits from Figs S13 & S14. (**c, f**) Calculated reduction rate constants ***k''***$_{red}$(GSH) and ***k''***$_{red}$(DTT). Exact values should be treated cautiously - especially for DTT, where determination of ***k''***$_{red}$ from different concentrations did not afford comparable results. However, ***k'***$_{red}$s determined from these assays can be compared to ***k'***$_{cyc}$, in that they are orders of magnitude faster than ***k'***$_{cyc}$ and thus reduction is the rate-determining step for probe activation. (**d, g**) Qualitative, key findings of thiol reduction assays: Monothiol-resistance follows design considerations (**SeSC** completely inert to GSH), and dithiol-based reduction rates for **SeSC** are markedly lower than for **SSeC**, **P-SeSC** and **RX1**. For reference probe **A3**, DTT and GSH data from prior work[3] was used.

5 Enzymatic assays (Figures S17-S19)

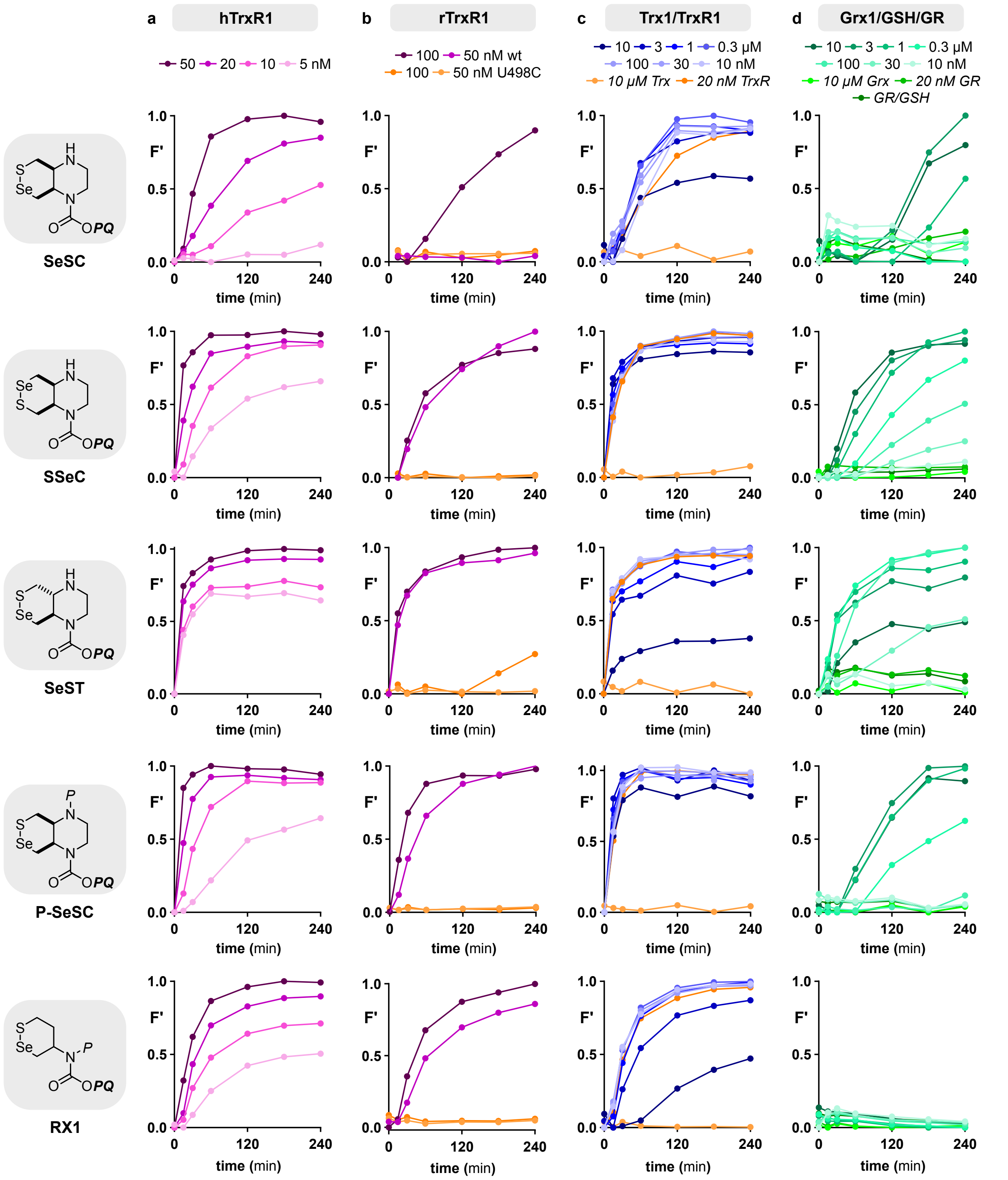

Figure S17 *In vitro* enzymatic assays with recombinant protein. Probe activation is evaluated as the time-dependent increase of the relative fluorescence signal from 0-4 h and probes were used as 10 µM solutions in TE-buffer. From all raw data, NADPH fluorescence (at λ_{ex} = 355 nm, λ_{em} = 520 nm) was subtracted. For normalisation (**F'**), the lowest value in each individual data set was set as 0, the highest value in the entire data set for, e.g., hTrxR assays with **SeSC**, was set as 1. (**a**) hTrxR1 titrations from 5-50 nM enzyme using NADPH (200 µM) as the native upstream reductant. (**b**) Probe activation by wildtype human hTrxR and rat rTrxR (both U498, C497) compared to mutant $rTrxR^{U498C}$.[30] 10 and 20 nM concentrations of rTrxR1 were tested, but not shown for all probes since they generally did not result in significant probe activation. For **SeSC** and **SSeC**, data points at 0 min were omitted for clarity. (**c**) Trx1 titration assays. The redoxin is applied at a range of concentrations (0 nM – 10 µM); NADPH (200 µM) and TrxR1 (20 nM) were applied as native upstream components. (**d**) Grx1 is applied at a range of concentrations (0 nM – 10 µM); NADPH (200 µM), GSH (100 µM) and GR (20 nM) were applied as native upstream components.

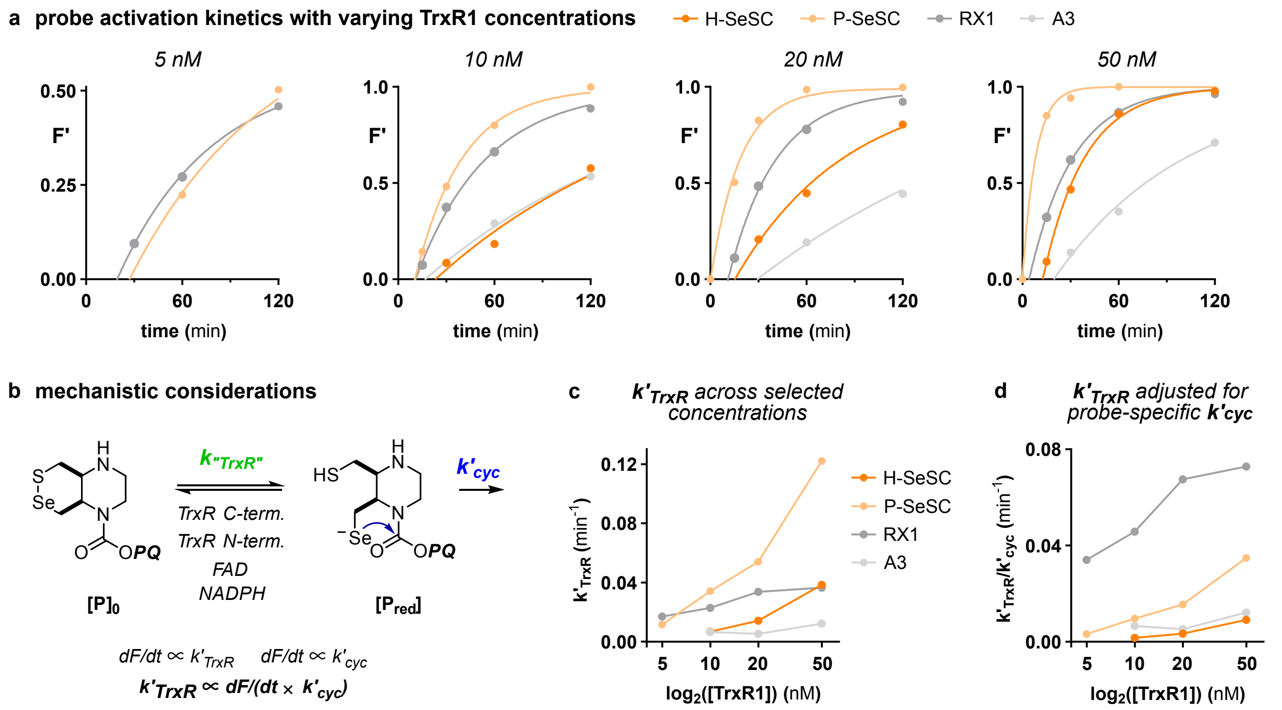

Figure S18 (1/2) TrxR1 activation kinetics for SeSC, **P-SeSC** and reference TrxR probes **RX1** and **A3**. Data for **A3** was taken from an earlier study.[3] (**a**) Concentration-dependent TrxR1 activation kinetics across probes. Individual datapoints were fitted via a two-phase association (constrained to $F_0 = \geq -1$ and $F_{max} = 1$): $F(t) = F_{fast}\times(1-\exp(-\boldsymbol{k_{fast}}\times t)) + F_{slow}*(1-\exp(-\boldsymbol{k_{slow}}\times t))$, with $\boldsymbol{k_{fast}}$ representing an estimate for the TrxR1-concentration dependent reaction rate. Due to the non-linear correlation between PQOH release and its fluorescent signal at low concentrations (< 1 µM, i.e., 10% probe activation), datapoints below the dye's precipitation threshold were excluded from the curve fit. Accordingly, data of **SeSC** and **A3** was not sufficient to extract their TrxR1 activation rates via this method. (**b**) Mechanistic challenge for TrxR1 assays: the "TrxR1 rate" that can be extracted from probe activation data is a combined rate through NADPH-reduction of TrxR's FAD cofactor, subsequent reductions of the *N*-, then *C*-terminal active sites and finally reduction of the probe. Since $\boldsymbol{k'_{cyc}}$ and $\boldsymbol{k'_{TrxR}}$ are within the same order of magnitude, above methods for analysis of reduction rates are not feasible. Instead, only qualitative analysis was conducted: (**c**) Concentration-dependent TrxR-system activation rates. Bicyclic thiaselenanes (**SeSC**, **P-SeSC**) outperform monocyclic analogs (**RX1**, **A3**) at higher enzyme concentrations (i.e., reduction equilibrium shifted towards reduced form), consistent with their much higher $\boldsymbol{k'_{cyc}}$. (**d**) Division of a probe's $\boldsymbol{k'_{TrxR}}$ by $\boldsymbol{k'_{cyc}}$ provides a qualitative measure for TrxR1 reduction rates.

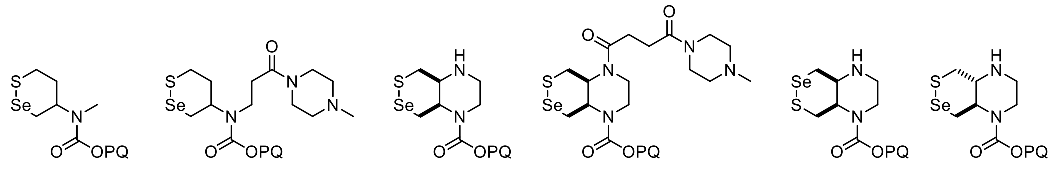

rel. rate (qualitative)	**A3**	**RX1**	**SeSC**	**P-SeSC**	**SSeC**	**SeST**
k_{cyc} (*via TCEP*)	–	–	++	++	++	+
k_{red} (*via DTT*)	+	+	o	+	+	+++
k_{retro} (*via GSH*)	++	+	+++	+	– –	–
k_{TrxR}	o	++	o	+	+	+++

Figure S18 (2/2) Qualitative comparison of individual activation rates for Se-centred probes. Green plus signs indicate fast reaction step rates, orange circles moderate rates, and red minus signs are slow rates, under typical settings.

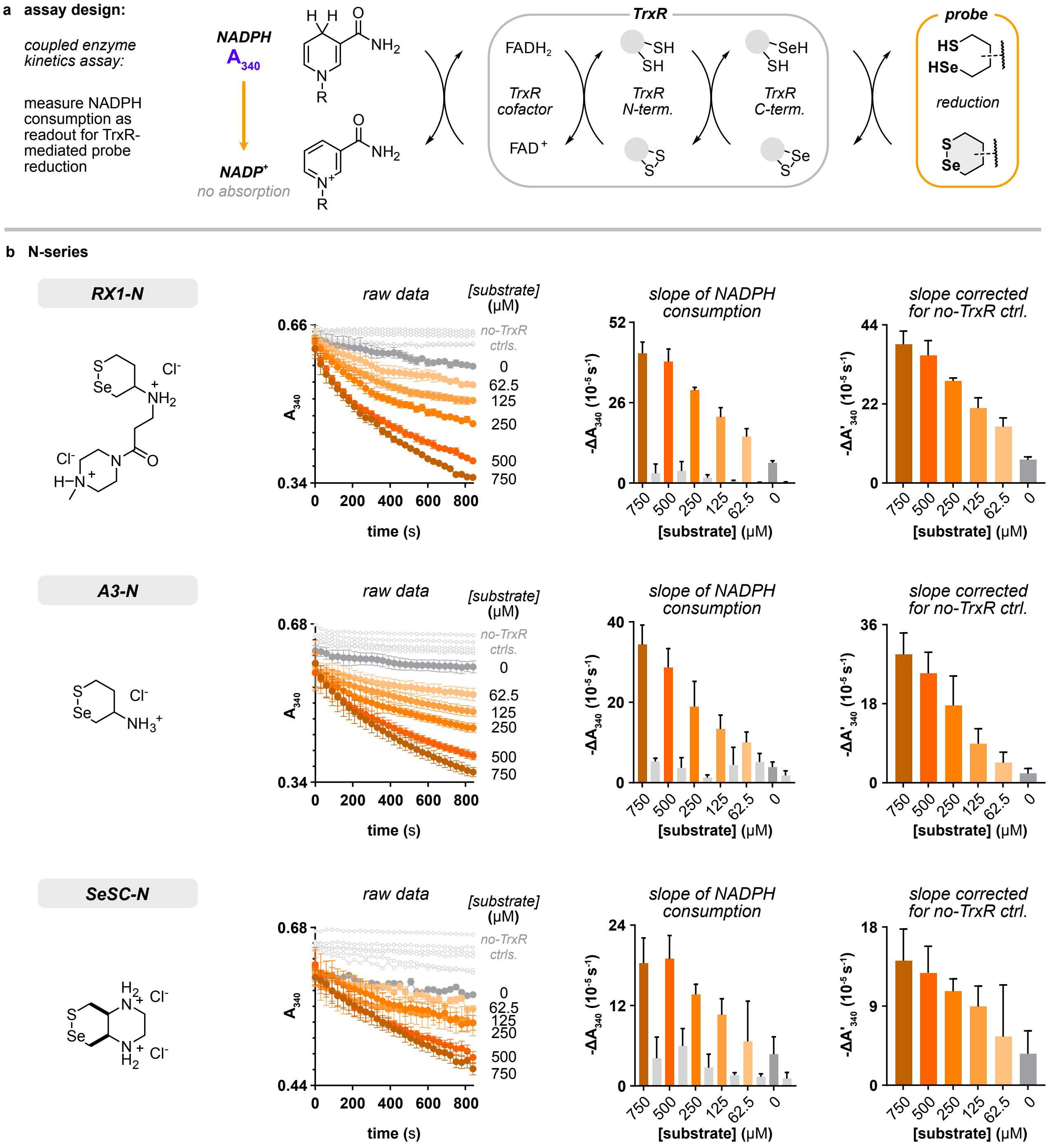

Figure S19 (1/2) Michaelis-Menten kinetics (**a**) Schematic overview of the NADPH-oxidation kinetics assay. NADPH functions as indirect reductant of the respective thiaselenane probe, coupled via TrxR's $FADH_2$ cofactor, its *N*-terminal active site and finally its *C*-terminal active site. (**b**) ***N-series****:* features the reducible 1,2-thiaselenane motifs of PQOH-based release probes. Non-release probes were applied at a range of concentrations (62.5 – 750 μM) and subjected to TrxR (20 nM) and NADPH (100 μM). 1% DMSO as cosolvent was present during the assay. Absorption at 340 nm was measured every 30 s ("raw data"), showing mean with SD of technical triplicates. The 0 μM control accounts for TrxR-catalysed baseline consumption of NADPH under aerobic, aqueous conditions, whereas the "no-TrxR" controls can pick up potential decomposition or reaction of NADPH with the SeS probes. The initial, linear slope of NADPH consumption was calculated as ΔA.

c D-series

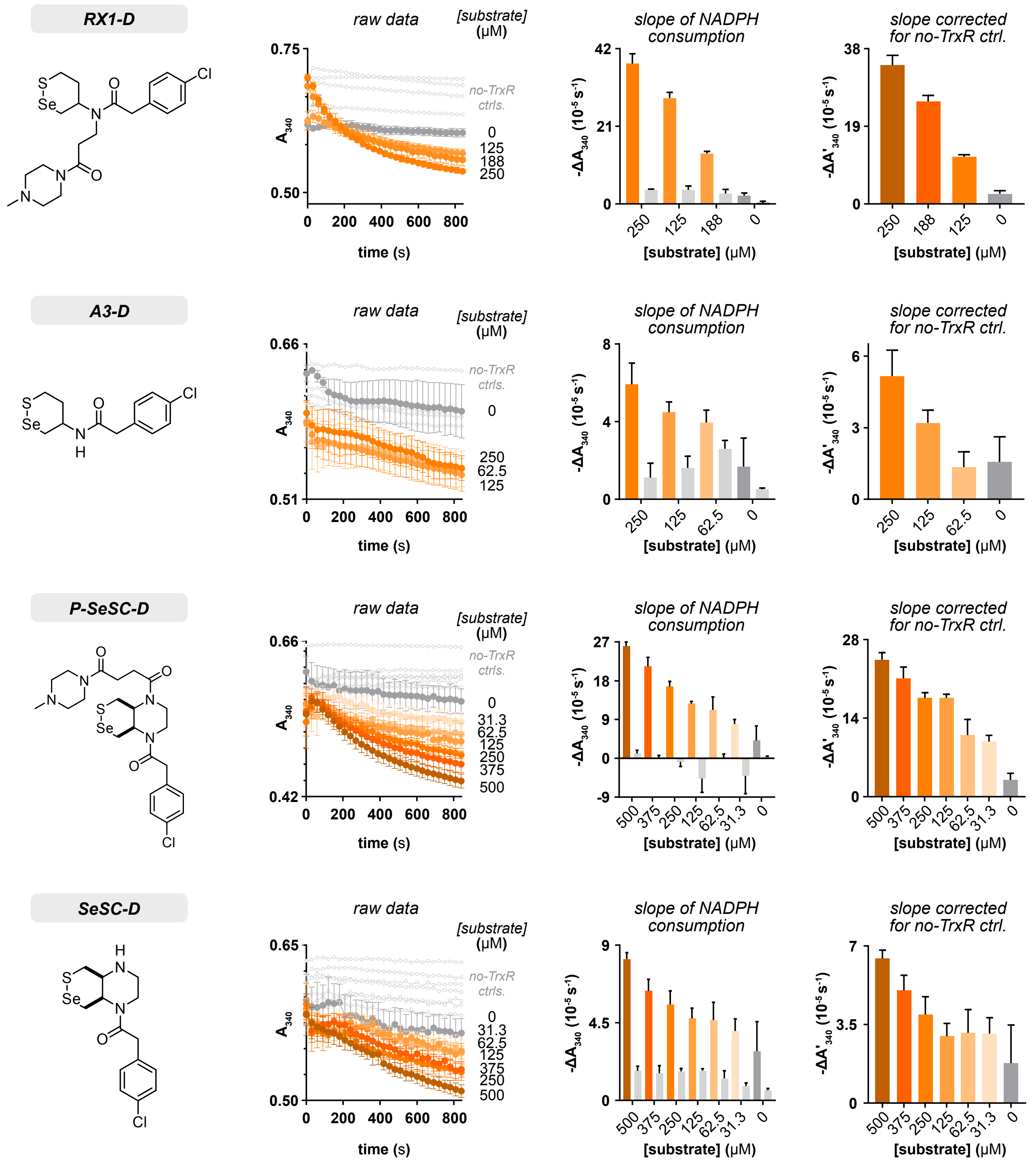

Figure S19 (2/2) Michaelis-Menten kinetics (**c**) ***D-series***: represents condensed, non-release mimics of PQOH-based probes. In contrast to the **N-series**, mono-acylation renders all members of the **D-series** rather insoluble in aqueous buffer. To address this, 5% of DMSO cosolvent was required. Still, particularly **RX1-D** and **A3-D** recurringly precipitated and prevented testing concentrations above 250 μM. Data quality in this series overall does not match the **N-series**, and we advise to solely draw qualitative conclusions from it.

Note on potential enzymatic off-targets

With regards to other selenoproteins as potential offtargets: we previously tested the activation of the more labile monocyclic RX1 by the cytosolic GPx1 and there was no fluorescence detected (**Figure S15** in Zeisel et al, Chem 2022[31]). GPx4 was not an activator either; which makes sense as it is a monoselenol so cannot complete a bimolecular reduction. In Supporting Note 6 of that paper[31], we had discussed the selenoproteins as follows: *"As well as the mono/dithiols we assessed here, we were most worried about other selenol proteins: because initial exchange by any RSeH could have similar thermodynamics and kinetics as the initial exchange by TrxR (there are no low-molecular-weight selenol bioreductants at significant concentrations in cells). Luckily, the human proteome codes for just 25 selenol-bearing proteins, of which many have restricted and/or low expression (much information is to be found in Gladyshev et al). Of these, we considered that two monoselenol glutathione peroxidases (GPx1/4) had sufficiently broad expression at significant levels, to potentially confound TrxR-selectivity; and their ability to react with e.g. cumene hydroperoxide (GPx1) and cholesterol hydroperoxide (GPx4) indicates that they can accept hydrophobic small molecules as substrates. However, they are monoselenol enzymes, so they would require on-protein cyclisation to cause signal generation. We therefore challenged both RX1 and G1 with GPx1 in a titration assay (100-1000 nM) using GSH (200 µM, 20 equiv.) as the native upstream component to recover oxidised GPx1 (Fig S15a). None of the applied conditions resulted in measurable probe activation classifying both thiaselenane-probes inert to this monoselenol enzyme. Although it is likely that GPx1 is simply not able to react with larger molecules due to its sterically confined active site, this clearly excludes GPx1 as potential bioreductant of RX1. The relative contributions of GPx1's specificity for its native substrate, or of the general inhibition of polar cyclisation of a charged chalcogenide (more difficult for the less acidic thiol of the G series, rather than the selenide of the A series), remain open for further testing - as does the possibility of profiling all other GPxs and all other monoselenols. Focusing instead on selenolthiol enzymes, there are only very few other than TrxR, at any expression level. The enzyme methionine-R-sulfoxide reductase (MsrB1) is one selenolthiol-active-site enzyme, however it has very high substrate specificity for methionine-R-sulfoxide residues in proteins."* Here we assume that whatever did not reduce the more active RX1 will not reduce the more resistant SeSC. A logical question is to ask whether TrxR2, the mitochondrial isoform, can activate RX1 or SeSCs. Genetic knockout of this enzyme is lethal and thus not feasible as a way of probe validation, and chemical inhibitors of this enzyme are still lacking. In vitro assays with isolated TrxR2 from our prior work (Chem 2022) indicate that TrxR2 can indeed activate selenenyl sulfide probes, but *much less efficiently* than TrxR1 (only tested at pH~7.4); and combined with the vastly lower overall expression of TrxR2 compared to TrxR1, though, we expect that TrxR2 is an insignificant reaction partner in cells (matches also the TrxR1 knockout cell line result).

There are many other redox-active-thiol proteins that might be considered as potential offtargets, e.g. peroxiredoxins (Prxs). Since Prxs do not leverage an intramolecular dithiol mechanism as do Grxs and Trxs, we believe that they should not be able to outcompete the retro rate of cyclic selenenyl sulfide probes, even though Prxs' interlocked, dimer-like mechanism likely results in more efficient reduction of substrates than termolecular reductions with two equivalents of non-associated thiols (i.e. Prx reduction molecularity expected to fall between that for monothiols and for vicinal dithiols, which could be assessed with isolated Prxs in future experiments).

It must be remembered though that this is a somewhat hypothetical discussion: the major indication of cellular selectivity remains unquestioned, in that the knockout of the single enzyme TrxR1 is sufficient to completely abolish SeSC probe signal - thus although potential offtargets do exist, our data contradict that any of them may be actually relevant here.

6 Cellular assays (Figures S20-S22)

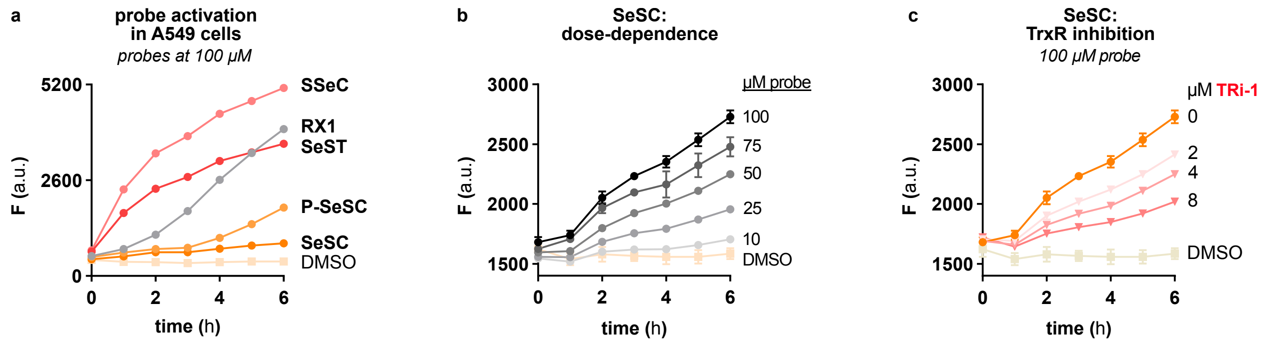

Figure S20 ***A549 cells.*** **(a)** Preliminary cellular assays in A549 cells testing all novel thiaselenane probes in a single biological replicate. The non-reducible negative/probe hydrolysis control C1 confirmed the cellular stability of PQOH-tertiary carbamate probes in an earlier study.[3] Monothiol-labile mechanistic controls **SSeC** and **SeST** (for the hypothesis that a net trimolecular monothiol reduction pathway would be needed to gain TrxR selectivity) were activated to very high levels while not being primarily dependent on TrxR (Figure S22). It was clear that much of their cellular signal reports on other redox-active species, whether GSH, other monothiols, Grxs, or any of the other redoxin superfamily members. Nevertheless, we tentatively assign their signal to monothiols, since **SSeC** and **SeST** gave almost identical cellular signal time courses as each other: recalling their near-identical monothiol lability profiles (Figure 5c), not their very different vicinal dithiol labilities (Figure 5d). (**b**) Dose-response of **SeSC** from 10-100 µM (**c**) TrxR inhibition studies using 2-8 µM of best-in-class TrxR1 inhibitor TRi-1.[32]

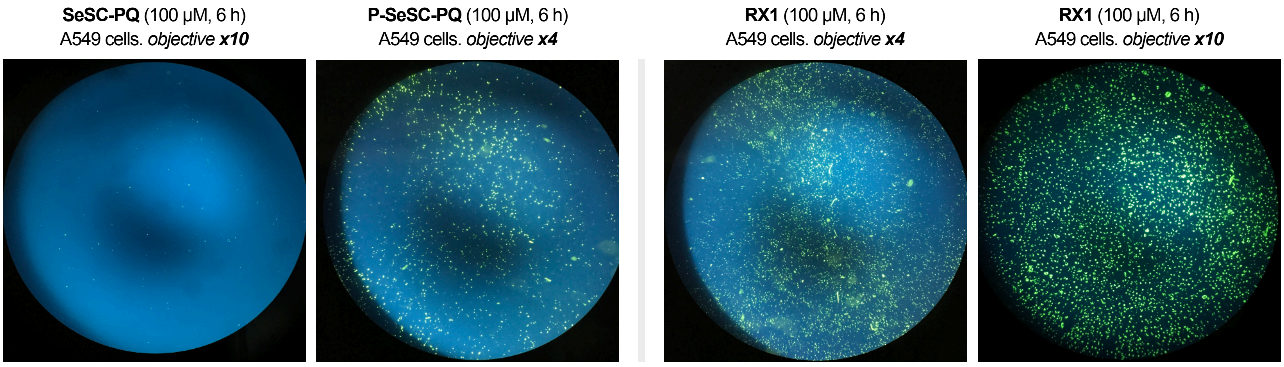

Figure S21 ***Fluorescence microscopy.*** Intracellular formation of solid green-fluorescent PQOH precipitates corresponding to a time-dependent fluorescence increase was confirmed using a Nikon Eclipse Ti2 upright microscope (ex/em 355bp50/410lp). Qualitative images (mobile phone camera) indicate the cell-marking performance of **SeSC**, **P-SeSC** and reference probe **RX1**.

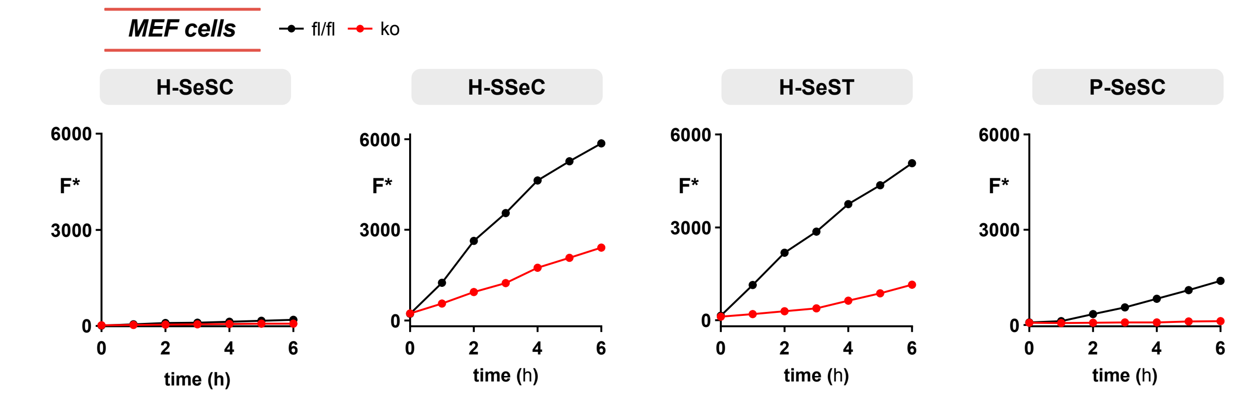

Figure S22 ***MEF cells (wildtype and TrxR-ko)***. Time-dependent probe activation (single biological replicate, mean of raw fluorescence values of technical replicates, corrected for DMSO background baseline signal - **F*** = F(t, probe)-F(t, DMSO)) in selenium-supplemented MEF cell lines with altered TrxR1-availability. Activation of **SeSC** in these cell lines was below a reliable detection limit.

7 Organic synthesis

Instrumentation

High resolution mass spectrometry (**HRMS**) was conducted either using a *Thermo Finnigan LTQ FT Ultra FourierTransform* ion cyclotron resonance spectrometer from *ThermoFisher Scientific GmbH* applying electron spray ionisation (ESI) with a spray capillary voltage of 4 kV at temperature 250 °C with a method dependent range from 50 to 2000 u or a *Finnigan MAT 95* from *Thermo Fisher Scientific* applying electron ionisation (EI) at a source temperature of 250 °C and an electron energy of 70 eV with a method dependent range from 40 to 1040 u. All reported *m/z* values refer to positive ionisation mode, unless stated otherwise.

Nuclear magnetic resonance (**NMR**) spectroscopy was performed using a *Bruker Avance* (600/150 MHz, with *TCI cryoprobe*) *or a Bruker Avance III HD Biospin* (400/100 MHz, with BBFO cryoprobeTM) from Bruker Corp. either at 400 MHz or 500 MHz. NMR-spectra were measured at 298 K, unless stated otherwise, and were analysed with the program *MestreNova 12* developed by *MestreLab Ltd*. ^{1}H-NMR spectra chemical shifts (δ) in parts per million (ppm) relative to tetramethylsilane (δ = 0 ppm) are reported using the residual protic solvent ($CHCl_3$ in $CDCl_3$: δ = 7.26 ppm, DMSO-d_5 in DMSO-d_6: δ = 2.50 ppm, CHD_2OD in CD_3OD: δ = 3.31 ppm, CHD_2CN in CD_3CN: δ = 1.94 ppm) as an internal reference. For ^{13}C-NMR spectra, chemical shifts in ppm relative to tetramethylsilane (δ = 0 ppm) are reported using the central resonance of the solvent signal ($CDCl_3$: δ = 77.16 ppm, DMSO-d_6: δ = 39.52 ppm, CD_3OD: δ = 49.00 ppm, CD_3CN: δ = 1.32, 118.26 ppm) as an internal reference. For ^{1}H-NMR spectra in addition to the chemical shift the following data is reported in parenthesis: multiplicity, coupling constant(s) and number of hydrogen atoms. The abbreviations for multiplicities and related descriptors are s = singlet, d = doublet, t = triplet, q = quartet, or combinations thereof, m = multiplet and br = broad.

High Performance liquid chromatography (**HPLC**) coupled with mass spectrometry (**LRMS**). Analytical HPLC-MS was performed on an Agilent 1100 SL with (a) a binary pump to deliver H_2O:MeCN eluent mixtures containing 0.1% formic acid at a 0.4 mL/min flow rate, (b) YMC-Triart C18 column (3.0 µm; 50 mm × 3 mm) maintained at 40 °C (c) an Agilent 1100 series diode array detector, (d) an Agilent LC/MSD iQ mass spectrometer. Typically, a linear gradient of H_2O:MeCN from 90:10 to 0:100 for 5 min, followed by 2 min of a 0:100 flush was employed; the column was then (re)equilibrated with a 90:10 eluent mixture for 2 min.

Preparative HPLC (**prepHPLC**) was performed on an Agilent 1200 SL with a binary pump to deliver H_2O:MeCN eluent mixtures containing 0.1% formic acid at 20 mL/min flow rate. We used an Agilent-Zorbax C18 column (10.0 µm; 250 mm × 30 mm) maintained at room temperature, an Agilent 1200 series diode array detector, and an Agilent 1200 series fraction collector.

X-Ray crystallography (**X-Ray**). The X-ray intensity data were measured on a Bruker D8 Venture TXS system equipped with a multilayer mirror monochromator and a MoK_α rotating anode X-ray tube (λ = 0.71073 Å). The frames were integrated with the Bruker SAINT software package. Data were corrected for absorption effects using the Multi-Scan method (SADABS). The structure was solved and refined using the Bruker SHELXTL Software Package.[33] All C-bound hydrogen atoms have been calculated in ideal geometry riding on their parent atoms while the N-bound hydrogen atom has been refined freely. The structure has been refined as an inversion twin (BASF 0.04). The figure has been drawn at the 50% ellipsoid probability level.[34]

Laboratory techniques

Unless stated otherwise, all reactions were performed without precautions in regard of potential air- and moisture-sensitivity and were stirred with Teflon-coated magnetic stir bars. For work under inert gas (nitrogen) atmosphere, a Schlenk apparatus equipped with a liquid nitrogen trap and a high vacuum pump from Vacuubrand GmbH were used. Air- and moisture-sensitive liquids were transferred via syringe or cannula. Organic solutions were concentrated by rotary evaporation, the water bath temperature was kept at 40 °C. Reactions were monitored by thin layer chromatography (TLC) on TLC plates (*Si 60 F254 on aluminium sheets*) provided by Merck GmbH and visualised by UV irradiation and/or $KMnO_4$ stain (3.0 g $KMnO_4$, 20 g K_2CO_3, 0.30 g KOH, 0.30 L H_2O). For solvent evaporation, a *Laborota 400* from Heidolph GmbH equipped with a vacuum pump was used. The term “column chromatography” refers to either (a) manual flash column chromatography conducted under positive nitrogen pressure using *Ceduran® Si 60 silica gel* (35−70 µm) from Merck GmbH as stationary phase, or (b) purification on a Biotage Selekt system using prepacked silica cartridges purchased from Biotage. All eluent and solvent mixtures are given as volume ratios unless otherwise specified.

Materials

Commercial sovents and reagents were obtained from Sigma-Aldrich, TCI, BLDpharm or abcr, were used as received and without purification. Anhydrous tetrahydrofuran (THF), dichloromethane (CH_2Cl_2), acetonitrile (CH_3CN) and dimethylformamide (DMF) were provided by Acros and were stored under argon atmosphere and dried over molecular sieves. TLC control, extractions and column chromatography were conducted using distilled, technical grade solvents. The term hexanes refers to distilled, technical-grade crude isohexane. Sodium (tert-butylsulfonyl)chloroamide was prepared according to Sharpless,[35] and was used in 50% purity, according to NMR. **RX1**, **A3**, **RX1-N** and **A3-N** were synthesised according to our established protocols.[36]

2-((1,1-dimethylethyl)sulfonamido)-3-((2-nitrophenyl)sulfonamido)butane-1,4-diyl dimethanesulfonate (S1)

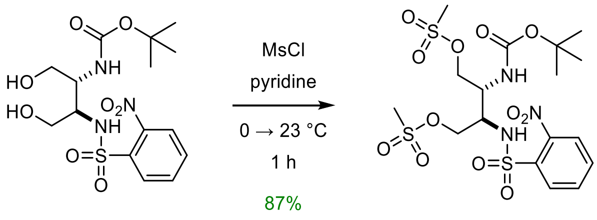

Boc-Ns diol[37] (0.10 g, 0.25 mmol, 1.0 equiv.) was dissolved in pyridine (1.0 mL, 0.25 M), cooled to 0 °C and MsCl (57 µL, 0.74 mmol, 3.0 equiv.) was added carefully. A colour change from colourless to bright yellow occurred. The reaction was allowed to warm to room temperature and stirred for 1 h, upon which TLC control indicated full conversion of the starting material. The reaction mixture was diluted with CH_2Cl_2 (10 mL) and transferred into a separatory funnel. The organic layer was washed with HCl (2 M, 3×5 mL) and brine, dried over $MgSO_4$, filtered, and concentrated *in vacuo*, affording **S1** as a pale-yellow foam (0.12 g, 0.21 mmol, 87%) without requiring further purification. Analytical data match the literature.[37]

Notes: (1) Perfoming the reaction in CH_2Cl_2 (0.1 M) using pyridine (5.0 equiv.) as base results in a complex reaction mixture. (2) The dimesylate is very prone to decomposition and should immediately be used for follow-up transformations. Removal of the solvent after workup should ideally be done under a stream of nitrogen; or, at larger scales, using a rotary evaporator with a water bath not exceeding 30 °C.

tert-Butyl 1'-((2-nitrophenyl)sulfonyl)-[2,2'-biaziridine]-1-carboxylate (S10)

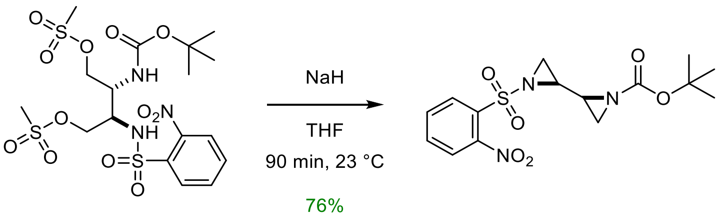

Dimesylate **S1** (0.20 g, 0.36 mmol, 1.0 equiv.) was dissolved in anhydrous THF (4.0 mL, 0.1 M), and NaH (36 mg, 0.89 mmol, 2.5 equiv.) was added. The reaction was stirred at 23 °C for 30 min, and another batch of NaH (36 mg, 0.89 mmol, 2.5 equiv.) was added. The reaction was stirred for another hour, upon which LCMS analysis indicated full conversion of the starting material. H_2O (10 mL) was added, and the reaction was transferred into a separatory funnel. The aqueous layer was extracted with EtOAc (2×10 mL), and the combined organics were dried over $MgSO_4$, filtered, and concentrated *in vacuo*. The residue was washed with heptane (3×1 mL), affording biaziridine **S10** (0.10 g, 0.27 mmol, 76%) as a colourless film.

Note: The biaziridine is very prone to decomposition. During initial screening of reaction conditions, reaction control by LCMS often suggested good yields, the surprisingly low isolated yields were likely due to decomposition on non-basified SiO_2. In addition, one batch of crude **S10** even decomposed before chromatographic purification.

TLC R_f = 0.44 (hexanes/EtOAc 1:1). **^{1}H NMR** (400 MHz, $CDCl_3$): δ (ppm) = 8.24 – 8.18 (m, 1H), 7.80 – 7.70 (m, 3H), 3.32 (ddd, J = 7.0, 4.7, 3.0 Hz, 1H), 2.93 (d, J = 7.0 Hz, 1H), 2.76 (dt, J = 6.3, 3.3 Hz, 1H), 2.55 (d, J = 4.7 Hz, 1H), 2.31 (d, J = 6.2 Hz, 1H), 2.10 (d, J = 3.5 Hz, 1H), 1.41 (s, 9H). **^{13}C NMR** (101 MHz, $CDCl_3$): δ (ppm) = 161.6, 148.6, 134.6, 132.3, 131.8, 131.5, 124.4, 81.8, 39.2, 34.7, 34.3, 30.2, 27.8. **HRMS** (ESI): $C_{15}H_{19}N_3NaO_6S^+$ $[M+Na]^+$: calc. m/z 392.08868, found: 392.08865.

tert-Butyl 2-((1-((2-nitrophenyl)sulfonamido)-2-selenocyanatoethyl)aziridine-1-carboxylate (S11)

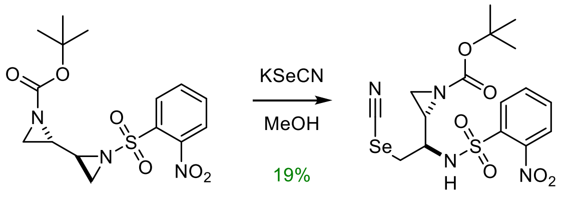

To a suspension of biaziridine **S10** (40 mg, 0.11 mmol, 1.0 equiv.) in MeOH (0.50 mL, 0.2 M), KSeCN (20 mg, 0.14 mmol, 1.3 equiv.) was added at room temperature. Dissolution of all solids within a few minutes coincided with full conversion to

the selenocyanate intermediate, as suggested by TLC. The reaction mixture was diluted with EtOAc (5 mL) and transferred into a separatory funnel. The organic layer was washed with H_2O (5 mL) and brine (3 mL), dried over $MgSO_4$ and filtered. The residue was purified by chromatography (SiO_2, 20% EtOAc in hexanes initially, grading to 50% EtOAc) affording selenocyanate **S11** as a light yellow solid (10 mg, 21 µmol, 20%).

Note: We again assign the discrepancy between "crude LCMS yield" (>80%) and isolated yield after chromatography to decomposition on silica.

TLC R_f = 0.52 (hexanes/EtOAc 1:1). **^{1}H NMR** (400 MHz, $CDCl_3$): *δ* (ppm) = 8.19 – 8.15 (m, 1H), 7.94 – 7.89 (m, 1H), 7.82 – 7.74 (m, 2H), 5.73 (d, J = 7.9 Hz, 1H), 4.07 – 3.96 (m, 1H), 3.29 (d, J = 6.6 Hz, 2H), 2.72 (dt, J = 6.1, 3.5 Hz, 1H), 2.36 (d, J = 6.1 Hz, 1H), 2.21 (d, J = 3.5 Hz, 1H), 1.43 (s, 9H). **^{13}C NMR** (101 MHz, $CDCl_3$): *δ* (ppm) = 161.2, 147.7, 134.2, 134.1, 133.5, 130.8, 125.8, 101.1, 82.7, 53.9, 38.3, 32.6, 29.8, 27.9. **HRMS** (ESI): $C_{16}H_{19}N_4O_6SSe^-$ [M-H]$^-$: calc. m/z 475.01960, found: 475.01930.

(1-((2-nitrophenyl)sulfonyl)aziridine-2,3-diyl)dimethanol (S4)

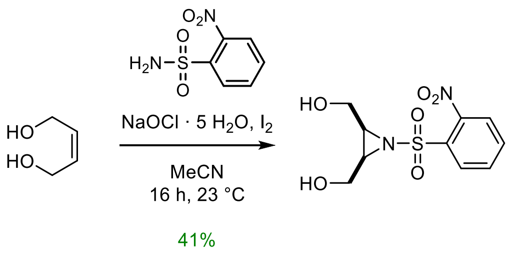

(Z)-But-2-ene-1,4-diol (0.30 g, 3.4 mmol, 1.0 equiv.) was dissolved in MeCN (15 mL, 0.2 M), followed by sequential addition of 2-nitrobenzenesulfonamide (0.76 g, 3.8 mmol, 1.1 equiv.), sodium hypochlorite pentahydrate (0.62 g, 3.8 mmol, 1.1 equiv.) and I_2 (86 mg, 0.34 mmol, 0.1 equiv.). The resulting, pale yellow heterogeneous mixture was stirred at room temperature for 16 h. The reaction was quenched by adding $Na_2S_2O_3$ (1 M aq., 50 mL) and the aqueous layer was extracted with EtOAc (80 mL). The combined organic extracts were washed with brine (0.10 L), dried over anhydrous $MgSO_4$ and concentrated under reduced pressure. The colourless residue was purified via chromatography (SiO_2, EtOAc) affording aziridine **S4** as a colourless solid (0.41 g, 1.4 mmol, 41%).

Notes: (1) TLC analysis should always be performed using both UV and $KMnO_4$ stain, since a non-UV active species elutes right after the aziridine (i.e. slightly lower R_f) (2) **S4** can also be purified by recrystallisation from EtOAc. (3) **S4** appeared to decompose when left to sit for > days in a solution of DMSO-d_6.

TLC R_f = 0.33 (EtOAc). **^{1}H NMR** (400 MHz, DMSO-d_6): *δ* (ppm) = 8.21 (dd, J = 7.8, 1.5 Hz, 1H), 8.04 (dd, J = 7.9, 1.4 Hz, 1H), 7.97 (td, J = 7.7, 1.5 Hz, 1H), 7.90 (td, J = 7.6, 1.4 Hz, 1H), 5.00 (s, 2H), 3.59 – 3.52 (m, 4H), 3.04 (td, J = 4.0, 2.0 Hz, 2H). **^{13}C NMR** (101 MHz, DMSO-d_6): *δ* (ppm) = 147.9, 135.6, 132.8, 130.9, 129.7, 124.5, 57.6 (2C), 46.1 (2C). **HRMS** (ESI): $C_{10}H_{13}N_2O_6S^+$ [M+H]$^+$: calc. m/z 289.04888, found: 289.04877.

(1-(tert-butylsulfonyl)aziridine-2,3-diyl)dimethanol (S12)

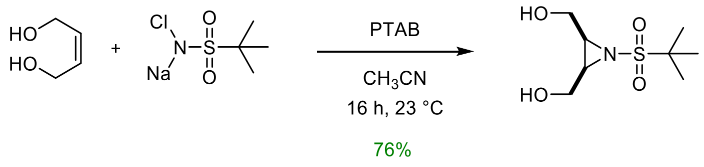

To a solution of *(Z)*-but-2-ene-diol (10 mg, 0.12 mmol, 1.0 equiv.) in anhydrous CH_3CN (1.2 mL, 0.1 M), sodium (tert-butylsulfonyl)chloroamide (50 wt%, 55 mg, 0.14 mmol, 1.2 equiv.) and PTAB (4.5 mg, 12 µmol, 0.1 equiv.) were added and the reaction was stirred at room temperature for 16 h. The reaction mixture was filtered over Celite and the filtrate was concentrated. The residue was purified by column chromatography (SiO_2, 2% MeOH-DCM initially, grading to 5% MeOH), affording aziridine **S12** as a colourless oil (20 mg, 90 µmol, 76%).

Note: Even though this procedure appears to promise a much more efficient entry to **S5** (below), synthesis of BusNClNa was not very reproducible. We thus resorted to the one-pot protocol shown below.

TLC R_f = 0.42 (EtOAc). **^{1}H NMR** (400 MHz, MeOD-d_4): *δ* (ppm) = 3.77 – 3.67 (m, 4H), 2.96 – 2.87 (m, 2H), 1.49 (s, 9H). **^{13}C NMR** (101 MHz, MeOD-d_4): *δ* (ppm) = 60.6, 59.9, 44.7, 24.5. **LRMS** (ESI): $C_{10}H_{13}N_2O_6S^+$ [M+H]$^+$: calc. m/z 224.1, found: 224.1.

***N*-(3-((1,1-dimethylethyl)sulfonamido)-1,4-dihydroxybutan-2-yl)-2-nitrobenzenesulfonamide (S5)**

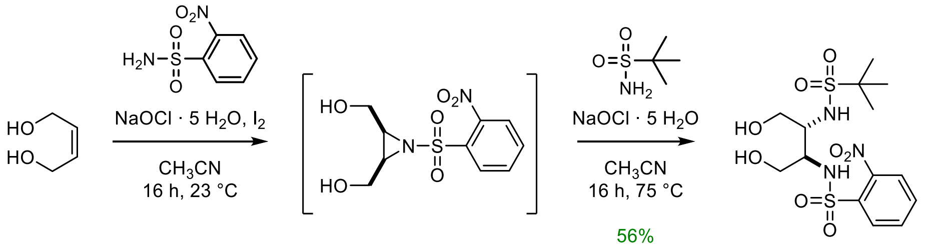

According to Okumura's 1,2-diamination method, *(Z)*-but-2-ene-1,4-diol (2.2 g, 25 mmol, 1.0 equiv.) was dissolved in MeCN (0.13 L, 0.2 M), followed by sequential addition of 2-nitrobenzenesulfonamide (5.0 g, 25 mmol, 1.0 equiv.), sodium hypochlorite pentahydrate (4.1 g, 25 mmol, 1.0 equiv.) and I_2 (0.63 g, 2.5 mmol, 0.1 equiv.). The resulting, pale yellow heterogeneous mixture was stirred at room temperature for 16 h. 2-Methylpropane-2-sulfonamide (3.4 g, 25 mmol, 1.0 equiv.) and sodium hypochlorite pentahydrate (4.1 g, 25 mmol, 1.0 equiv.) were added, the reaction mixture was heated to 75 °C and monitored by LCMS. Full conversion of the aziridine intermediate was achieved within 16 h. The reaction was quenched by adding $Na_2S_2O_3$ (1 M aq., 0.30 L) and the aqueous layer was extracted with CH_2Cl_2 (3×0.30 L). The combined organic extracts were washed with brine (0.20 L), dried over anhydrous $MgSO_4$ and concentrated under reduced pressure. The solid residue was taken up in CH_2Cl_2 (40 mL), vigorously stirred for 10 min, and the suspension was filtered. The filtrate was concentrated *in vacuo* and two additional solid-phase extractions using $CHCl_3$ (2×25 mL) afforded a reasonably pure, pale-yellow crude oil. Purification via a short silica plug (CH_2Cl_2 initially, grading to 5% MeOH) yielded *trans*-diol **S5** as a colourless solid (5.8 g, 14 mmol, 56%).

Notes: (1) During the first step, up to 40% of the intermediary aziridine react further with $NsNH_2$, affording the di-*o*-nosyl 1,2-diamine as undesired side product. (2) Solid-phase extractions allow for removal of residual Ns-aziridine intermediate, $NsNH_2$ and $BusNH_2$ reactants. (3) Synthesis of diamine **S5** from isolated aziridine **S4** proceeds smoothly (71% yield, see synthesis of **3** below), but we deemed the two-step two-purification sequence less efficient than the one-pot procedure shown above.

TLC R_f = 0.48 (EtOAc). **^{1}H NMR** (400 MHz, $CDCl_3$): δ (ppm) = 8.19 – 8.13 (m, 1H), 7.91 – 7.84 (m, 1H), 7.79 – 7.72 (m, 2H), 6.20 (d, J = 8.4 Hz, 1H), 5.06 (d, J = 9.4 Hz, 1H), 3.93 – 3.67 (m, 4H), 3.56 (hept, J = 6.6, 5.9 Hz, 2H), 3.42 (t, J = 6.0 Hz, 1H), 3.36 (t, J = 6.3, 5.4 Hz, 1H), 1.40 (s, 9H). **^{13}C NMR** (101 MHz, $CDCl_3$): δ (ppm) = 147.8, 134.2, 133.5, 133.4, 131.2, 125.8, 61.4, 60.7, 60.7, 55.9, 55.8, 24.4. **HRMS** (ESI): $C_{14}H_{22}N_3O_8S_2^-$ $[M-H]^-$: calc. m/z 424.08538, found: 424.08446.

2-((1,1-dimethylethyl)sulfonamido)-3-((2-nitrophenyl)sulfonamido)butane-1,4-diyl dimethanesulfonate (S6)

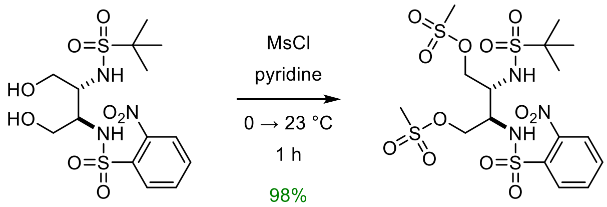

Diol **S5** (5.5 g, 13 mmol, 1.0 equiv.) was dissolved in pyridine (40 mL, 0.3 M), cooled to 0 °C and MsCl (2.2 mL, 28 mmol, 2.2 equiv.) was added carefully. A colour change from colourless to bright yellow occurred. The reaction was allowed to warm to room temperature and stirred for 1 h, upon which TLC control indicated full conversion of the starting material. The reaction mixture was diluted with CH_2Cl_2 (0.30 L) and transferred into a separatory funnel. The organic layer was washed with HCl (1 M, 3×0.15 L) and brine, dried over $MgSO_4$, filtered, and concentrated *in vacuo*, affording **S6** as a colourless foam (7.4 g, 13 mmol, 98%) without requiring further purification.

TLC R_f = 0.52 (hexanes/EtOAc 1:2). **^{1}H NMR** (400 MHz, $CDCl_3$): δ (ppm) = 8.13 – 8.02 (m, 1H), 7.89 – 7.80 (m, 1H), 7.77 – 7.66 (m, 2H), 6.31 (s, 1H), 5.09 (d, J = 9.8 Hz, 1H), 4.41 – 4.31 (m, 2H), 4.18 (d, J = 5.9 Hz, 2H), 4.02 (s, 1H), 3.89 (dddd, J = 10.1, 6.4, 5.1, 3.6 Hz, 1H), 3.09 (s, 3H), 2.82 (s, 3H), 1.36 (s, 9H). **^{13}C NMR** (101 MHz, $CDCl_3$): δ (ppm) = 147.7, 134.4, 133.7, 133.6, 131.1, 125.9, 68.4, 68.0, 61.0, 54.6, 54.1, 37.7, 37.3, 24.4. **HRMS** (ESI): $C_{16}H_{31}N_4O_{12}S_4^+$ $[M+NH_4]^+$: calc. m/z 599.08158, found: 599.08116.

1-(tert-butylsulfonyl)-1'-((2-nitrophenyl)sulfonyl)-2,2'-biaziridine (S7)

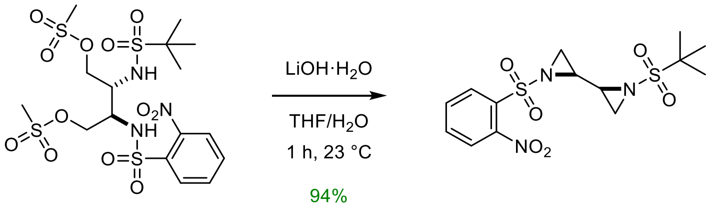

To a vigorously stirred solution of dimesylate **S6** (7.4 g, 13 mmol, 1.0 equiv.) in THF/H_2O (2:1, 80+40 mL, 0.1 M), LiOH monohydrate (1.2 g, 29 mmol, 2.3 equiv.) was added in one batch at room temperature, resulting in a colourless suspension which gradually changed its colour to light orange. The reaction was closely monitored *via* TLC, and once completion was reached within 1 h, diluted with EtOAc (0.30 L) and transferred into a separatory funnel. The organic layer was washed with H_2O (0.15 L) and NaCl (sat. aq., 0.15 L), dried over $MgSO_4$, filtered and concentrated, affording biaziridine **S7** as a light orange foam (4.8 g, 12 mmol, 94%).

Notes: (1) At <1 g scale, the reaction usually reaches completion within 10-20 min. (2) Initial attempts to isolate **S7** *via* chromatographic purification on (deactivated) SiO_2, resulted in severely compromised yields. (3) Monitoring the reaction via LCMS (sample submitted in MeOH) led to the detection of significant amounts of a single MeOH-addition Spezi – a testament to the exceptional reactivity of the (nosyl)aziridine.

TLC R_f = 0.67 (hexanes/EtOAc 1:2). **^{1}H NMR** (400 MHz, $CDCl_3$): δ (ppm) = 8.17 (dd, J = 7.5, 1.9 Hz, 1H), 7.86 – 7.72 (m, 3H), 3.19 (dt, J = 7.1, 4.6 Hz, 1H), 2.93 – 2.85 (m, 2H), 2.62 (d, J = 6.9 Hz, 1H), 2.49 (d, J = 4.5 Hz, 1H), 2.28 (d, J = 4.2 Hz, 1H), 1.46 (s, 9H). **^{13}C NMR** (101 MHz, $CDCl_3$): δ (ppm) = 148.7, 134.9, 132.4, 131.5, 131.4, 124.6, 60.0, 39.1, 36.1, 34.1, 31.9, 24.2. **HRMS** (ESI): $C_{14}H_{19}N_3NaO_6S_2^+$ [M+Na]$^+$: calc. m/z 412.06075, found: 412.06062.

N-(1-(1-(tert-butylsulfonyl)aziridin-2-yl)-2-selenocyanatoethyl)-2-nitrobenzenesulfonamide (S8)

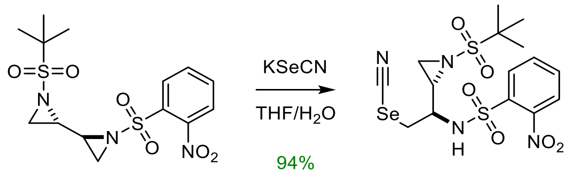

To a solution of bis-aziridine **S7** (20 mg, 51 µmol, 1.0 equiv.) in THF (0.50 mL, 0.1 M), KSeCN (7.4 mg, 51 µmol, 1.0 equiv.) in H_2O (50 µL) was added at room temperature. A colour change to orange occurred within a few minutes, and full conversion to the selenocyanate intermediate was monitored via TLC. The reaction mixture was diluted with EtOAc (2 mL) and transferred into a separatory funnel. The organic layer was washed with H_2O (2 mL) and brine (1 mL), dried over $MgSO_4$ and filtered. The solution was concentrated, affording selenocyanate **S8** as a light yellow solid (24 mg, 48 µmol, 94%).

TLC R_f = 0.37 (hexanes/EtOAc 1:1). **^{1}H NMR** (400 MHz, $CDCl_3$): 8.22 – 8.18 (m, 1H), 7.94 – 7.91 (m, 1H), 7.81 (ddd, J = 7.5, 4.9, 1.8 Hz, 2H), 5.51 (s, 1H), 4.30 (td, J = 6.6, 2.4 Hz, 1H), 3.17 (dd, J = 6.7, 3.1 Hz, 2H), 3.11 (ddd, J = 6.8, 4.4, 2.4 Hz, 1H), 2.67 (d, J = 6.9 Hz, 1H), 2.58 (d, J = 4.3 Hz, 1H), 1.50 (s, 9H). **^{13}C NMR** (101 MHz, $CDCl_3$): δ (ppm) = 147.8, 134.4, 134.0, 133.8, 130.7, 125.9, 100.9, 60.3, 52.2, 35.8, 34.5, 32.7, 24.3. **HRMS** (ESI): $C_{15}H_{19}N_4O_6S_2Se^-$ [M-H]$^-$ calc. m/z 494.99167, found: 494.99138.

N-(5-((1,1-dimethylethyl)sulfonamido)-1,2-thiaselenan-4-yl)-2-nitrobenzenesulfonamide (S9)

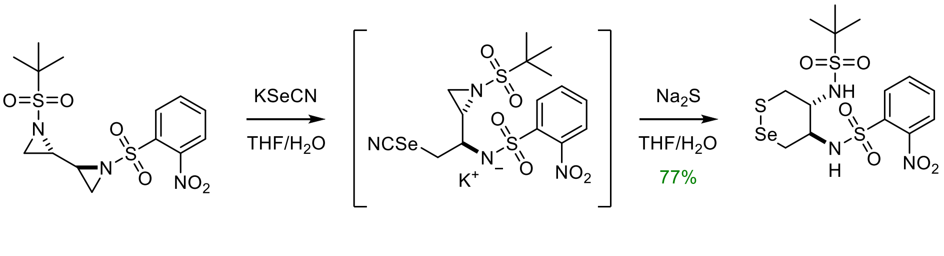

To a solution of bis-aziridine **S7** (10 mg, 26 µmol, 1.0 equiv.) in THF/H_2O (1:1, 0.50 mL, 50 mM), KSeCN (3.7 mg, 26 µmol, 1.0 equiv.) in H_2O (50 µL) was added at room temperature. A colour change to orange occurred within a few minutes, and

full conversion to the selenocyanate intermediate was monitored via TLC (R_f = 0.37 (hexanes/EtOAc 1:1). Next, a solution of Na_2S (7.4 mg, 31 µmol, 1.2 equiv.) in H_2O (50 µL) was added. An immediate colour change to bright yellow occurred and the reaction was stirred for 10 min. Full conversion of the intermediate was again monitored via TLC. The reaction mixture was diluted with EtOAc (5 mL) and transferred into a separatory funnel. The organic layer was washed with H_2O (5 mL) and brine (5 mL), dried over $MgSO_4$, filtered and concentrated. The yellow crude solids were purified by chromatography (SiO_2, hexanes/EtOAc 2:1 → 3:2 → 1:1, 1% AcOH) yielding 1,2-thiaselenane **S9** as a pale-yellow foam (10 mg, 20 µmol, 77%).

Notes: (1) A recurring side product of this reaction is the tetrahydroselenophene-3,4-diamine, its formation is discussed in chapter 1. Chromatographic separation of this species from a tetrahydroselenophene impurity is very challenging and, if probe assembly is desired later, can be postponed until prepHPLC purification of final compounds. (2) ^{13}C signals of the 1,2-thiaselenane core are exceptionally broad and don't even show in the HSQC at reasonable measurement times. Unequivocal assignment of the carbons is reported for the ensuing synthetic intermediate **7T**.

TLC R_f = 0.46 (hexanes/EtOAc 1:1). **^{1}H NMR** (400 MHz, $CDCl_3$): δ (ppm) = 8.23 – 8.17 (m, 1H), 7.89 – 7.85 (m, 1H), 7.82 – 7.73 (m, 2H), 6.35 (br s, 1H), 5.00 (br s, 1H), 4.03 – 3.89 (m, 1H), 3.86 – 3.62 (m, 3H), 2.75 (d, J = 14.3 Hz, 1H), 2.47 (br s, 1H), 1.40 (s, 9H). **^{13}C NMR** (101 MHz, $CDCl_3$): δ (ppm) = 148.1, 134.2, 133.6, 133.4, 131.5, 125.6, 60.6, 24.4. **HRMS** (ESI): $C_{14}H_{21}N_3NaO_6S_3Se^+$ $[M+Na]^+$: calc. m/z 525.96499, found: 525.96458.

4-(tert-butylsulfonyl)-1-((2-nitrophenyl)sulfonyl)octahydro-[1,2]thiaselenino[4,5-b]pyrazine (7T)

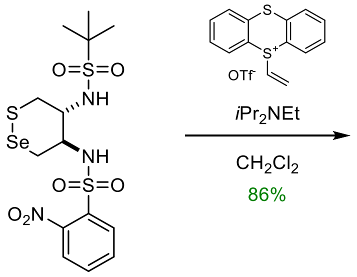

1,2-Thiaselenane **S9** (0.24 g, 0.48 mmol, 1.0 equiv.) was dissolved in CH_2Cl_2 (5.0 mL, 0.1 M) and *i*-Pr_2NEt (0.41 mL, 2.4 mmol, 5.0 equiv.) was added. The bright yellow solution was stirred for 5 min at room temperature, upon which VTT (0.21 g, 0.53 mmol, 1.1 equiv.) in CH_2Cl_2 (1.0 mL) was added in a dropwise manner. The reaction mixture's yellow colour intensified, and reaction progress was monitored via LCMS. After 4 h, the reaction mixture was transferred into a separatory funnel and the organic layer was extracted twice with NH_4Cl (20 mL), dried over $MgSO_4$, filtered and concentrated. The pale-yellow crude solids were purified by chromatography (hexanes/EtOAc 3:1→2:1→3:2), affording piperazine **7T** as a pale-yellow foam (0.22 g, 0.41 mmol, 86%).

TLC R_f = 0.48 (hexanes/EtOAc 1:1). **^{1}H NMR** (400 MHz, $CDCl_3$): δ (ppm) = 8.14 – 8.08 (m, 1H), 7.74 – 7.64 (m, 3H), 4.28 (td, J = 10.4, 10.0, 2.6 Hz, 1H), 3.94 – 3.58 (m, 7H), 3.47 – 3.39 (m, 1H), 3.14 (dd, J = 12.1, 2.7 Hz, 1H), 1.32 (s, 9H). **^{13}C NMR** (101 MHz, $CDCl_3$): δ (ppm) = 147.9, 135.1, 133.8, 132.3, 130.9, 124.5, 64.8, 63.3, 61.9, 46.4, 42.8, 40.0, 30.9, 24.7. **HRMS** (ESI): $C_{16}H_{23}N_3O_6S_3Se^+$ $[M+Na]^+$: calc. m/z 551.98064, found: 551.98012.

4-(tert-butylsulfonyl)octahydro-[1,2]thiaselenino[4,5-b]pyrazine (S13)

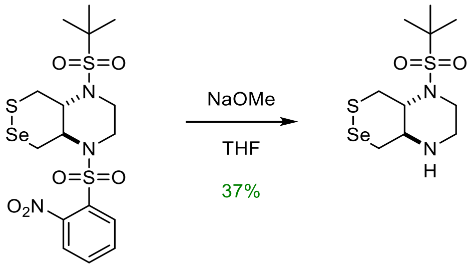

Orthogonally protected piperazine **7T** (0.20 g, 0.38 mmol, 1.0 equiv.) was dissolved in anhydrous THF (4.0 mL, 0.1 M) and cooled to 0 °C. To the pale-yellow solution, NaOMe (5.4 M in anhydrous MeOH, 32 µL, 0.17 mmol, 0.45 equiv.) was added in one batch, an immediate colour change to orange occurred. After 10 min at retained temperature, another batch of NaOMe (5.4 M in anhydrous MeOH, 32 µL, 0.17 mmol, 0.45 equiv.) was added and the reaction was stirred until completion was indicated by TLC after 1 h. The reaction mixture was concentrated, and the orange crude oil purified by

chromatography (SiO_2, CH_2Cl_2 initially, grading to 2% MeOH) to afford busyl-protected piperazine **S13** as a bright yellow foam (49 mg, 0.14 mmol, 37%).

Note: The deprotection required quite extensive screening. Among other variables, performing the reaction in pure MeOH, at ambient temperature, or using older batches of NaOMe all gave complex reaction mixtures. Precise, batch-wise addition of a sub-stoichiometric amount of NaOMe appeared essential to reliably produce satisfactory quantities of **S13**. An analytically pure sample of **S13** was obtained via preparative HPLC (reverse phase, 10% CH_3CN in H_2O initially, grading to 100% CH_3CN, 0.1% formic acid).

TLC R_f = 0.56 (CH_2Cl_2/MeOH 9:1). **^{1}H NMR** (400 MHz, CD_3CN): *δ* (ppm) = 3.67 (q, J = 7.9 Hz, 1H), 3.44 (ddd, J = 14.4, 8.7, 5.7 Hz, 1H), 3.39 – 3.28 (m, 3H), 3.19 – 3.04 (m, 3H), 3.02 – 2.94 (m, 2H), 1.36 (s, 9H). *HSQC data can be found in the appendix, although not all carbons of **S13** could be reliably assigned.* **HRMS** (ESI): $C_{10}H_{21}N_2O_2S_2Se^+$ [M+H]$^+$: calc. m/z 345.02042, found: 345.02032.

(1-((2-nitrophenyl)sulfonyl)aziridine-2,3-diyl)dimethanol (2a)

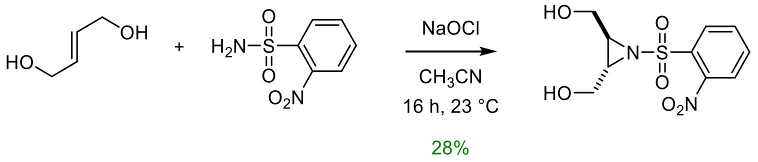

(Z)-but-2-ene-1,4-diol (0.50 g, 5.7 mmol, 1.0 equiv.) was dissolved in MeCN (25 mL, 0.2 M), followed by sequential addition of 2-nitrobenzenesulfonamide (1.1 g, 5.7 mmol, 1.0 equiv.), sodium hypochlorite pentahydrate (1.2 g, 7.4 mmol, 1.3 equiv.) and I_2 (0.14 g, 0.57 mmol, 0.1 equiv.). The resulting, pale yellow heterogeneous mixture was stirred at room temperature for 16 h. The reaction was quenched by adding a 1:1 mixture of water (25 mL) and $Na_2S_2O_3$ (sat. aq., 25 mL) and the aqueous layer was extracted with EtOAc (80 mL). The combined organic extracts were washed with brine (25 mL), dried over anhydrous $MgSO_4$ and concentrated under reduced pressure. The colourless residue was purified via chromatography (SiO_2, EtOAc) affording aziridine **2a** as a colourless solid (0.46 g, 1.6 mmol, 28%).

Notes: (1) TLC analysis should always be performed using both UV and $KMnO_4$ stain, since a non-UV active species elutes right after the aziridine (i.e. slightly lower R_f) (2) As for other diols reported herein, mixtures of DCM/MeOH as eluent for chromatography will likely result in higher isolated yields (3) **2a** can also be purified by recrystallisation from EtOAc. (4) **2a** appeared to decompose when left to sit for > days in a solution of DMSO-*d_6*.

TLC R_f = 0.30 (EtOAc). **^{1}H NMR** (400 MHz, DMSO-*d_6*): *δ* (ppm) = 8.20 (dd, J = 7.3, 1.8 Hz, 1H), 8.03 (dd, J = 7.9, 1.6 Hz, 1H), 7.95 – 7.87 (m, 2H), 5.07 (t, J = 6.0, 5.5 Hz, 2H), 3.76 (dddd, J = 12.1, 6.2, 3.6, 1.1 Hz, 2H), 3.68 – 3.59 (m, 2H), 3.08 (p, J = 4.2, 3.7 Hz, 2H). **^{13}C NMR** (101 MHz, DMSO-*d_6*): *δ* (ppm) = 147.4, 135.1, 132.9, 132.5, 130.2, 124.5, 58.9, 58.8, 49.3, 49.2. **LRMS** (ESI): $C_{10}H_{13}N_2O_6S^+$ [M+H]$^+$: calc. m/z 289.0, found: 289.1.

(1-(tert-butylsulfonyl)aziridine-2,3-diyl)dimethanol (2b)

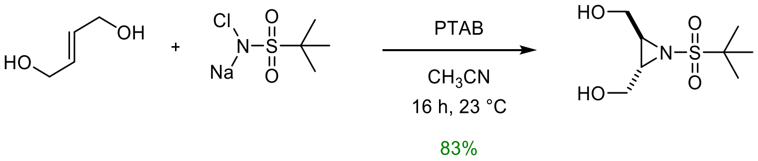

To a solution of *(E)*-but-2-ene-diol (50 mg, 0.57 mmol, 1.0 equiv.) in anhydrous CH_3CN (5.5 mL, 0.1 M), sodium (tert-butylsulfonyl)chloroamide (50 wt%, 0.28 g, 1.4 mmol, 1.2 equiv.) and PTAB (21 mg, 57 µmol, 0.1 equiv.) were added and the reaction was stirred at room temperature for 16 h. The reaction mixture was filtered over Celite and the filtrate was concentrated. The residue was purified by column chromatography (SiO_2, 2% MeOH in CH_2Cl_2 initially, grading to 5% MeOH), affording aziridine **2b** as a colourless oil (0.11 g, 0.47 mmol, 83%).

Notes: (1) "wet" BusNClNa, i.e. reagent that has not been dried on high vacuum at 80 °C, did only result in 5% conversion to target (determined by NMR). (2) Even though this procedure appears to promise a much more efficient entry to **3**, synthesis of BusNClNa was not very reproducible. We thus resorted to the one-pot protocol shown below.

TLC R_f = 0.45 (EtOAc). **^{1}H NMR** (400 MHz, MeOD-*d_4*): *δ* (ppm) = 3.87 – 3.78 (m, 4H), 2.93 – 2.87 (m, 2H), 1.47 (s, 9H). **^{13}C NMR** (101 MHz, MeOD-*d_4*): *δ* (ppm) = 61.4, 47.8, 24.4. **HRMS** (ESI): $C_{10}H_{13}N_2O_6S^+$ [M+H]$^+$: calc. m/z 246.07705, found: 246.07720.

***N*-3-((1,1-dimethylethyl)sulfonamido)-1,4-dihydroxybutan-2-yl)-2-nitrobenzenesulfonamide (3)**

Two-step procedure:

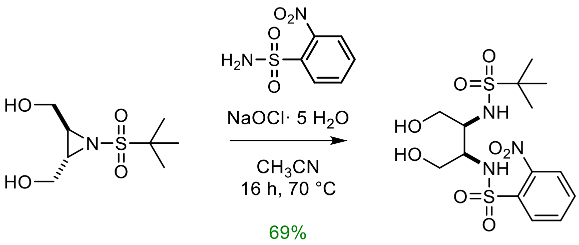

To a solution of busyl-aziridine **2b** (10 mg, 45 µmol, 1.0 equiv.) in CH_3CN (0.5 mL, 0.1 M), 2-nitrobenzenesulfonamide (10 mg, 50 µmol, 1.1 equiv.), and sodium hypochlorite pentahydrate (8.1 mg, 50 µmol, 1.1 equiv.) were added sequentially and the colourless suspension was heated to 70 °C. Reaction progress was monitored by TLC, and full conversion was reached within 16 h. $Na_2S_2O_3$ (14 mg, 90 µmol, 2.0 equiv.) was added, and the mixture was vigorously stirred for 30 min, then filtered over Celite. The filtrate was concentrated *in vacuo*, then purified by chromatography (SiO_2, CH_2Cl_2 initially, grading to 5% MeOH) to afford diamine **3** as a colourless foam (13 mg, 31 µmol, 69%).

One-pot procedure:

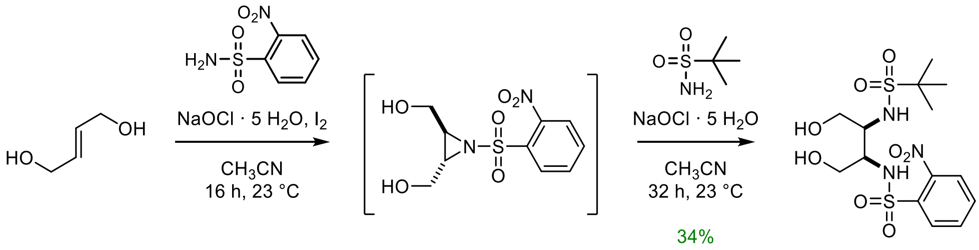

According to Okumura's 1,2-diamination method, *(E)*-but-2-ene-1,4-diol (2.2 g, 25 mmol, 1.0 equiv.) was dissolved in MeCN (0.13 L, 0.2 M), followed by sequential addition of 2-nitrobenzenesulfonamide (5.0 g, 25 mmol, 1.0 equiv.), sodium hypochlorite pentahydrate (4.1 g, 25 mmol, 1.0 equiv.) and I_2 (0.63 g, 2.5 mmol, 0.1 equiv.). The resulting, pale yellow heterogeneous mixture was stirred at room temperature for 16 h. 2-Methylpropane-2-sulfonamide (3.4 g, 25 mmol, 1.0 equiv.) and sodium hypochlorite pentahydrate (4.1 g, 25 mmol, 1.0 equiv.) were added, the reaction mixture was kept stirring at room temperature and monitored by LCMS. Full conversion of the aziridine intermediate was achieved within 32 h. The reaction was quenched by adding a 1:1 mixture of water (0.15 L) and $Na_2S_2O_3$ (sat. aq., 0.15 L) and the aqueous layer was extracted with CH_2Cl_2 (3×0.30 L). The combined organic extracts were washed with brine (0.20 L), dried over anhydrous $MgSO_4$ and concentrated under reduced pressure. The solid residue was taken up in CH_2Cl_2 (40 mL), vigorously stirred for 10 min, and the suspension was filtered. The filtrate was concentrated *in vacuo* and two additional solid-phase extractions using $CHCl_3$ (2×25 mL) afforded a reasonably pure, pale-yellow crude oil. Purification via a short silica plug (CH_2Cl_2 initially, grading to 2% MeOH) yielded *cis*-diol **3** as a colourless solid (3.6 g, 8.5 mmol, 34%).

Notes: (1) During the first step, up to 40% of the intermediary aziridine react further with $NsNH_2$, affording the di-*o*-nosyl 1,2-diamine as undesired side product. (2) Solid-phase extractions allow for removal of residual Ns-aziridine intermediate, $NsNH_2$ and $BusNH_2$ reactants. (3) During chromatographic purification, a slow gradient from 1 to 2% MeOH is essential for good separation. A sharp, bright yellow band elutes right before **3** and can be observed by eye.

TLC R_f = 0.36 (CH_2Cl_2/MeOH 95:5). **^{1}H NMR** (400 MHz, $CDCl_3$): δ (ppm) = 8.09 (dd, J = 5.9, 3.4 Hz, 1H), 7.96 (dd, J = 5.9, 3.3 Hz, 1H), 7.86 – 7.80 (m, 2H), 6.39 (d, J = 8.2 Hz, 1H), 4.64 (t, J = 5.0 Hz, 1H), 3.60 – 3.49 (m, 4H), 3.44 (t, J = 4.8 Hz, 2H), 1.27 (s, 9H). **^{13}C NMR** (101 MHz, $CDCl_3$): δ (ppm) = 147.4, 133.9, 133.8, 132.6, 130.1, 124.4, 61.5, 60.5, 59.0, 57.5, 56.5, 23.9. **HRMS** (ESI): $C_{14}H_{22}N_3O_8S_2^-$ [M-H]$^-$: calc. m/z 424.08538, found: 424.08479.

2-((1,1-dimethylethyl)sulfonamido)-3-((2-nitrophenyl)sulfonamido)butane-1,4-diyl dimethanesulfonate (4)

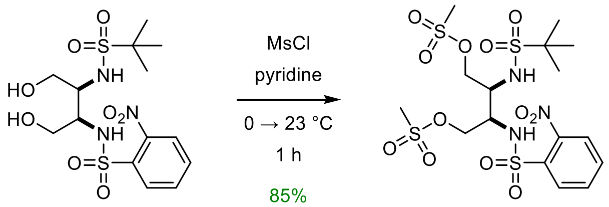

Diol **3** (5.7 g, 13 mmol, 1.0 equiv.) was dissolved in pyridine (50 mL, 0.3 M), cooled to 0 °C and MsCl (2.3 mL, 30 mmol, 2.2 equiv.) was added carefully. A colour change from colourless to bright yellow occurred. The reaction was allowed to warm to room temperature and stirred for 1 h, upon which TLC control indicated full conversion of the starting material. The reaction mixture was diluted with CH_2Cl_2 (0.30 L) and transferred into a separatory funnel. The organic layer was washed with HCl (1 M, 3×0.25 L) and brine, dried over $MgSO_4$, filtered, and concentrated *in vacuo*, affording **4** as a colourless foam (6.6 g, 11 mmol, 85%) without requiring further purification.

Note: An analytically pure sample of **4** was obtained *via* chromatography (reverse phase, 10% CH_3CN in H_2O initially, grading to 90% CH_3CN, 0.1% formic acid).

TLC R_f = 0.71 (EtOAc). **^{1}H NMR** (400 MHz, $CDCl_3$): δ (ppm) = 8.48 (d, J = 8.7 Hz, 1H), 8.07 – 7.96 (m, 2H), 7.92 – 7.83 (m, 2H), 7.28 (d, J = 8.9 Hz, 1H), 4.31 (dd, J = 10.4, 3.3 Hz, 2H), 4.20 (dt, J = 10.6, 5.6 Hz, 2H), 3.94 – 3.75 (m, 2H), 3.13 (s, 3H), 2.92 (s, 3H), 1.29 (s, 9H). **^{13}C NMR** (101 MHz, $CDCl_3$): δ (ppm) = 147.1, 134.4, 133.4, 133.1, 130.0, 124.8, 68.9, 68.4, 59.1, 53.4, 53.0, 36.7, 36.4, 23.7. **HRMS** (ESI): $C_{16}H_{27}N_3O_{12}NaS_4^+$ $[M+Na]^+$: calc. m/z 604.03698, found: 604.03639

1-(tert-butylsulfonyl)-1'-((2-nitrophenyl)sulfonyl)-2,2'-biaziridine (5)

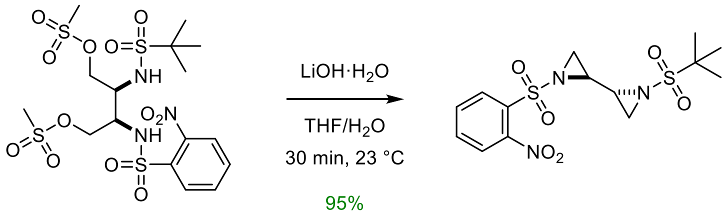

To a vigorously stirred solution of dimesylate **4** (0.58 g, 1.0 mmol, 1.0 equiv.) in THF/H_2O (7+3 mL, 0.1 M), LiOH monohydrate (96 mg, 2.3 mmol, 2.3 equiv.) was added in one batch at room temperature, resulting in a colourless suspension which gradually changed its colour to light orange. The reaction was closely monitored *via* TLC, and once completion was reached within 1 h, diluted with EtOAc (20 mL) and transferred into a separatory funnel. The organic layer was washed with H_2O (10 mL) and NaCl (sat. aq., 10 mL), dried over $MgSO_4$, filtered and concentrated, affording biaziridine **5** as a colourless foam (0.37 g, 0.95 mmol, 95%).

Notes: (1) Attempts to isolate **5** *via* chromatography on (deactivated) SiO_2, results in severely compromised yields, likely due to decomposition. (2) Monitoring the reaction via LCMS (sample submitted in MeOH) led to the detection of significant amounts of a single MeOH-addition species – a testament to the exceptional reactivity of the (nosyl)aziridine.

TLC R_f = 0.40 (hexanes/EtOAc 1:1). **^{1}H NMR** (400 MHz, $CDCl_3$): δ (ppm) = 8.24 – 8.19 (m, 1H), 7.84 – 7.74 (m, 3H), 3.26 (ddd, J = 7.0, 4.6, 3.6 Hz, 1H), 2.98 (dt, J = 6.9, 4.0 Hz, 1H), 2.92 (d, J = 7.1, 0.8 Hz, 1H), 2.63 (d, J = 6.9 Hz, 1H), 2.46 (d, 1H), 2.26 (d, J = 4.3 Hz, 1H), 1.39 (s, 9H). **^{13}C NMR** (101 MHz, $CDCl_3$): δ (ppm) = 148.7, 134.1, 132.5, 131.8, 131.5, 124.6, 60.0, 40.4, 36.9, 32.5, 30.3, 24.1. **HRMS** (ESI): $C_{14}H_{19}N_3NaO_6S_2^+$ $[M+Na]^+$: calc. m/z 412.06075, found: 412.06064.

***N*-(5-((1,1-dimethylethyl)sulfonamido)-1,2-thiaselenan-4-yl)-2-nitrobenzenesulfonamide (6)**

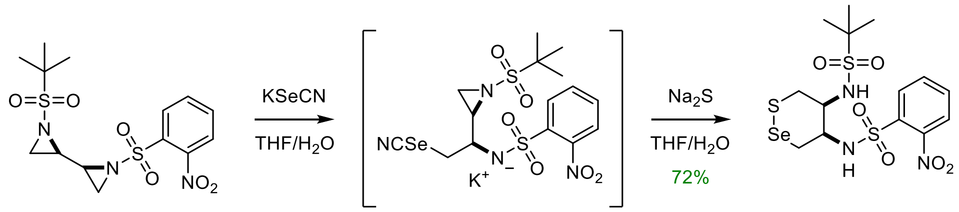

To a suspension of biaziridine **5** (1.7 g, 4.4 mmol, 1.0 equiv.) in THF/H_2O (1:1, 60 mL, 75 mM), KSeCN (0.63 g, 4.4 mmol, 1.0 equiv.) in H_2O (5 mL) was added at room temperature. A colour change to orange occurred within a few minutes, and full conversion to the selenocyanate intermediate was monitored via TLC (R_f = 0.38 (hexanes/EtOAc 1:1). Next, a solution of Na_2S (1.2 g, 4.8 mmol, 1.1 mmol) in H_2O (5 mL) was added. An immediate colour change to bright yellow occurred and the reaction was stirred for 10 min. Full conversion of the intermediate was again monitored via TLC. The reaction mixture was diluted with EtOAc (0.15 L) and transferred into a separatory funnel. The organic layer was washed with H_2O (0.10 L) and brine (0.10 L), dried over $MgSO_4$, filtered and concentrated. The yellow crude solids were purified by chromatography (SiO_2, hexanes/EtOAc 2:1 → 3:2 → 1:1, 1% AcOH) yielding 1,2-thiaselenane **6** as a pale-yellow foam (1.6 g, 3.1 mmol, 72%).

Notes: (1) A recurring side product of this reaction is the tetrahydroselenophene-3,4-diamine, its formation is discussed in chapter 1. Chromatographic separation of this species from a tetrahydroselenophene impurity is very challenging and, if probe assembly is desired, can be postponed until prepHPLC purification of final probes. (2) As for the related trans compound, ^{13}C signals of the 1,2-thiaselenane core of **6** are exceptionally broad and don't even show in the HSQC at reasonable measurement times. Unequivocal carbon assignment is reported for the ensuing synthetic intermediate **7**.

TLC R_f = 0.60 (hexanes/EtOAc 1:1, 1% AcOH). **^{1}H NMR** (400 MHz, $CDCl_3$): δ (ppm) = 8.21 – 8.15 (m, 1H), 7.91 – 7.85 (m, 1H), 7.82 – 7.75 (m, 2H), 6.43 (d, J = 9.1 Hz, 1H), 4.77 (d, J = 9.8 Hz, 1H), 3.97 (ddt, J = 9.2, 6.6, 2.6 Hz, 1H), 3.70 (d, J = 11.6 Hz, 1H), 3.42 – 2.97 (m, 3H), 2.73 (br s, 1H), 1.42 (s, 9H). **^{13}C NMR** (101 MHz, $CDCl_3$): δ (ppm) = 148.1, 134.5, 133.3 (2C), 131.1, 125.8, 60.4, 24.3. **HRMS** (ESI): $C_{14}H_{21}N_3NaO_6S_3Se^+$ $[M+Na]^+$: calc. m/z 525.96499, found: 525.96462.

4-(tert-butylsulfonyl)-1-((2-nitrophenyl)sulfonyl)octahydro-[1,2]thiaselenino[4,5-b]pyrazine (7)

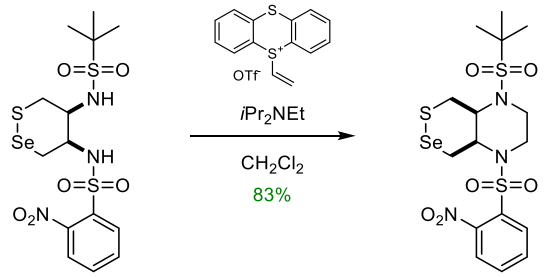

1,2-Thiaselenane **6** (0.30 g, 0.60 mmol, 1.0 equiv.) was dissolved in CH_2Cl_2 (6 mL, 0.1 M) and *i*-Pr_2NEt (0.51 mL, 3.0 mmol, 5.0 equiv.) was added. The bright yellow solution was stirred for 5 min at room temperature, upon which vinyl thianthrenium triflate (0.26 g, 0.66 mmol, 1.1 equiv.) in CH_2Cl_2 (1 mL) was added in a dropwise manner. The reaction mixture's colour intensified, and reaction progress was monitored via LCMS. After 4 h, the reaction mixture was transferred into a separatory funnel and the organic layer was extracted with NH_4Cl (sat. aq., 2 × 10 mL), dried over $MgSO_4$, filtered and concentrated. The pale-yellow crude solids were purified by chromatography (SiO_2, hexanes/EtOAc 3:1 → 2:1 → 3:2) yielding piperazine **7** as a pale-yellow foam (0.26 g, 0.50 mmol, 83%).

Note: Single crystals of **7** were grown via liquid diffusion: A small sample (10 mg) was dissolved in $CHCl_3$ (0.2 mL) and placed in an NMR tube. Hexanes (0.8 mL) was carefully layered down the side of the tube and the biphasic sample was left to sit for 36 h, upon which pale yellow single crystals had formed.

TLC R_f = 0.45 (hexanes/EtOAc 1:1). **^{1}H NMR** (400 MHz, $CDCl_3$): δ (ppm) = 8.06 (d, J = 7.4, 1H), 7.81 – 7.70 (m, 3H), 4.29 – 4.20 (m, 1H), 4.12 – 4.01 (m, 1H), 3.89 – 3.81 (m, 2H), 3.79 – 3.68 (m, 2H), 3.57 – 3.42 (m, 2H), 3.37 (dd, J = 13.9, 2.7 Hz, 1H), 3.09 (dd, J = 14.1, 2.9 Hz, 1H), 1.34 (s, 9H). **^{13}C NMR** (101 MHz, $CDCl_3$): δ (ppm) = 148.5, 134.6, 133.0, 132.4, 130.9, 125.2, 62.0, 57.6, 57.1, 46.1, 43.4, 35.8, 31.9, 24.5. **HRMS** (ESI): $C_{16}H_{23}N_3O_6S_3Se^+$ $[M+Na]^+$: calc. m/z 551.98064, found: 551.98025.

4-(tert-butylsulfonyl)octahydro-[1,2]thiaselenino[4,5-b]pyrazine (8)

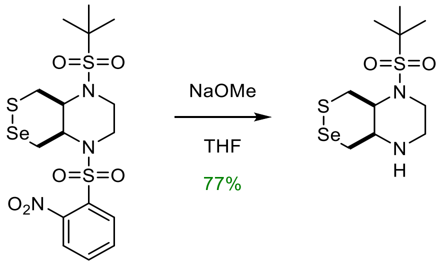

Orthogonally protected piperazine **7** (50 mg, 95 µmol, 1.0 equiv.) was dissolved in anhydrous THF (1.0 mL, 0.1 M) and cooled to 0 °C. To the pale-yellow solution, NaOMe (5.4 M in anhydrous MeOH, 12 µL, 53 µmol, 0.55 equiv.) was added in one batch, an immediate colour change to orange occurred. After 10 min at retained temperature, another batch of NaOMe (5.4 M in anhydrous MeOH, 10 µL, 44 µmol, 0.45 equiv.) was added and the reaction was stirred until completion was indicated by TLC after 1 h. The reaction mixture was concentrated, and the orange crude oil purified by chromatography (SiO_2, CH_2Cl_2 initially, slowly grading to 2% MeOH) to afford busyl-protected piperazine **8** as a bright yellow foam (25 mg, 73 µmol, 77%).

Note: (1) The deprotection required quite extensive screening. Among other variables, performing the reaction in pure MeOH, at ambient temperature, or using older batches of NaOMe all gave complex reaction mixtures. Precise, batch-wise addition of a (sub-)stoichiometric amount of NaOMe appeared essential to reliably produce satisfactory quantities of **8**. (2) During chromatography, all bright yellow fractions are to be discarded. The first yellow band elutes with 0-1% MeOH; separation of the second fraction requires patient elution with 1.5% MeOH. From here, the CH_2Cl_2/MeOH solvent ratio should not be changed until the second yellow band is collected. Otherwise, **8** will co-elute with this side product.

TLC R_f = 0.38 (CH_2Cl_2/MeOH 95:5). **^{1}H NMR** (400 MHz, $CDCl_3$): *δ* (ppm) = 3.81 – 3.72 (m, 2H), 3.67 (dd, J = 13.0, 2.5 Hz, 1H), 3.35 (dd, J = 14.4, 4.7 Hz, 1H), 3.25 (dd, J = 4.3, 2.5 Hz, 1H), 3.15 (m, 2H), 3.10 – 3.02 (m, 2H), 2.84 (d, J = 10.6 Hz, 1H), 1.35 (s, 9H). **^{13}C NMR** (101 MHz, $CDCl_3$): *δ* (ppm) = 61.5, 56.8, 53.6, 45.9, 43.0, 35.5, 30.5, 24.5. **HRMS** (ESI): $C_{10}H_{21}N_2O_2S_2Se^+$ $[M+H]^+$: calc. m/z 345.02042, found: 345.02034.

4-(tert-butylsulfonyl)octahydro-[1,2]thiaselenino[4,5-b]pyrazine (S14)

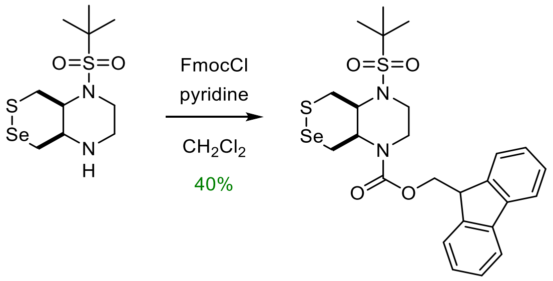

Busyl-piperazine **8** (30 mg, 87 µmol, 1.0 equiv.) was dissolved in anhydrous CH_2Cl_2 (1.0 mL, 0.1 M) and the yellow solution was cooled to 0 °C. Pyridine (18 µL, 0.22 mmol, 2.5 equiv.) and FmocCl (25 mg, 96 µmol, 1.1 equiv.) were added sequentially, and the reaction was allowed to warm to room temperature and stirred overnight. The reaction was concentrated, and the crude oil was purified by chromatography (SiO_2, hexanes/EtOAc 5:1 initially, grading to hexanes/EtOAc 3:1), affording orthogonally protected **S14** (20 mg, 35 µmol, 40%) as a pale-yellow solid.

Note: **S14** ionised very poorly under ESI conditions; reaction progress is best monitored by TLC.

TLC R_f = 0.24 (hexanes/EtOAc 3:1). **^{1}H NMR** (400 MHz, $CDCl_3$): *δ* (ppm) = 7.77 (ddt, J = 7.6, 2.1, 0.9 Hz, 2H), 7.55 (ddd, J = 7.1, 6.0, 1.1 Hz, 2H), 7.45 – 7.37 (m, 2H), 7.32 (tdd, J = 7.4, 5.8, 1.2 Hz, 2H), 4.61 (dtd, J = 21.2, 10.6, 5.6 Hz, 2H), 4.23 (q, J = 5.2, 4.0 Hz, 1H), 4.13 – 3.88 (m, 2H), 3.88 – 2.58 (m, 8H), 1.35 (s, 9H). **^{13}C NMR** (101 MHz, $CDCl_3$): *δ* (ppm) = 155.2, 143.8, 141.5, 127.9, 127.3, 120.2, 67.1, 62.0, 53.3, 47.4, 44.4, 42.5, 29.8, 24.6. **HRMS** (ESI): $C_{25}H_{30}N_2O_4NaS_2Se^+$ $[M+Na]^+$: calc. m/z 589.07044, found: 589.07009.

(4aS,8aR)-4-(tert-butylsulfonyl)octahydro-[1,2]thiaselenino[4,5-b]pyrazine (S15)

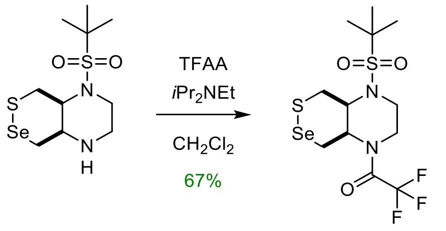

Busyl-piperazine **8** (35 mg, 0.10 mmol, 1.0 equiv.) was dissolved in anhydrous CH_2Cl_2 (1.0 mL, 0.1 M) and the yellow solution was cooled to 0 °C. *i*Pr_2NEt (53 µL, 0.31 mmol, 3.0 equiv.) and trifluoroacetic anhydride (30 µL, 0.21 mmol, 2.1 equiv.) were added sequentially, and the reaction was allowed to warm to room temperature and stirred for 1 h. The reaction was filtered over a silica plug eluting with CH_2Cl_2 and the filtrate was concentrated. The residue was purified by chromatography (SiO_2, 50% CH_2Cl_2 in hexanes initially, grading to 100% CH_2Cl_2), affording orthogonally protected **S15** (30 mg, 68 µmol, 68%) as a pale-yellow solid.

TLC R_f = 0.52 (CH_2Cl_2). **^{1}H NMR** (400 MHz, $CDCl_3$): *δ* (ppm) = 4.82 – 4.57 (m, 1H), 4.20 (s, 1H), 4.09 – 3.93 (m, 2H), 3.83 – 3.60 (m, 4H), 3.37 – 3.17 (m, 2H), 1.39 (s, 9H). **^{13}C NMR** (101 MHz, $CDCl_3$): *δ* (ppm) = 156.8 (q, J = 29.4 Hz), 116.0 (q, J = 288.0 Hz), 62.2, 53.6, 53.4, 44.3, 43.1, 36.6, 29.3, 24.5. **HRMS** (ESI): $C_{12}H_{19}ClF_3N_2O_3S_2Se^-$ $[M+Cl]^-$: calc. m/z 474.96484, found: 474.96483.

(4aS,8aR)-4-(tert-butylsulfonyl)octahydro-[1,2]thiaselenino[4,5-b]pyrazine (S16)

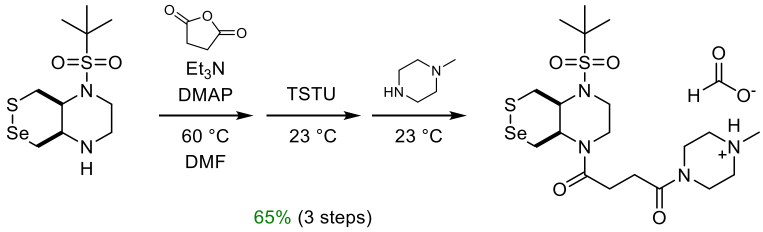

Busyl-piperazine **8** (50 mg, 0.15 mmol, 1.0 equiv.) was dissolved in anhydrous DMF (1.5 mL, 0.1 M). To the yellow solution, Et_3N (97 µL, 0.73 mmol, 5.0 equiv.), succinic anhydride (44 mg, 0.44 mmol, 3.0 equiv.) and DMAP (18 mg, 0.15 mmol, 1.0 equiv.) were added sequentially, the reaction was heated to 60 °C and stirred for 16 h. Upon full conversion to the intermediary succinate, the reaction was allowed to warm to room temperature, TSTU () was added, the mixture was stirred for 1 h, followed by addition of 1-methylpiperazine (). After stirring for another 30 min, the reaction mixture was diluted with CH_2Cl_2 (10 mL) and transferred into a separatory funnel. The organic layer was washed with brine (3×10 mL), dried over $MgSO_4$, filtered and concentrated. The orange residue was directly purified via chromatography (reverse phase, 10% CH_3CN in H_2O initially, grading to 100% CH_3CN, 0.1% formic acid), affording **S16** as a pale yellow solid (50 mg, 95 µmol, 65%).

Note: **S16** was synthesised with the aim to produce the control probe P-SSeC. Unfortunately, busyl-deprotection conditions using TfOH were not tolerated by the piperazine amide side chain and instead produced the fully deprotected piperazine.

TLC R_f = 0.38 (CH_2Cl_2/MeOH 95:5). **^{1}H NMR** (400 MHz, MeOD-*d_4*): *δ* (ppm) = 8.37 (s, 1H), 4.79 – 4.68 (m, 1H), 4.28 (s, 1H), 3.97 – 3.67 (m, 10H), 3.63 (dd, J = 13.0, 9.0 Hz, 1H), 3.27 (dd, J = 11.6, 3.1 Hz, 1H), 3.15 – 3.06 (m, 1H), 3.01 (t, J = 5.1 Hz, 2H), 2.92 (t, J = 5.2 Hz, 2H), 2.75 – 2.69 (m, 4H), 2.68 (s, 3H), 1.40 (s, 9H)z. **^{13}C NMR** (101 MHz, MeOD-*d_4*): *δ* (ppm) = 173.9, 172.8, 168.2, 62.8, 54.7, 54.6, 54.3, 53.4, 45.6, 44.5, 44.5, 44.2, 40.9, 39.5, 29.3, 29.1, 28.5, 24.9. **HRMS** (ESI): $C_{19}H_{35}N_4O_4S_2Se^+$ $[M+H]^+$: calc. m/z 527.12595, found: 527.12536.

H-SeS66C-PQ (SeSC)

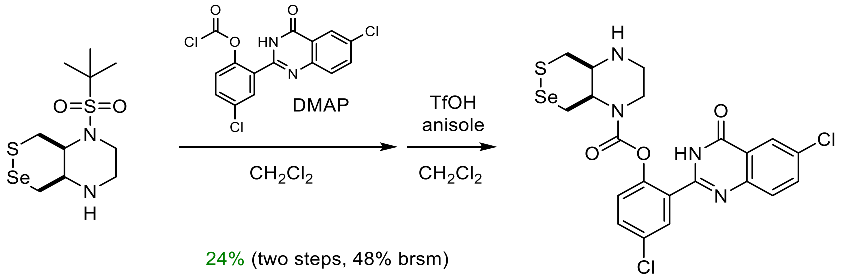

Step 1: Crude PQOC(O)Cl[38] (60 wt%, 0.18 g, 0.31 mmol, 1.2 equiv.) was suspended in anhydrous CH_2Cl_2 (3.0 mL, 0.1 M) and filtered through a pad of cotton. To the pale-yellow solution, a solution of busyl-piperazine **8** (88 mg, 0.26 mmol, 1.0 equiv.) and DMAP (0.13 g, 1.0 mmol, 4.0 equiv.) in anhydrous CH_2Cl_2 (3.0 mL, 0.1 M) was added dropwise. The reaction was stirred at room temperature for 2 h, then filtered over Celite. The filtrate was concentrated *in vacuo* and the residue was purified by chromatography (SiO_2, DCM initially, grading to 5% MeOH), to recover unreacted starting material **8** (42 mg, 0.12 mmol, 48%) and obtain **Bus-SeSC** (68 mg, 0.10 mmol, 38%) in satisfactory purity.

Step 2: The carbamate obtained above was dissolved in anhydrous CH_2Cl_2 (2.0 mL, 50 mM), and anisole (0.22 mL, 2.0 mmol, 20 equiv.) then TfOH (63 µL, 0.70 mmol, 7.0 equiv.) were added. An immediate colour change to pink occurred, and quantitative removal of the busyl protecting group was confirmed by LCMS within 5 min. All volatiles were removed under a stream of nitrogen, the residue was redissolved in MeOH/H_2O and purified by preparative HPLC (reverse phase, UV detection at 300 nm, 10% CH_3CN in H_2O initially, grading to 65% CH_3CN over 21 min, 0.1% formic acid). Lyophilisation afforded **SeSC** (35 mg, 63 µmol, 62%) as a colourless solid.

Notes: (1) Purity of PQOC(O)Cl was estimated by reaction with methyl-piperazine (Me-pip). The resulting ratio of Me-pip-PQ-carbamate vs PQOH was determined by LCMS. (2) Both ^{1}H and ^{13}C NMR spectroscopy of **SeSC** revealed the presence of two rotameric species.

^{1}H NMR (400 MHz, $CDCl_3$): δ (ppm) = 8.28 – 8.22 (m, 1H), 7.98 (dd, J = 16.5, 2.6 Hz, 1H), 7.78 – 7.69 (m, 2H), 7.52 (dt, J = 8.7, 2.5 Hz, 1H), 7.20 (dd, J = 15.5, 8.7 Hz, 1H), 4.52 (dt, J = 12.3, 3.3 Hz, 0.5H), 4.42 (dt, J = 12.2, 3.6 Hz, 0.5H), 4.00 (dd, J = 12.9, 3.7 Hz, 0.5H), 3.95 – 3.84 (m, 1H), 3.76 (t, J = 12.2 Hz, 0.5H), 3.56 (dd, J = 14.4, 2.3 Hz, 1H), 3.41 – 3.14 (m, 3.5H), 3.12 – 2.89 (m, 1.5H), 2.62 (dd, J = 12.4, 3.8 Hz, 0.5H), 2.45 (dd, J = 12.2, 3.9 Hz, 0.5H). **^{13}C NMR** (101 MHz, $CDCl_3$): δ (ppm) = 160.9, 153.2, 152.3, 149.2, 147.5, 135.7, 133.7, 132.6, 130.7, 127.7, 126.2, 125.2, 122.3, 53.7, 53.5, 53.2, 52.6, 45.3, 45.2, 20.7, 19.6. **HRMS** (ESI): $C_{21}H_{17}Cl_2N_4O_3SSe^-$ [M-H]$^-$: calc. m/z 554.95691, found: 554.95564.

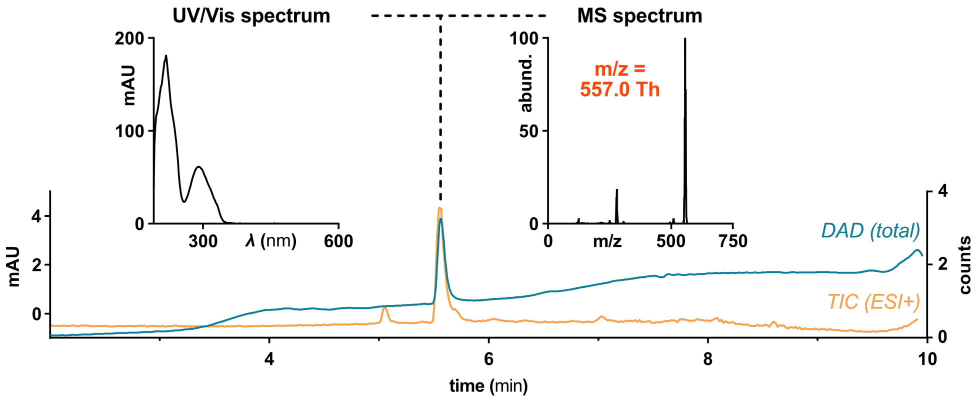

P-SeS66C-PQ (P-SeSC)

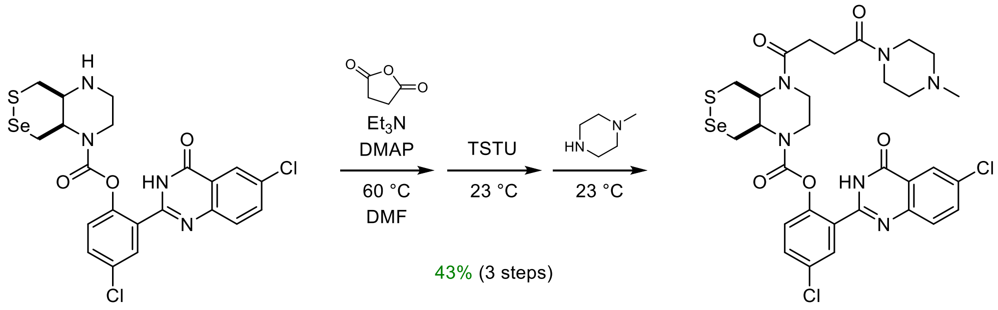

SeSC (8.0 mg, 14 µmol, 1.0 equiv.) was dissolved in anhydrous DMF (1 mL, 15 mM). To the pale-yellow solution, Et_3N (15 µL, 86 µmol, 6.0 equiv.), succinic anhydride (7.1 mg, 71 µmol, 5.0 equiv.) and DMAP (1.8 mg, 14 µmol, 1.0 equiv.) were added sequentially, the reaction was heated to 60 °C and stirred for 16 h. Upon full conversion to the intermediary succinate, the reaction was allowed to warm to room temperature, TSTU (22 mg, 72 µmol, 5.0 equiv.) was added, the mixture was stirred for 1 h, followed by addition of 1-methylpiperazine (16 µL, 0.14 mmol, 10 equiv.). After stirring for another 30 min, the reaction mixture was diluted with CH_2Cl_2 (10 mL) and transferred into a separatory funnel. The organic layer was washed with brine (3×10 mL), dried over $MgSO_4$, filtered and concentrated. The orange residue was directly purified via chromatography (reverse phase, 10% CH_3CN in H_2O initially, grading to 100% CH_3CN, 0.1% formic acid), affording **P-SeSC** as a pale yellow solid (50 mg, 95 µmol, 65%).

^{1}H NMR (400 MHz, $CDCl_3$): δ (ppm) = 8.45 (br s, 1H), 8.19 (d, J = 2.4 Hz, 1H), 7.85 – 7.81 (m, 2H), 7.73 (d, J = 8.7 Hz, 1H), 7.64 (dd, J = 8.7, 2.6 Hz, 1H), 7.38 (d, J = 8.7 Hz, 1H), 4.75 – 3.86 (m, 3H), 3.86 – 3.37 (m, 9H), 3.27 – 3.08 (m, 1H), 2.77 – 2.47 (m, 9H), 2.41 (s, 3H).. **^{13}C NMR** (101 MHz, MeOD-*d$_4$*): δ (ppm) = 172.5, 171.2, 152.8, 147.4, 135.0, 132.7, 131.7, 131.2, 129.7, 129.0, 128.9, 125.2, 125.0, 122.3, 56.3, 56.1, 55.9, 54.2, 53.9, 44.3, 42.1, 40.7, 40.6, 27.5, 27.3, 23.3, 16.1, 15.9, 15.7. **HRMS** (ESI): $C_{30}H_{31}Cl_2N_6O_5SSe^-$ [M-H]$^-$: calc. m/z 737.06244, found: 737.06147.

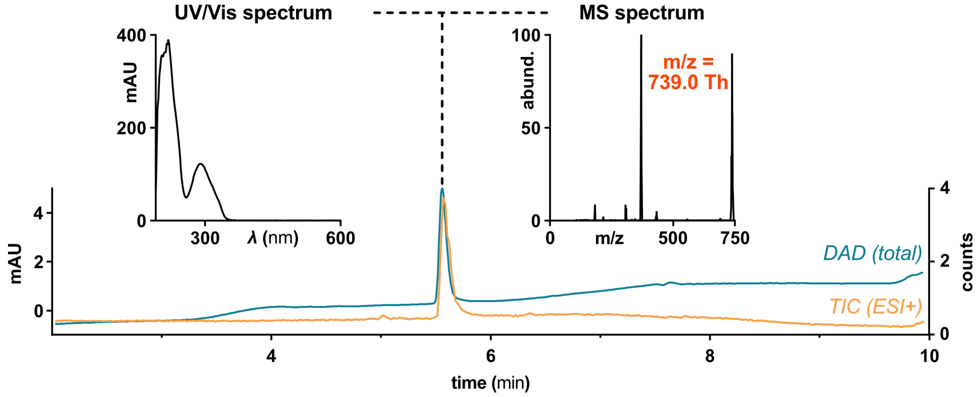

H-SSe66C-PQ (SSeC)

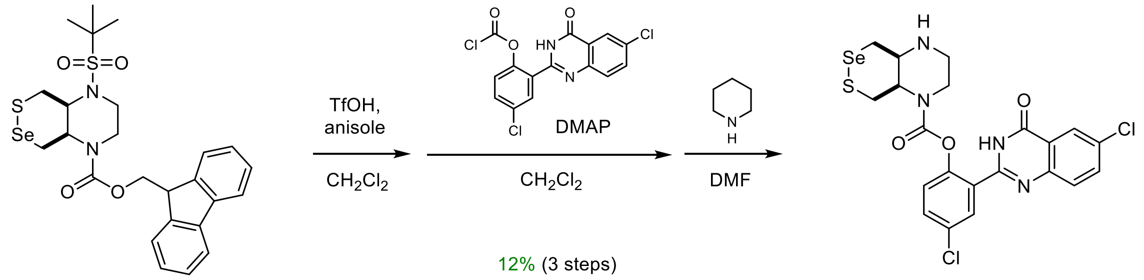

Step 1: Orthogonally protected piperazine **S14** (20 mg, 35 µmol, 1.0 equiv.) was dissolved in anhydrous CH_2Cl_2 (0.7 mL, 0.05 M), and anisole (77 µL, 0.71 mmol, 20 equiv.) then TfOH (16 µL, 0.18 mmol, 5.0 equiv.) were added. An immediate colour change to pink occurred, and full conversion of the starting material was confirmed by TLC (hexanes/EtOAc 3:1) within 10 min. Et_3N (24 µL, 0.18 mmol, 5.0 equiv.) was added and the reaction mixture was filtered over a pad of silica (CH_2Cl_2 initially, then 5% MeOH in CH_2Cl_2). The CH_2Cl_2 fraction was discarded, concentration of the 5% MeOH fraction gave the targeted Fmoc-piperazine as a pale-yellow oil.

Step 2: Crude PQOC(O)Cl[38] (60 wt%, 40 mg, 39 µmol, 1.1 equiv.) was suspended in anhydrous CH_2Cl_2 (0.80 mL, 50 mM) and filtered through a pad of cotton. To the pale-yellow solution, a solution of the Fmoc-piperazine obtained above (16 mg, 35 µmol, 1.0 equiv.) and DMAP (8.7 mg, 71 µmol, 2.0 equiv.) in anhydrous CH_2Cl_2 (0.70 mL, 50 mM) was added dropwise. The reaction was stirred at room temperature for 2 h, then filtered over Celite. The filtrate was concentrated *in vacuo* and the residue was purified by chromatography (SiO_2, CH_2Cl_2 initially, grading to 5% MeOH), to recover unreacted Fmoc-piperazine intermediate (12 mg, 27 µmol, 76%) and obtain **Fmoc-SSeC** (6.0 mg, 7.7 µmol, 22%) in satisfactory purity.

Step 3: **Fmoc-SSeC** (6.0 mg, 7.7 µmol, 1.0 equiv.) was dissolved in DMF (0.20 mL, 50 mM) and to the pale yellow solution, piperidine (15 µL, 0.15 mmol, 20 equiv.) was added. An immediate colour change to bright yellow occurred, and the reaction was stirred at room temperature for 1 h. Upon completion, the reaction was directly purified by preparative HPLC (reverse phase, UV detection at 300 nm, 10% CH_3CN in H_2O initially, grading to 65% CH_3CN over 21 min, 0.1% formic acid), affording **SSeC** (2.4 mg, 4.3 µmol, 56%) as a colourless solid after lyophilisation.

Notes: (1) Purity of PQOC(O)Cl was estimated by reaction with methyl-piperazine (Me-pip). The resulting ratio of Me-pip-PQ-carbamate vs PQOH was determined by LCMS. (2) Purification of the carbamate intermediate was solely done to recover starting material from the crude material. Separation was straightforward and elution of both thiaselenane-containing fractions could be followed by eye. NMR analysis was of qualitative nature, to confirm its identity (diagnostic protons (ppm): 8.26 (dd, 4.2, 2.4 Hz, 1H) and 4.23 (dt, *J* = 9.9, 5.5 Hz, 1H), which integrate 1:1). Analysis by LCMS(ESI) again wasn't helpful due to poor ionisation. (3) Both ^{1}H and ^{13}C NMR spectroscopy of **SSeC** revealed the presence of two rotameric species.

^{1}H NMR (400 MHz, $CDCl_3$): δ (ppm) = 8.20 (d, J = 2.4 Hz, 1H), 7.85 (dt, J = 8.8, 2.9 Hz, 1H), 7.81 (t, J = 2.6 Hz, 1H), 7.74 (dd, J = 8.7, 1.0 Hz, 1H), 7.62 (dd, J = 8.7, 2.6 Hz, 1H), 7.37 (dd, J = 26.5, 8.7 Hz, 1H), 4.35 (dt, J = 12.0, 3.5 Hz, 0.5H), 4.03 – 3.90 (m, 1H), 3.75 (dd, J = 13.7, 12.0 Hz, 0.5H), 3.65 (dt, J = 13.5, 2.9 Hz, 1H), 3.59 – 3.50 (m, 1H), 3.17 – 2.80 (m, 4.5H), 2.72 – 2.59 (m, 1H), 2.09 (dd, J = 13.6, 3.8 Hz, 0.5H). **^{13}C NMR** (101 MHz, $CDCl_3$): δ (ppm) = 154.1, 153.4, 136.4, 134.2, 133.0, 132.4, 131.0, 130.4, 126.6, 126.2, 123.6, 55.4, 54.7, 54.1, 53.9, 46.0, 41.4, 40.5, 34.8, 29.7, 28.8. **HRMS** (ESI): $C_{21}H_{19}Cl_2N_4O_3SSe^+$ $[M+H]^+$: calc. m/z 556.97146, found: 556.97044.

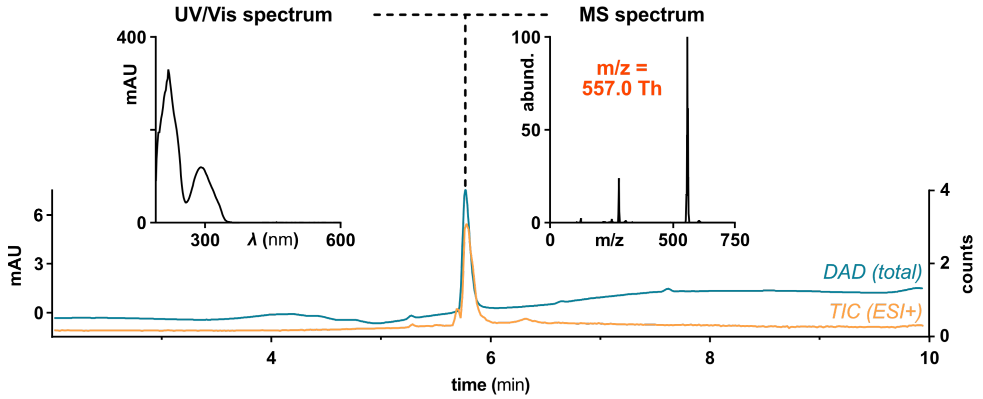

H-SeS66T-PQ (SeST)

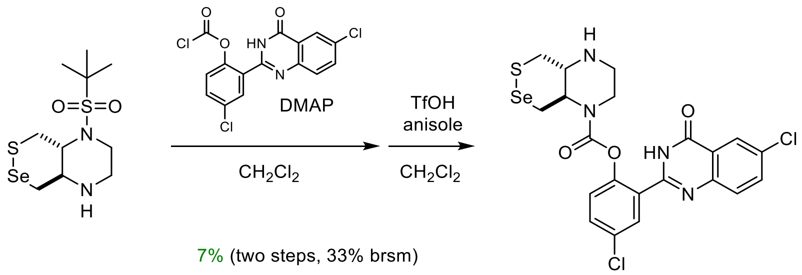

Step 1: Crude PQOC(O)Cl[38] (60 wt%, 0.11 g, 0.17 mmol, 1.2 equiv.) was suspended in anhydrous CH_2Cl_2 (3.0 mL, 0.1 M) and a solution of busyl-piperazine **S13** (49 mg, 0.14 mmol, 1.0 equiv.) and DMAP (70 mg, 0.57 mmol, 4.0 equiv.) in anhydrous CH_2Cl_2 (3.0 mL, 0.1 M) was added dropwise. The reaction was stirred at room temperature for 2 h, then filtered over Celite. The filtrate was concentrated *in vacuo* and the residue was purified by chromatography (SiO_2, DCM initially, grading to 5% MeOH), to recover unreacted starting material **S13** (16 mg, 47 µmol, 33%) and obtain **Bus-SeST** (32 mg, 44 µmol, 31%) in satisfactory purity.

Step 2: The carbamate obtained above was dissolved in anhydrous CH_2Cl_2 (3.0 mL, 15 mM), and anisole (0.10 mL, 0.96 mmol, 20 equiv.) then TfOH (30 µL, 0.33 mmol, 7.0 equiv.) were added. An immediate colour change to pink occurred, and quantitative removal of the busyl protecting group was confirmed by LCMS within 5 min. All volatiles were removed under a stream of nitrogen, the residue was redissolved in MeOH/H_2O and purified by preparative HPLC (reverse phase, UV detection at 300 nm, 10% CH_3CN in H_2O initially, grading to 65% CH_3CN over 21 min, 0.1% formic acid). Lyophilisation afforded **SeST** (6.1 mg, 11 µmol, 23%) as a colourless solid.

^{1}H NMR (400 MHz, $CDCl_3$): δ (ppm) = 8.16 (d, J = 2.5 Hz, 1H), 7.84 (d, J = 2.5 Hz, 1H), 7.80 (dd, J = 8.7, 2.5 Hz, 1H), 7.70 (d, J = 8.7 Hz, 1H), 7.60 (dd, J = 8.8, 2.7 Hz, 1H), 7.52 (d, J = 9.0 Hz, 1H), 4.49 (s, 1H), 4.23 – 3.99 (m, 1H), 3.71 – 3.45 (m, 4H), 3.41 – 3.26 (m, 3H), 3.15 (d, J = 15.0 Hz, 1H), 3.18 – 3.01 (m, 1H). **HRMS** (ESI): $C_{21}H_{17}Cl_2N_4O_3SSe^-$ [M-H]$^-$: calc. m/z 554.95691, found: 554.95562.

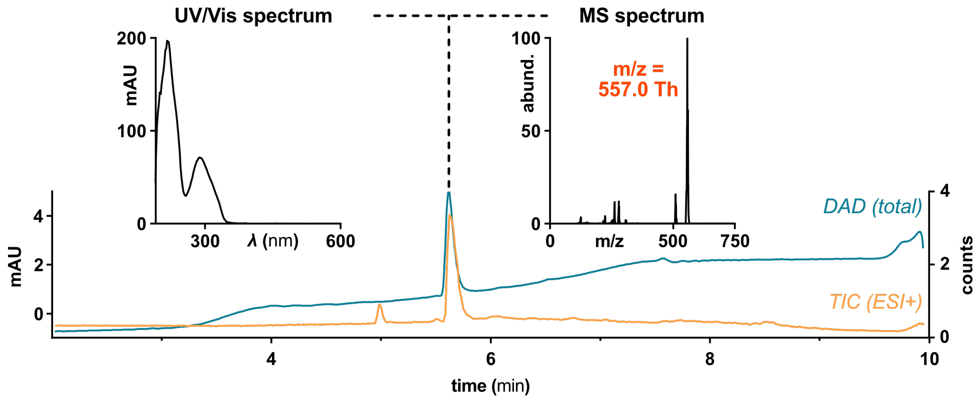

SeSC-N

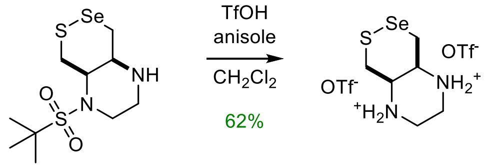

Busyl-protected piperazine **8** (20 mg, 58 µmol, 1.0 equiv.) was dissolved in anhydrous CH_2Cl_2 (1.0 mL, 50 mM), and anisole (0.13 mL, 1.2 mmol, 20 equiv.) then TfOH (36 µL, 0.40 mmol, 6.9 equiv.) were added. Within minutes, the homogeneous reaction mixture turned cloudy; after 24 h, full conversion of the starting material was confirmed by LCMS. The stir plate was turned off and the precipitate was allowed to settle. The solution was discarded, and the residue was washed with Et_2O (3×1 mL), affording the fully deprotected thiaselenane as an off-white solid (8.0 mg, 36 µmol, 62%).

^{1}H NMR (400 MHz, CD_3OD): *δ* (ppm) = 4.39 – 4.34 (m, 1H), 4.08 (s, 1H), 3.79 – 3.71 (m, 1H), 3.62 – 3.50 (m, 5H), 3.45 – 3.38 (m, 1H), 3.19 – 3.10 (m, 1H).

SeSC-D

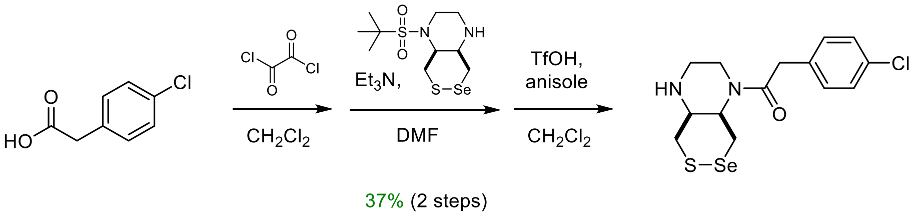

4-Chlorophenylacetic acid (21 mg, 0.12 mmol, 2.0 equiv.) was dissolved in anhydrous CH_2Cl_2 (0.1 M, 1.2 mL) and oxalyl chloride (10 µL, 0.12 mmol, 2.0 equiv.) was added, followed by a single drop of DMF. Vigorous gas formation occurred, and the reaction was stirred for 30 min at room temperature. All volatiles were removed under a stream of nitrogen and the residue was re-dissolved in DMF (0.2 M, 0.6 mL). A solution of Bus-protected piperazine **8** (20 mg, 58 µmol, 1.0 equiv.), Et_3N (78 µL, 0.58 mmol, 10 equiv.) and DMAP (0.70 mg, 5.8 µmol, 0.1 equiv.) in anhydrous DMF (0.6 mL, 0.1 M) was added, and the suspension was stirred for 1 h. Upon completion, the mixture was diluted with EtOAc (5 mL) and washed with NH_4Cl (sat. aq., 5 mL), $NaHCO_3$ (sat. aq., 2×5 mL) and brine (sat. aq., 5 mL). The organic layer was dried over $MgSO_4$, filtered and concentrated *in vacuo*. The residue was dissolved in anhydrous CH_2Cl_2 (1.0 mL, 0.05 M) and anisole (95 µL, 0.87 mmol, 15 equiv.) then TfOH (26 µL, 0.29 mmol, 5.0 equiv.) were added. An immediate colour change to pink occurred, and full conversion of the starting material was confirmed by TLC (hexanes/EtOAc 3:1) within 3 h. All volatiles were removed under a stream of nitrogen, the residue was re-dissolved in MeOH/H_2O (9:1, 1.0 mL) and the pH was adjusted to ca. 5 using $NaHCO_3$. Purification by preparative HPLC (reverse phase, UV detection at 300 nm, 10% CH_3CN in H_2O initially, grading to 65% CH_3CN over 25 min, 0.1% formic acid) provided **SeSC-D** (8.0 mg, 21 µmol, 37%) as a pale-yellow solid upon lyophilisation. *Note*: NMR spectroscopy revealed the presence of two rotameric species.

^{1}H NMR (400 MHz, CD_3OD): *δ* (ppm) = 7.40 – 7.33 (m, 2H), 7.31 – 7.22 (m, 2H), 5.13 (dt, *J* = 12.5, 3.8 Hz, 0.7H), 4.70 – 4.57 (m, 0.7H), 4.10 (dd, *J* = 15.1, 3.9 Hz, 0.7H), 3.94 – 3.71 (m, 5H), 3.56 – 3.35 (m, 3H), 3.28 – 3.05 (m, 2H), 2.70 (dd, *J* = 12.8, 4.1 Hz, 0.7H), 2.41 (dd, *J* = 13.0, 3.8 Hz, 0.3H). **HRMS** (ESI): $C_{14}H_{18}ON_2ClSSe^+$ $[M+H]^+$: calc. m/z 376.9988, found: 376.9993.

P-SeSC-D

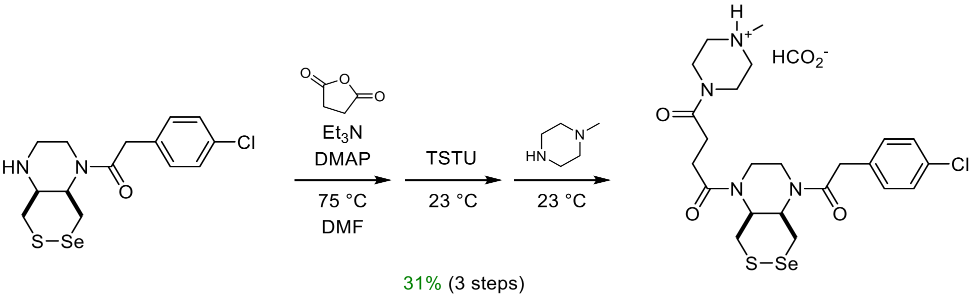

SeSC-D (24 mg, 63 µmol, 1.0 equiv.) was dissolved in anhydrous DMF (0.60 mL, 0.1 M). To the pale-yellow solution, Et_3N (60 µL, 0.43 mmol, 7.0 equiv.), succinic anhydride (20 mg, 0.2 mmol, 3.2 equiv.) and DMAP (8.0 mg, 66 µmol, 1.0 equiv.) were added sequentially, the reaction was heated to 75 °C and stirred for 72 h. Upon full conversion to the intermediary succinate, the reaction was allowed to warm to room temperature, TSTU (56 mg, 0.19 mmol, 3.0 equiv.) was added, the mixture was stirred for 1 h, followed by addition of 1-methylpiperazine (69 µL, 0.63 mmol, 10 equiv.). After stirring for

another 2 h, the reaction mixture was diluted with CH_2Cl_2 (5 mL) and transferred into a separatory funnel. The organic layer was washed with brine (3×5 mL), dried over $MgSO_4$, filtered and concentrated. The orange residue was directly purified via preparative HPLC (reverse phase, 10% CH_3CN in H_2O initially, grading to 100% CH_3CN over 18 min, 0.1% formic acid), affording **P-SeSC-D** as a colourless film (14 mg, 23 µmol, 37%).

^{1}H NMR (600 MHz, CD_3OD): δ (ppm) = 8.33 (s, 1H), 7.33 (dt, *J* = 8.5, 2.6, 1.7 Hz, 2H), 7.26 (dt, *J* = 8.4, 2.4 Hz, 2H), 4.61 (s, 1H), 4.49 (s, 1H), 4.09 – 3.98 (m, 1H), 3.97 – 3.57 (m, 11H), 3.16 – 3.08 (m, 1H), 2.89 – 2.79 (m, 2H), 2.79 – 2.73 (m, 2H), 2.72 – 2.59 (m, 4H), 2.56 (d, *J* = 6.1 Hz, 3H). **^{13}C NMR** (151 MHz, CD_3OD): δ (ppm) = 174.2, 173.3, 172.8, 167.8, 135.0, 133.9, 131.8 (2C), 129.8 (2C), 55.2, 55.0, 49.9, 45.1 (2C), 43.9, 41.5, 41.0, 29.1, 28.6. **HRMS** (ESI): $C_{23}H_{32}ClN_4O_3SSe^+$ [M+H]$^+$: calc. m/z 559.1043, found: 559.1041

A3-D

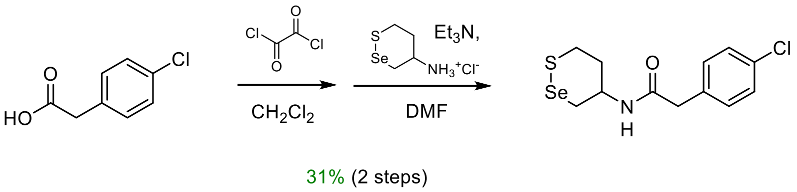

4-Chlorophenylacetic acid (9.4 mg, 55 µmol, 2.0 equiv.) was dissolved in anhydrous CH_2Cl_2 (0.1 M, 0.6 mL) and oxalyl chloride (4.7 µL, 55 µmol, 2.0 equiv.) was added, followed by a single drop of DMF. Vigorous gas formation occurred, and the reaction was stirred for 30 min at room temperature. All volatiles were removed under a stream of nitrogen and the residue was re-dissolved in DMF (0.2 M, 0.3 mL). A solution of 1,2-thiaselenan-4-amine hydrochloride[36] (6.0 mg, 27 µmol, 1.0 equiv.), Et_3N (37 µL, 0.27 mmol, 10 equiv.) and DMAP (0.34 mg, 2.7 µmol, 0.1 equiv.) in anhydrous DMF (0.3 mL, 0.1 M) was added, and the suspension was stirred for 1 h. Reaction progress was monitored by LCMS (using methyl-piperazine to assess the fraction of remaining acid chloride) and upon completion, the mixture was diluted with MeOH/H_2O (9:1, 1.0 mL). Purification by preparative HPLC (reverse phase, UV detection at 300 nm, 10% CH_3CN in H_2O initially, grading to 65% CH_3CN over 25 min, 0.1% formic acid) provided **A3-D** (2.8 mg, 8.4 µmol, 31%) as a pale-yellow solid upon lyophilisation.

^{1}H NMR (400 MHz, CD_3OD): δ (ppm) = 7.34 – 7.25 (m, 4H), 4.06 (tt, *J* = 10.5, 3.5 Hz, 1H), 3.48 (s, 2H), 3.26 (ddd, *J* = 14.1, 5.6, 3.0 Hz, 1H), 3.12 (dd, *J* = 14.3, 10.7 Hz, 1H), 3.02 – 2.88 (m, 2H), 2.17 (ddt, *J* = 13.7, 5.3, 2.6 Hz, 1H), 1.72 (dtd, *J* = 14.1, 11.1, 3.0 Hz, 1H). **HRMS** (ESI): $C_{12}H_{13}ONClSSe^-$ [M-H]$^-$: calc. m/z 333.9577, found: 333.9568.

RX1-D

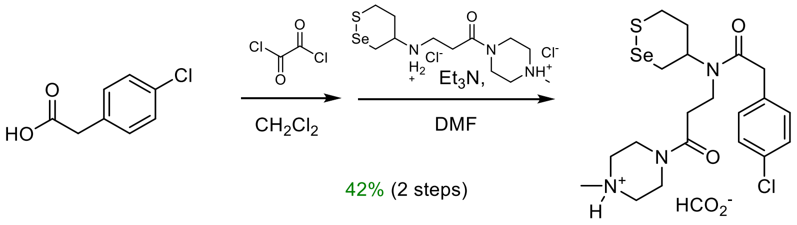

4-Chlorophenylacetic acid (8.4 mg, 49 µmol, 2.0 equiv.) was dissolved in anhydrous CH_2Cl_2 (0.1 M, 0.5 mL) and oxalyl chloride (4.2 µL, 49 µmol, 2.0 equiv.) was added, followed by a single drop of DMF. Vigorous gas formation occurred, and the reaction was stirred for 30 min at room temperature. All volatiles were removed under a stream of nitrogen and the residue was re-dissolved in DMF (0.2 M, 0.3 mL). A solution of *P*-1,2-thiaselenan-4-amine dihydrochloride[3] (10 mg, 24 µmol, 1.0 equiv.), Et_3N (33 µL, 0.24 mmol, 10 equiv.) and DMAP (0.30 mg, 2.4 µmol, 0.1 equiv.) in anhydrous DMF (0.3 mL, 0.1 M) was added, and the suspension was stirred for 1 h. Reaction progress was monitored by LCMS (using piperidine to assess the fraction of remaining acid chloride) and upon completion, the mixture was diluted with MeOH/H_2O (9:1, 1.0 mL). Purification by preparative HPLC (reverse phase, UV detection at 300 nm, 10% CH_3CN in H_2O initially, grading to 65% CH_3CN over 25 min, 0.1% formic acid) provided **RX1-D** (5.0 mg, 10 µmol, 42%) as a pale-yellow solid upon lyophilisation.

Note: NMR spectroscopy revealed the presence of two rotameric species.

^{1}H NMR (400 MHz, CD_3OD): δ (ppm) = 8.30 (s, 1H), 7.40 – 7.22 (m, 4H), 4.21 (tdd, *J* = 11.1, 5.0, 2.8 Hz, 1H), 3.87 – 3.77 (m, 2H), 3.76 – 3.60 (m, 4H), 3.58 – 3.48 (m, 2H), 3.30 – 3.12 (m, 2H), 2.84 (dt, *J* = 27.5, 5.1 Hz, 3H), 2.70 (dtd, *J* = 18.3, 9.0, 8.5, 5.9 Hz, 3H), 2.63 – 2.50 (m, 4H), 2.28 (q, *J* = 12.4 Hz, 0.3H), 2.13 – 2.03 (m, 0.3H), 2.02 – 1.92 (m, 1.3H). **HRMS** (ESI): $C_{20}H_{29}O_2N_3ClSSe^+$ [M+H]$^+$: calc. m/z 490.0829, found: 490.0830.

8 Analytics

8.1 Atomic coordinates, imaginary frequencies and energies of optimised structures

A (SeST)
E= -3613.24
Nimag=0

C	0.260424000	1.517981000	-0.174695000
H	0.304426000	1.955499000	0.821438000
C	-0.330529000	0.109400000	-0.136810000
H	-0.206398000	-0.342453000	-1.123899000
C	0.366299000	-0.792191000	0.870044000
H	-0.150051000	-1.751961000	0.892637000
H	0.402586000	-0.355669000	1.867463000
C	-1.989337000	2.622065000	-0.106478000
H	-2.806703000	2.782982000	-0.808859000
H	-1.812113000	3.531347000	0.468107000
C	-2.227160000	1.404833000	0.788187000
H	-1.701487000	1.522301000	1.739257000
H	-3.287382000	1.313474000	1.003084000
C	-2.564507000	-0.802197000	-0.358595000
N	-0.749757000	2.358343000	-0.930082000
H	-1.011337000	1.844640000	-1.773216000
N	-1.763776000	0.226822000	0.103398000
O	-3.862959000	-0.569145000	-0.091688000
O	-2.112391000	-1.768944000	-0.935684000
S	2.852118000	0.910175000	0.339553000
C	1.634492000	1.610816000	-0.805790000
H	1.923307000	2.659812000	-0.940358000
H	1.676934000	1.092566000	-1.765632000
Se	2.190085000	-1.179423000	0.270408000
C	-4.753199000	-1.630063000	-0.515863000
H	-4.488244000	-2.559886000	-0.011674000
H	-5.747895000	-1.297818000	-0.225636000
H	-4.683313000	-1.766419000	-1.595472000
H	-0.323803000	3.236720000	-1.228207000

A(SeSC)
E= -3620.25;
Nimag=0

C	0.150200000	-1.284200000	0.941300000
C	-1.303100000	-2.598900000	-0.620700000
N	0.090800000	-2.315900000	-0.155700000
C	-0.509100000	-0.002600000	0.441600000
N	-1.866900000	-0.336500000	0.037400000
C	-1.992500000	-1.313200000	-1.020100000
H	-0.474800000	-1.677500000	1.745000000
H	-1.812100000	-3.068700000	0.221000000
H	-1.241100000	-3.298800000	-1.454700000
H	0.250300000	0.214400000	-1.608900000
O	-2.621000000	1.611000000	0.973100000
Se	2.018100000	1.307200000	-0.218000000
H	-0.361400000	1.687500000	-0.839600000
H	-1.580700000	-0.945800000	-1.967800000
H	-3.044700000	-1.530100000	-1.184800000
C	1.592100000	-1.182900000	1.405200000
S	2.709600000	-0.759200000	0.042900000
C	0.195500000	0.764200000	-0.670900000
H	1.672900000	-0.430100000	2.189000000
H	1.923600000	-2.144600000	1.813000000
H	-0.593200000	0.656300000	1.304400000
H	0.707800000	-1.960400000	-0.901400000
C	-2.830800000	0.639100000	0.279600000
O	-3.988800000	0.334000000	-0.328300000
C	-5.059700000	1.275000000	-0.072200000
H	-4.771300000	2.268400000	-0.417000000
H	-5.910100000	0.895900000	-0.635300000
H	-5.277900000	1.306500000	0.995700000
H	0.527800000	-3.176900000	0.177400000

TSA-B (SeST); E= -4899.69; Nimag=-321

C	4.841852000	1.188745000	8.323442000
H	4.396605000	1.999506000	8.898467000
C	4.139112000	1.001963000	6.979755000
H	4.503217000	0.080399000	6.521849000
C	2.629203000	0.897667000	7.097598000
H	2.202524000	0.907002000	6.097890000
H	2.209393000	1.700642000	7.701299000
C	6.225942000	3.114134000	7.504132000
H	7.106284000	3.252208000	6.876785000
H	6.297713000	3.750667000	8.386164000
C	4.924682000	3.335825000	6.730531000
H	4.133538000	3.668021000	7.406598000
H	5.070559000	4.105930000	5.979813000
C	4.594408000	1.843384000	4.746725000
N	6.236980000	1.683704000	7.977119000
H	6.601845000	1.086092000	7.234154000
N	4.545755000	2.098202000	6.101745000
O	5.001378000	2.931900000	4.058065000
O	4.310427000	0.765187000	4.268124000
S	3.352503000	-0.176752000	10.122276000
C	4.902826000	-0.046160000	9.199566000
H	5.730406000	0.049941000	9.913777000
H	5.070056000	-0.933733000	8.583858000
Se	2.072367000	-0.811814000	7.878263000
C	5.031049000	2.741683000	2.625385000
H	5.364619000	3.694625000	2.218848000
H	5.724934000	1.940775000	2.366998000
H	4.034079000	2.489556000	2.262105000
S	1.253851000	-1.846047000	5.927405000
C	-0.539312000	-1.609208000	6.085825000
H	-0.859030000	-1.794802000	7.111370000
H	-0.797854000	-0.589354000	5.799974000
H	-1.032623000	-2.310531000	5.409260000
H	1.609856000	-3.272328000	7.137347000
O	1.885444000	-3.974173000	7.885344000
H	1.109185000	-4.518364000	8.075567000
H	2.324870000	-3.508352000	8.881315000
O	2.761453000	-3.099354000	9.965603000
H	3.032452000	-2.091224000	10.036340000
H	3.496425000	-3.626662000	10.302591000
H	6.846670000	1.573096000	8.787659000

TS^{A-B} (C)
E=-4910.67
Nimag=-221

C	0.709660000	0.678783000	0.666136000
C	2.281753000	2.256347000	-0.505557000
N	0.875089000	1.942406000	-0.131887000
C	1.360297000	-0.481951000	-0.077978000
N	2.747217000	-0.111715000	-0.339825000
C	2.936357000	1.061241000	-1.162301000
H	1.284798000	0.828946000	1.581728000
H	2.796906000	2.511598000	0.421123000
H	2.268039000	3.121765000	-1.169440000
H	0.655209000	-0.204345000	-2.132359000
O	3.448643000	-2.207047000	0.255299000
Se	-1.094726000	-1.682337000	-1.189449000
H	1.322185000	-1.800073000	-1.740755000
H	2.530700000	0.916846000	-2.170293000
H	3.998878000	1.264445000	-1.261395000
C	-0.774089000	0.557943000	0.979221000

S	-1.789188000	0.861139000	-0.495355000
C	0.719252000	-0.974058000	-1.367647000
H	-0.954781000	-0.447450000	1.361990000
H	-1.030292000	1.278568000	1.762227000
H	1.390088000	-1.315370000	0.621373000
H	0.206658000	1.817867000	-0.924720000
C	3.687826000	-1.124142000	-0.236860000
O	4.881604000	-0.730789000	-0.725782000
C	5.926789000	-1.719179000	-0.591913000
H	6.092150000	-1.948162000	0.461675000
H	5.649826000	-2.630816000	-1.122714000
H	6.809401000	-1.261768000	-1.035189000
S	-0.652363000	-3.944632000	-1.156708000
C	-0.913399000	-4.453614000	-2.880160000
H	-0.989204000	-5.543260000	-2.893658000
H	-1.827627000	-4.010612000	-3.274586000
H	-0.060051000	-4.142960000	-3.483116000
H	-2.438346000	-3.763005000	-0.403734000
O	-3.361737000	-3.514291000	0.027698000
H	-4.050794000	-3.998889000	-0.447745000
H	-3.602350000	-2.357944000	0.065437000
O	-3.945295000	-1.165899000	0.115413000
H	-3.224058000	-0.456217000	-0.077258000
H	-4.371882000	-0.925301000	0.948039000
H	0.475944000	2.710861000	0.408739000

B(T)
E= -4263.16
Nimag=0

C	0.233135000	-1.135320000	-0.393432000
H	-0.731031000	-1.417486000	-0.816819000
C	0.554263000	0.305257000	-0.782053000
H	0.977521000	0.272449000	-1.787609000
C	-0.667373000	1.217413000	-0.862586000
H	-1.217510000	1.015611000	-1.780306000
H	-0.343607000	2.259124000	-0.872096000
C	1.147633000	-0.664846000	1.905338000
H	2.022097000	-1.291806000	1.741905000
H	0.847761000	-0.729716000	2.951880000
C	1.405053000	0.766018000	1.476360000
H	0.553723000	1.394642000	1.749196000
H	2.281318000	1.146200000	1.991803000
C	2.852830000	1.021998000	-0.543583000
N	0.032128000	-1.225607000	1.090967000
H	-0.071898000	-2.233569000	1.290685000
N	1.618027000	0.821049000	0.049968000
O	3.776371000	1.383532000	0.366531000
O	3.050278000	0.888919000	-1.734995000
S	1.098443000	-3.763764000	-0.076347000
C	1.303082000	-2.122355000	-0.823468000
H	2.293942000	-1.779912000	-0.527448000
H	1.301372000	-2.198384000	-1.909861000
Se	-2.055488000	1.100164000	0.554467000
C	5.079914000	1.668636000	-0.192715000
H	5.469711000	0.786900000	-0.702583000
H	5.008221000	2.496474000	-0.898827000
H	5.701497000	1.935088000	0.659891000
S	-3.295443000	-0.513433000	-0.248651000
C	-4.456918000	0.372010000	-1.321135000
H	-3.931911000	0.863954000	-2.139304000
H	-5.122040000	-0.397531000	-1.722365000
H	-5.033532000	1.088006000	-0.737138000
H	-0.003930000	-4.101939000	-0.771982000
H	-0.835835000	-0.690473000	1.298010000

B(C)
E= -4262.93
Nimag=0

C	0.308096000	1.697934000	0.223983000
C	1.236532000	1.265293000	-2.018556000
N	0.003796000	1.503081000	-1.226640000
C	0.951210000	0.430724000	0.765528000
N	2.104993000	0.110991000	-0.056626000
C	1.926174000	0.022930000	-1.489746000
H	1.050240000	2.497163000	0.260164000
H	1.867193000	2.147858000	-1.908998000
H	0.952696000	1.140482000	-3.063704000
H	0.618068000	-1.603544000	1.321668000
O	3.224420000	-0.586073000	1.812762000
Se	-1.123207000	-1.379683000	-0.494465000
H	-0.690536000	-0.523284000	1.809857000
H	1.348930000	-0.865873000	-1.770662000
H	2.901222000	-0.059844000	-1.961944000
C	-0.955596000	2.136695000	0.937010000
S	-1.762143000	3.540767000	0.118649000
C	0.016564000	-0.749110000	1.013821000
H	-1.702418000	1.342494000	0.930428000
H	-0.723623000	2.378486000	1.973984000
H	1.359668000	0.682607000	1.746912000
H	-0.609970000	0.655964000	-1.287463000
C	3.170185000	-0.484248000	0.604119000
O	4.108979000	-0.902036000	-0.263821000
C	5.277058000	-1.483336000	0.361401000
H	5.923842000	-1.775559000	-0.463568000
H	5.764437000	-0.744970000	0.999070000
H	4.988226000	-2.348501000	0.959011000
H	-0.811822000	4.449564000	0.411264000
S	-3.100356000	-0.707298000	0.146821000
C	-3.658002000	-2.071233000	1.200452000
H	-4.667124000	-1.792795000	1.516585000
H	-3.695261000	-2.997458000	0.628654000
H	-3.016565000	-2.173578000	2.075083000
H	-0.535199000	2.332554000	-1.522296000

TS^{B-C}(T)
E= -5546.02
Nimag=-150

C	3.875703000	1.184091000	1.115088000
H	2.862296000	0.994523000	0.767325000
C	4.106972000	2.690379000	1.202580000
H	4.348523000	3.033591000	0.196109000
C	2.876588000	3.479918000	1.656538000
H	2.202807000	3.575440000	0.804878000
H	3.196897000	4.482106000	1.940037000
C	5.123065000	0.913136000	3.275621000
H	5.966027000	0.417662000	2.796291000
H	4.980137000	0.496440000	4.273400000
C	5.315693000	2.416533000	3.323971000
H	4.509388000	2.869508000	3.903329000
H	6.263633000	2.646795000	3.799058000
C	6.412803000	3.411965000	1.313010000
N	3.900052000	0.603044000	2.490850000
H	3.797656000	-0.412004000	2.374598000
N	5.298234000	2.954124000	1.981802000
O	7.478403000	3.498704000	2.138847000
O	6.420865000	3.703961000	0.131776000
S	4.780333000	-1.335309000	0.347453000
C	4.872627000	0.473330000	0.221083000
H	5.895458000	0.737077000	0.487319000
H	4.719351000	0.788364000	-0.810179000
Se	1.822286000	2.745601000	3.164940000
C	8.665409000	4.031032000	1.513891000
H	8.970133000	3.396825000	0.680177000

H	8.475836000	5.041459000	1.149343000
H	9.421433000	4.035583000	2.297146000
S	0.442854000	0.414362000	2.199483000
C	0.210253000	1.172943000	0.573039000
H	0.583408000	0.514218000	-0.211821000
H	-0.844804000	1.380230000	0.393816000
H	0.764219000	2.106029000	0.578044000
H	3.566456000	-1.456226000	-0.221836000
H	3.050366000	1.033596000	2.964574000
S	-1.022143000	-1.153312000	1.707538000
C	-0.989662000	-1.944468000	3.330476000
H	-1.113511000	-1.201115000	4.119888000
H	-1.814732000	-2.657729000	3.359051000
H	-0.047399000	-2.476987000	3.462832000
H	-2.189100000	0.332720000	2.127194000
O	-2.679757000	1.228188000	2.332126000
H	-3.367426000	1.048354000	2.986866000
H	-1.940093000	2.121531000	2.680836000
O	-1.276845000	3.057893000	3.036488000
H	-0.255344000	2.963506000	2.952040000
H	-1.534695000	3.895866000	2.630224000

TS^{B-C}(C)
E= -5546.02
Nimag=-105

C	5.800961000	-2.304082000	1.239708000
C	6.898240000	-1.837800000	-0.911188000
N	5.609419000	-1.914701000	-0.186040000
C	6.418103000	-3.695524000	1.284418000
N	7.640632000	-3.679200000	0.498354000
C	7.572151000	-3.194817000	-0.866840000
H	6.514588000	-1.592141000	1.658728000
H	7.505077000	-1.074111000	-0.422354000
H	6.693141000	-1.543656000	-1.940834000
H	6.066026000	-5.760050000	0.902706000
O	8.633377000	-5.068450000	2.022812000
Se	4.430757000	-4.688422000	-0.721979000
H	4.767461000	-4.965140000	1.781314000
H	7.020178000	-3.895725000	-1.499053000
H	8.581089000	-3.093593000	-1.256443000
C	4.467576000	-2.198670000	1.949462000
S	3.662167000	-0.590744000	1.723513000
C	5.471087000	-4.850107000	0.956440000
H	3.771162000	-2.906666000	1.509477000
H	4.590029000	-2.423677000	3.008004000
H	6.742696000	-3.859215000	2.313609000
H	5.003873000	-2.694129000	-0.605477000
C	8.658388000	-4.496048000	0.950422000
O	9.673765000	-4.552336000	0.062155000
C	10.795221000	-5.346773000	0.501293000
H	11.513886000	-5.300659000	-0.315132000
H	11.217049000	-4.928616000	1.416417000
H	10.478741000	-6.374354000	0.685239000
H	4.535986000	0.116285000	2.466361000
S	1.774491000	-3.532127000	-0.172350000
C	1.699659000	-4.406673000	1.409888000
H	1.548326000	-3.702330000	2.227828000
H	0.891169000	-5.138152000	1.405378000
H	2.649065000	-4.922404000	1.524345000
H	5.078079000	-1.034476000	-0.187101000
S	-0.347693000	-3.081726000	-0.092069000
C	-0.426047000	-2.248912000	-1.691567000
H	0.015328000	-2.871785000	-2.471373000
H	-1.480928000	-2.073899000	-1.908475000
H	0.095620000	-1.293193000	-1.632950000
H	-0.377163000	-4.966630000	-0.673916000
O	-0.222880000	-5.958589000	-0.905845000
H	-0.734070000	-6.157318000	-1.701174000
H	0.980756000	-6.270928000	-1.014557000
O	2.076459000	-6.657339000	-1.136293000
H	2.834179000	-6.000662000	-0.875036000
H	2.232179000	-7.494367000	-0.678536000

C(SeST)
E= -3786.33
Nimag=0

C	0.632700000	-1.351300000	0.153000000
C	-0.193100000	-0.924300000	-2.124700000
N	0.973900000	-0.844400000	-1.211500000
C	-0.449000000	-0.467400000	0.755600000
N	-1.560000000	-0.380800000	-0.176100000
C	-1.308800000	-0.065000000	-1.567600000
H	0.211200000	-2.345300000	-0.004100000
H	-0.484400000	-1.973400000	-2.182400000
H	0.115600000	-0.572700000	-3.109300000
H	-0.851000000	1.446500000	1.631800000
O	-2.996000000	-0.312700000	1.602800000
Se	1.137300000	2.041800000	0.186800000
H	0.633800000	0.705100000	2.203700000
H	-1.056700000	0.990900000	-1.702600000
H	-2.212500000	-0.261900000	-2.138300000
C	1.909300000	-1.467200000	0.965000000
S	3.188300000	-2.429200000	0.106800000
C	0.016500000	0.870200000	1.320500000
H	2.360900000	-0.489400000	1.131100000
H	1.686900000	-1.904200000	1.938100000
H	-0.843200000	-1.009500000	1.618700000
H	1.261800000	0.155500000	-1.085600000
C	-2.811100000	-0.226400000	0.406000000
O	-3.754200000	0.012400000	-0.522000000
C	-5.094700000	0.129100000	0.011700000
H	-5.382500000	-0.802000000	0.501100000
H	-5.139500000	0.948600000	0.729600000
H	-5.726400000	0.329100000	-0.851500000
H	1.788400000	-1.394300000	-1.527300000
H	0.009300000	2.608400000	-0.564500000
H	2.552200000	-3.613700000	0.186700000

C (SeSC)
E= -3786.08
Nimag=0

C	0.632700000	-1.351300000	0.153000000
C	-0.193100000	-0.924300000	-2.124700000
N	0.973900000	-0.844400000	-1.211500000
C	-0.449000000	-0.467400000	0.755600000
N	-1.560000000	-0.380800000	-0.176100000
C	-1.308800000	-0.065000000	-1.567600000
H	0.211200000	-2.345300000	-0.004100000
H	-0.484400000	-1.973400000	-2.182400000
H	0.115600000	-0.572700000	-3.109300000
H	-0.851000000	1.446500000	1.631800000
O	-2.996000000	-0.312700000	1.602800000
Se	1.137300000	2.041800000	0.186800000
H	0.633800000	0.705100000	2.203700000
H	-1.056700000	0.990900000	-1.702600000
H	-2.212500000	-0.261900000	-2.138300000
C	1.909300000	-1.467200000	0.965000000
S	3.188300000	-2.429200000	0.106800000
C	0.016500000	0.870200000	1.320500000
H	2.360900000	-0.489400000	1.131100000
H	1.686900000	-1.904200000	1.938100000
H	-0.843200000	-1.009500000	1.618700000
H	1.261800000	0.155500000	-1.085600000
C	-2.811100000	-0.226400000	0.406000000
O	-3.754200000	0.012400000	-0.522000000
C	-5.094700000	0.129100000	0.011700000

H	-5.382500000	-0.802000000	0.501100000
H	-5.139500000	0.948600000	0.729600000
H	-5.726400000	0.329100000	-0.851500000
H	1.788400000	-1.394300000	-1.527300000
H	0.009300000	2.608400000	-0.564500000
H	2.552200000	-3.613700000	0.186700000

MeSSMe
E= -1095.48
Nimag=0

S	1.681988000	-3.577356000	-0.023951000
C	1.515590000	-5.241657000	-0.726597000
H	0.843029000	-5.841788000	-0.113456000
H	1.153540000	-5.191681000	-1.754113000
H	2.517855000	-5.679156000	-0.715650000
S	-0.234217000	-2.902867000	-0.002409000
C	-0.455536000	-2.263152000	-1.685567000
H	-0.386456000	-3.069379000	-2.416735000
H	-1.458794000	-1.828972000	-1.717351000
H	0.287585000	-1.493368000	-1.894471000

MeSH
E= -626.90
Nimag=0

S	0.321682000	-4.734858000	-4.069697000
C	-0.612326000	-4.041416000	-5.464493000
H	-0.368391000	-2.989481000	-5.617803000
H	-0.303731000	-4.607933000	-6.344754000
H	-1.686128000	-4.167914000	-5.321256000
H	-0.190755000	-3.921529000	-3.128544000

C’ (SeST)
E= -4940.67

C	-2.442900000	-1.121700000	-0.575500000
H	-3.379800000	-1.221000000	-1.127000000
C	-1.748700000	0.178300000	-0.973800000
H	-1.219500000	-0.021700000	-1.907400000
C	-2.694500000	1.341800000	-1.264400000
H	-3.154400000	1.205300000	-2.241100000
H	-2.121300000	2.267400000	-1.283600000
C	-1.764000000	-0.689200000	1.811900000
H	-1.045600000	-1.506900000	1.813300000
H	-2.211900000	-0.601500000	2.802400000
C	-1.120600000	0.606700000	1.360700000
H	-1.824500000	1.434500000	1.469900000
H	-0.258600000	0.819900000	1.984800000
C	0.598100000	0.262800000	-0.416500000
N	-2.857200000	-1.051600000	0.864800000
H	-3.211400000	-1.995700000	1.091400000
N	-0.708300000	0.497100000	-0.019600000
O	1.445300000	0.430300000	0.630200000
O	0.910900000	-0.045000000	-1.544400000
S	-2.249000000	-3.844100000	-0.009700000
C	-1.582300000	-2.353500000	-0.800900000
H	-0.586100000	-2.213700000	-0.384200000
H	-1.459100000	-2.513500000	-1.871000000
Se	-4.213100000	1.679200000	-0.030500000
H	-3.282700000	-4.010500000	-0.856700000
H	-5.209800000	1.028100000	-0.894100000
C	2.821300000	0.288900000	0.360600000
C	3.494400000	-0.747700000	0.984100000
C	3.475600000	1.204800000	-0.446500000
C	4.866000000	-0.876500000	0.796100000
C	4.844900000	1.076000000	-0.638900000
C	5.533100000	0.035400000	-0.017700000
H	2.957300000	-1.446300000	1.615600000
H	2.925500000	2.004900000	-0.926000000
H	5.415300000	-1.677500000	1.274500000
H	5.379800000	1.777100000	-1.267100000
Cl	7.236000000	-0.124100000	-0.259000000
H	-3.604100000	-0.324400000	0.909600000

C’ (SeSC)
E= -4940.24
Nimag=0

C	2.547970000	-1.444315000	0.030968000
C	1.927309000	-0.438729000	-2.132444000
N	3.011329000	-0.634510000	-1.136880000
C	1.420956000	-0.703549000	0.734362000
N	0.390834000	-0.387320000	-0.241105000
C	0.773849000	0.283885000	-1.466937000
H	2.137429000	-2.361222000	-0.394678000
H	1.629195000	-1.426855000	-2.483911000
H	2.326668000	0.143937000	-2.962719000
H	0.916808000	0.953734000	1.975970000
O	-1.197199000	-0.730614000	1.367633000
Se	2.921275000	1.907567000	0.790086000
H	2.383562000	0.129112000	2.484217000
H	1.045782000	1.330108000	-1.288618000
H	-0.073197000	0.280809000	-2.147463000
C	3.744731000	-1.778589000	0.900775000
S	5.101044000	-2.529558000	-0.044383000
C	1.824192000	0.472625000	1.617294000
H	4.171109000	-0.875247000	1.335741000
H	3.435006000	-2.433269000	1.714982000
H	0.958234000	-1.419589000	1.417573000
H	3.841826000	-1.117509000	-1.513215000
C	-0.903920000	-0.351140000	0.257387000
O	-1.752258000	0.152145000	-0.673022000
H	4.468962000	-3.690438000	-0.303273000
H	4.125727000	1.611520000	1.578929000
C	-3.119928000	0.190927000	-0.336063000
C	-3.834559000	-0.982326000	-0.158950000
C	-3.725611000	1.433758000	-0.264064000
C	-5.197114000	-0.909229000	0.099941000
C	-5.090277000	1.509142000	-0.008870000
C	-5.818015000	0.336146000	0.173319000
H	-3.336416000	-1.942267000	-0.213778000
H	-3.140130000	2.334423000	-0.409183000
H	-5.778461000	-1.811086000	0.244927000
H	-5.587335000	2.469200000	0.050431000
Cl	-7.513802000	0.424752000	0.492517000
H	3.299626000	0.296063000	-0.748591000

TS1 (SeST)
E= -4909.13
Nimag=-591

C	-1.958264000	-0.826508000	-2.126488000
H	-2.040096000	-0.048810000	-2.887519000
C	-1.333353000	-0.218416000	-0.882780000
H	-0.273304000	-0.082367000	-1.087648000
C	-1.942320000	1.147351000	-0.529585000
H	-3.031051000	1.153090000	-0.639586000
H	-1.539325000	1.876892000	-1.233212000
C	-3.490030000	-2.131450000	-0.639114000
H	-3.038692000	-3.080700000	-0.920455000
H	-4.551508000	-2.286052000	-0.445784000
C	-2.801680000	-1.519818000	0.554172000
H	-3.304251000	-0.610092000	0.912167000
H	-2.807102000	-2.245205000	1.363226000
C	-0.576872000	-0.937275000	1.255535000
N	-3.373355000	-1.238524000	-1.831394000
H	-3.670939000	-1.762673000	-2.678896000
N	-1.427137000	-1.178824000	0.215077000
O	-0.784059000	-1.701210000	2.311155000

O	0.558635000	-0.322032000	1.077745000
S	-2.161512000	-2.924748000	-3.952934000
C	-1.208669000	-2.019991000	-2.698774000
H	-0.975873000	-2.741635000	-1.916588000
H	-0.264430000	-1.674126000	-3.117858000
Se	-1.516481000	1.683190000	1.296434000
H	-2.080892000	-1.979579000	-4.909437000
H	0.204135000	0.692903000	1.056882000
C	-0.026438000	-1.425974000	3.486736000
C	0.989405000	-2.297359000	3.828791000
C	-0.383211000	-0.343061000	4.268089000
C	1.686299000	-2.072400000	5.010818000
C	0.316543000	-0.118298000	5.447314000
C	1.347232000	-0.983238000	5.811495000
H	1.237356000	-3.134972000	3.187618000
H	-1.184478000	0.314893000	3.958076000
H	2.489722000	-2.733872000	5.309309000
H	0.065959000	0.721946000	6.082612000
Cl	2.214960000	-0.701677000	7.276381000
H	-3.947746000	-0.405382000	-1.707532000

TS1 (SeSC)
E= -4908.87
Nimag=-530

C	2.214434000	-1.956194000	1.189054000
C	3.379592000	-2.132619000	-0.984240000
N	3.536034000	-1.934121000	0.483808000
C	1.359848000	-0.824932000	0.636761000
N	1.199950000	-1.057595000	-0.801060000
C	2.458862000	-1.069211000	-1.534073000
H	1.769800000	-2.915974000	0.922414000
H	2.969111000	-3.132878000	-1.121428000
H	4.365521000	-2.072800000	-1.445407000
H	1.643261000	0.810298000	1.986271000
O	-0.897950000	-0.053857000	-0.688518000
Se	1.152429000	1.943887000	-0.218937000
H	2.993683000	0.632428000	0.875012000
H	2.901433000	-0.063913000	-1.470222000
H	2.266493000	-1.283961000	-2.581618000
C	2.464961000	-1.901716000	2.684417000
S	3.665895000	-3.152760000	3.225188000
C	1.902624000	0.576686000	0.954601000
H	2.894650000	-0.941436000	2.972647000
H	1.521280000	-2.017159000	3.216975000
H	0.369563000	-0.923259000	1.076772000
H	4.117347000	-2.651310000	0.953447000
C	0.159001000	-0.389764000	-1.376069000
O	0.118021000	-0.479839000	-2.690802000
H	2.870505000	-4.218305000	3.009043000
H	-0.527822000	0.825847000	-0.211104000
C	-0.879142000	0.266018000	-3.383314000
C	-1.897124000	-0.435137000	-4.000467000
C	-0.750519000	1.639361000	-3.473194000
C	-2.836310000	0.273206000	-4.741686000
C	-1.692163000	2.345454000	-4.211628000
C	-2.729335000	1.659086000	-4.841209000
H	-1.960796000	-1.512769000	-3.906067000
H	0.064020000	2.146512000	-2.973296000
H	-3.648268000	-0.243647000	-5.237382000
H	-1.623609000	3.422331000	-4.301481000
Cl	-3.896171000	2.537872000	-5.759975000
H	3.969163000	-1.024560000	0.663178000

INT (SeSC)
E= -4924.13
Nimag=0

C	2.276736000	-1.976991000	1.196786000
C	3.660711000	-2.082120000	-0.847510000
N	3.640010000	-1.770120000	0.611930000
C	1.284993000	-1.098323000	0.460411000
N	1.334693000	-1.404634000	-0.967453000
C	2.656073000	-1.195745000	-1.540189000
H	2.040904000	-3.020629000	0.985607000
H	3.402805000	-3.136386000	-0.944434000
H	4.671114000	-1.908616000	-1.217430000
H	0.956505000	0.703041000	1.636714000
O	-0.905203000	-1.086730000	-1.171459000
Se	0.593540000	1.306540000	-0.813599000
H	2.485106000	0.736563000	0.745944000
H	2.960727000	-0.140220000	-1.476089000
H	2.627745000	-1.461057000	-2.594141000
C	2.350271000	-1.755748000	2.695073000
S	3.685241000	-2.713231000	3.471195000
C	1.442461000	0.410523000	0.707889000
H	2.568804000	-0.711548000	2.925937000
H	1.389284000	-2.000902000	3.146571000
H	0.293986000	-1.391901000	0.802007000
H	4.283015000	-2.343482000	1.183974000
C	0.322635000	-0.605034000	-1.588087000
O	0.510297000	-0.614886000	-2.958288000
H	3.115532000	-3.920720000	3.292918000
H	-1.533260000	-0.353387000	-1.222894000
C	-0.315681000	0.264018000	-3.663511000
C	-1.622871000	-0.094466000	-3.971546000
C	0.214079000	1.479364000	-4.079199000
C	-2.420873000	0.789485000	-4.688183000
C	-0.582488000	2.364753000	-4.795583000
C	-1.899213000	2.018095000	-5.089282000
H	-2.002645000	-1.061892000	-3.668671000
H	1.241475000	1.726801000	-3.844418000
H	-3.440391000	0.527954000	-4.943123000
H	-0.187305000	3.316064000	-5.129502000
Cl	-2.893760000	3.118136000	-5.974063000
H	3.905502000	-0.793044000	0.752170000

INT (SeST)
E= -4924.73
Nimag=0

C	-1.972722000	-0.817099000	-2.122657000
H	-1.989961000	0.053156000	-2.780786000
C	-1.149232000	-0.503122000	-0.889272000
H	-0.104440000	-0.553156000	-1.191388000
C	-1.400160000	0.894689000	-0.304602000
H	-2.458660000	1.162946000	-0.280718000
H	-0.860207000	1.650177000	-0.873910000
C	-3.563127000	-2.053217000	-0.638513000
H	-3.247925000	-3.014624000	-1.038330000
H	-4.622667000	-2.096691000	-0.387122000
C	-2.723594000	-1.643589000	0.541637000
H	-3.089361000	-0.706037000	0.984046000
H	-2.803221000	-2.414508000	1.304114000
C	-0.449330000	-1.200896000	1.201832000
N	-3.406802000	-1.060674000	-1.746972000
H	-3.835506000	-1.444892000	-2.614039000
N	-1.333694000	-1.534389000	0.125637000
O	-0.788988000	-1.949845000	2.313432000
O	0.846364000	-1.395695000	0.753913000
S	-2.636539000	-2.646190000	-4.126752000
C	-1.459465000	-2.027895000	-2.892115000
H	-1.266133000	-2.854358000	-2.209465000
H	-0.515435000	-1.766939000	-3.368476000
Se	-0.747842000	0.827977000	1.542357000
H	-2.499248000	-1.621846000	-4.990857000
H	1.413730000	-0.786988000	1.246090000
C	-0.113486000	-1.596394000	3.484056000
C	1.174318000	-2.062685000	3.720913000

C	-0.776201000	-0.808045000	4.416529000
C	1.817803000	-1.715895000	4.902981000
C	-0.134549000	-0.460467000	5.599298000
C	1.162687000	-0.910677000	5.832918000
H	1.657738000	-2.704354000	2.995470000
H	-1.785666000	-0.472248000	4.216387000
H	2.819058000	-2.072907000	5.109953000
H	-0.635200000	0.151304000	6.339384000
Cl	1.964116000	-0.478358000	7.300275000
H	-3.852702000	-0.180384000	-1.493519000

TS2(SeSC)
E= -4912.22
Nimag=-185

C	2.163448000	0.094236000	1.156326000
C	3.593786000	-0.590785000	-0.781902000
N	3.067910000	0.525789000	0.061285000
C	0.884418000	-0.484713000	0.536642000
N	1.256798000	-1.225107000	-0.670035000
C	2.600853000	-1.748958000	-0.762270000
H	2.694100000	-0.694341000	1.691102000
H	4.563379000	-0.900046000	-0.392586000
H	3.684074000	-0.192648000	-1.789836000
H	-0.805341000	0.809155000	0.970976000
O	0.645035000	-1.805750000	-2.816650000
Se	-1.153837000	-0.220281000	-1.322017000
H	0.311800000	1.474897000	-0.256408000
H	2.687242000	-2.328885000	-1.677397000
H	2.770142000	-2.412595000	0.087738000
C	1.935411000	1.264326000	2.099870000
S	3.491850000	2.013177000	2.654842000
C	-0.137450000	0.570137000	0.147696000
H	1.404321000	2.070541000	1.593983000
H	1.336828000	0.943581000	2.952184000
H	0.441964000	-1.184710000	1.253804000
H	3.825407000	1.049648000	0.527177000
C	0.448995000	-1.105368000	-1.722689000
O	1.575461000	0.467845000	-3.029555000
H	3.882515000	0.980262000	3.426320000
H	0.854183000	-1.074793000	-3.467937000
C	1.391762000	1.679835000	-2.614898000
C	0.136095000	2.345595000	-2.686006000
C	2.438250000	2.406558000	-1.969376000
C	-0.104165000	3.513385000	-1.984273000
C	2.197802000	3.592998000	-1.277029000
C	0.908890000	4.107721000	-1.224967000
H	-0.643029000	1.912669000	-3.299028000
H	3.461767000	2.079530000	-2.132650000
H	-1.081622000	3.981530000	-2.019327000
H	3.009244000	4.125601000	-0.792992000
Cl	0.574170000	5.524863000	-0.286166000
H	2.598674000	1.234602000	-0.578028000

TS2(SeST)
E=-4917.14
Nimag=-84

C	-1.620670000	-0.236438000	-0.461963000
H	-1.512125000	0.321863000	0.463331000
C	-0.533098000	-1.312314000	-0.522680000
H	-0.444153000	-1.662709000	-1.555558000
C	0.821082000	-0.756464000	-0.071208000
H	0.754264000	0.306429000	0.150989000
H	1.589242000	-0.923790000	-0.822434000
C	-3.017143000	-2.258697000	-0.919572000
H	-2.687586000	-2.201281000	-1.955520000
H	-4.050516000	-2.604082000	-0.902512000
C	-2.130297000	-3.164649000	-0.078110000
H	-2.635173000	-3.448834000	0.844384000
H	-1.901429000	-4.073895000	-0.642406000
C	-0.296615000	-2.697650000	1.463416000
N	-2.965536000	-0.892956000	-0.353613000
H	-3.620330000	-0.229115000	-0.815471000
N	-0.918727000	-2.446624000	0.287253000
O	-1.376972000	-2.117349000	2.906831000
O	-0.331129000	-3.982633000	1.843551000
S	-3.102959000	1.726541000	-1.764262000
C	-1.605732000	0.708114000	-1.652612000
H	-1.570583000	0.154028000	-2.593953000
H	-0.716918000	1.336299000	-1.605450000
Se	1.350145000	-1.698144000	1.563988000
H	-2.966324000	2.332423000	-0.568295000
H	-0.387175000	-4.000611000	2.813028000
C	-1.906603000	-0.917573000	2.777494000
C	-1.144621000	0.270194000	2.854338000
C	-3.301201000	-0.776395000	2.573244000
C	-1.722693000	1.511898000	2.641355000
C	-3.887884000	0.473570000	2.384246000
C	-3.086653000	1.611157000	2.364795000
H	-0.093192000	0.195799000	3.092475000
H	-3.920782000	-1.667067000	2.628774000
H	-1.123277000	2.413619000	2.695696000
H	-4.958836000	0.566099000	2.243307000
Cl	-3.786094000	3.158262000	2.021279000
H	-3.203474000	-0.909016000	0.661521000

P (SeSC)
E= -3086.33
Nimag=0

C	1.412716000	0.189803000	0.996522000
C	1.897145000	-1.906955000	-0.337186000
N	2.385548000	-0.920774000	0.682500000
C	0.067415000	-0.441027000	1.381515000
N	-0.355448000	-1.374616000	0.372985000
C	0.553537000	-2.459471000	0.089991000
H	1.833801000	0.698591000	1.866581000
H	2.661062000	-2.679677000	-0.430477000
H	1.814758000	-1.368777000	-1.278853000
H	-1.743336000	0.201777000	2.361069000
O	-1.367347000	-1.473741000	-1.688983000
Se	-1.994814000	0.763747000	-0.086983000
H	-0.654157000	1.539245000	1.934277000
H	0.135686000	-3.057521000	-0.719023000
H	0.653853000	-3.088613000	0.980053000
C	1.403518000	1.146656000	-0.185995000
S	3.079920000	1.544706000	-0.770972000
C	-1.043976000	0.574245000	1.615111000
H	0.885409000	0.720343000	-1.041630000
H	0.873750000	2.055886000	0.083303000
H	0.234118000	-0.997530000	2.311913000
H	2.651782000	-1.405602000	1.539229000
C	-1.188477000	-0.914938000	-0.635618000
H	3.438131000	2.283678000	0.297105000
H	3.211138000	-0.413503000	0.292564000

P (SeST)
E= -3086.69
Nimag=0

C	1.356278000	-0.077745000	-0.166281000
H	0.940014000	-0.263476000	0.824314000
C	0.195356000	0.088746000	-1.150728000
H	0.600760000	0.338232000	-2.138629000
C	-0.690517000	-1.138357000	-1.259003000
H	-0.129498000	-2.068551000	-1.251701000
H	-1.295697000	-1.081500000	-2.162270000
C	1.181301000	2.367408000	0.387440000
H	1.784970000	3.275289000	0.419343000

H	0.835725000	2.114761000	1.388962000
C	0.013757000	2.470778000	-0.577243000
H	-0.709057000	3.189536000	-0.194581000
H	0.351527000	2.799255000	-1.564534000
C	-1.578859000	0.866723000	0.279672000
N	2.060072000	1.247029000	-0.068243000
H	2.876258000	1.070591000	0.548195000
N	-0.637017000	1.184570000	-0.698804000
O	-2.098269000	1.666853000	1.016068000
S	3.858164000	-1.042271000	0.568076000
C	2.391194000	-1.132887000	-0.505615000
H	2.763656000	-1.005203000	-1.525884000
H	1.946928000	-2.123097000	-0.436038000
Se	-1.875460000	-1.052283000	0.296355000
H	3.239228000	-1.481570000	1.681065000
H	2.446632000	1.477012000	-0.987538000

C’(A3*)
E=-4516.29
Nimag=0

C	2.535000000	-3.187900000	-0.530000000
C	3.348000000	-1.904700000	-0.524200000
C	2.539800000	-0.690000000	-0.082000000
C	3.403400000	0.559100000	-0.040500000
H	3.153400000	-4.017300000	-0.879500000
H	1.683200000	-3.100100000	-1.205100000
H	3.758500000	-1.740100000	-1.526600000
H	4.195100000	-2.012500000	0.156800000
H	2.174200000	-0.881900000	0.923200000
H	3.815300000	0.801400000	-1.021400000
H	4.231700000	0.407000000	0.650100000
S	1.936100000	-3.710300000	1.105300000
Se	2.417000000	2.171300000	0.496200000
N	1.349600000	-0.507800000	-0.898700000
C	1.511600000	-0.060900000	-2.267200000
H	2.480400000	-0.401200000	-2.628000000
H	0.736200000	-0.487800000	-2.899800000
H	1.468300000	1.030000000	-2.336200000
C	0.150100000	-0.470300000	-0.264800000
O	-0.053900000	-0.742500000	0.903500000
O	-0.843000000	-0.113400000	-1.153800000
H	1.775600000	1.503500000	1.638300000
C	-2.122800000	0.053600000	-0.642600000
C	-2.385900000	0.987800000	0.351100000
C	-3.147100000	-0.684300000	-1.218300000
C	-3.693700000	1.185100000	0.774200000
C	-4.459000000	-0.485500000	-0.802300000
C	-4.722500000	0.448100000	0.193900000
H	-1.573000000	1.548700000	0.792600000
H	-2.915600000	-1.409500000	-1.990100000
H	-3.918200000	1.906800000	1.549800000
H	-5.270000000	-1.051900000	-1.242900000
Cl	-6.358400000	0.699900000	0.720300000
H	1.011300000	-2.741800000	1.238800000

TS1(A3*)
E= -4489.48
Nimag=-331

C	0.155856000	-2.772622000	-3.399373000
C	1.438847000	-2.860986000	-2.589444000
C	1.466094000	-4.016384000	-1.605771000
C	2.719232000	-4.046408000	-0.733832000
H	0.226310000	-1.956176000	-4.121623000
H	-0.700671000	-2.572182000	-2.755558000
H	1.604718000	-1.909042000	-2.075999000
H	2.286787000	-3.005038000	-3.262493000
H	1.432253000	-4.934875000	-2.184661000
H	2.886416000	-3.079156000	-0.257033000
H	3.570683000	-4.261968000	-1.379845000
S	-0.210870000	-4.241548000	-4.406382000
Se	2.602637000	-5.423462000	0.653612000
N	0.263881000	-4.040528000	-0.759602000
C	0.050739000	-2.880995000	0.096962000
H	-0.990603000	-2.832070000	0.404596000
H	0.692873000	-2.968270000	0.981049000
H	0.304857000	-1.985959000	-0.461005000
C	-0.036114000	-5.241284000	-0.238144000
O	0.261793000	-6.374204000	-0.843960000
O	-1.035126000	-5.222989000	0.648711000
H	-0.514004000	-5.053250000	-3.378868000
H	1.272960000	-6.450830000	-0.593886000
C	-1.358075000	-6.391337000	1.352726000
C	-2.702917000	-6.715476000	1.431940000
C	-0.386474000	-7.130761000	2.007827000
C	-3.091672000	-7.816721000	2.186185000
C	-0.774700000	-8.233364000	2.757662000
C	-2.122980000	-8.570029000	2.842262000
H	-3.436484000	-6.113104000	0.908645000
H	0.652333000	-6.841331000	1.928453000
H	-4.136299000	-8.091249000	2.262058000
H	-0.034558000	-8.829038000	3.277543000
Cl	-2.603514000	-9.947495000	3.781313000

INT (A3*)
E=-4499.75
Nimag=0

C	3.506575000	-2.767473000	-1.111119000
C	3.620388000	-1.302729000	-0.718046000
C	2.363628000	-0.765401000	-0.064745000
C	2.440248000	0.728227000	0.273249000
H	4.461437000	-3.126949000	-1.500849000
H	2.752928000	-2.902592000	-1.885797000
H	3.881400000	-0.714710000	-1.603229000
H	4.436213000	-1.178947000	-0.001668000
H	2.232962000	-1.303620000	0.870708000
H	2.796350000	1.318893000	-0.570605000
H	3.084627000	0.898313000	1.134869000
S	3.105800000	-3.887957000	0.263468000
Se	0.608016000	1.280255000	0.694433000
N	1.147707000	-1.028420000	-0.838740000
C	1.150881000	-0.440348000	-2.171937000
H	1.112598000	0.655844000	-2.135615000
H	2.054546000	-0.753072000	-2.688776000
H	0.287813000	-0.800159000	-2.726008000
C	0.063455000	-0.607948000	-0.036421000
O	-0.080185000	-1.449729000	1.067059000
O	-1.092709000	-0.548577000	-0.809997000
H	-0.111059000	-0.869113000	1.839164000
C	-2.189764000	0.035232000	-0.201557000
C	-2.510871000	1.346208000	-0.536832000
C	-2.983907000	-0.686277000	0.683682000
C	-3.630383000	1.949507000	0.023786000
C	-4.103926000	-0.086609000	1.247890000
C	-4.416477000	1.228809000	0.917126000
H	-1.876536000	1.885914000	-1.227983000
H	-2.722170000	-1.708752000	0.920670000
H	-3.892822000	2.969908000	-0.226822000
H	-4.734544000	-0.634260000	1.937479000
Cl	-5.814808000	1.978972000	1.622574000
H	1.826831000	-3.490535000	0.384375000

TS2(A3*)
E=-4487.05
Nimag=-239

C	2.306328000	-3.164548000	-2.242097000
C	2.384902000	-4.243081000	-1.171073000
C	1.391373000	-5.386354000	-1.287493000
C	1.927018000	-6.620874000	-0.589616000
H	3.096004000	-2.431840000	-2.061589000
H	1.362983000	-2.624079000	-2.234720000
H	2.299733000	-3.806451000	-0.174997000
H	3.379317000	-4.690503000	-1.224605000
H	1.224211000	-5.619910000	-2.345243000
H	2.345161000	-6.387594000	0.386534000
H	2.648894000	-7.139833000	-1.216028000
S	2.617903000	-3.751345000	-3.937547000
Se	0.367153000	-7.758738000	-0.294134000
N	0.069653000	-5.107998000	-0.700188000
C	-0.542001000	-3.799482000	-0.773083000
H	-1.455289000	-3.819690000	-0.186748000
H	0.138167000	-3.059979000	-0.357275000
H	-0.775134000	-3.544962000	-1.811252000
C	-0.598720000	-6.149809000	-0.232143000
O	-1.905868000	-6.111912000	-0.012742000
O	-0.662620000	-5.795905000	2.068150000
H	1.385978000	-4.235576000	-4.170259000
H	-1.893906000	-6.138889000	0.987361000
C	0.588867000	-5.732125000	2.387179000
C	1.335728000	-6.877639000	2.777544000
C	1.319486000	-4.516792000	2.281385000
C	2.709064000	-6.827649000	2.960294000
C	2.690545000	-4.462497000	2.475475000
C	3.390296000	-5.624811000	2.786559000
H	0.801963000	-7.811246000	2.910720000
H	0.767879000	-3.615203000	2.038145000
H	3.262007000	-7.719956000	3.232617000
H	3.228399000	-3.525505000	2.379122000
Cl	5.124478000	-5.569401000	2.975106000

P (A3*)
E=-2666.87
Nimag=0

C	2.649380000	1.014815000	-0.765125000
C	1.447706000	0.096522000	-0.895069000
C	0.730953000	-0.181122000	0.424434000
C	-0.055460000	1.017172000	0.932327000
H	2.377906000	1.961273000	-0.292981000
H	3.057030000	1.253616000	-1.748497000
H	1.774585000	-0.850928000	-1.327019000
H	0.727379000	0.540598000	-1.588593000
H	1.477142000	-0.464262000	1.170163000
H	0.438685000	1.957843000	0.702824000
H	-0.240436000	0.946702000	2.003543000
S	3.990994000	0.363669000	0.281778000
Se	-1.773622000	0.934258000	0.000300000
N	-0.209361000	-1.280127000	0.295783000
C	0.284577000	-2.636555000	0.229649000
H	0.848655000	-2.819919000	-0.692062000
H	0.935545000	-2.827793000	1.086030000
H	-0.570024000	-3.310047000	0.252725000
C	-1.478428000	-1.004665000	-0.119078000
O	-2.312870000	-1.802403000	-0.487819000
H	4.278407000	-0.689310000	-0.505261000

Cl-PhOH
E= -1849.49
Nimag=0

O	-4.515772000	-0.195535000	2.389299000
C	-5.517985000	0.600671000	1.908554000
C	-5.184434000	1.545079000	0.937857000
C	-6.836773000	0.499128000	2.350412000
C	-6.161862000	2.379886000	0.414908000
C	-7.817578000	1.334359000	1.827962000
C	-7.475903000	2.272653000	0.861280000
H	-4.156918000	1.617389000	0.599754000
H	-7.103433000	-0.234544000	3.106395000
H	-5.907350000	3.114818000	-0.339094000
H	-8.842579000	1.257512000	2.169644000
Cl	-8.698178000	3.320012000	0.205701000
H	-4.893813000	-0.792350000	3.047310000

8.2 X-Ray crystallography

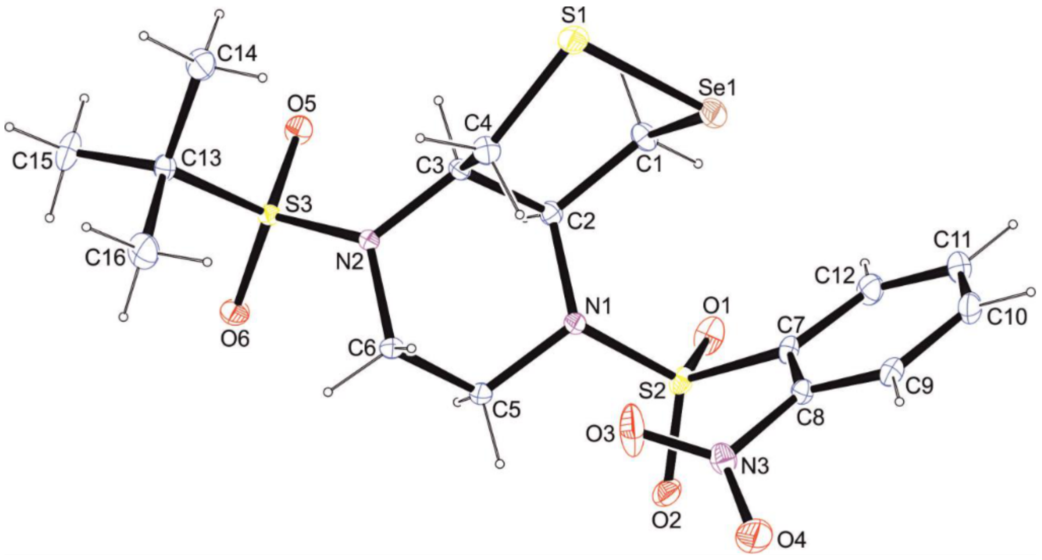

compound number	7
CCDC number	2423042
net formula	$C_{16}H_{23}N_3O_6S_3Se$
M_r/g mol^{-1}	528.51
crystal size/mm	0.130 × 0.070 × 0.020
T/K	173.(2)
radiation	MoKα
diffractometer	'Bruker D8 Venture TXS'
crystal system	orthorhombic
space group	'P b c a'
a/Å	12.0452(8)
b/Å	12.0210(8)
c/Å	28.5679(17)
α/°	90
β/°	90
γ/°	90
V/Å^3	4136.5(5)
Z	8
calc. density/g cm^{-3}	1.697
μ/mm^{-1}	2.158
absorption correction	Multi-Scan
transmission factor range	0.85–0.96
refls. measured	69488
R_{int}	0.0536
mean σ(*I*)/*I*	0.0233
θ range	3.211–27.480
observed refls.	4274
x, y (weighting scheme)	0.0579, 16.7050
hydrogen refinement	constr
Flack parameter	?
refls in refinement	4730
parameters	265
restraints	0
$R(F_{obs})$	0.0487
$R_w(F^2)$	0.1342
S	1.072
shift/error$_{max}$	0.001
max electron density/e Å^{-3}	3.557
min electron density/e Å^{-3}	−1.165

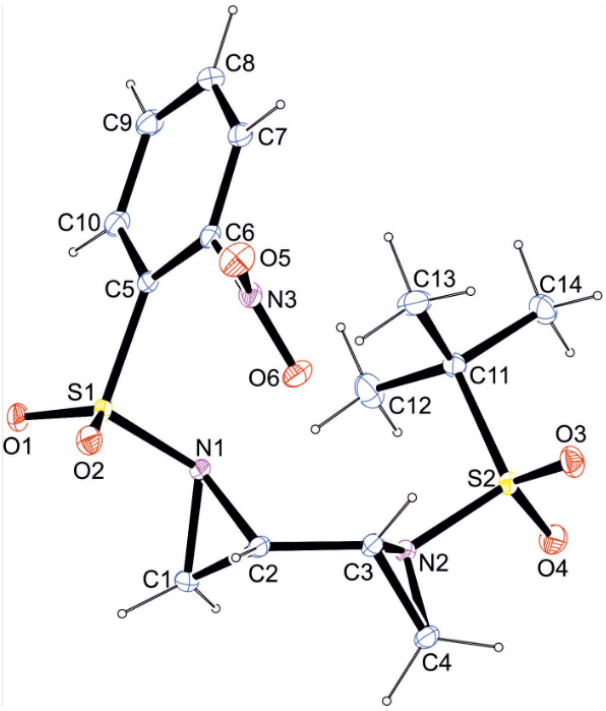

compound number	5
CCDC number	2423041
net formula	$C_{14}H_{19}N_3O_6S_2$
M_r/g mol^{-1}	389.44
crystal size/mm	0.200 × 0.190 × 0.150
T/K	173.(2)
radiation	MoKα
diffractometer	'Bruker D8 Venture TXS'
crystal system	monoclinic
space group	'P 1 21/n 1'
a/Å	12.8193(10)
b/Å	7.6755(5)
c/Å	18.2018(13)
α/°	90
β/°	105.580(2)
γ/°	90
V/Å^3	1725.2(2)
Z	4
calc. density/g cm^{-3}	1.499
μ/mm^{-1}	0.346
absorption correction	Multi-Scan
transmission factor range	0.90–0.95
refls. measured	29124
R_{int}	0.0442
mean σ(*I*)/*I*	0.0273
θ range	3.125–27.477
observed refls.	3375
x, y (weighting scheme)	0.0369, 1.1236
hydrogen refinement	constr
Flack parameter	?
refls in refinement	3941
parameters	229
restraints	0
$R(F_{obs})$	0.0327
$R_w(F^2)$	0.0848
S	1.032
shift/error$_{max}$	0.001
max electron density/e Å^{-3}	0.396
min electron density/e Å^{-3}	−0.456

8.3 NMR spectra

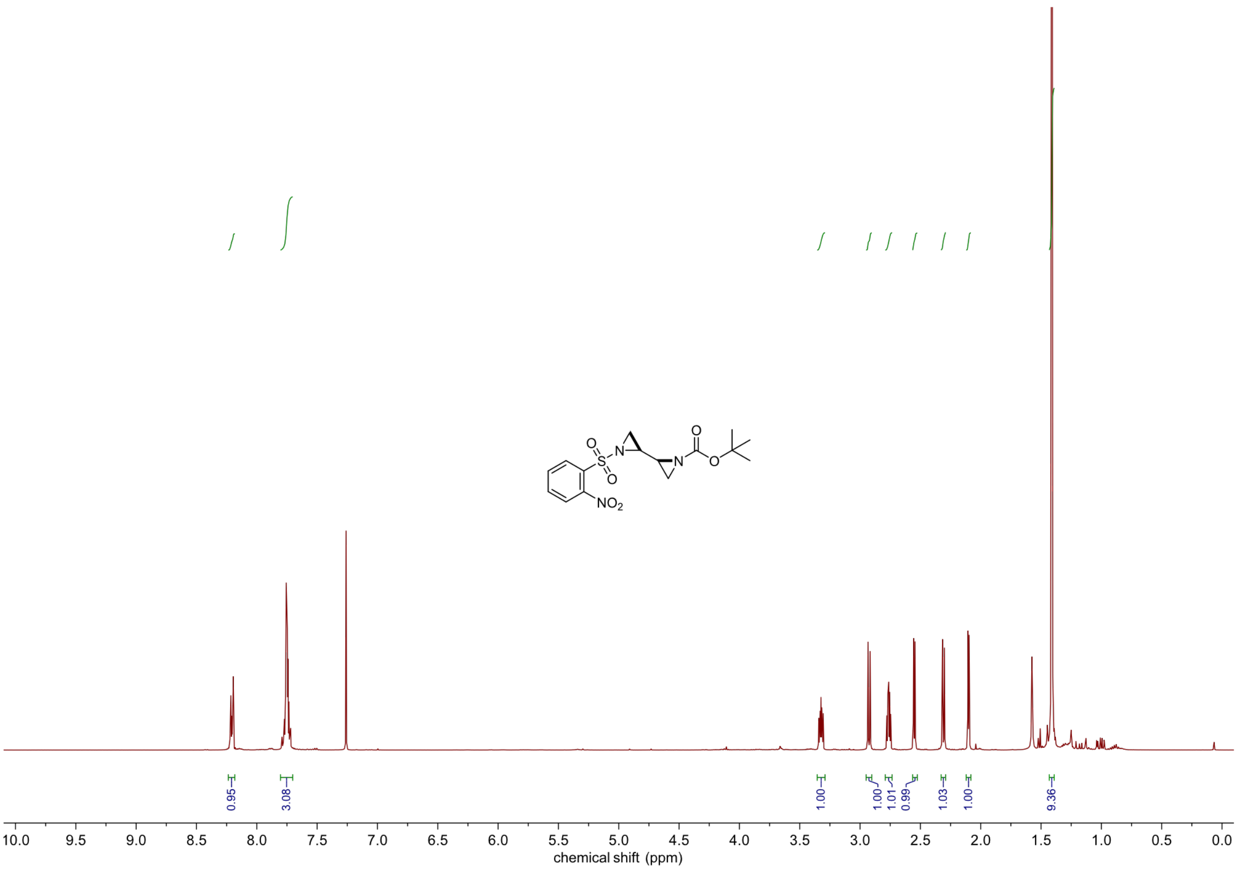

^{1}H NMR spectrum of **S10**.

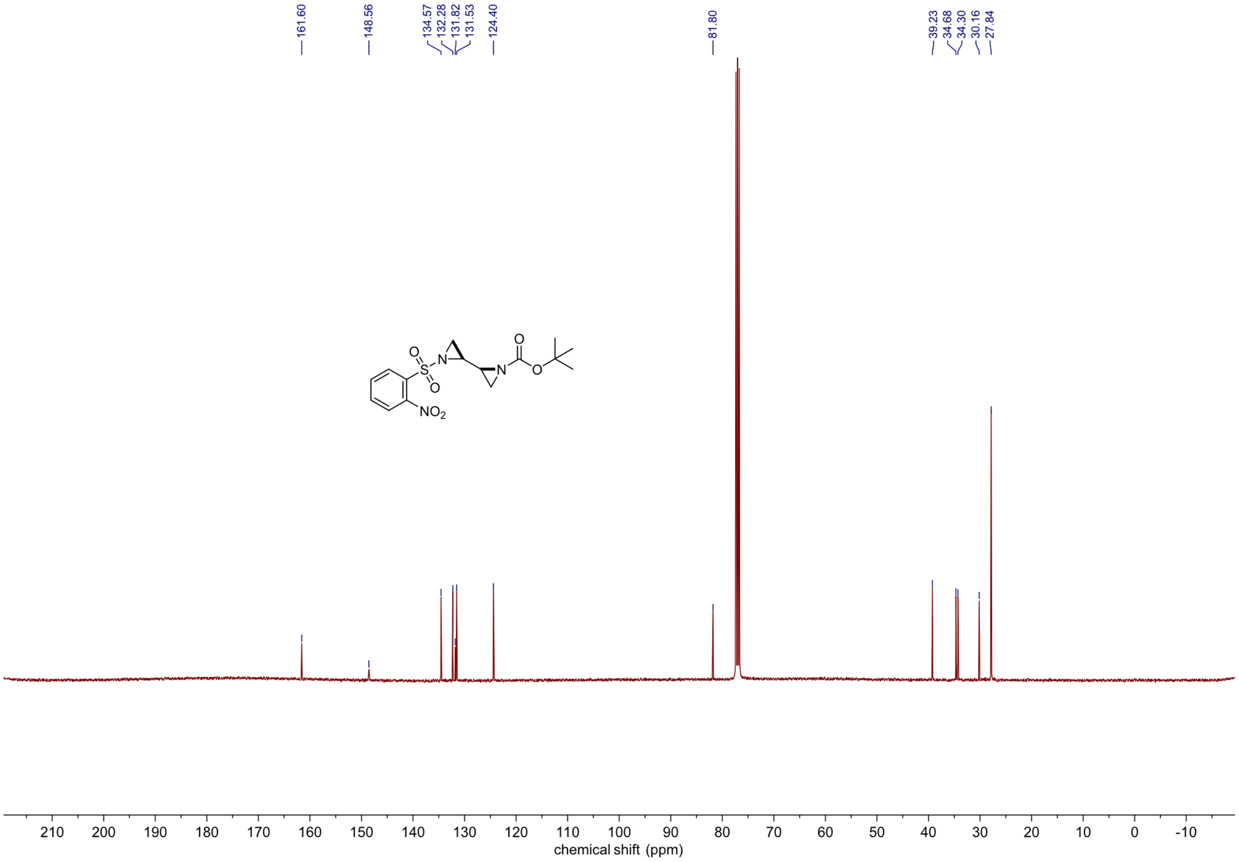

^{13}C NMR spectrum of **S10**.

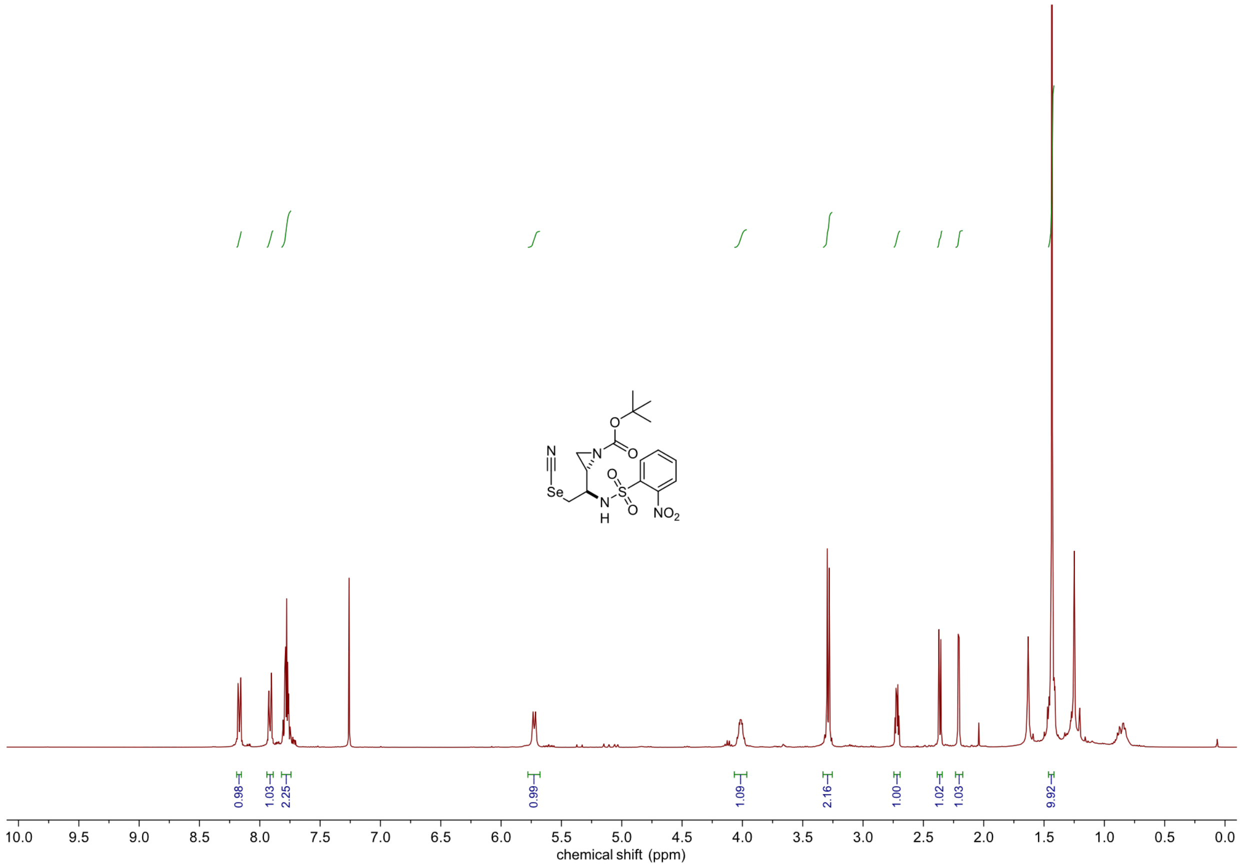

^{1}H NMR spectrum of **S11**.

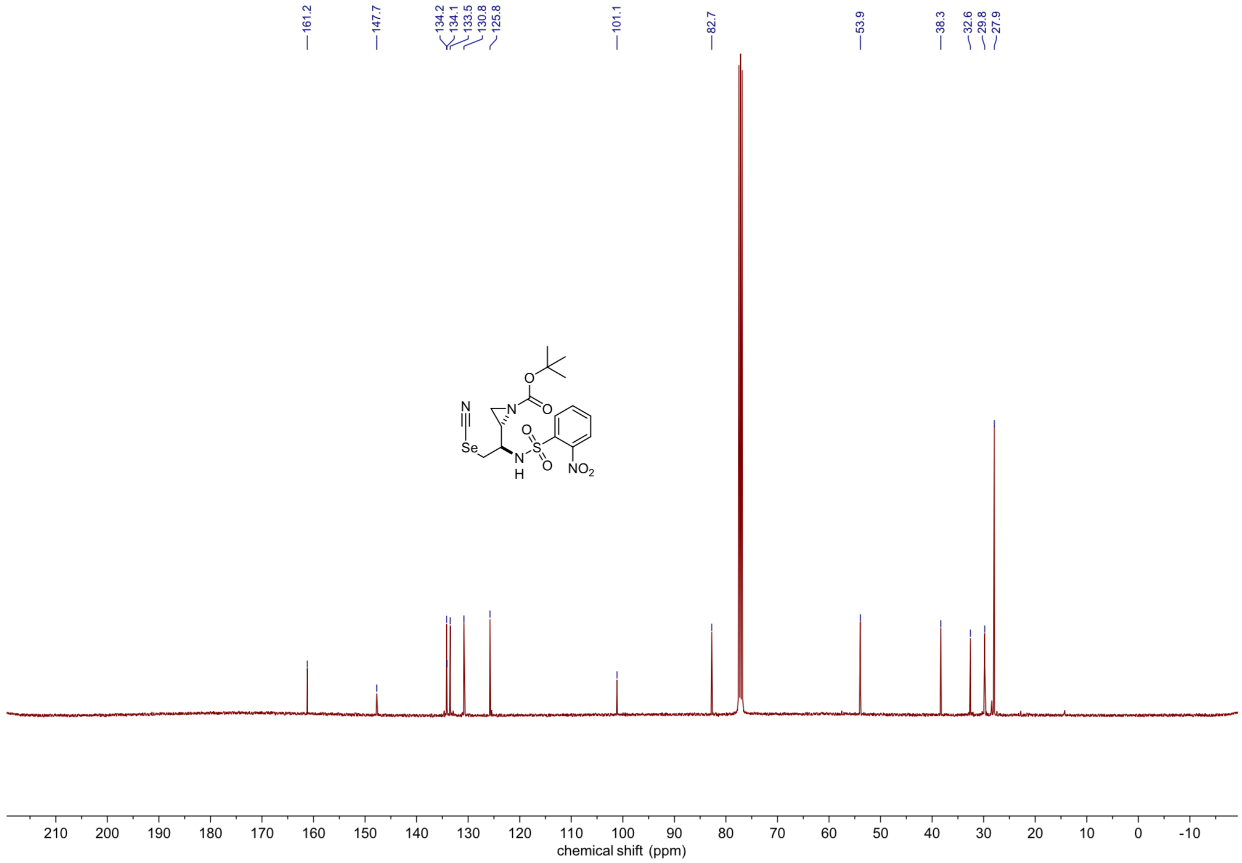

^{13}C NMR spectrum of **S11**.

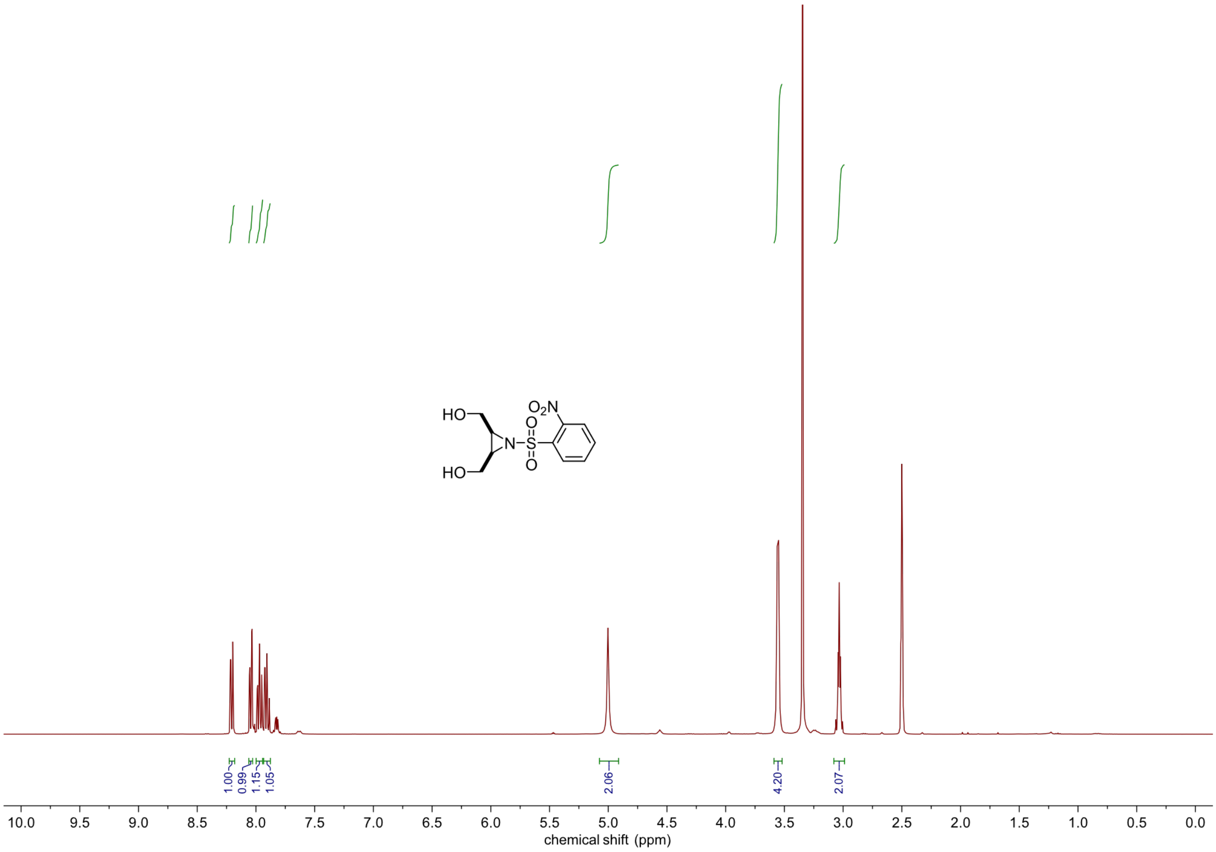

^{1}H NMR spectrum of **S4**.

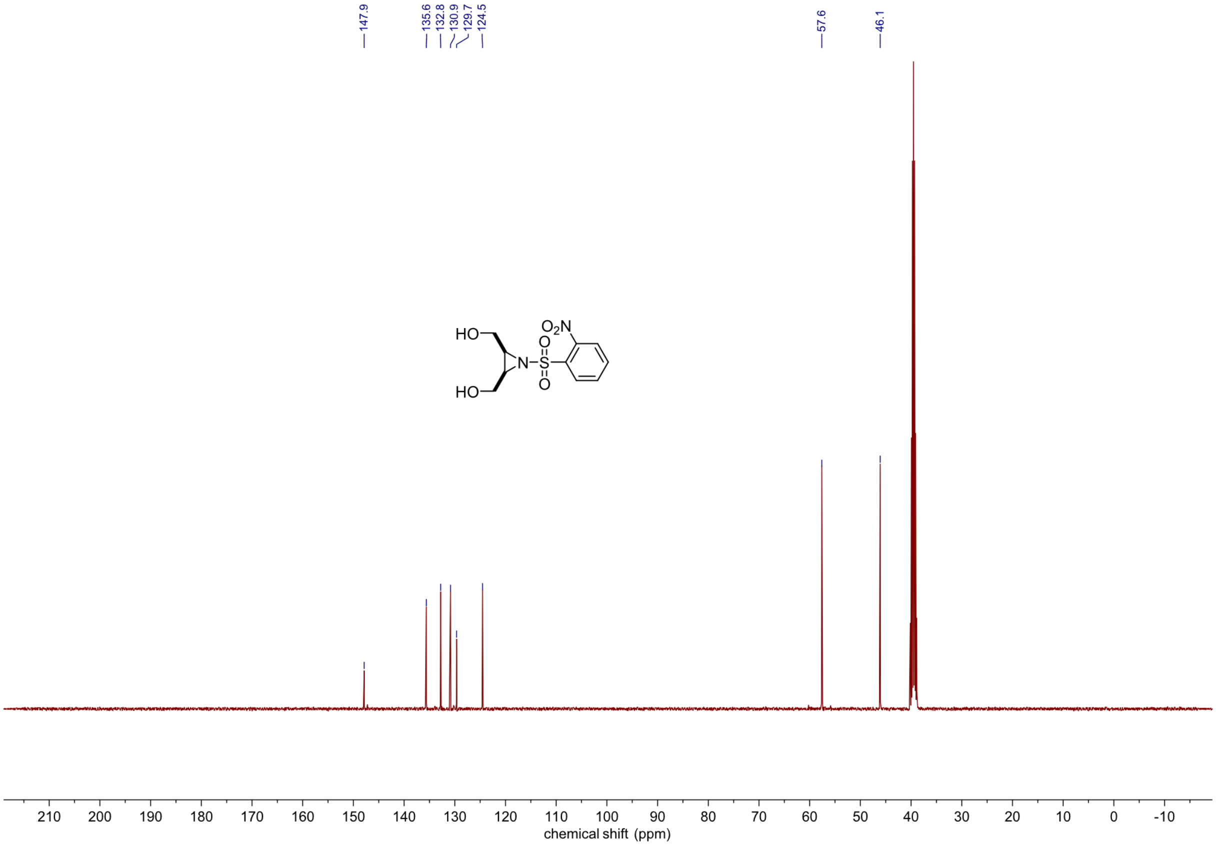

^{13}C NMR spectrum of **S4**.

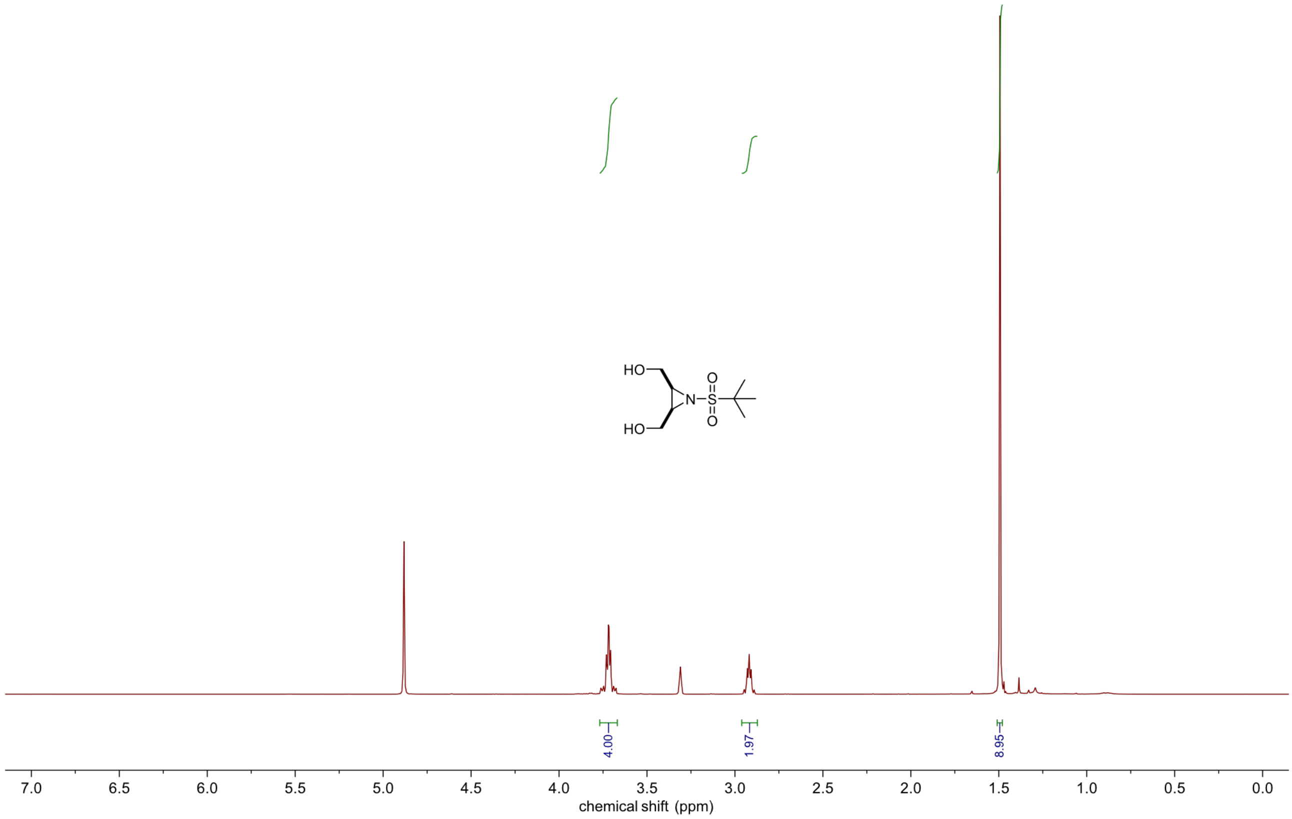

^{1}H NMR spectrum of **S12**.

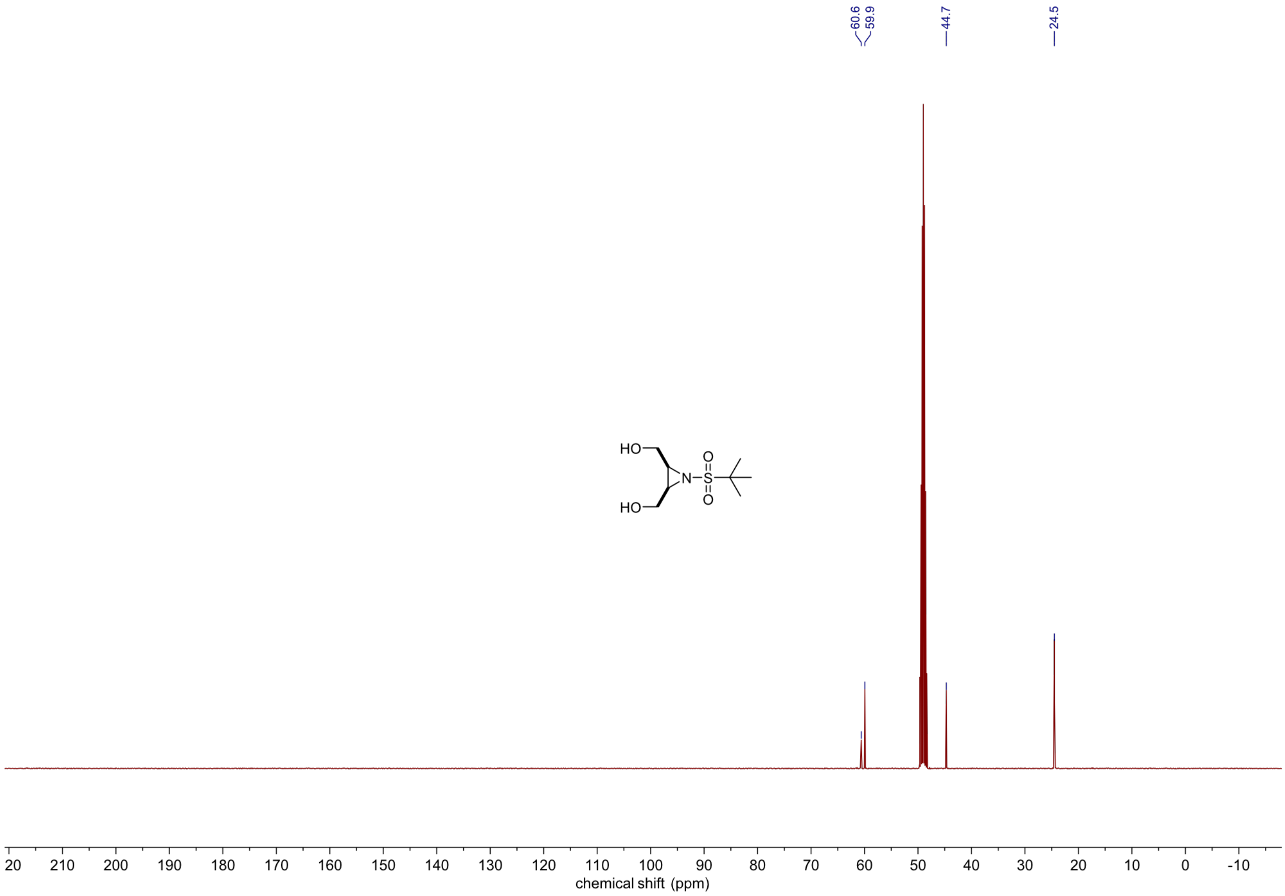

^{13}C NMR spectrum of **S12**.

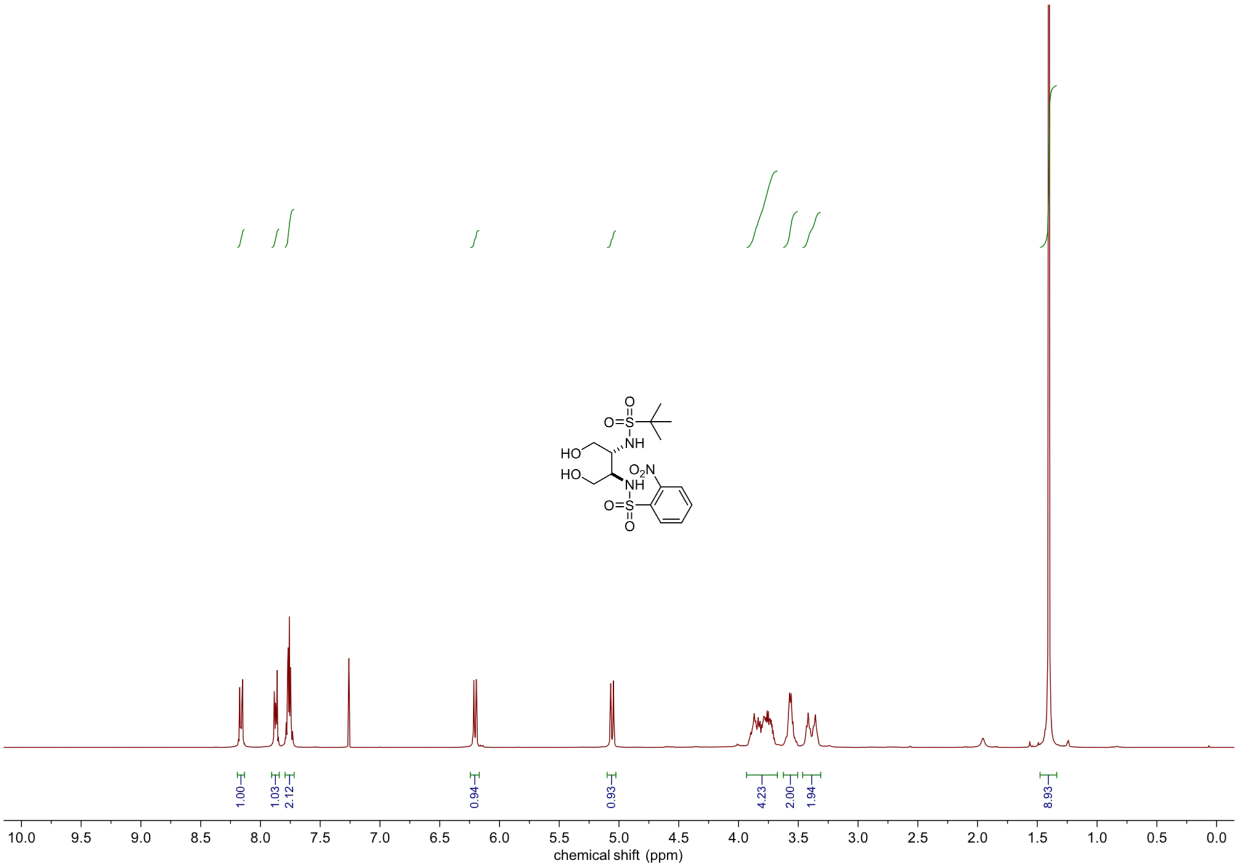

^{1}H NMR spectrum of **S5**.

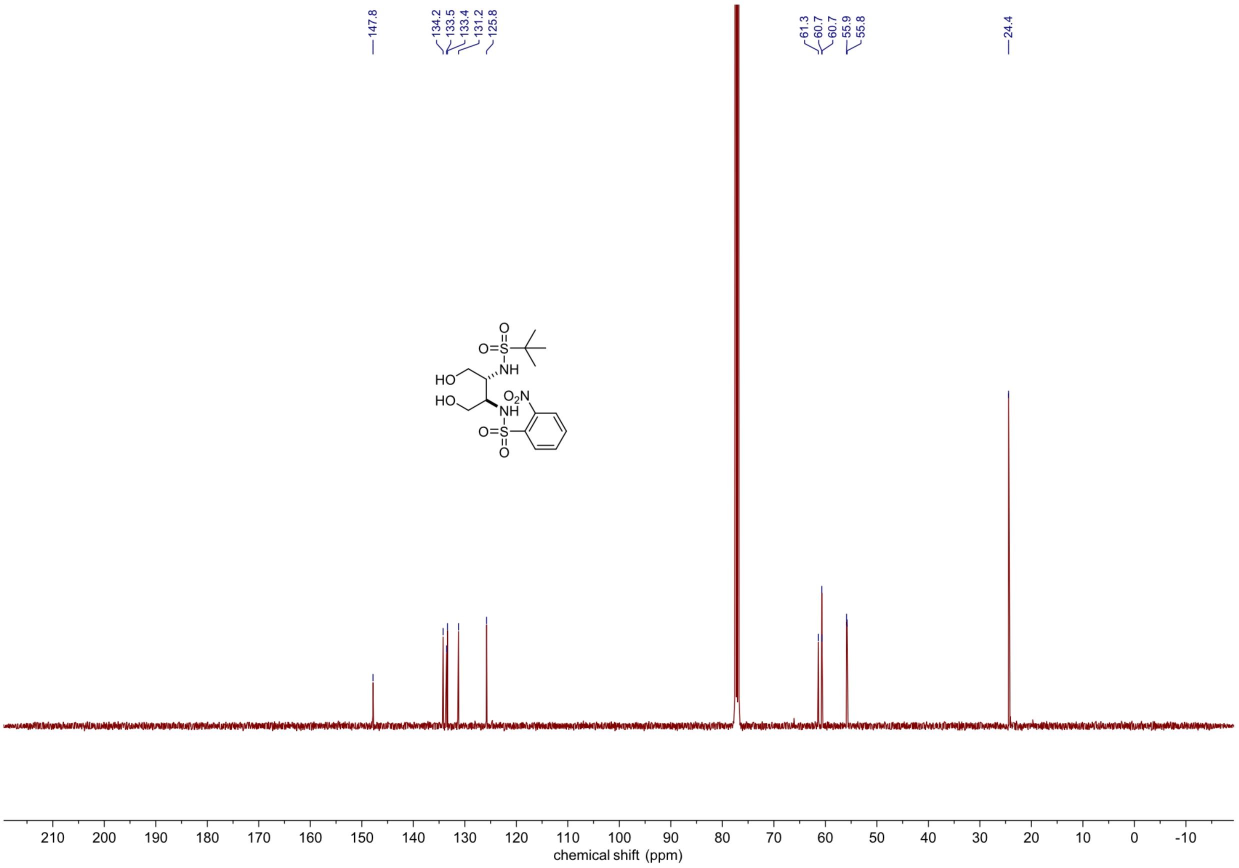

^{13}C NMR spectrum of **S5**.

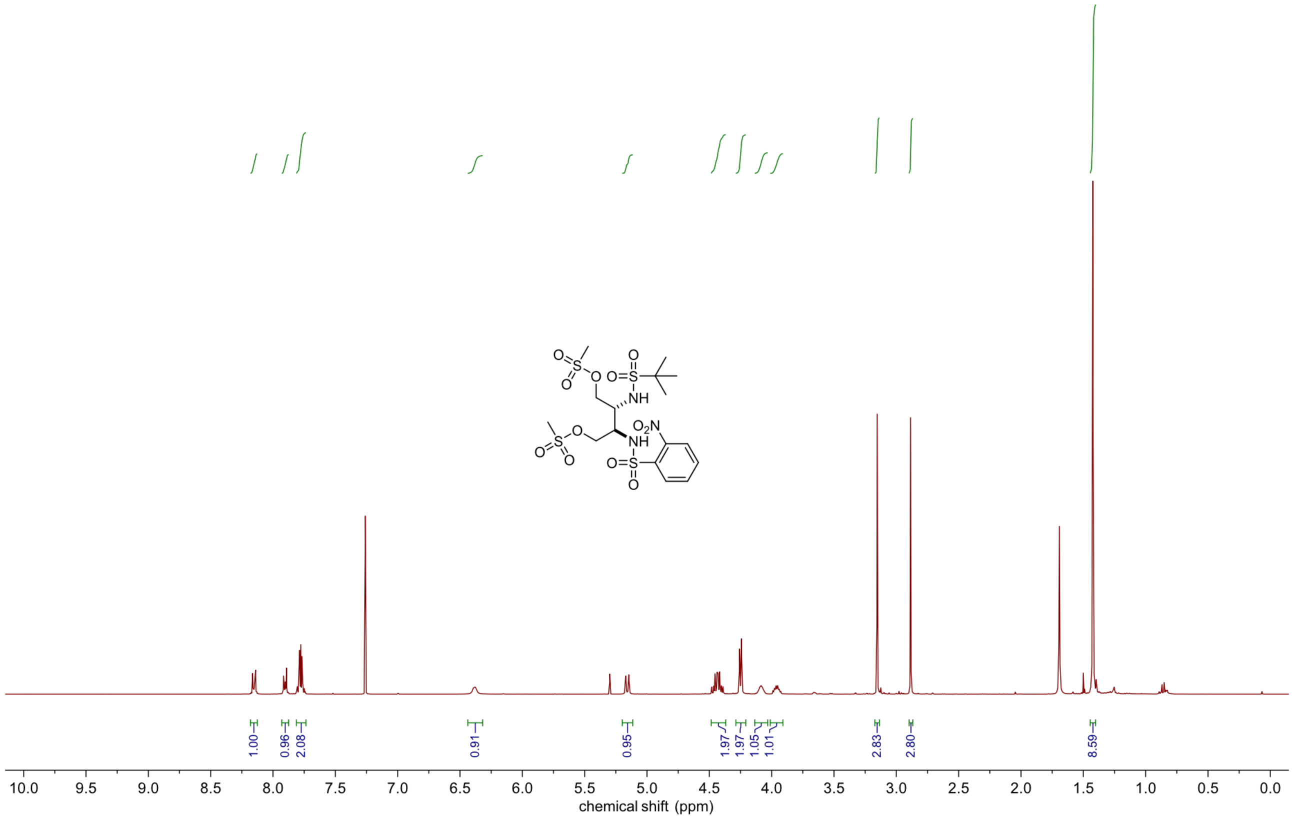

^{1}H NMR spectrum of **S6**.

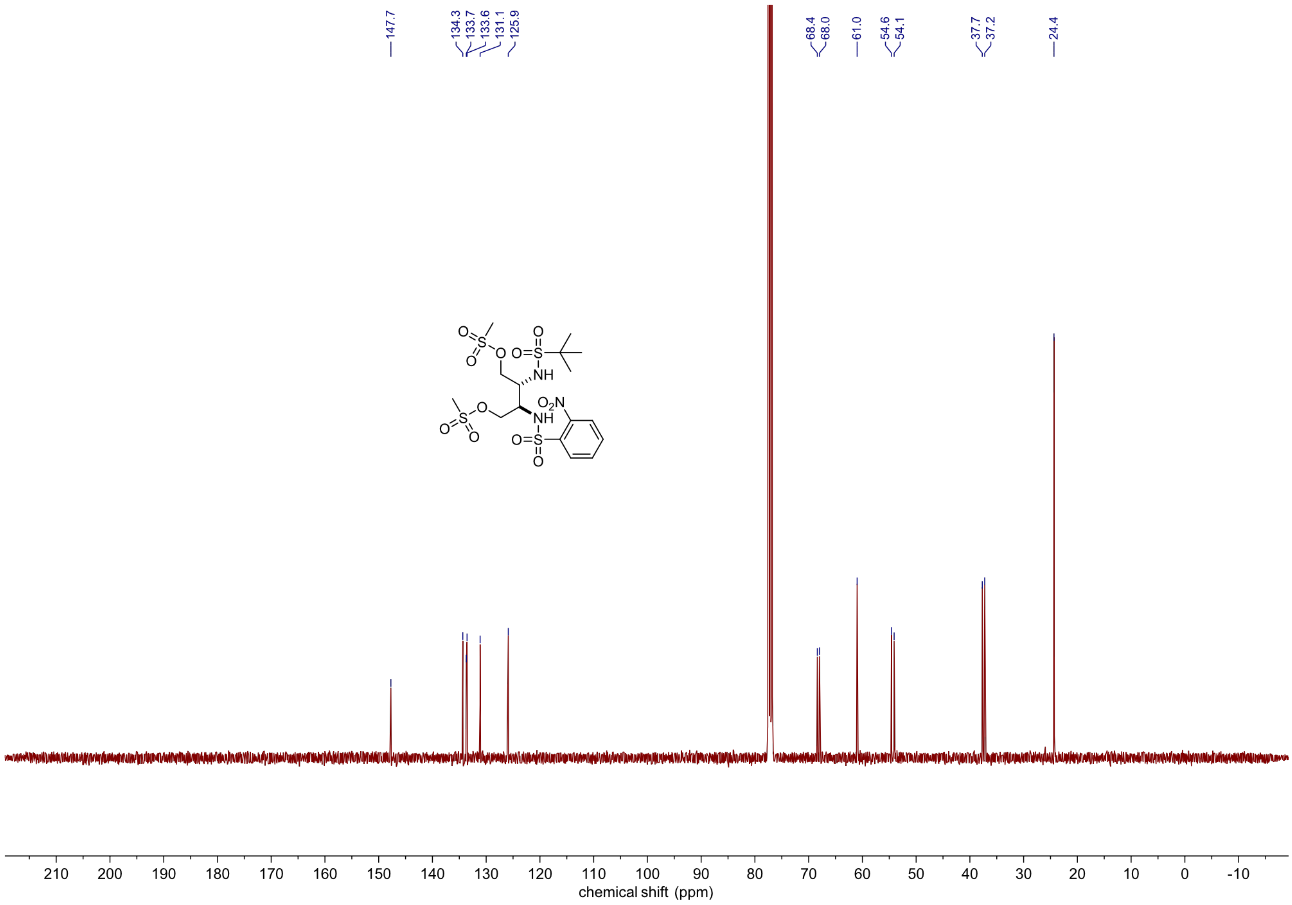

^{13}C NMR spectrum of **S6**.

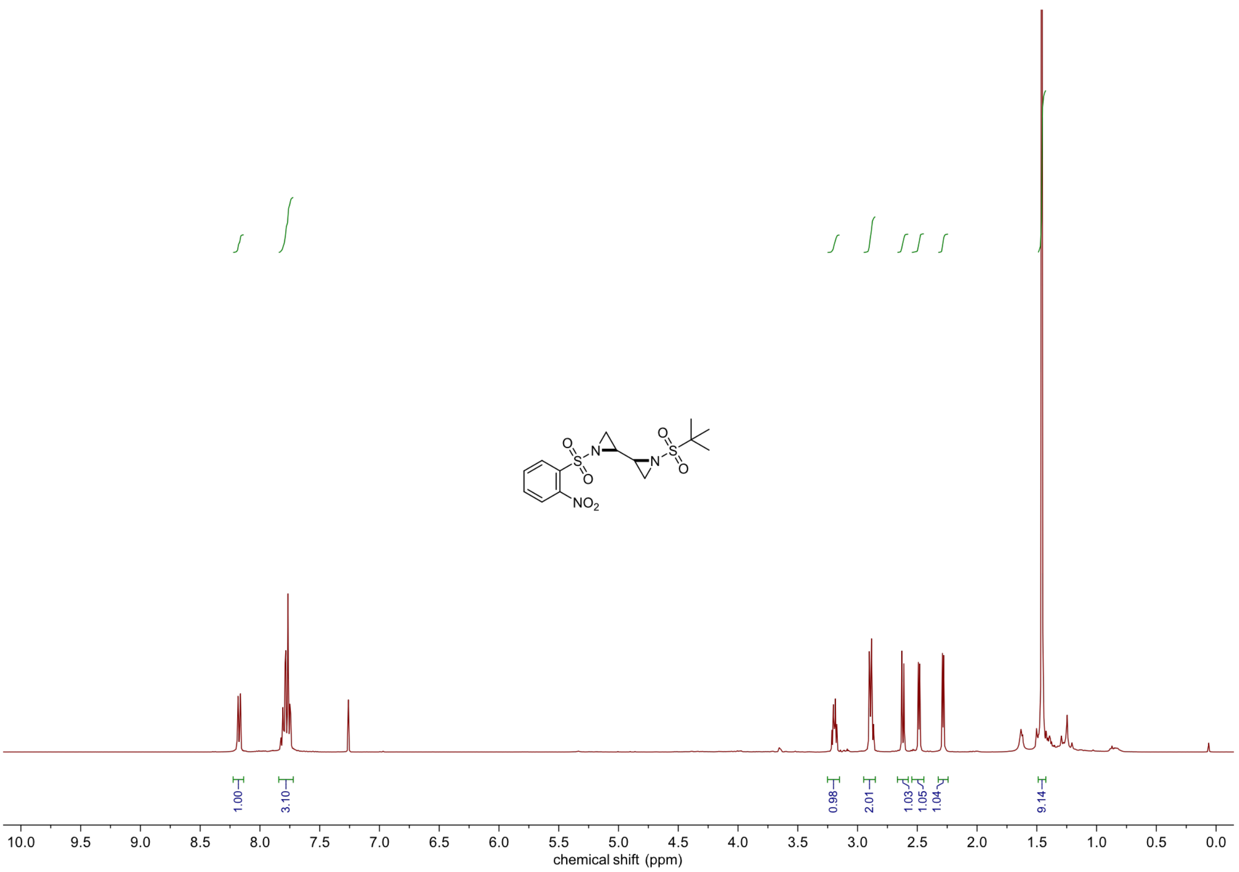

^{1}H NMR spectrum of **S7**.

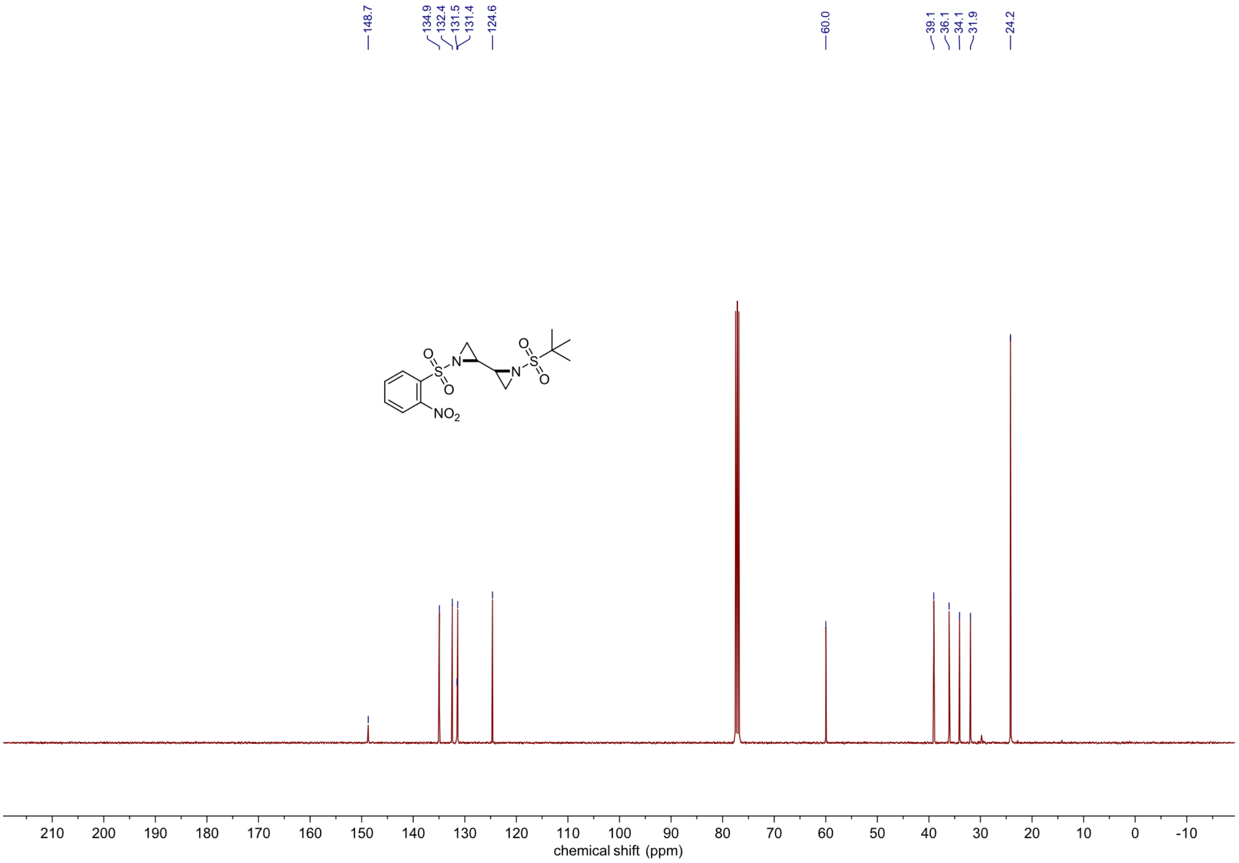

^{13}C NMR spectrum of **S7**.

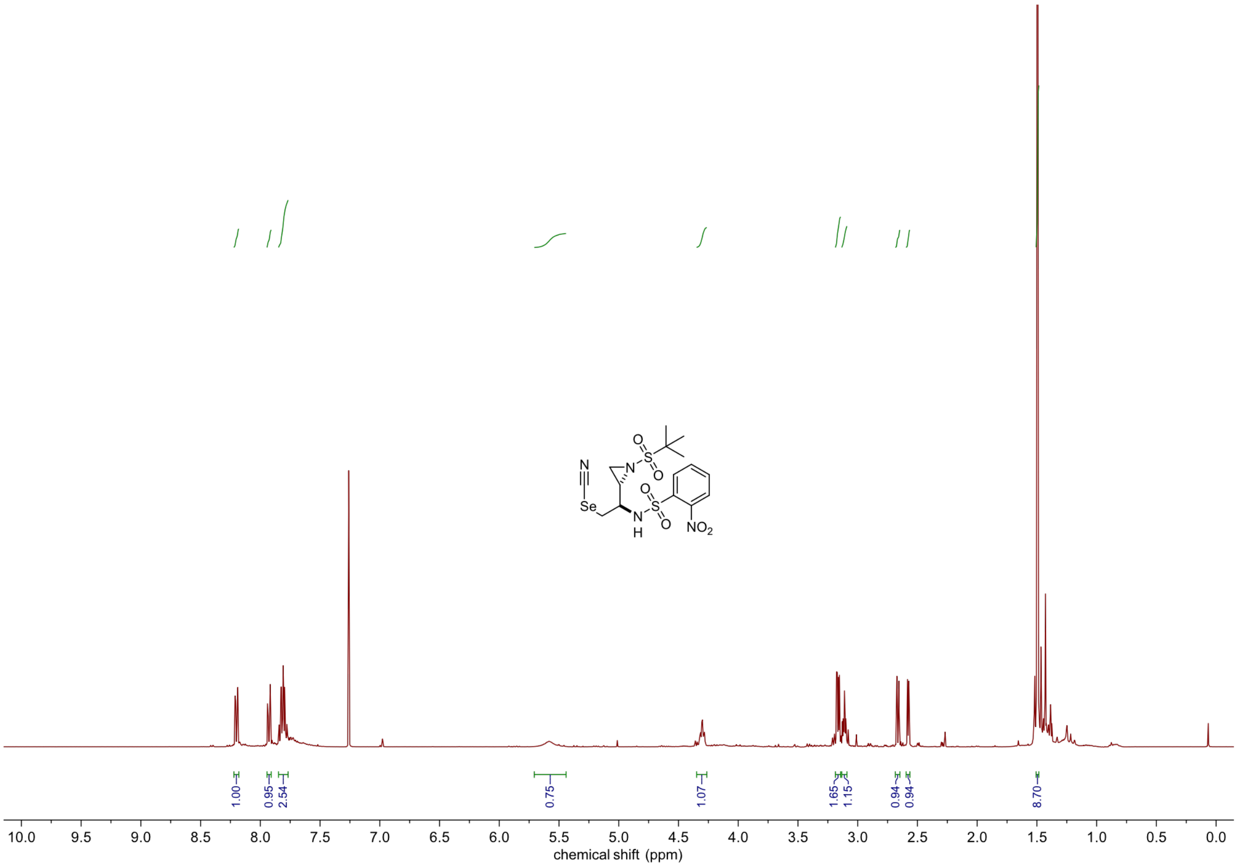

^{1}H NMR spectrum of **S8**.

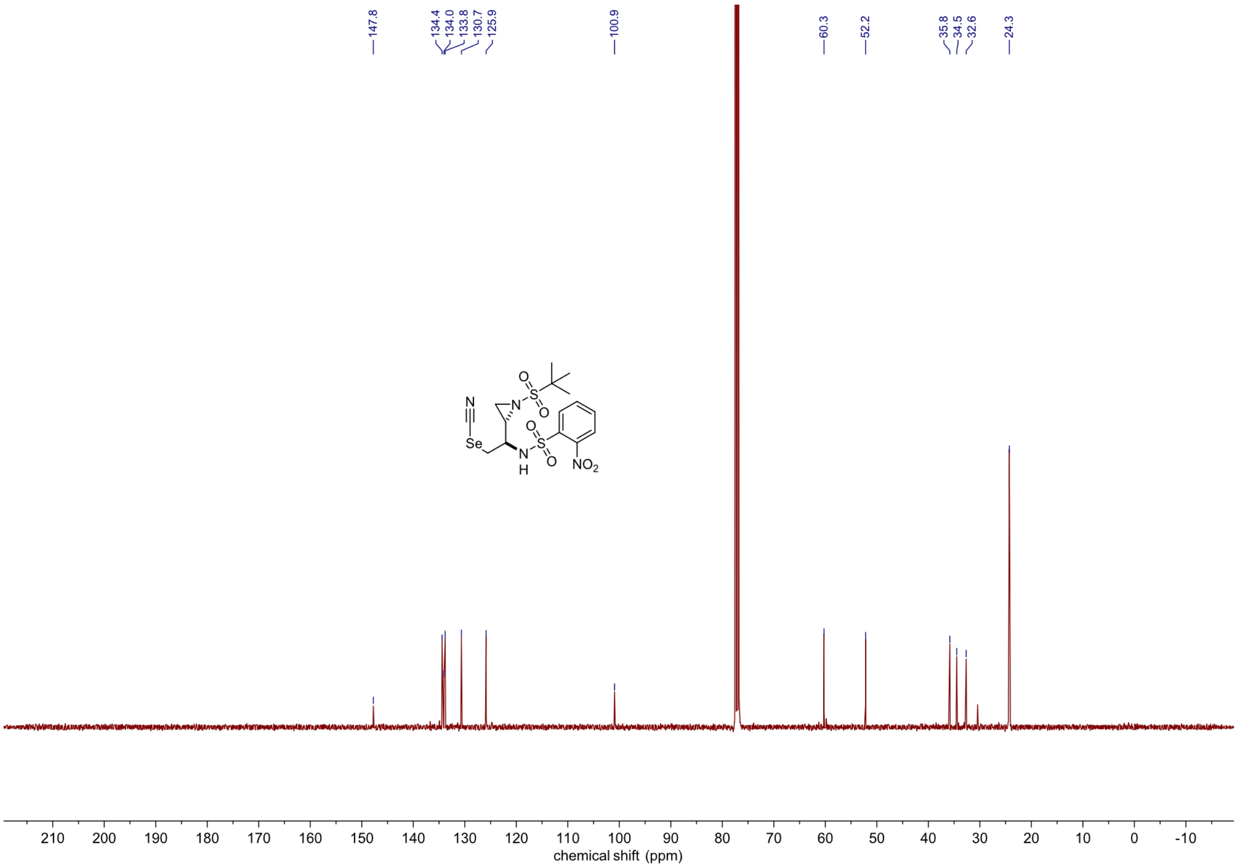

^{13}C NMR spectrum of **S8**.

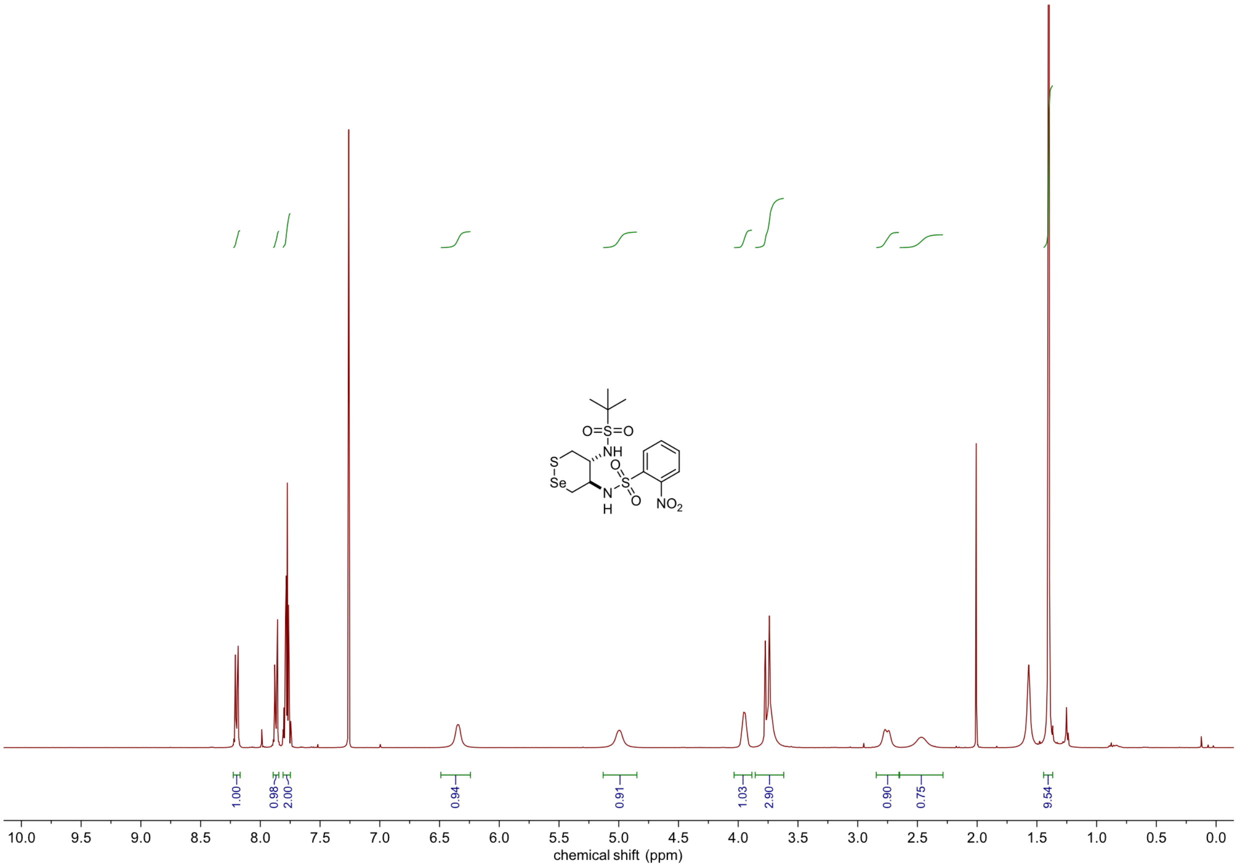

^{1}H NMR spectrum of **S9**.

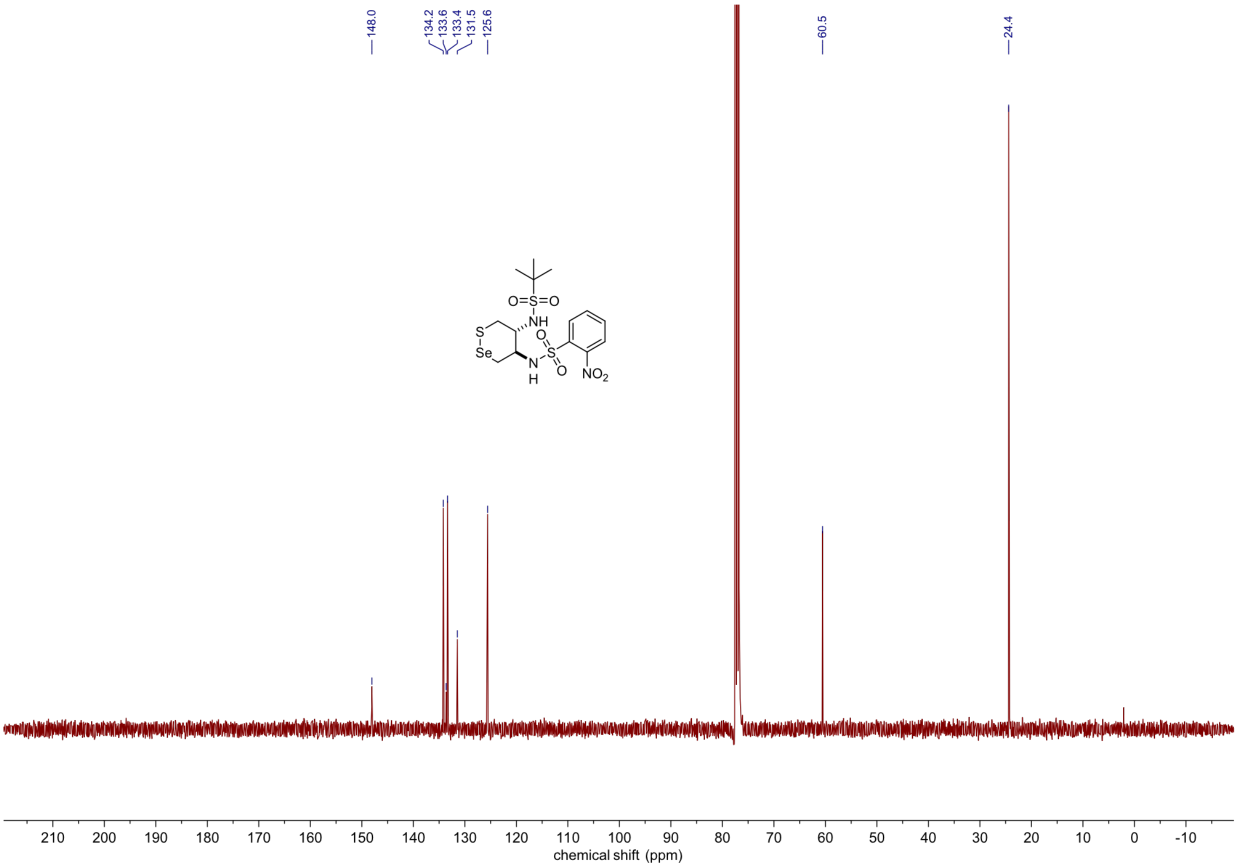

^{13}C NMR spectrum of **S9**.

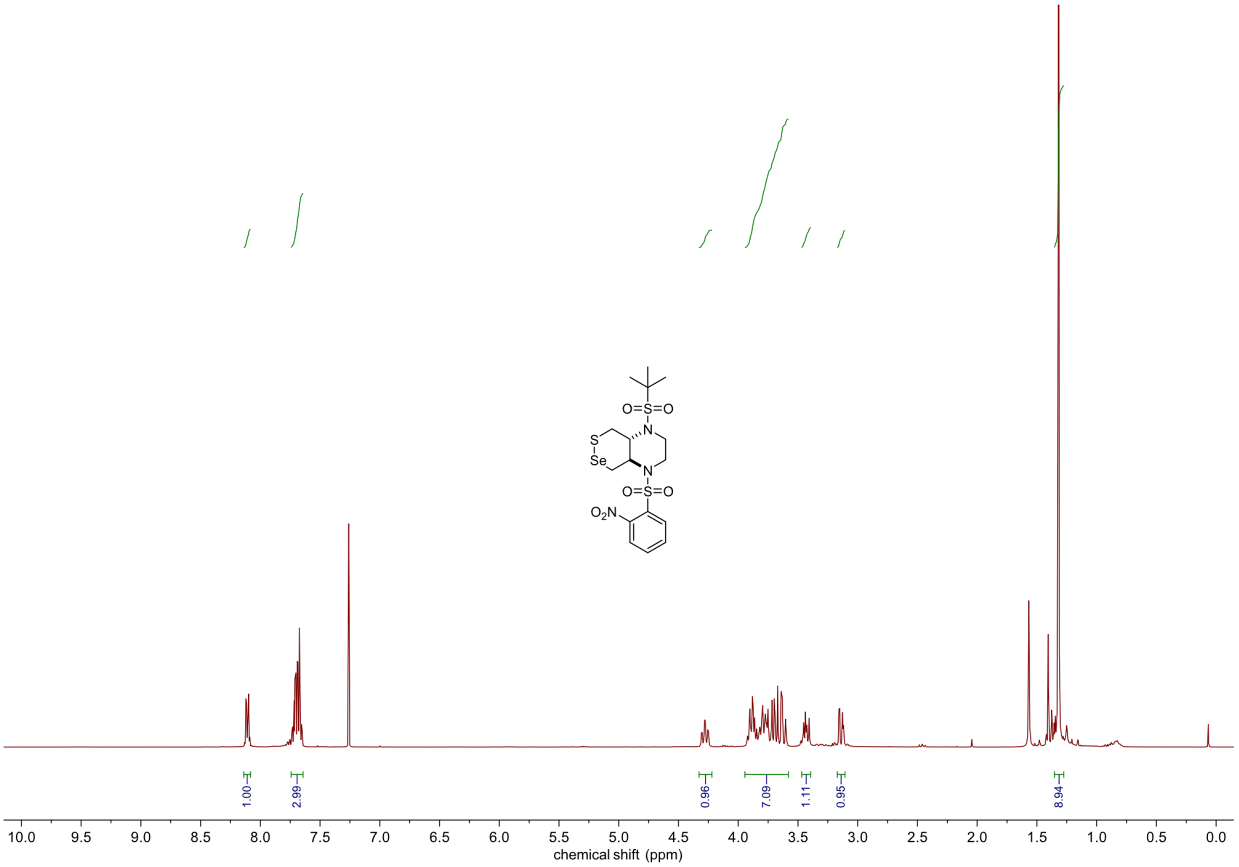

^1H NMR spectrum of **7T**.

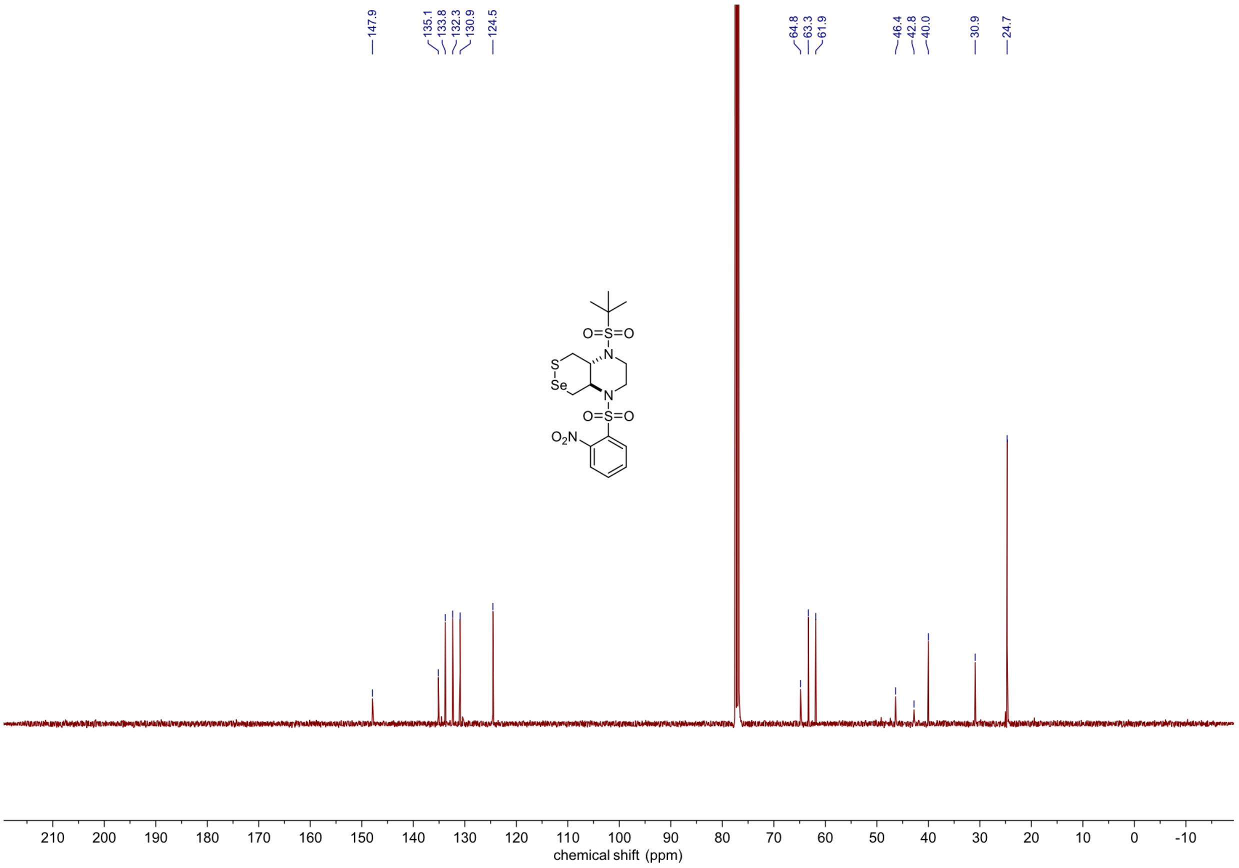

^{13}C NMR spectrum of **7T**.

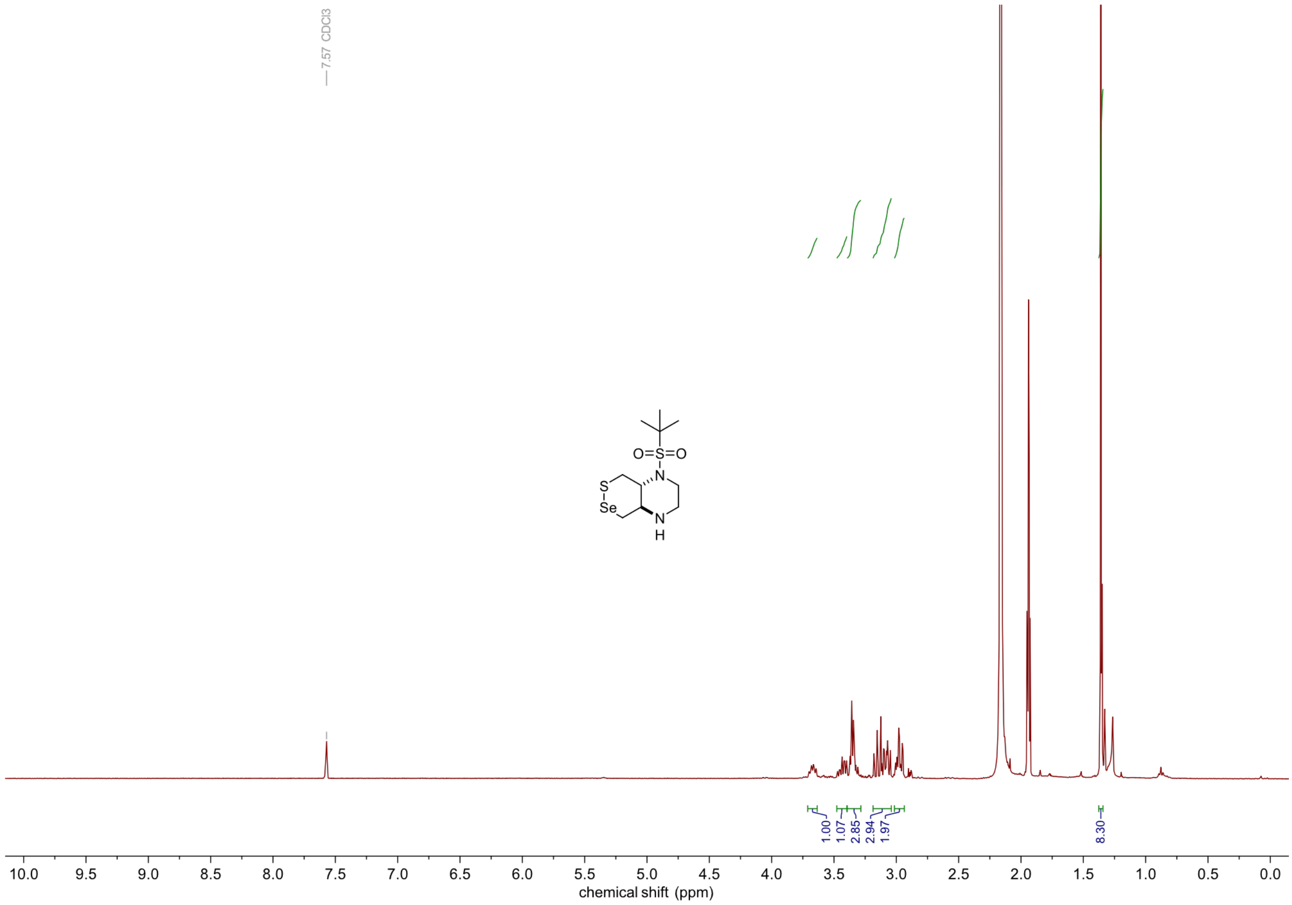

^{1}H NMR spectrum of **S13**.

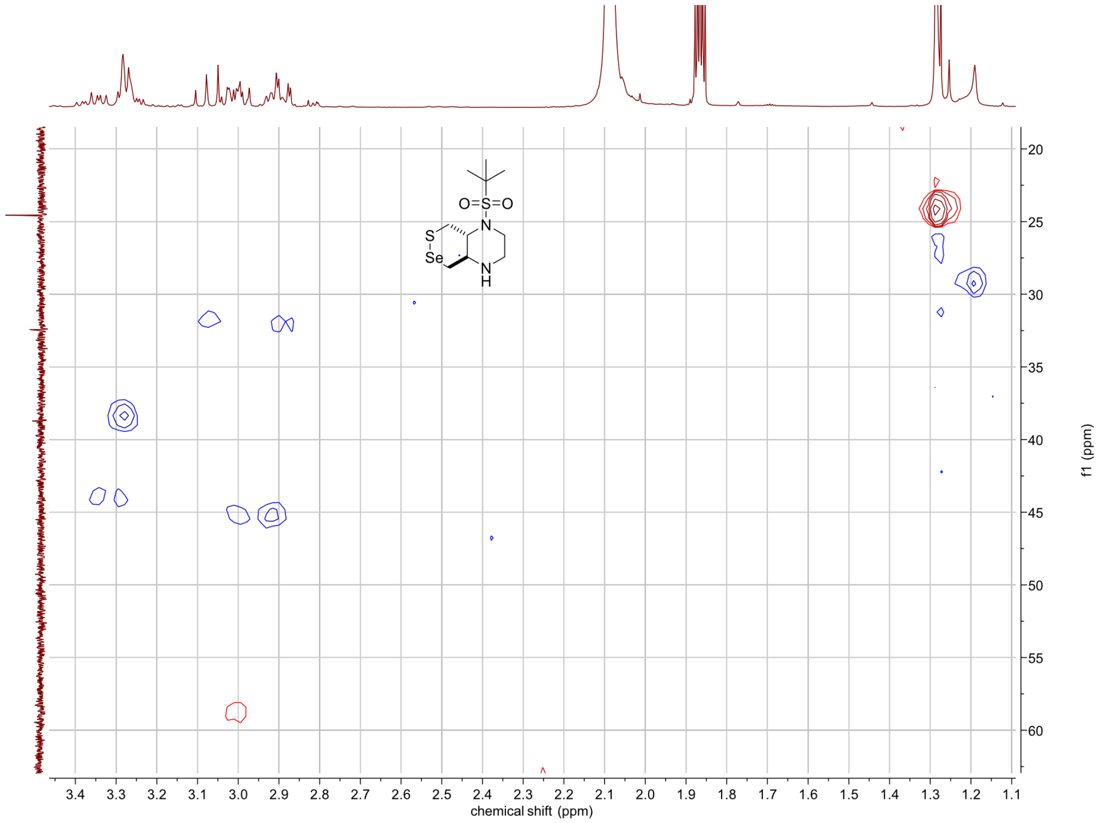

HSQC spectrum of **S13**.

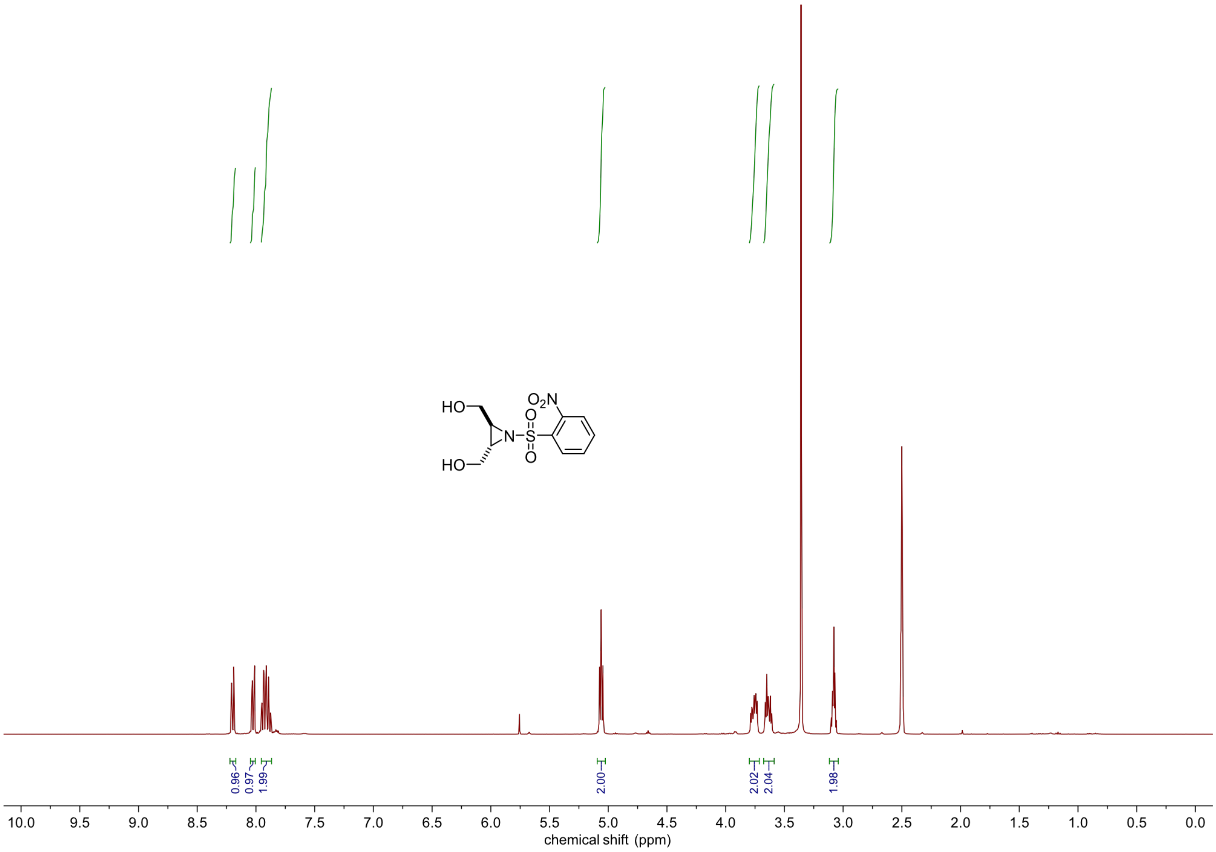

^{1}H NMR spectrum of **2a**.

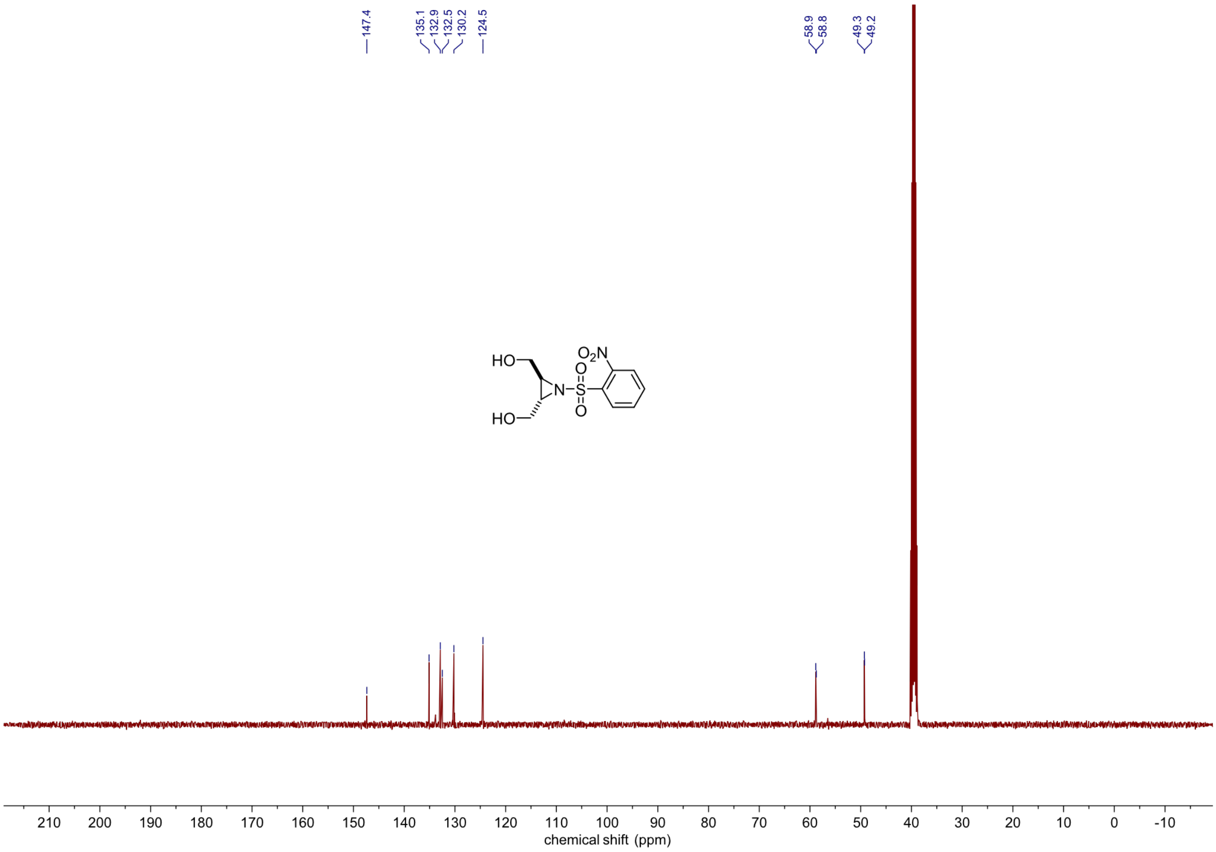

^{13}C NMR spectrum of **2a**.

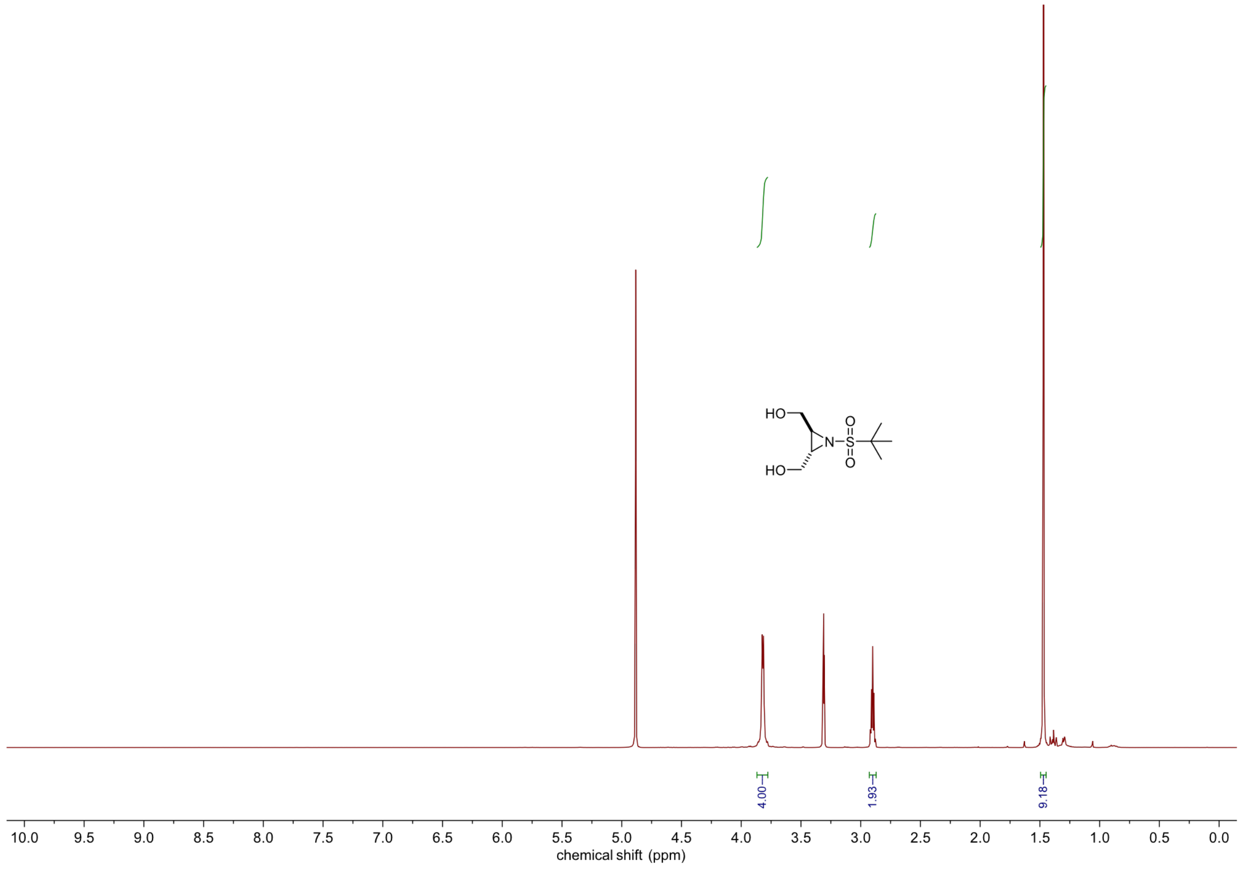

^{1}H NMR spectrum of **2b**.

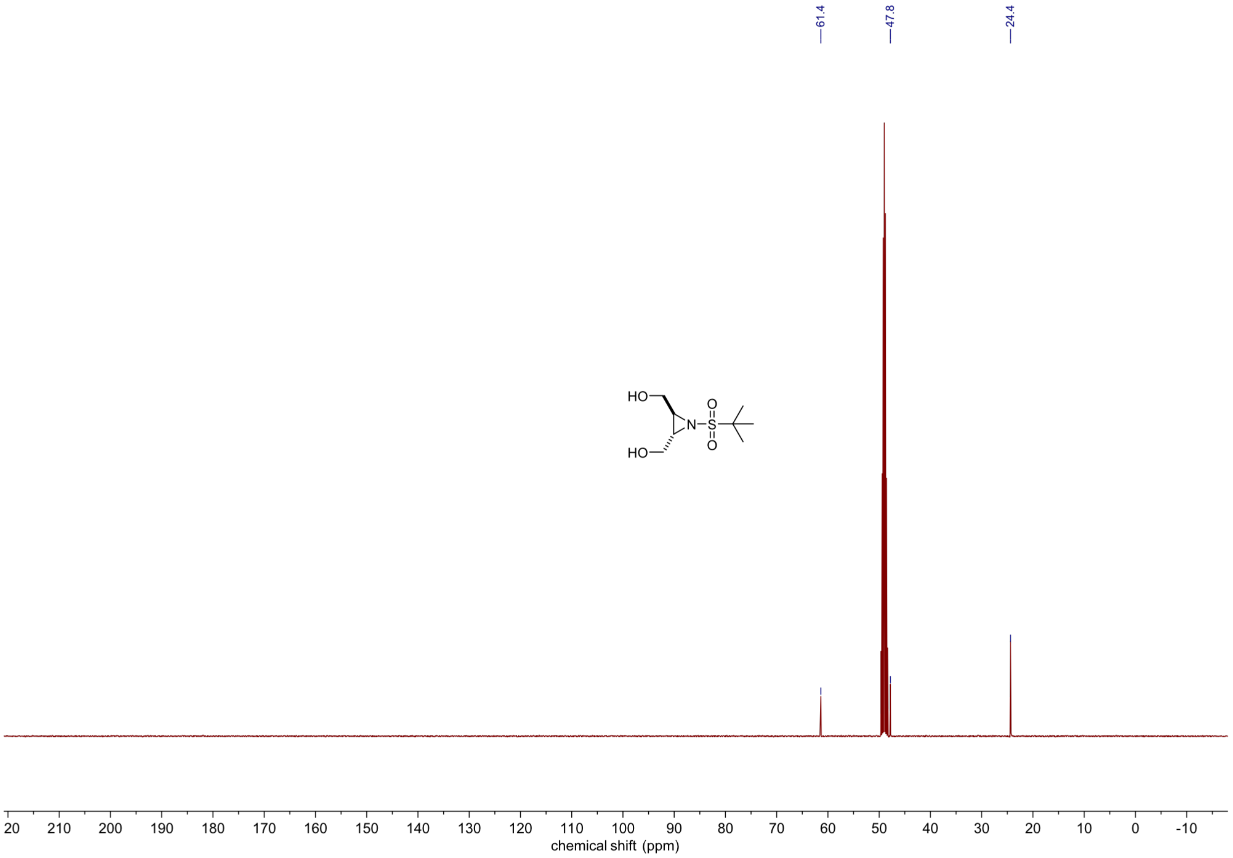

^{13}C NMR spectrum of **2b**.

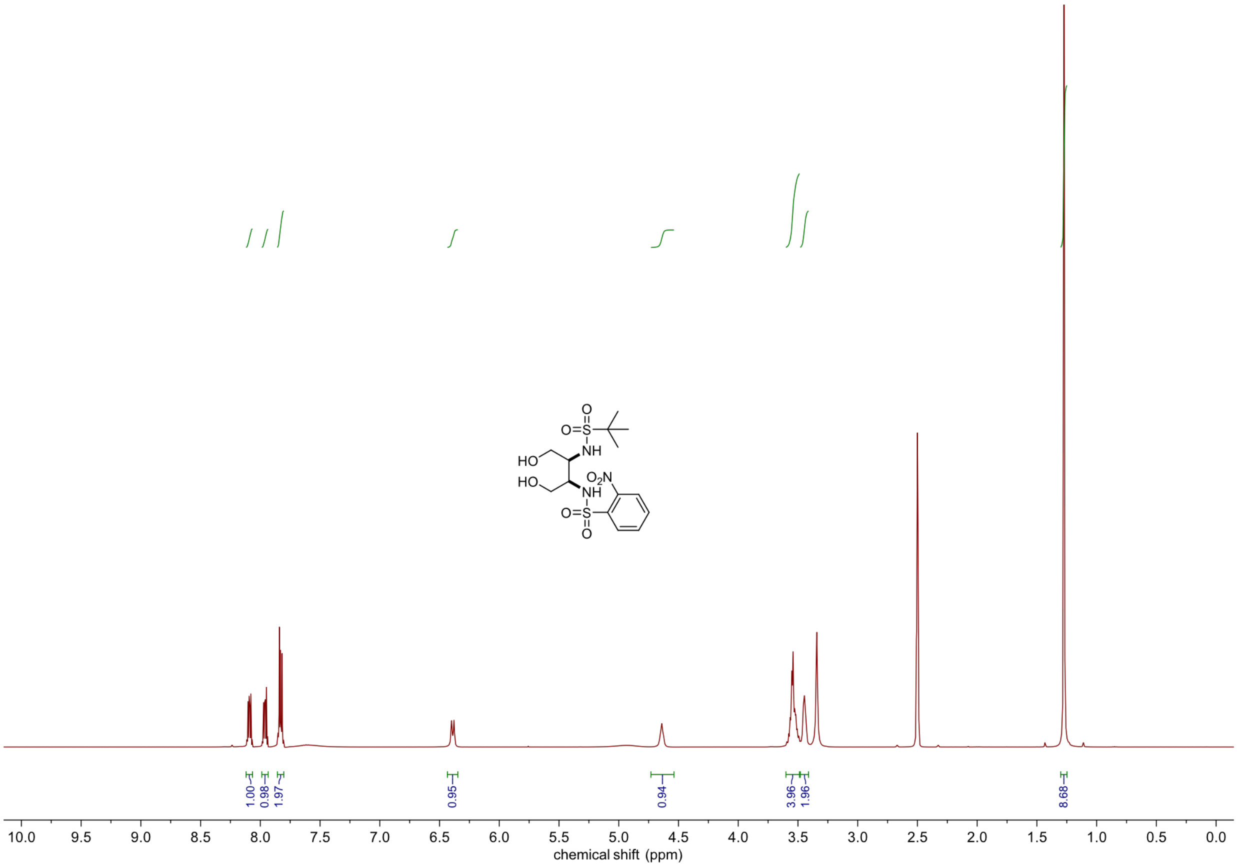

^{1}H NMR spectrum of **3**.

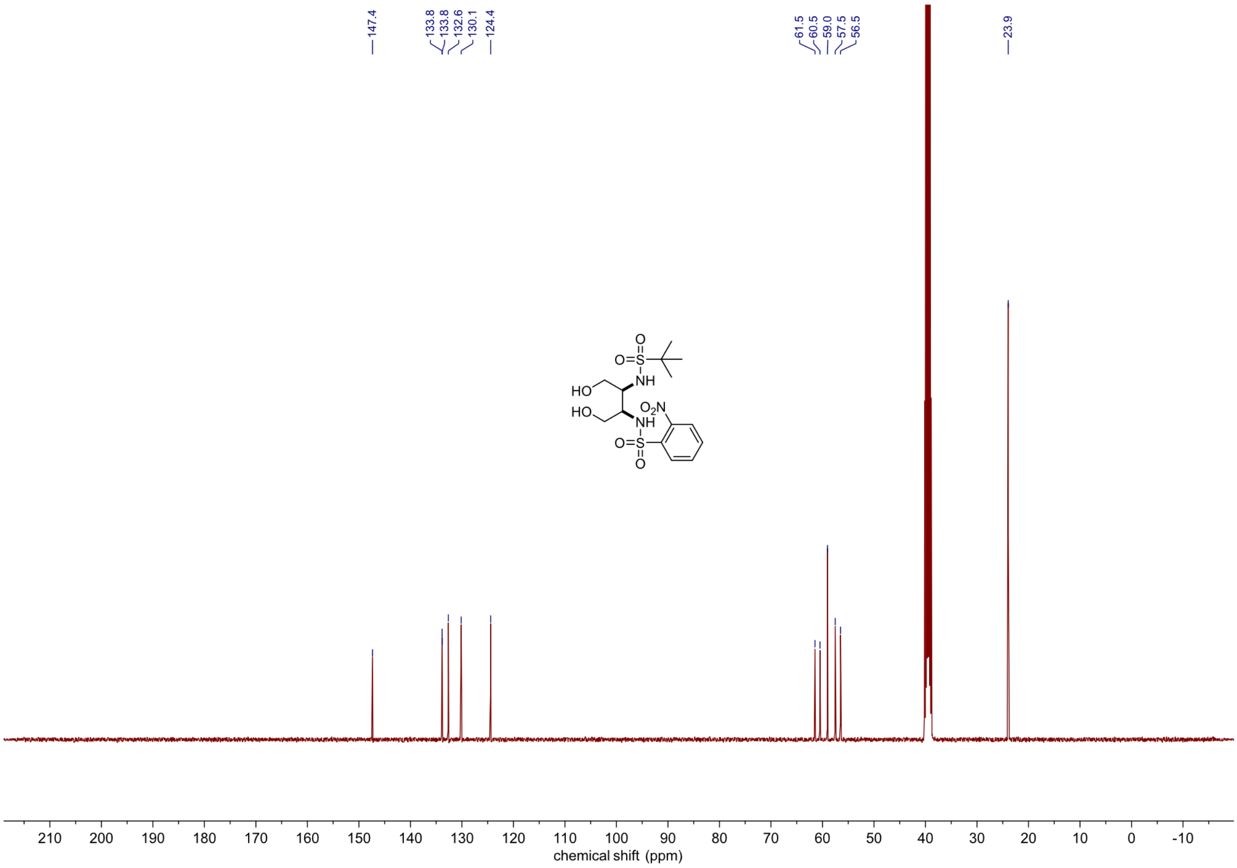

^{13}C NMR spectrum of **3**.

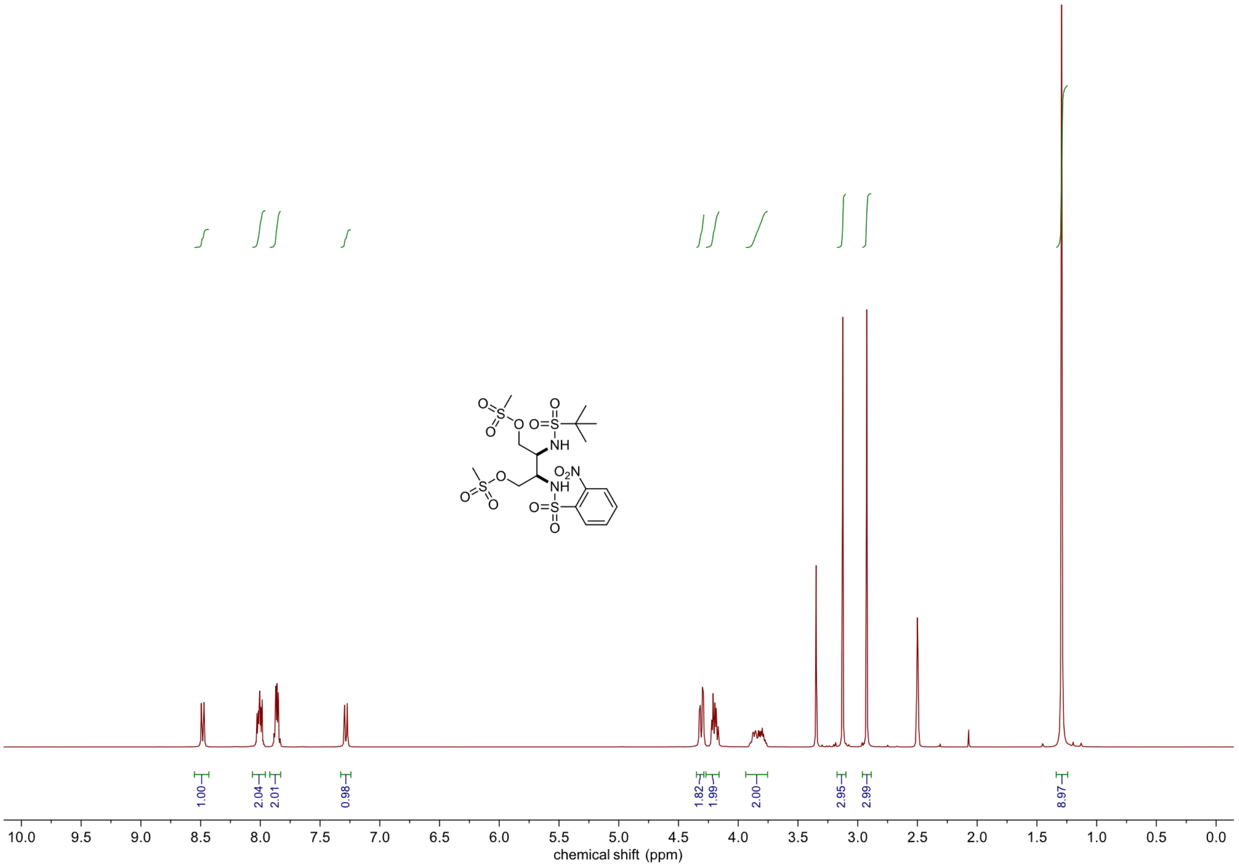

^{1}H NMR spectrum of **4**.

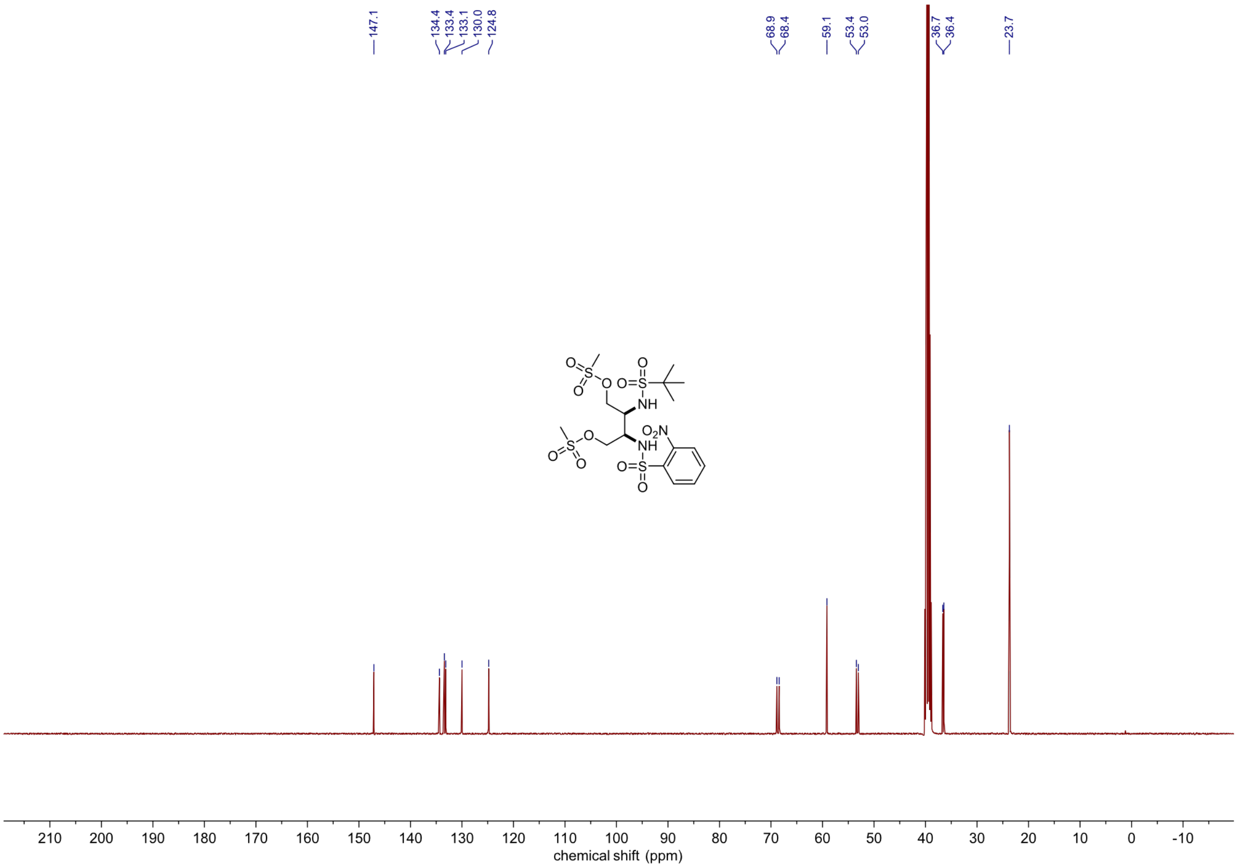

^{13}C NMR spectrum of **4**.

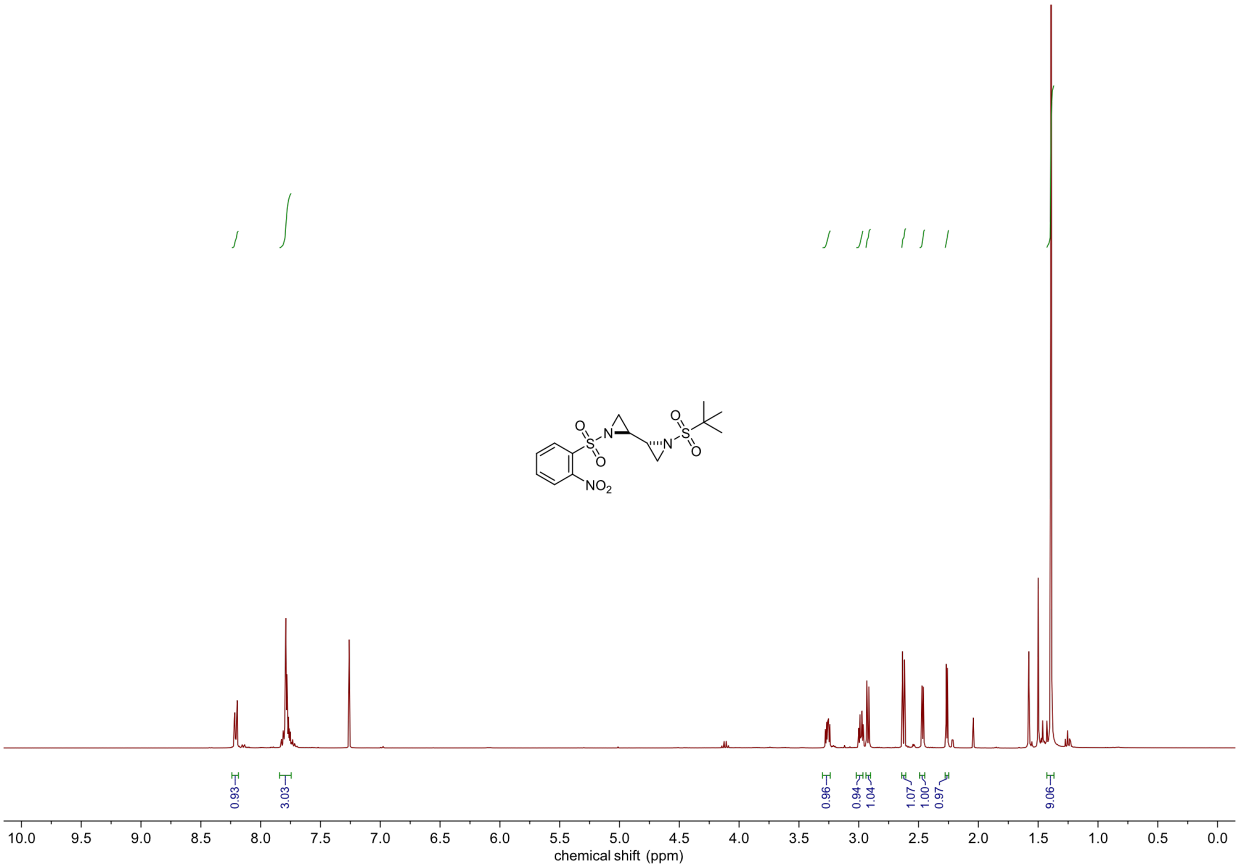

^{13}C NMR spectrum of **5**.

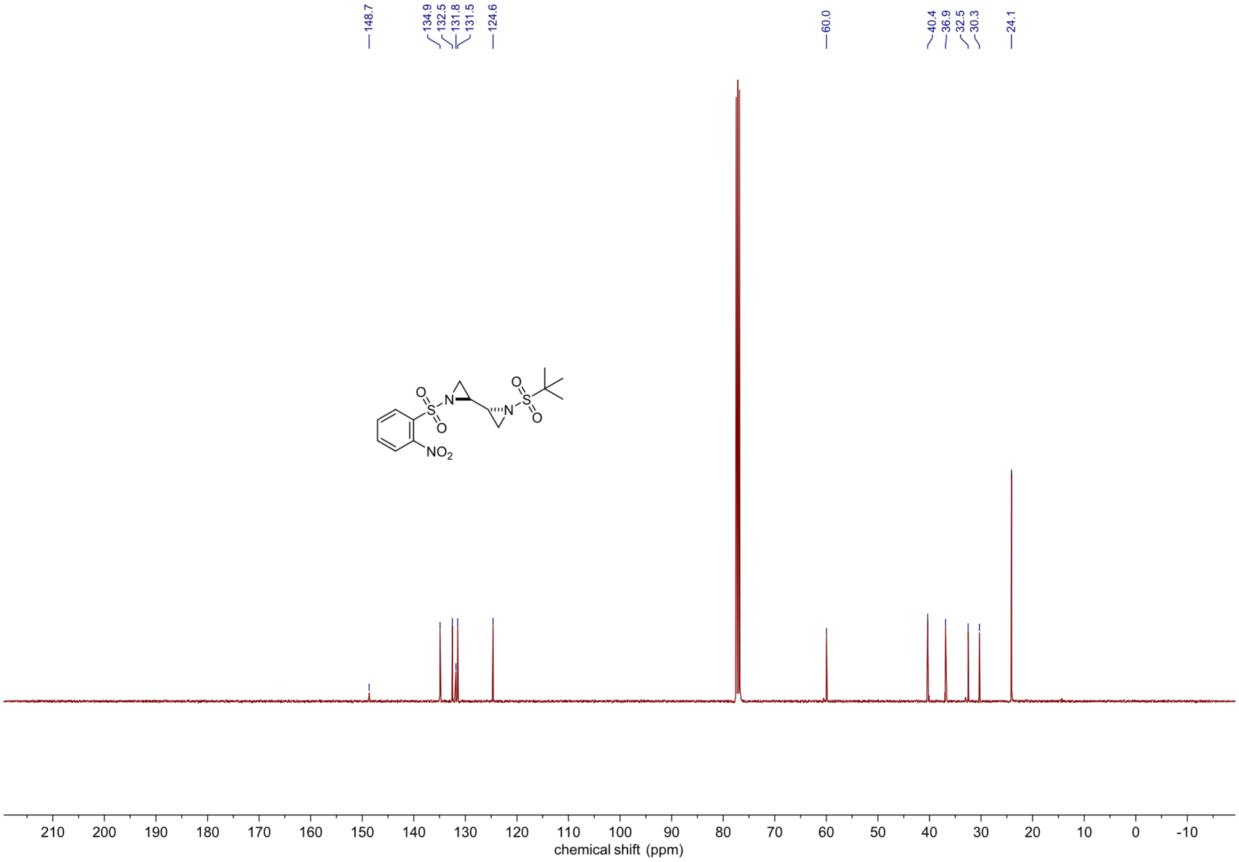

^{13}C NMR spectrum of **5**.

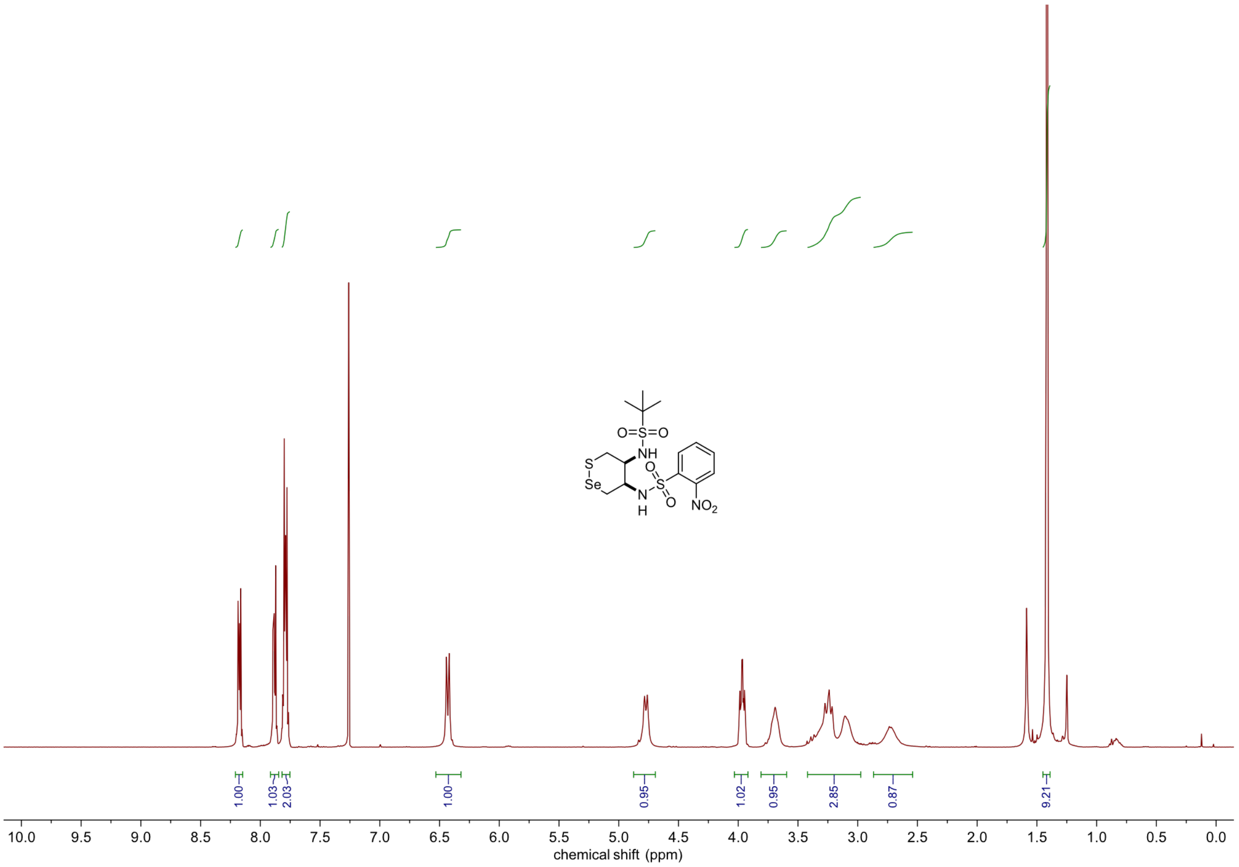

^{1}H NMR spectrum of **6**.

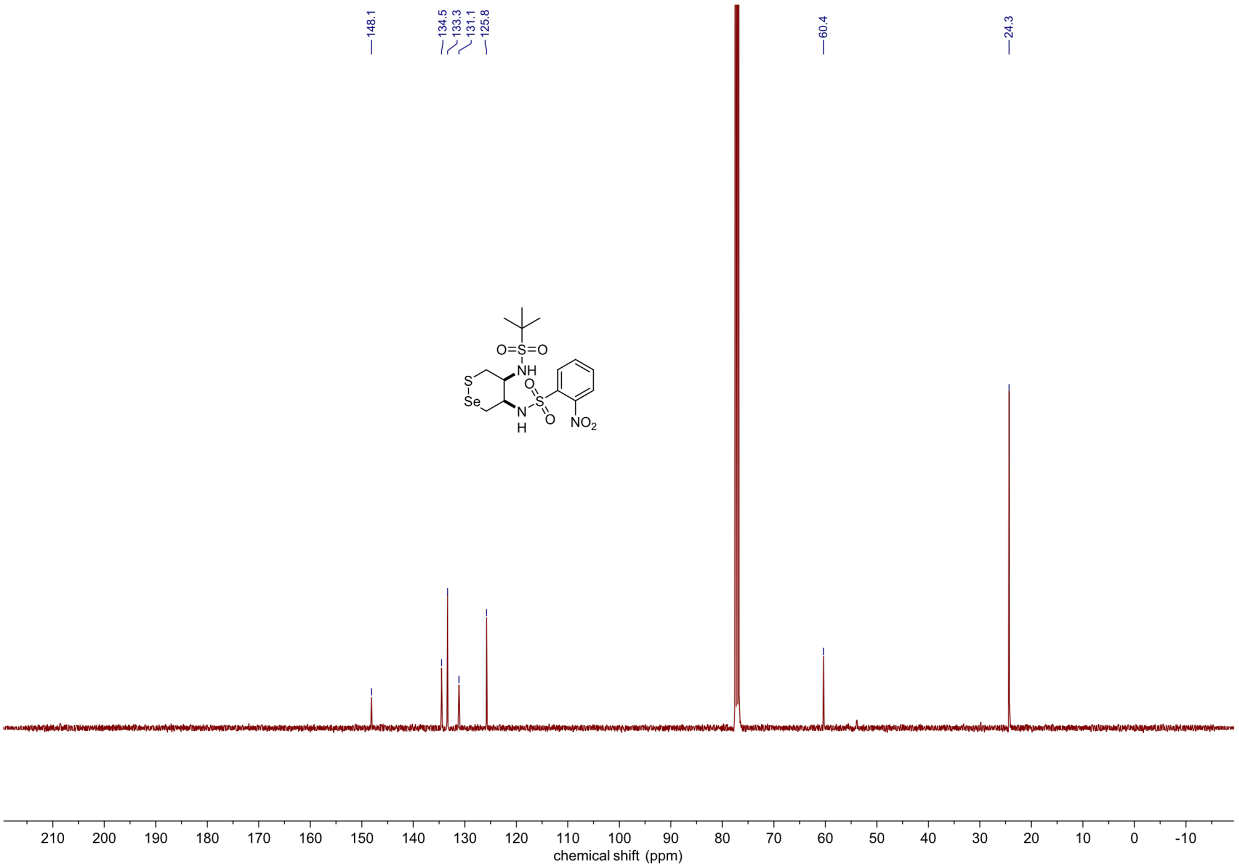

^{13}C NMR spectrum of **6**.

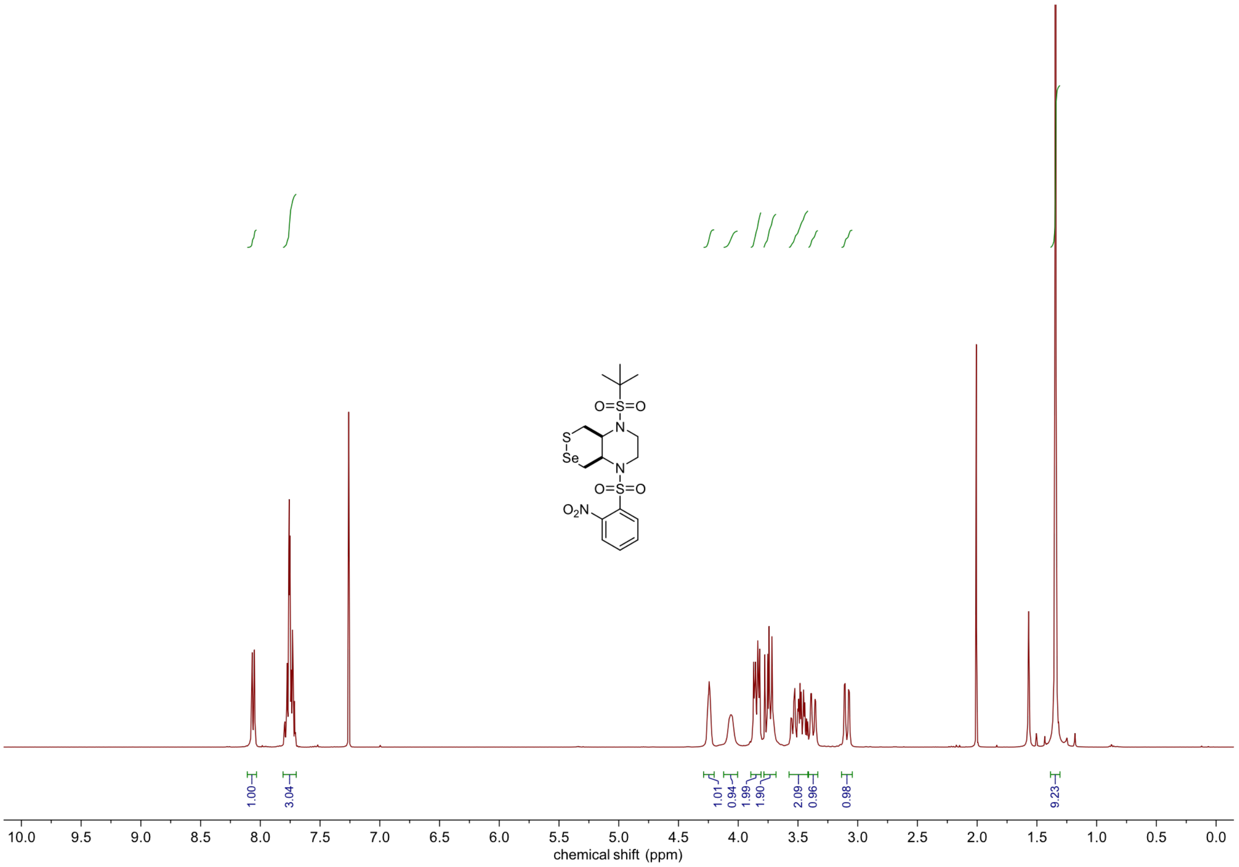

^{1}H NMR spectrum of **7**.

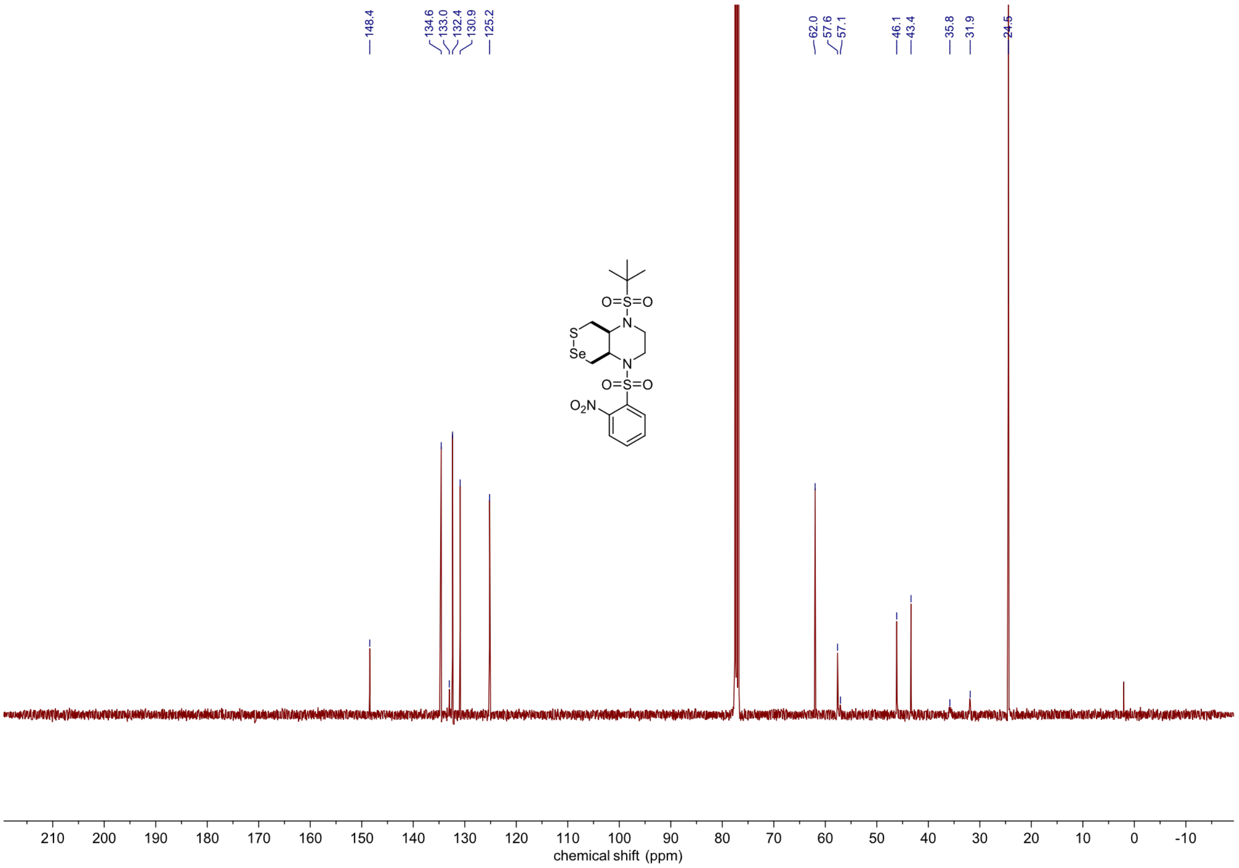

^{13}C NMR spectrum of **7**.

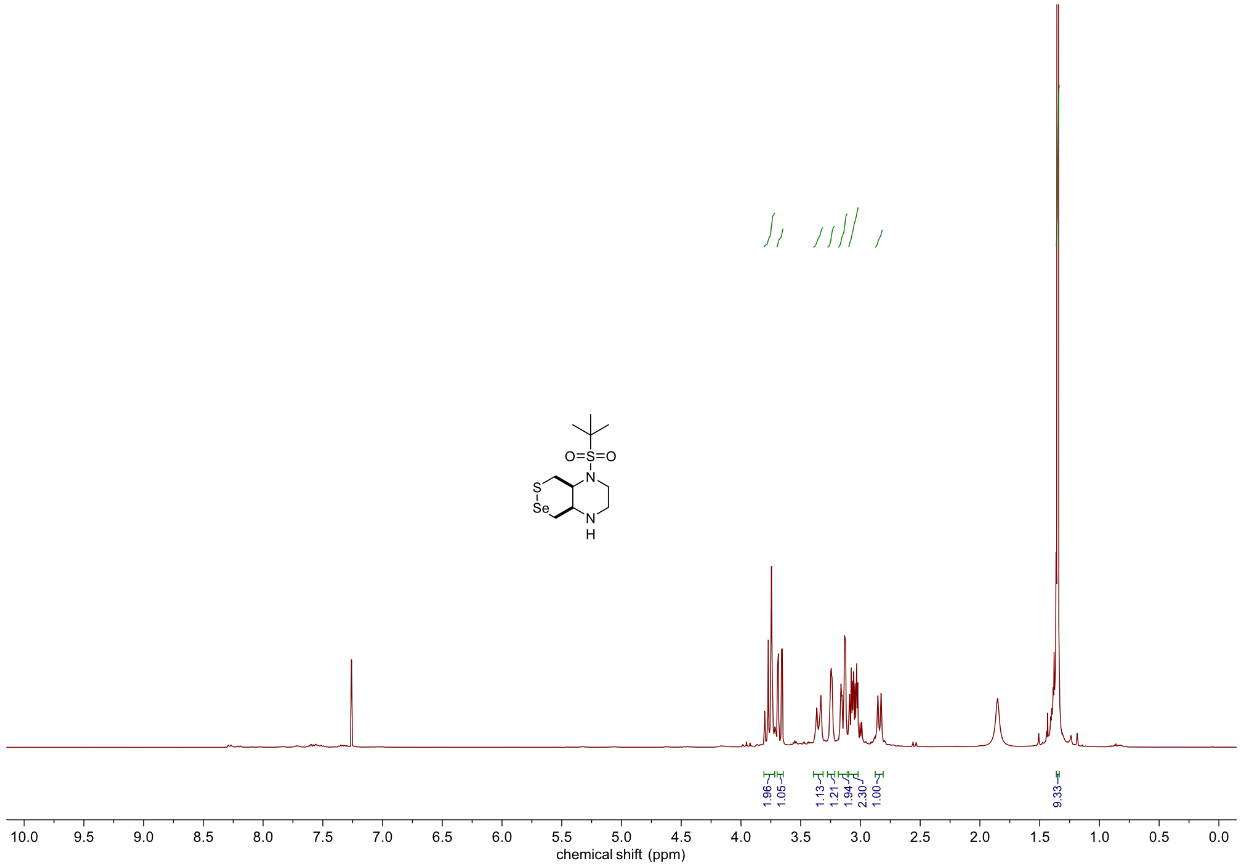

^{1}H NMR spectrum of **8**.

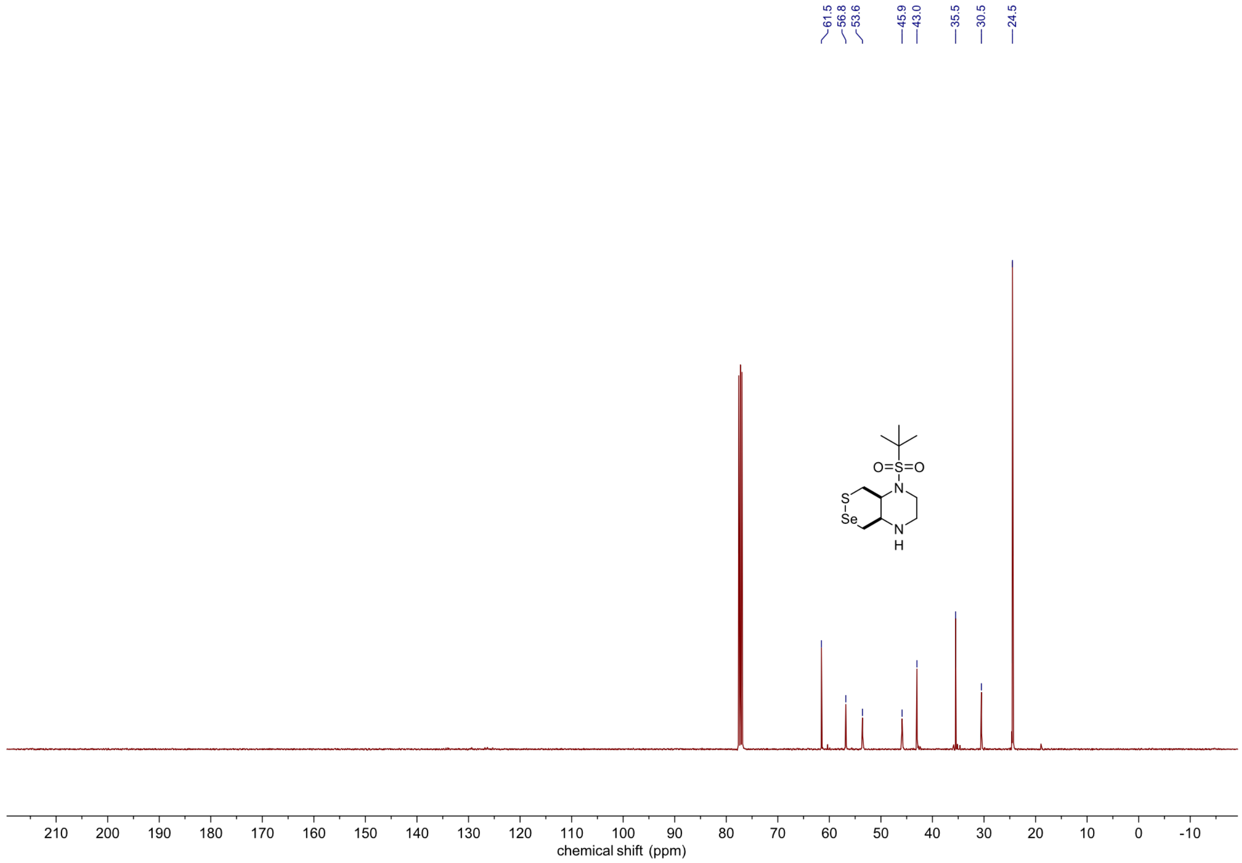

^{13}C NMR spectrum of **8**.

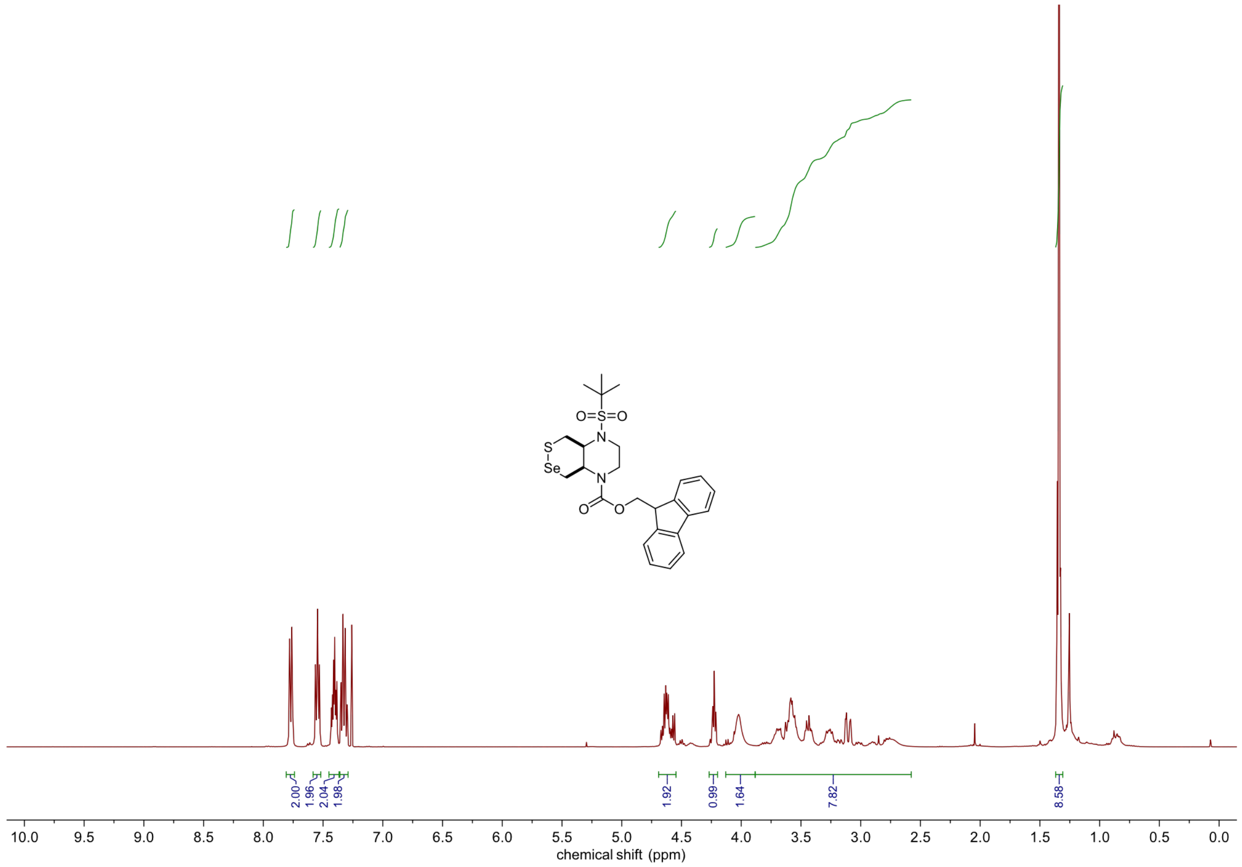

^{1}H NMR spectrum of **S14**.

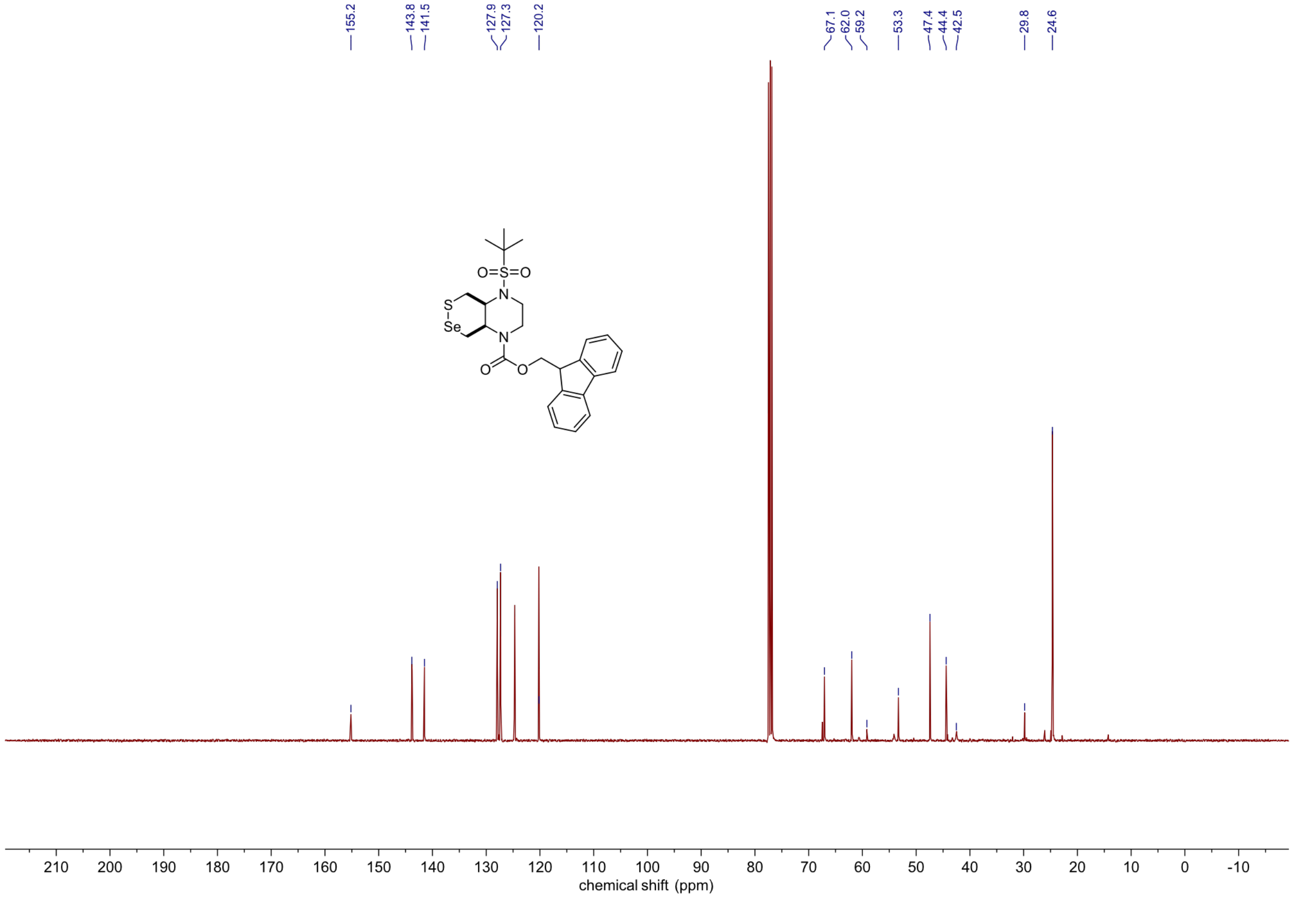

^{13}C NMR spectrum of **S14**.

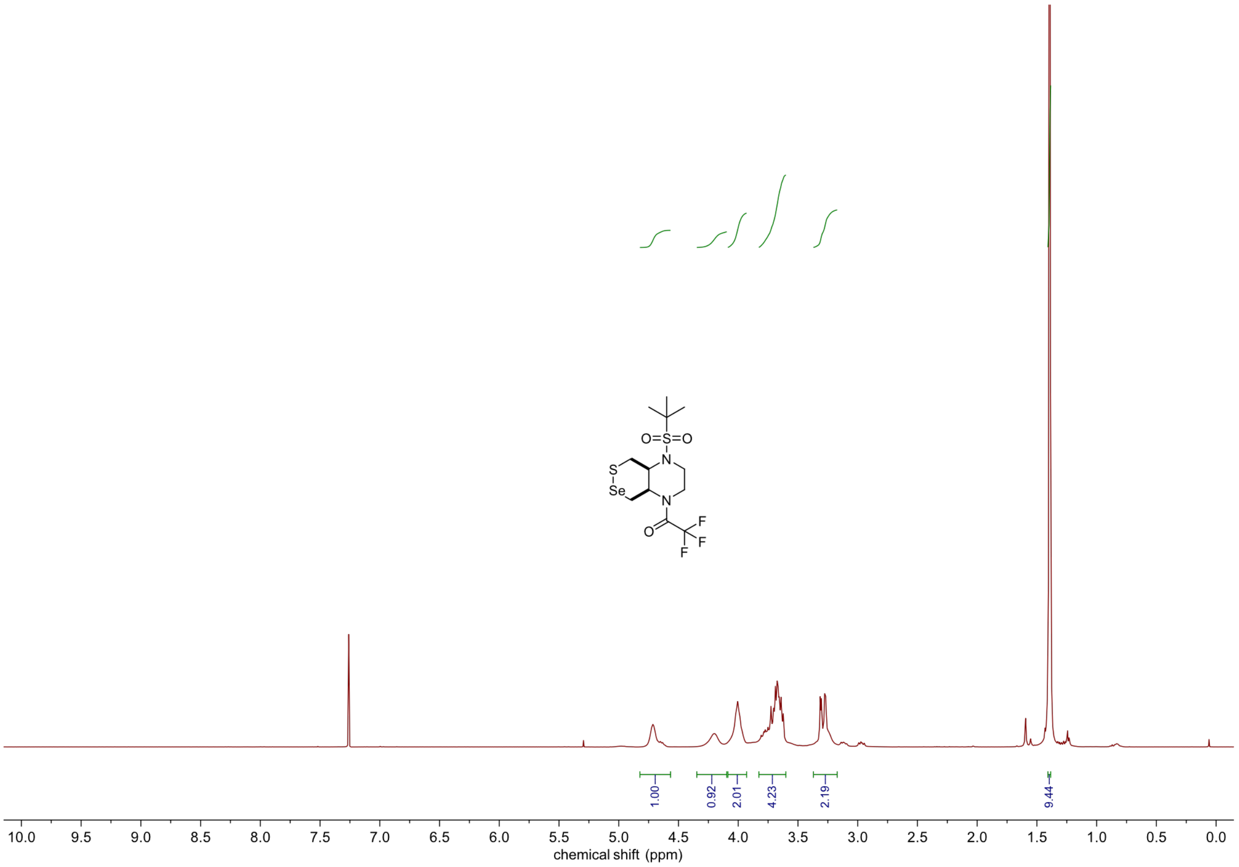

^{1}H NMR spectrum of **S15**.

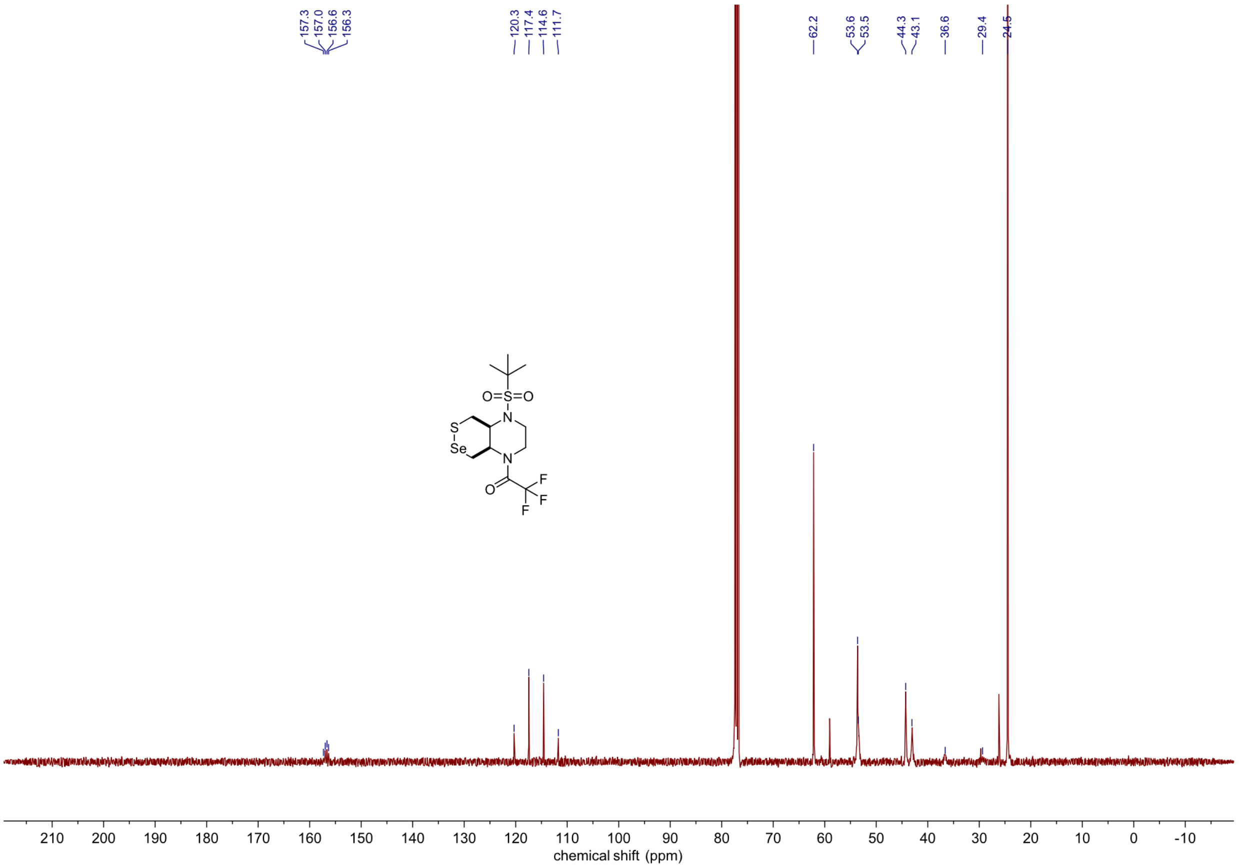

^{13}C NMR spectrum of **S15**.

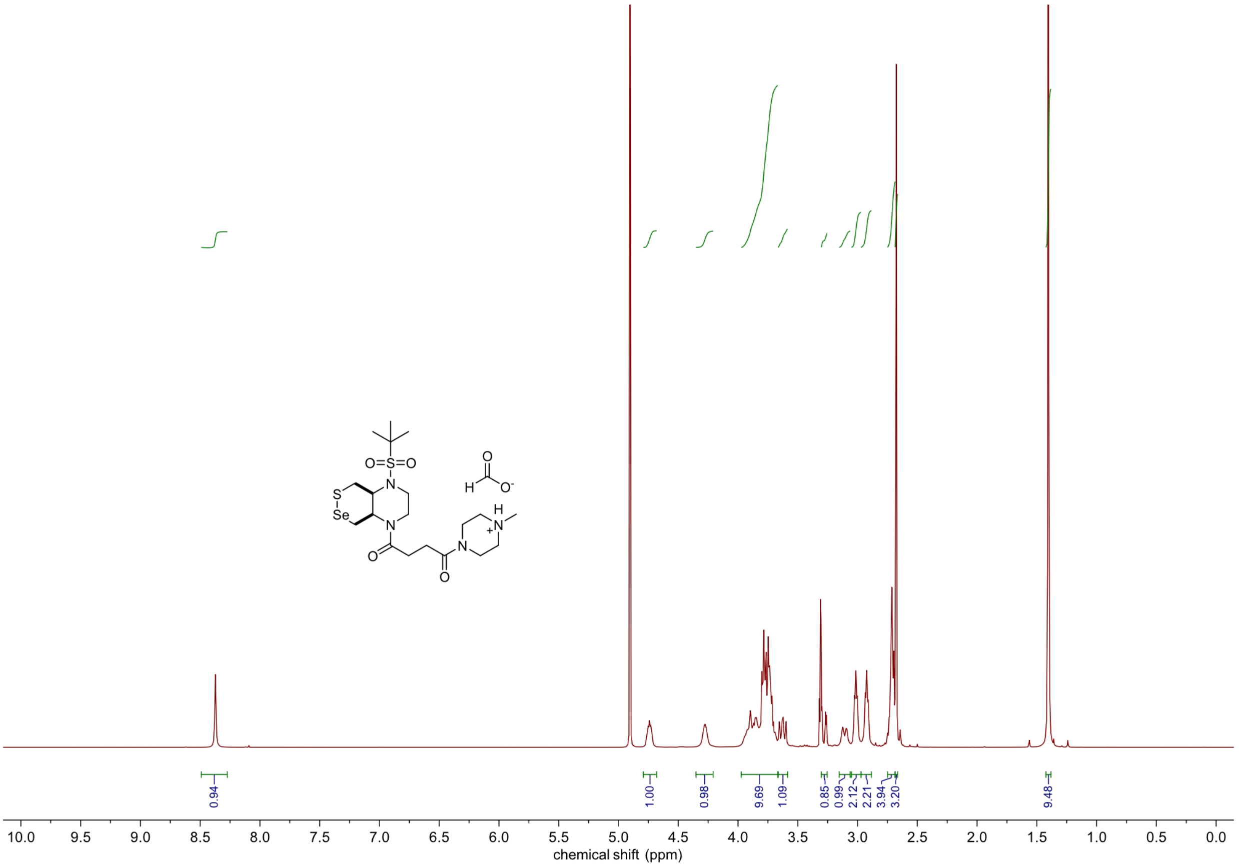

^{1}H NMR spectrum of **S16**.

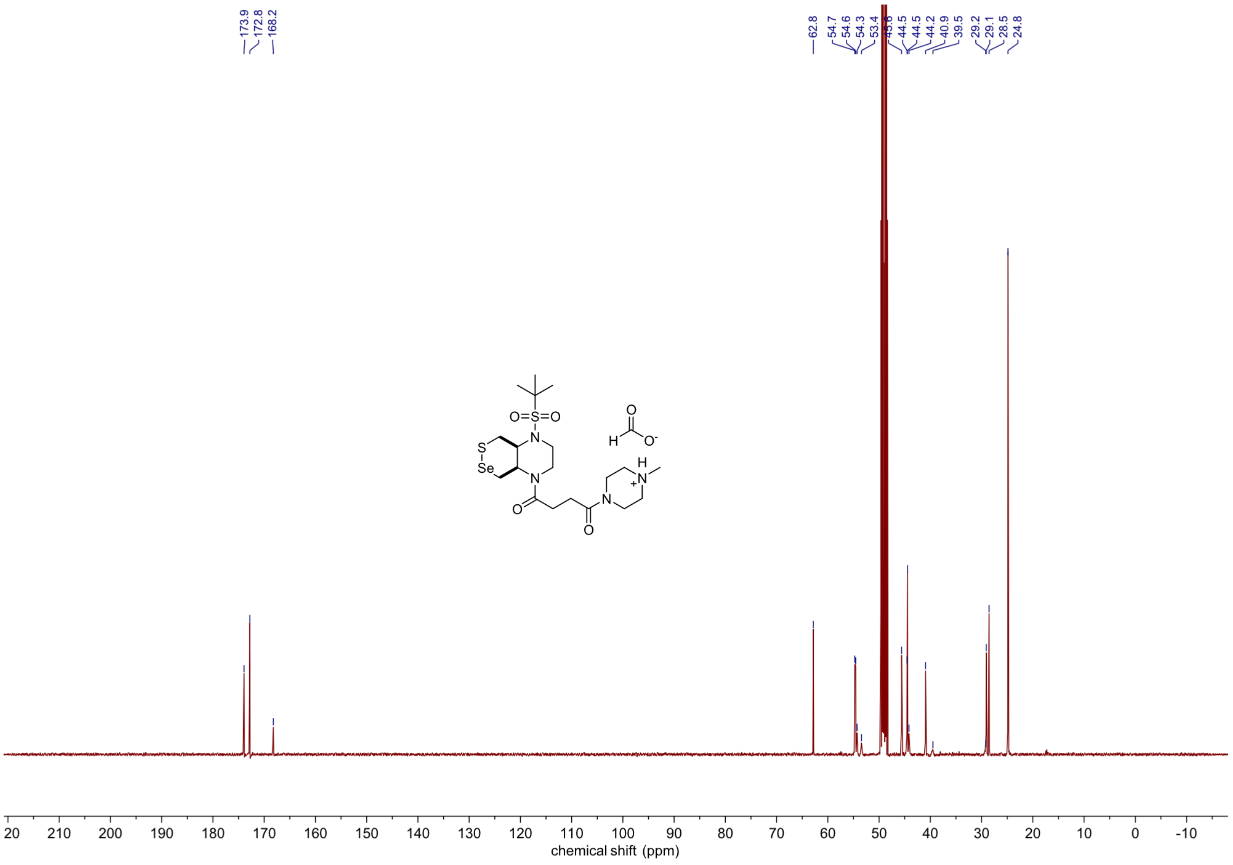

^{13}C NMR spectrum of **S16**.

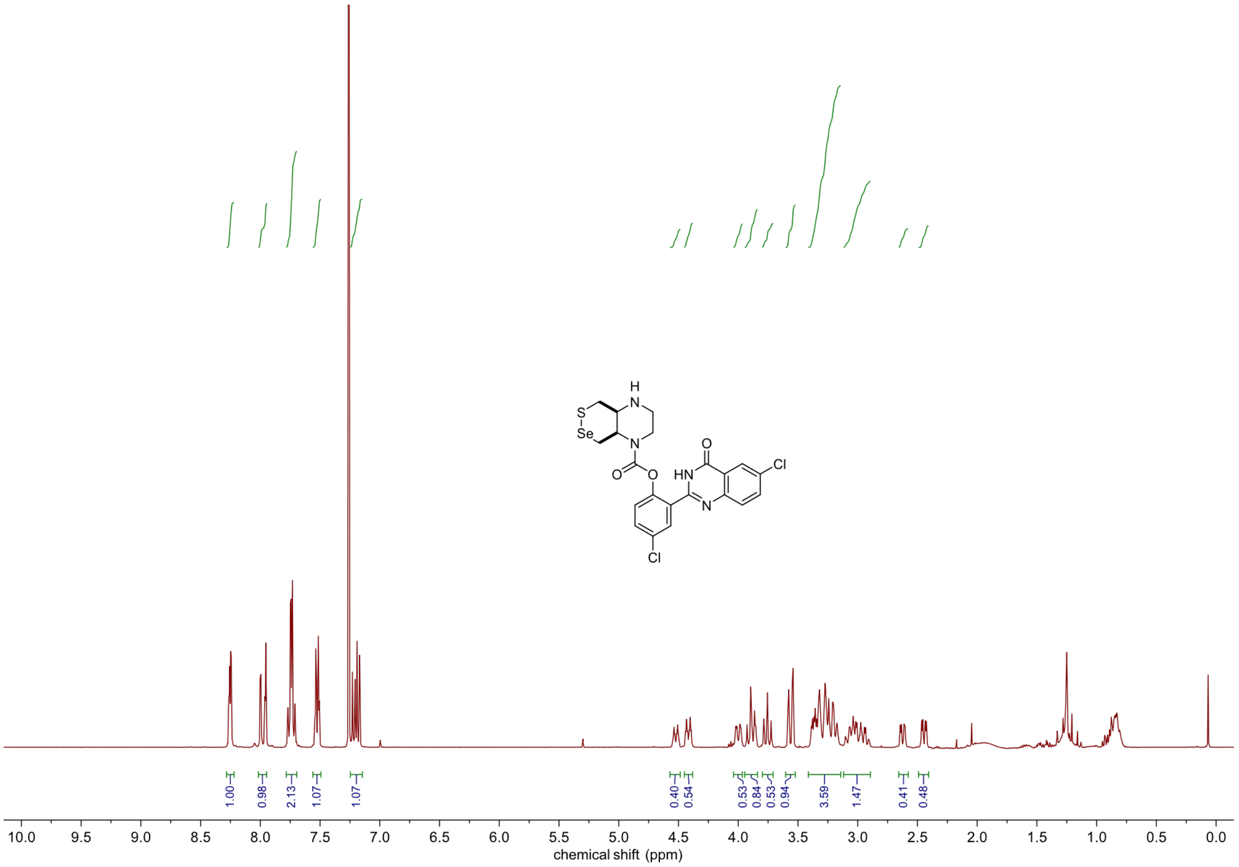

^{1}H NMR spectrum of **SeSC**.

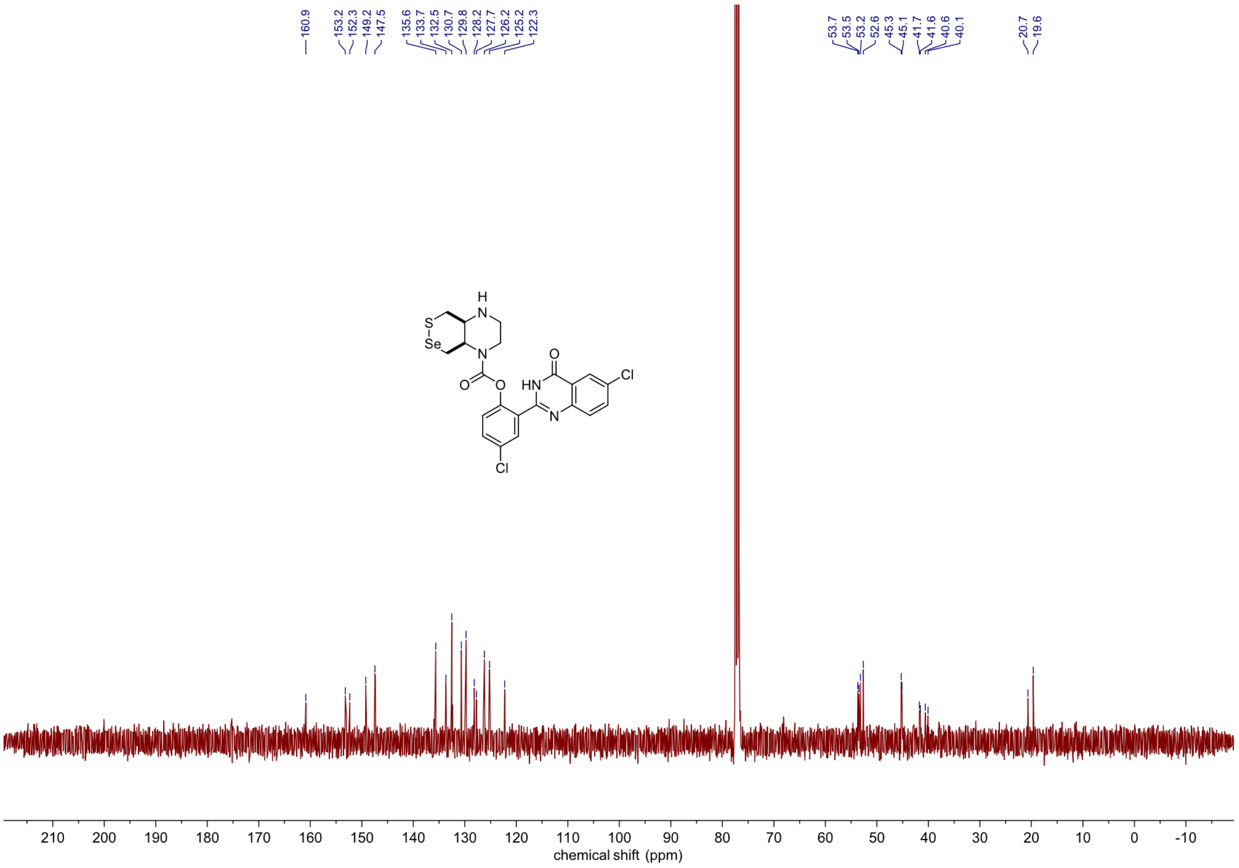

^{13}C NMR spectrum of **SeSC**.

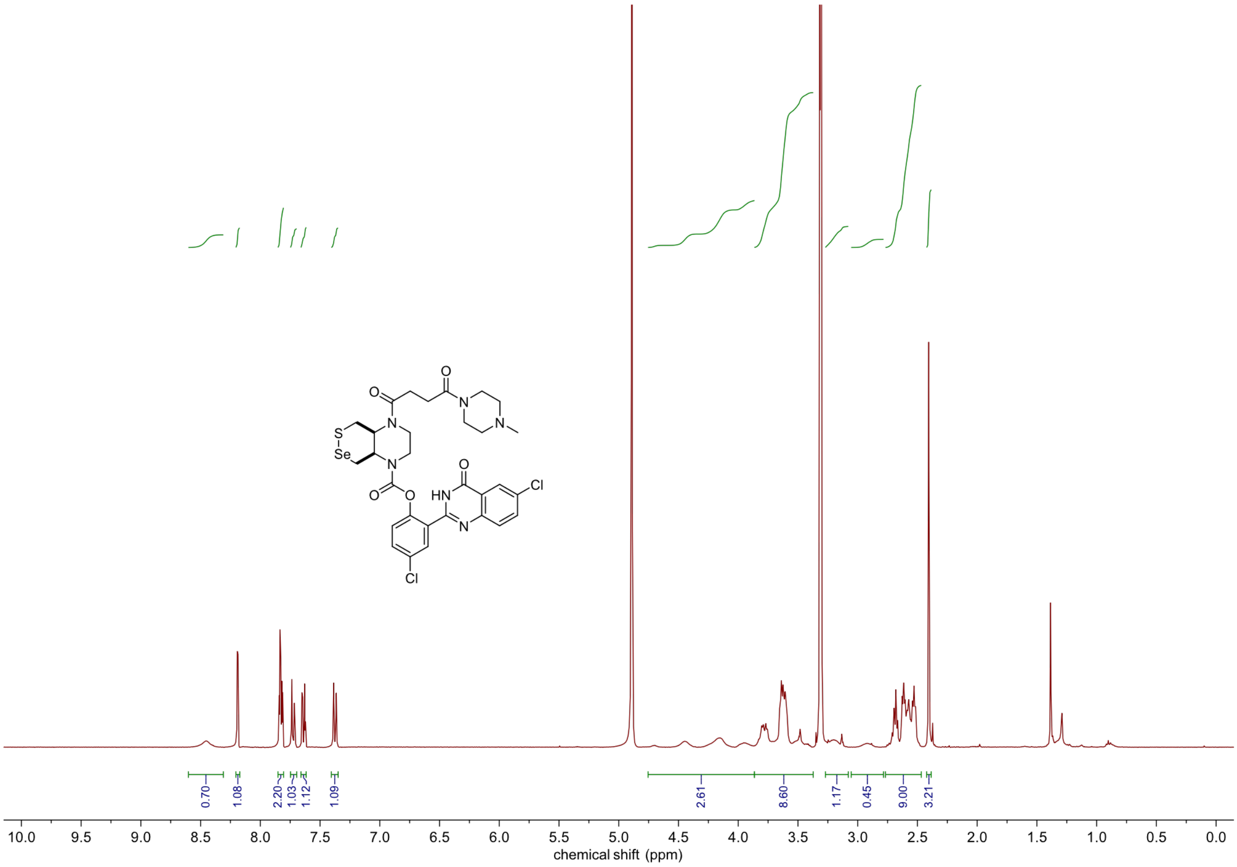

^{1}H NMR spectrum of **P-SeSC**.

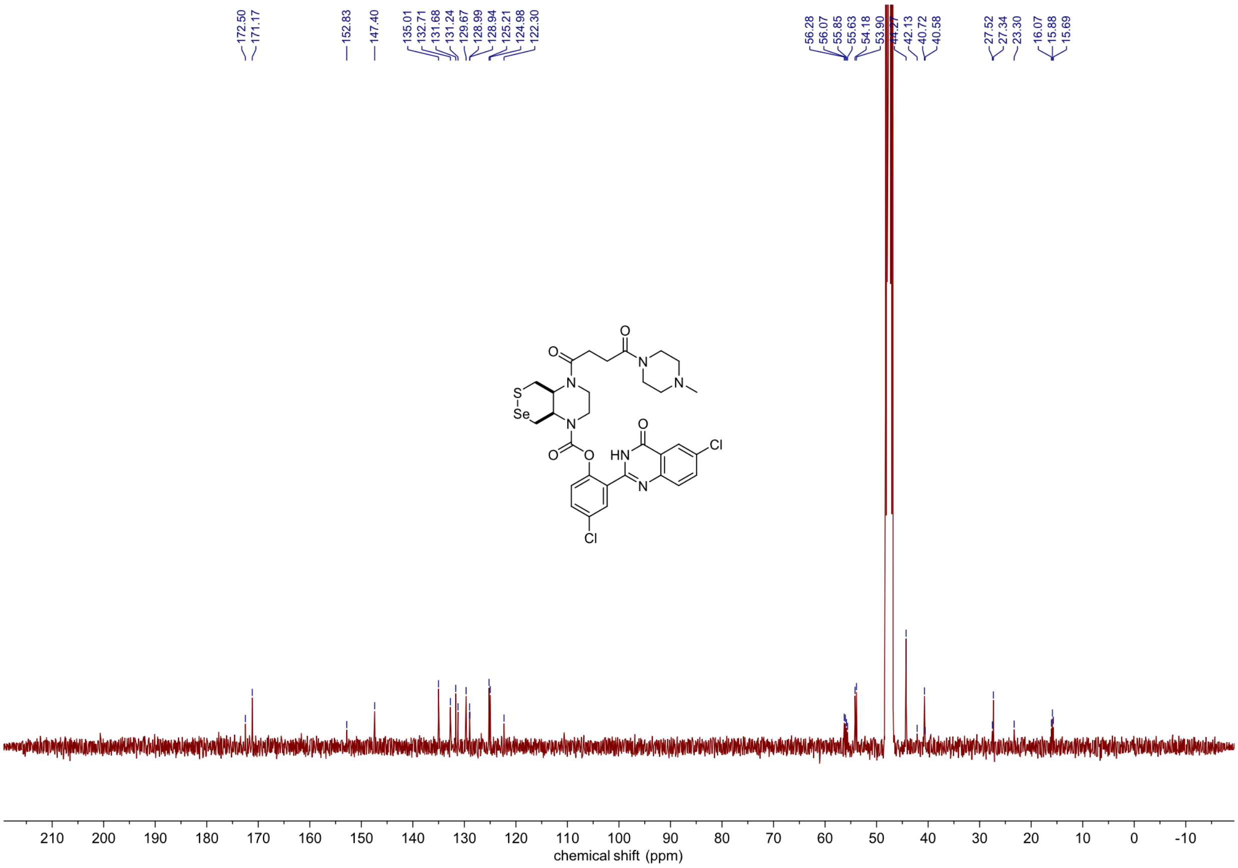

^{13}C NMR spectrum of **P-SeSC**.

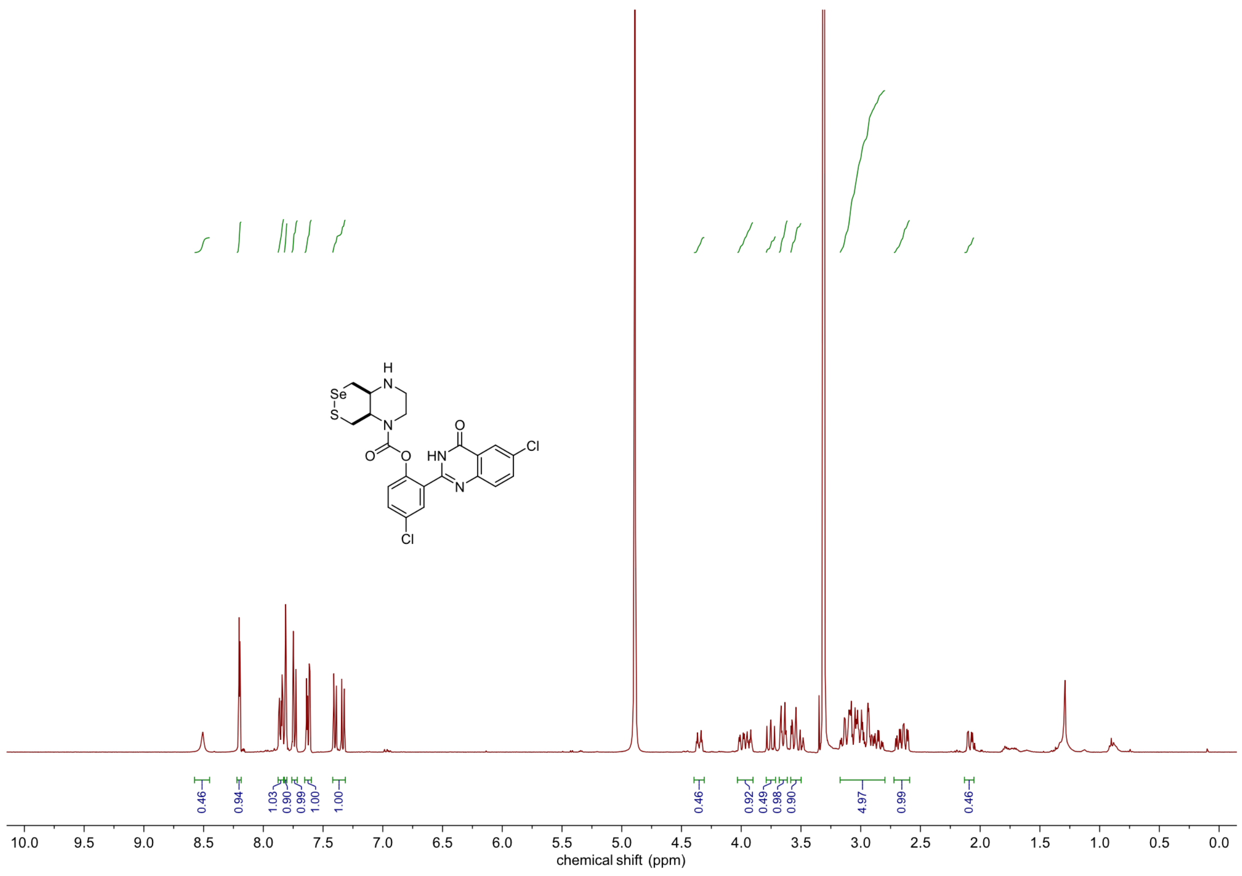

^{1}H NMR spectrum of **SSeC**.

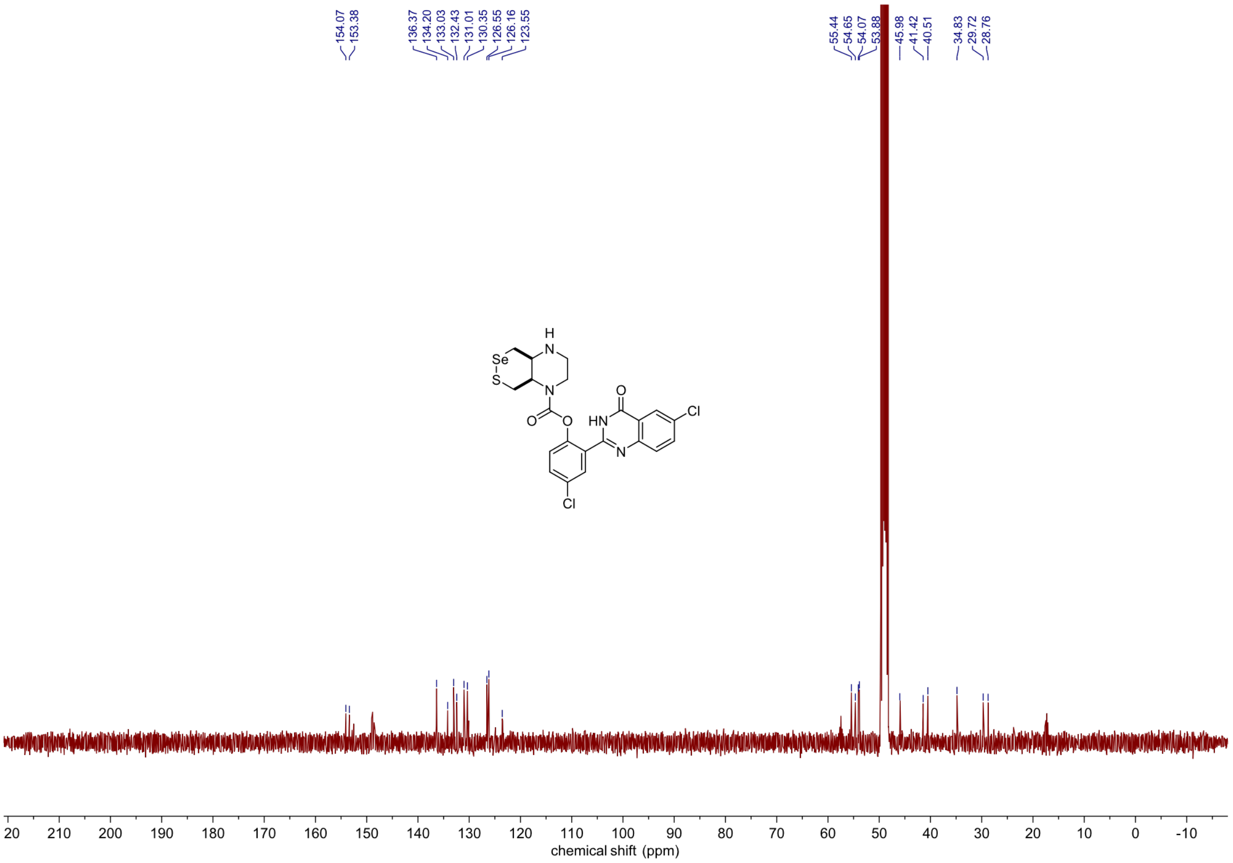

^{13}C NMR spectrum of **SSeC**.

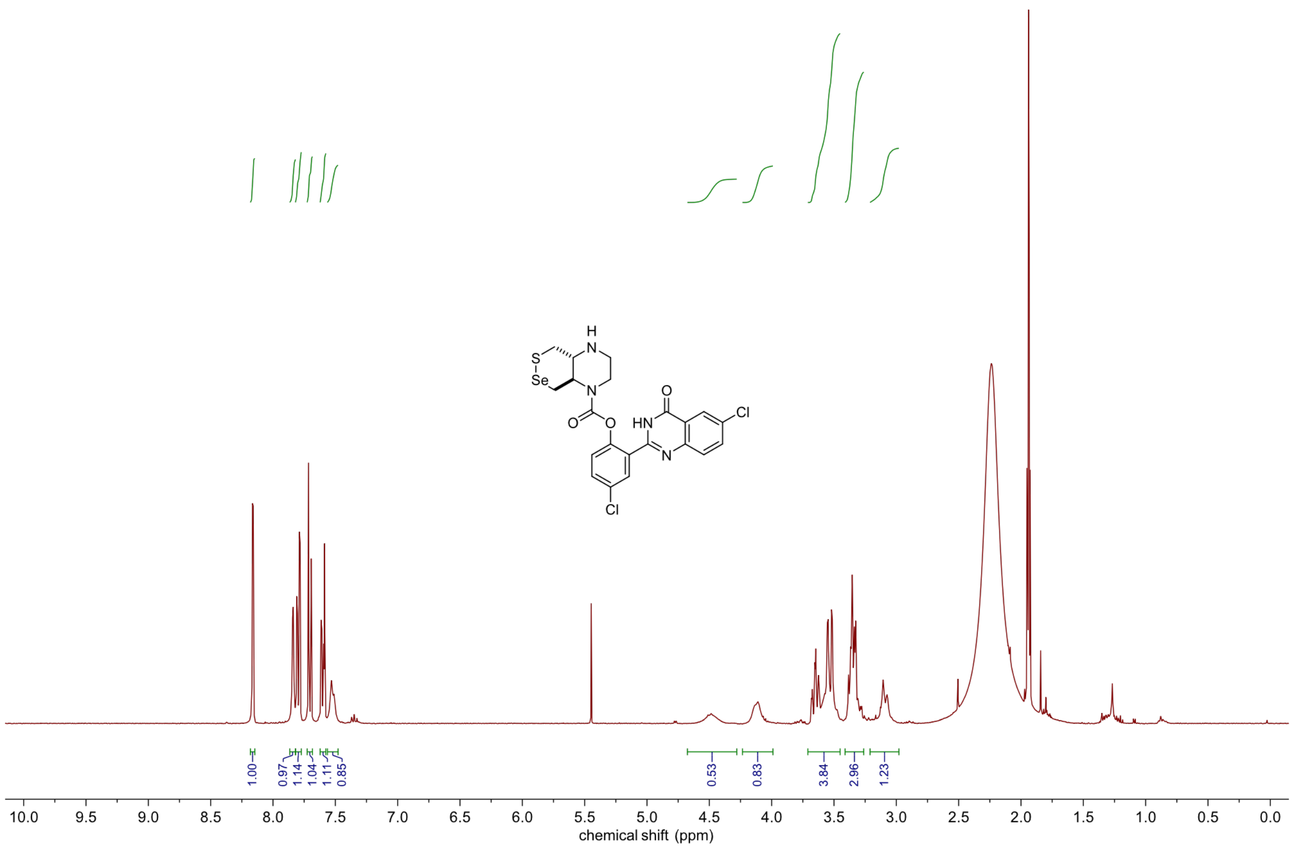

^{1}H NMR spectrum of **SeST**.

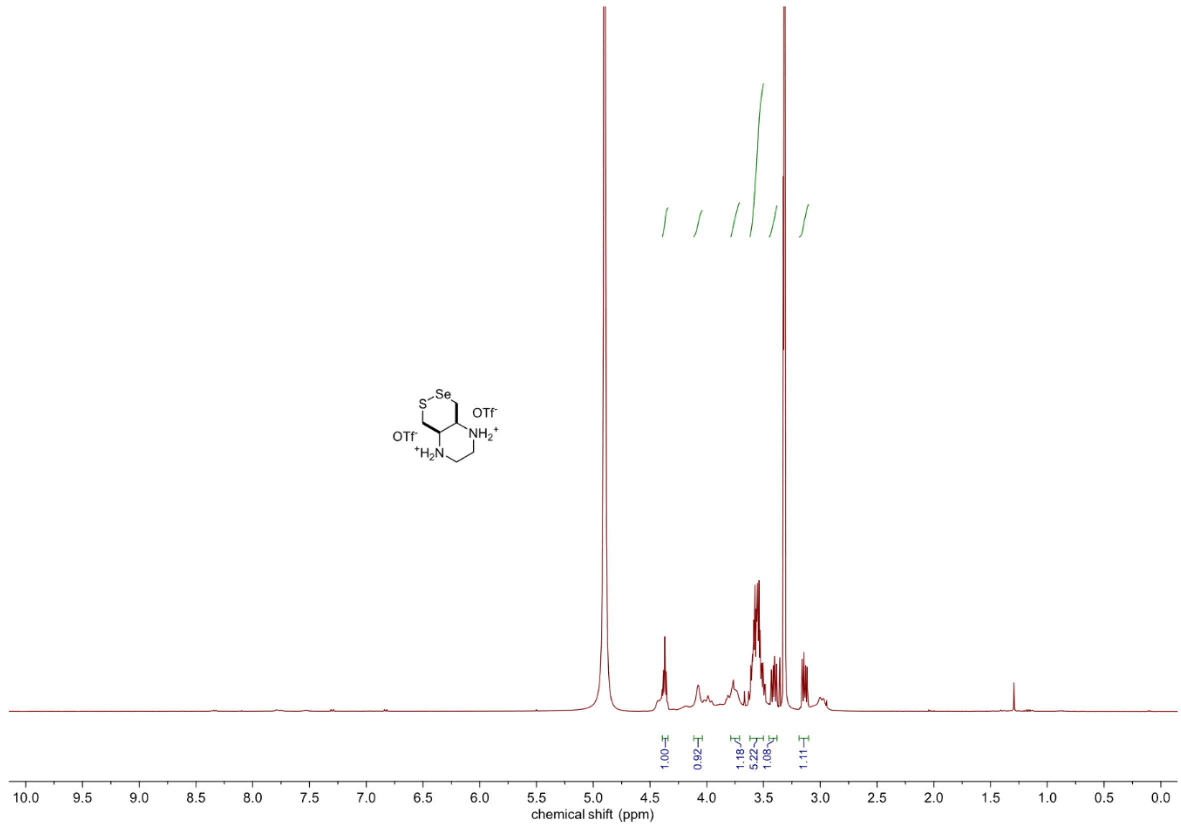

^{1}H NMR spectrum of **SeSC-N**.

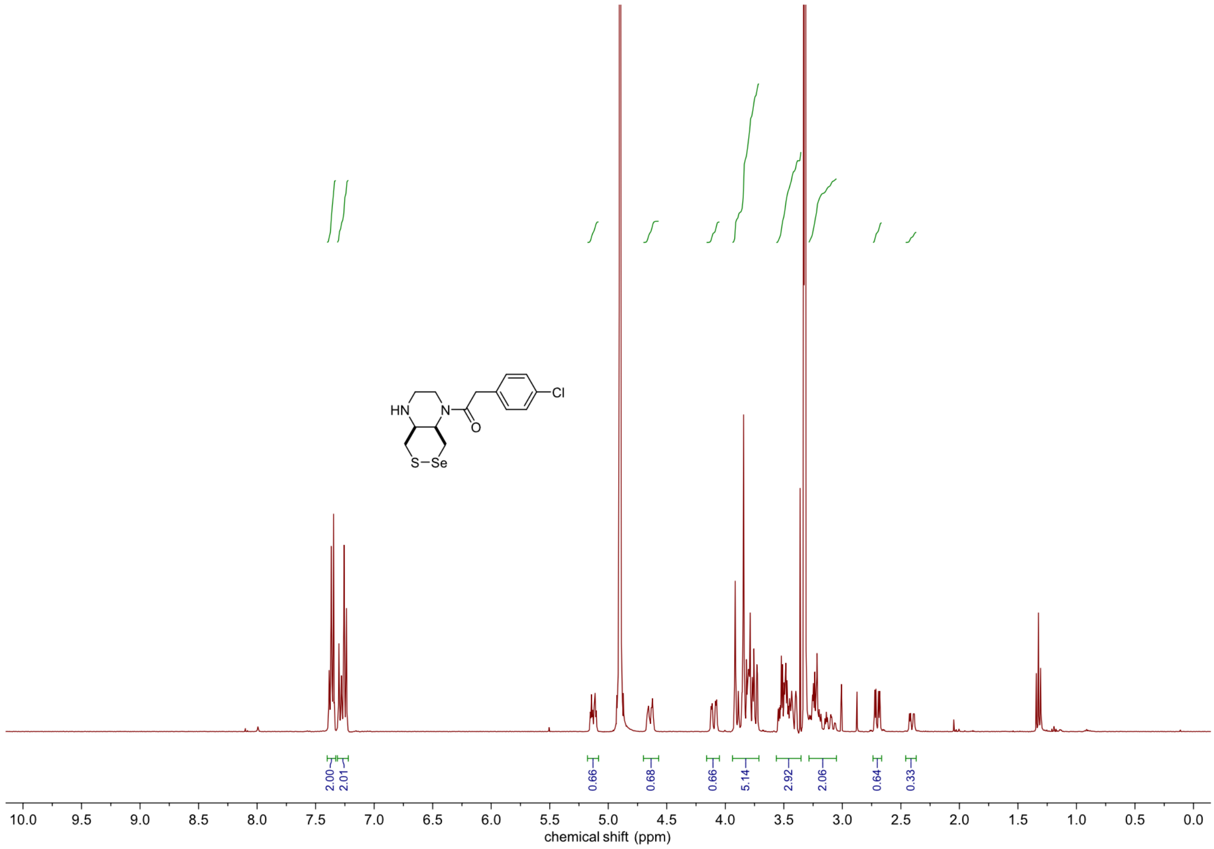

^{1}H NMR spectrum of **SeSC-D**.

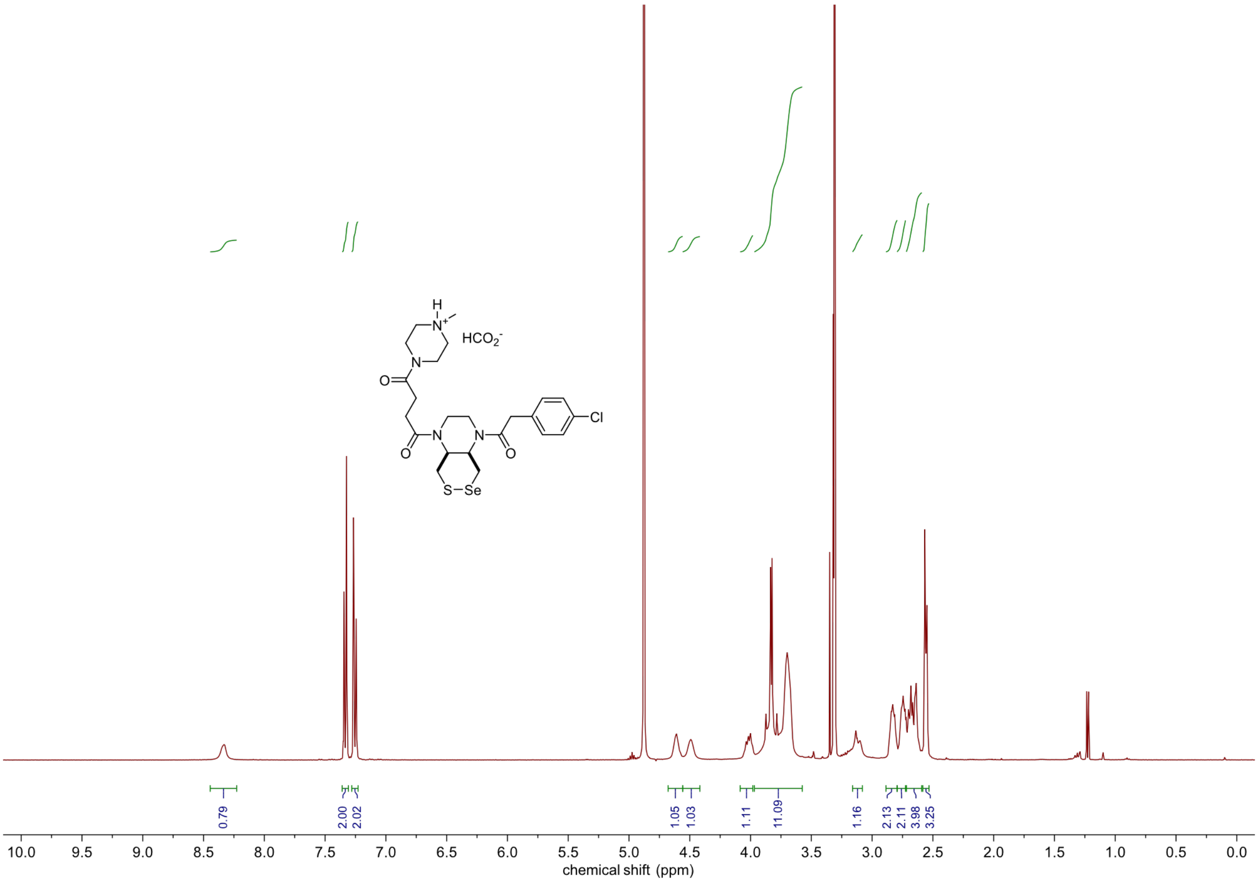

^{1}H NMR spectrum of **P-SeSC-D**.

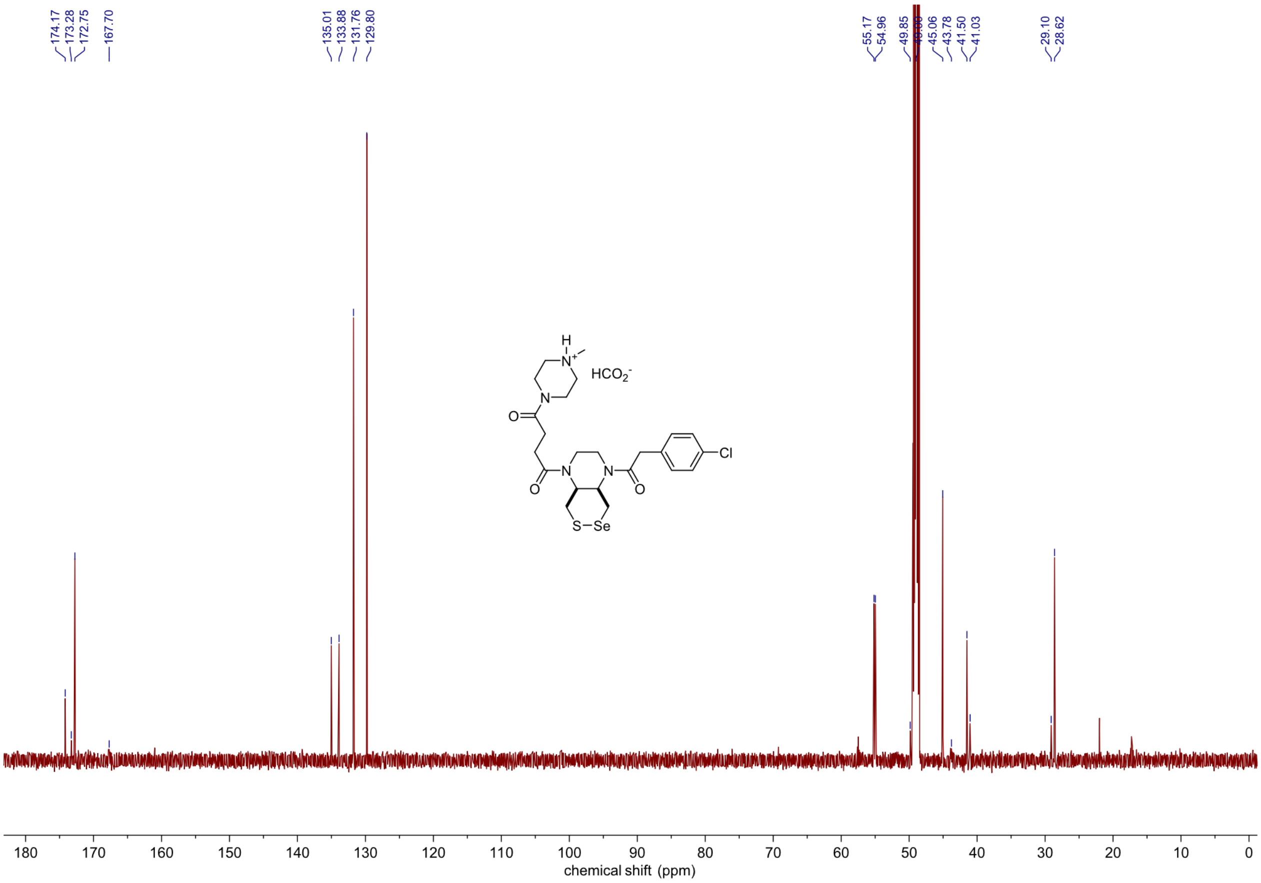

^{13}C NMR spectrum of **P-SeSC-D**.

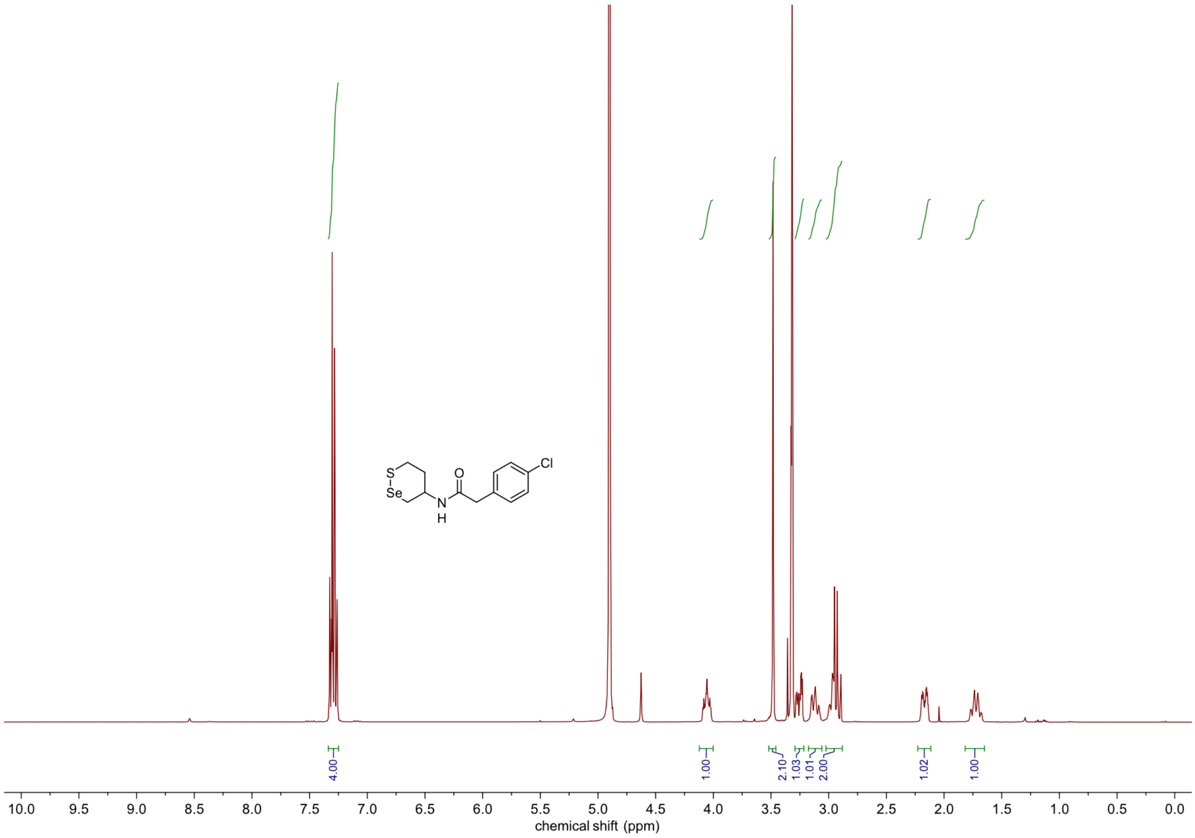

^{1}H NMR spectrum of **A3-D**.

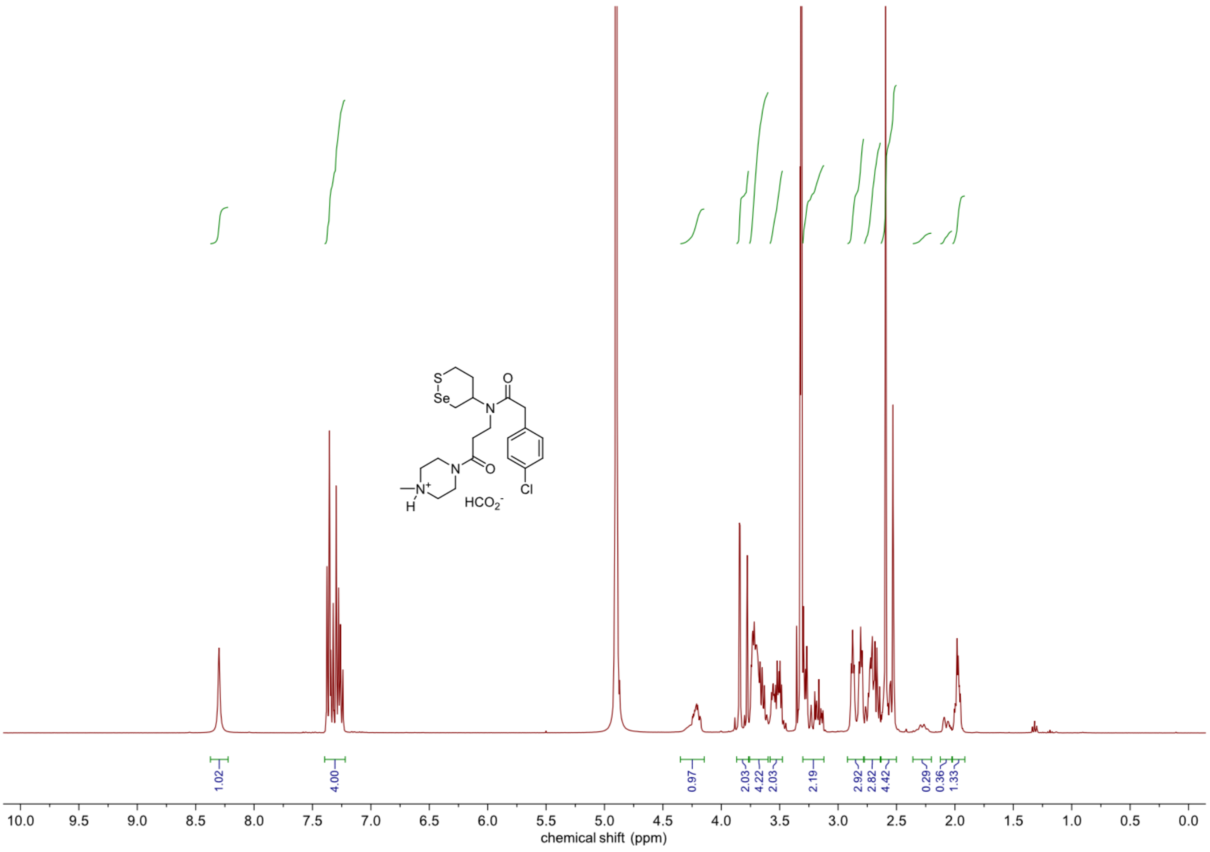

^{1}H NMR spectrum of **RX1-D**.

9 Supporting references

(1) Li, X.; Zhang, B.; Yan, C.; Li, J.; Wang, S.; Wei, X.; Jiang, X.; Zhou, P.; Fang, J. A Fast and Specific Fluorescent Probe for Thioredoxin Reductase That Works via Disulphide Bond Cleavage. *NComm* **2019**, *10* (1), 2745. https://doi.org/10.1038/s41467-019-10807-8.

(2) Dittert, L. W.; Higuchi, T. Rates of Hydrolysis of Carbamate and Carbonate Esters in Alkaline Solution. *Journal Of Pharmaceutical Sciences* **1963**, *52* (9), 852–857. https://doi.org/10.1002/jps.2600520908.

(3) Zeisel, L.; Felber, J. G.; Scholzen, K. C.; Poczka, L.; Cheff, D.; Maier, M. S.; Cheng, Q.; Shen, M.; Hall, M. D.; Arnér, E. S. J.; Thorn-Seshold, J.; Thorn-Seshold, O. Selective Cellular Probes for Mammalian Thioredoxin Reductase TrxR1: Rational Design of RX1, a Modular 1,2-Thiaselenane Redox Probe. *Chem* **2022**, *8* (5), 1493–1517. https://doi.org/10.1016/j.chempr.2022.03.010.

(4) Fosnacht, K. G.; Pluth, M. D. Activity-Based Fluorescent Probes for Hydrogen Sulfide and Related Reactive Sulfur Species. *Chemical Reviews* **2024**, *124* (7), 4124–4257. https://doi.org/10.1021/acs.chemrev.3c00683.

(5) Brigelius-Flohé, R. Tissue-Specific Functions of Individual Glutathione Peroxidases. *Free Radical Biology Medicine* **1999**, *27* (9–10), 951–965. https://doi.org/10.1016/S0891-5849(99)00173-2.

(6) Begas, P.; Liedgens, L.; Moseler, A.; Meyer, A. J.; Deponte, M. Glutaredoxin Catalysis Requires Two Distinct Glutathione Interaction Sites. *NComm* **2017**, *8* (1), 14835. https://doi.org/10.1038/ncomms14835.

(7) Handy, N. C.; Cohen, A. J. Left-Right Correlation Energy. *Molecular Physics* **2001**, *99* (5), 403–412. https://doi.org/10.1080/00268970010018431.

(8) Lee, C.; Yang, W.; Parr, R. G. Development of the Colle-Salvetti Correlation-Energy Formula into a Functional of the Electron Density. *Physical Review B* **1988**, *37* (2), 785–789. https://doi.org/10.1103/PhysRevB.37.785.

(9) Zaccaria, F.; Wolters, L. P.; Fonseca Guerra, C.; Orian, L. Insights on Selenium and Tellurium Diaryldichalcogenides: A Benchmark DFT Study. *Journal Of Computational Chemistry* **2016**, *37* (18), 1672–1680. https://doi.org/10.1002/jcc.24383.

(10) Bortoli, M.; Wolters, L. P.; Orian, L.; Bickelhaupt, F. M. Addition–Elimination or Nucleophilic Substitution? Understanding the Energy Profiles for the Reaction of Chalcogenolates with Dichalcogenides. *Journal Of Chemical Theory Computation* **2016**, *12* (6), 2752–2761. https://doi.org/10.1021/acs.jctc.6b00253.

(11) Bortoli, M.; Zaccaria, F.; Tiezza, M. D.; Bruschi, M.; Guerra, C. F.; Bickelhaupt, F. M.; Orian, L. Oxidation of Organic Diselenides and Ditellurides by H2O2 for Bioinspired Catalyst Design. *Phys Chem Chem Phys* **2018**, *20* (32), 20874–20885. https://doi.org/10.1039/C8CP02748J.

(12) Antony, S.; Bayse, C. A. Modeling the Mechanism of the Glutathione Peroxidase Mimic Ebselen. *Inorganic Chemistry* **2011**, *50* (23), 12075–12084. https://doi.org/10.1021/ic201603v.

(13) Masuda, R.; Kuwano, S.; Sase, S.; Bortoli, M.; Madabeni, A.; Orian, L.; Goto, K. Model Study on the Catalytic Cycle of Glutathione Peroxidase Utilizing Selenocysteine-Containing Tripeptides: Elucidation of the Protective Bypass Mechanism Involving Selenocysteine Selenenic Acids. *Bull Chem Soc Jpn* **2022**, *95* (9), 1360–1379. https://doi.org/10.1246/bcsj.20220156.

(14) Grimme, S. Density Functional Theory with London Dispersion Corrections. *WIREs Computational Molecular Science* **2011**, *1* (2), 211–228. https://doi.org/10.1002/wcms.30.

(15) Grimme, S.; Antony, J.; Schwabe, T.; Mück-Lichtenfeld, C. Density Functional Theory with Dispersion Corrections for Supramolecular Structures, Aggregates, and Complexes of (Bio)Organic Molecules. *OBC* **2007**, *5* (5), 741–758. https://doi.org/10.1039/B615319B.

(16) Johnson, E. R.; Mackie, I. D.; DiLabio, G. A. Dispersion Interactions in Density-Functional Theory. *Journal Of Physical Organic Chemistry* **2009**, *22* (12), 1127–1135. https://doi.org/10.1002/poc.1606.

(17) Johnson, E. R.; Becke, A. D. A Post-Hartree–Fock Model of Intermolecular Interactions. *J Chem Phys* **2005**, *123* (2), 024101. https://doi.org/10.1063/1.1949201.

(18) Klamt, A.; Schüürmann, G. COSMO: A New Approach to Dielectric Screening in Solvents with Explicit Expressions for the Screening Energy and Its Gradient. *Journal Of Chemical Society Perkin Transactions 2* **1993**, No. 5, 799–805. https://doi.org/10.1039/P29930000799.

(19) Pye, C. C.; Ziegler, T. An Implementation of the Conductor-like Screening Model of Solvation within the Amsterdam Density Functional Package. *Theoretical Chemistry Accounts* **1999**, *101* (6), 396–408. https://doi.org/10.1007/s002140050457.

(20) Ho, J.; Klamt, A.; Coote, M. L. Comment on the Correct Use of Continuum Solvent Models. *JPC-A* **2010**, *114* (51), 13442–13444. https://doi.org/10.1021/jp107136j.

(21) Grimme, S.; Bannwarth, C.; Shushkov, P. A Robust and Accurate Tight-Binding Quantum Chemical Method for Structures, Vibrational Frequencies, and Noncovalent Interactions of Large Molecular Systems Parametrized for All

Spd-Block Elements (Z = 1–86). *Journal Of Chemical Theory Computation* **2017**, *13* (5), 1989–2009. https://doi.org/10.1021/acs.jctc.7b00118.

(22) Pracht, P.; Bohle, F.; Grimme, S. Automated Exploration of the Low-Energy Chemical Space with Fast Quantum Chemical Methods. *Phys Chem Chem Phys* **2020**, *22* (14), 7169–7192. https://doi.org/10.1039/C9CP06869D.

(23) Madabeni, A.; Zeisel, L.; Thorn-Seshold, O.; Orian, L. Selenium Nucleophilicity and Electrophilicity in the Intra- and Intermolecular SN2 Reactions of Selenenyl Sulfide Probes. *Chemistry – A European Journal* **2025**.

(24) Singh, R.; Whitesides, G. M. Degenerate Intermolecular Thiolate-Disulfide Interchange Involving Cyclic Five-Membered Disulfides Is Faster by .Apprx.103 than That Involving Six- or Seven-Membered Disulfides. *JACS* **1990**, *112* (17), 6304–6309. https://doi.org/10.1021/ja00173a018.

(25) Pappas, J. A. Theoretical Studies of the Reactions of the Sulfur-Sulfur Bond. 1. General Heterolytic Mechanisms. *JACS* **1977**, *99* (9), 2926–2930. https://doi.org/10.1021/ja00451a013.

(26) Patterson, G. H.; Knobel, S. M.; Arkhammar, P.; Thastrup, O.; Piston, D. W. Separation of the Glucose-Stimulated Cytoplasmic and Mitochondrial NAD(P)H Responses in Pancreatic Islet β Cells. *Proceedings Of National Academy Of Sciences* **2000**, *97* (10), 5203–5207. https://doi.org/10.1073/pnas.090098797.

(27) Dóka, É.; Pader, I.; Bíró, A.; Johansson, K.; Cheng, Q.; Ballagó, K.; Prigge, J. R.; Pastor-Flores, D.; Dick, T. P.; Schmidt, E. E.; Arnér, E. S. J.; Nagy, P. A Novel Persulfide Detection Method Reveals Protein Persulfide- and Polysulfide-Reducing Functions of Thioredoxin and Glutathione Systems. *Science Advances* **2016**, *2* (1), e1500968. https://doi.org/10.1126/sciadv.1500968.

(28) Cheng, Q.; Arnér, E. S. J. Overexpression of Recombinant Selenoproteins in E. Coli. In *Selenoproteins: Methods and Protocols*; Chavatte, L., Ed.; Methods in Molecular Biology; Springer: New York, NY, 2018; pp 231–240. https://doi.org/10.1007/978-1-4939-7258-6_17.

(29) Mandal, P. K.; Schneider, M.; Kolle, P.; Kuhlencordt, P.; Forster, H.; Beck, H.; Bornkamm, G. W.; Conrad, M. Loss of Thioredoxin Reductase 1 Renders Tumors Highly Susceptible to Pharmacologic Glutathione Deprivation. *Cancer Res* **2010**, *70* (22), 9505–9514. https://doi.org/10.1158/0008-5472.CAN-10-1509.

(30) Cheng, Q.; Antholine, W. E.; Myers, J. M.; Kalyanaraman, B.; Arnér, E. S. J.; Myers, C. R. The Selenium-Independent Inherent Pro-Oxidant NADPH Oxidase Activity of Mammalian Thioredoxin Reductase and Its Selenium-Dependent Direct Peroxidase Activities*. *J Biol Chem* **2010**, *285* (28), 21708–21723. https://doi.org/10.1074/jbc.M110.117259.

(31) Zeisel, L.; Felber, J. G.; Scholzen, K. C.; Poczka, L.; Cheff, D.; Maier, M. S.; Cheng, Q.; Shen, M.; Hall, M. D.; Arnér, E. S. J.; Thorn-Seshold, J.; Thorn-Seshold, O. Selective Cellular Probes for Mammalian Thioredoxin Reductase TrxR1: Rational Design of RX1, a Modular 1,2-Thiaselenane Redox Probe. *Chem* **2022**, *8* (5), 1493–1517. https://doi.org/10.1016/j.chempr.2022.03.010.

(32) Stafford, W. C.; Peng, X.; Olofsson, M. H.; Zhang, X.; Luci, D. K.; Lu, L.; Cheng, Q.; Trésaugues, L.; Dexheimer, T. S.; Coussens, N. P.; Augsten, M.; Ahlzén, H.-S. M.; Orwar, O.; Östman, A.; Stone-Elander, S.; Maloney, D. J.; Jadhav, A.; Simeonov, A.; Linder, S.; Arnér, E. S. J. Irreversible Inhibition of Cytosolic Thioredoxin Reductase 1 as a Mechanistic Basis for Anticancer Therapy. *SciTransMed* **2018**, *10* (428), eaaf7444. https://doi.org/10.1126/scitranslmed.aaf7444.

(33) Sheldrick, G. M. SHELXT – Integrated Space-Group and Crystal-Structure Determination. *Acta Crystallographica Section A Foundations Advances* **2015**, *71* (1), 3–8. https://doi.org/10.1107/S2053273314026370.

(34) Farrugia, L. J. WinGX and ORTEP for Windows: An Update. *Journal Of Applied Crystallography* **2012**, *45* (4), 849–854. https://doi.org/10.1107/S0021889812029111.

(35) Gontcharov, A. V.; Liu, H.; Sharpless, K. B. Tert-Butylsulfonamide. A New Nitrogen Source for Catalytic Aminohydroxylation and Aziridination of Olefins. *OL* **1999**, *1* (5), 783–786. https://doi.org/10.1021/ol990761a.

(36) Zeisel, L.; Maier, M. S.; Thorn-Seshold, O. Efficient and Scalable Syntheses of 1,2-Thiaselenane-4-Amine and 1,2-Thiaselenane-5-Amine. *Synthesis* **2023**, *55* (09), 1385–1393. https://doi.org/10.1055/a-2022-1398.

(37) Zeisel, L.; Felber, J. G.; Scholzen, K. C.; Schmitt, C.; Wiegand, A. J.; Komissarov, L.; Arnér, E. S. J.; Thorn-Seshold, O. Piperazine-Fused Cyclic Disulfides Unlock High-Performance Bioreductive Probes of Thioredoxins and Bifunctional Reagents for Thiol Redox Biology. *JACS* **2024**, *146* (8), 5204–5214. https://doi.org/10.1021/jacs.3c11153.

(38) Thorn-Seshold, O.; Vargas-Sanchez, M.; McKeon, S.; Hasserodt, J. A Robust, High-Sensitivity Stealth Probe for Peptidases. *Chemical Communications* **2012**, *48* (50), 6253–6255. https://doi.org/10.1039/C2CC32227G.
